# Burden of disease attributable to tobacco, high alcohol use, and drug use from 1990 to 2021

**DOI:** 10.3389/fnut.2026.1701968

**Published:** 2026-05-28

**Authors:** Wenshuai Zheng, Rui Qin, Mingjuan Liu, Kaidi Yang, Jiahang Li, Songsong Liu, Tao Wang, Xiaoning Gao

**Affiliations:** 1 Department of Hematology, Hainan Hospital of Chinese PLA General Hospital, Sanya, Hainan, China; 2 Department of Hematology, The Central Hospital of Enshi Tujia and Miao Autonomous Prefecture, Enshi, Hubei, China; 3 Senior Department of Hematology, Chinese PLA General Hospital, Beijing, China; 4 Department of Oncology, Hainan Hospital of Chinese PLA General Hospital, Sanya, Hainan, China; 5 Department of Rehabilitation Medicine, Hainan Hospital of Chinese PLA General Hospital, Sanya, Hainan, China; 6 Senior Department of Hepatobiliary Surgery, Hainan Hospital of Chinese PLA General Hospital, Sanya, Hainan, China; 7 Department of Emergency, Hainan Hospital of Chinese PLA General Hospital, Sanya, Hainan, China; 8 State Key Laboratory of Experimental Hematology, Senior Department of Hematology, Chinese PLA General Hospital, Beijing, China

**Keywords:** burden, drug use, epidemiology, Global Burden of Disease Study, high alcohol use, substance use, tobacco

## Abstract

**Background:**

Substance use, including tobacco, high alcohol use, and drug use, has negative consequences on the health, economy, productivity, and social aspects of communities. Understanding trends in the burden by age, over time, and location and sex is important for policymaking. This study aims to provide a comprehensive estimation of the global burden attributable to substance use.

**Methods:**

Using data from the Global Burden of Disease study (GBD) 2021, we estimated the burden attributable to substance use in terms of the number and age-standardized rates (ASRs) of deaths and disability-adjusted life years (DALYs) from 1990 to 2021. Using histogram plots, world maps, and Pearson’s correlation analysis, we conducted a stratified analysis of substance use by sex, age, geographic location, sociodemographic index (SDI) level, and disease. Additionally, the Bayesian model for age-period-cohort was introduced to forecast the burden.

**Results:**

In 2021, substance use was responsible for a substantial burden globally. From 1990 to 2021, the number of deaths and DALYs increased for nearly all substance use subtypes, while the age-standardized death rate (ASDR) and age-standardized DALY rate (ASDALYR) decreased. Furthermore, trends varied significantly by age, sex, geographic region, and SDI. The ASRs for all substance use subtypes were higher in male individuals than in female individuals, with the exception of secondhand smoke. Colder regions were associated with a higher burden attributable to high alcohol use, whereas the burden attributable to drug use increased with higher SDI levels. The burden attributable to chewing tobacco was notably more pronounced in South Asia than in other regions. Drug use disproportionately affected the 20–45 years age group. Projections indicate that the burden attributable to drug use and chewing tobacco will increase consistently from 2022 to 2040.

**Conclusion:**

Substance use is an important contributor to the global disease burden. Population-specific interventions, including raising taxes, increasing price, formulating laws and regulations, and raising public awareness on the risks of substance use, should be implemented to prevent and reduce the substance use burden.

## Introduction

Substance use, including tobacco, high alcohol use, and drug use, collectively represents a major global risk factor for disability and premature mortality (1). Given their shared addictive nature, we classify them as substance use. The associated health burden results in substantial economic costs, including healthcare expenditures, law enforcement expenses, productivity losses, and other direct and indirect consequences (2, 3). Tobacco is a predominant risk factor for the development and progression of periodontal disease, cardiovascular disorders, respiratory conditions, and various cancers (4). Alcohol increases the risk of both unintentional and intentional injuries, as well as non-communicable disease and infectious diseases (5). Drug use may contribute to the onset of schizophrenia, psychosis, cardiovascular diseases, asthma, infections, and cognitive impairment (6–8).

Despite the global implementation of policies aimed at reducing the use of tobacco, alcohol, and drugs (9–11), substance use remains among the most pressing public health challenges worldwide. According to the World Health Organization (WHO), smoking is the world’s second-leading cause of mortality. Tobacco and its derivatives are anticipated to cause more than six million deaths annually worldwide. Cigarette smoking is also expected to cause more than eight million deaths per year by 2030 (12). Total alcohol per capita consumption in the world increased from 2010 to 2013, and then slightly declined until 2018, followed by a slight increase in 2019. Despite an overall reduction, some regions and individual countries increased their alcohol consumption between 2010 and 2019. The highest increases were found in the subregions of South Eastern Asia and Southern Europe (13). According to global data, the prevalence of drug use is rising, particularly in low- and middle-income countries (14). Therefore, accurate estimation of the burden attributable to substance use at country, regional, and global levels is essential for quantifying its severity. Such evidence is critical for guiding health behaviors, including abstaining from tobacco, alcohol, and drug, and guiding government, including policymaking, service planning, and policy evaluation.

The Global Burden of Disease Study (GBD) 2021, coordinated by the Institute for Health Metrics and Evaluation, provides comprehensive estimates of the burden of diseases, injuries, and risk factors worldwide. GBD 2021 incorporates tobacco, high alcohol use, and drug use as major risk factors and offers detailed data on their contributions to health loss by geographic location, age, and sex, thereby serving as a valuable resource for investigating the health effects of substance use. Although previous studies have utilized GBD data to examine substance use-related burden (4, 15–19), these studies have several limitations: (1) many studies rely on outdated estimates, (2) comprehensive assessments across different substance use subtypes remain limited, and (3) rare studies conduct future projections. Therefore, this study employs GBD 2021 data to estimate the global, regional, and national burden attributable to tobacco, high alcohol use, and drug use. In addition to separate analysis, this study also examines the interplay between different substance use subtypes. The findings aim to support the development of targeted, population-specific policies and interventions.

## Methods

### Data source and collection

We conducted a secondary analysis of the GBD 2021^1^. The GBD 2021 study provides an accessible epidemiological assessment of 371 diseases, injuries, and impairments, as well as 88 risk factors, encompassing 204 nations and territories (1, 20). Its extensive data sources include vital registration systems, epidemiological surveys, disease surveillance systems, cancer registries, police records, and open-source databases. Detailed methodologies for data collection and processing have been described in previous publications (1, 20, 21). Downstream data analysis is performed using advanced statistical models such as meta-regression Bayesian, regularized, trimmed, Disease Modeling Meta-Regression, version 2.1, and spatiotemporal Gaussian process regression. The project adhered to the Guidelines for Accurate and Transparent Health Estimates Reporting (22). The study did not involve any personal or sensitive information. Consequently, no ethics approval was required for the execution of this study.

This study utilized data from the GBD 2021 database, including the number of deaths and disability-adjusted life-years (DALYs) for substance use from 1990 to 2021, along with their corresponding age-standardized rates (ASRs). These metrics were extracted via the GBD visualization platform (see text footnote 1).

Moreover, this study utilized the sociodemographic index (SDI), a composite indicator based on estimates of total fertility rate in those younger than 25 years, mean years of education in individuals older than 15 years, and lag-distributed income per capita (23), to explore the relationship between the disease burden attributable to substance use and the development status of a region or country. The SDI ranges from 0 (less developed) to 1 (most developed). The 204 countries and territories were classified into five SDI regions based on SDI quintiles in 2021: low, low-middle, middle, high-middle, and high.

### Estimated burden attributable to substance use

GBD 2021 quantified tobacco, high alcohol use, and drug use as risk factors for other health outcomes in the comparative risk assessment. The definition of tobacco is comprehensive, including smoking, chewing tobacco, and secondhand smoke. The detailed definitions of every substance use subtype can been seen in Supplementary material. In the present study, we measured the burden attributable to substance use by deaths and DALYs. DALYs were the sum of Years of Life Lost due to premature death (YLLs) and Years Lived with Disability (YLDs). YLLs, an indicator that measured the burden of premature death, were calculated as the sum of the standard life expectancy of each death at the age of death. YLDs, an indicator that quantified the burden of non-fatal health outcomes, were estimated as the product of a disability weight for the health states of each sequela and prevalence and then adjusted for comorbidity. The detailed methods used to assess deaths and DALYs attributable to risk factors, including tobacco, high alcohol use, and drug use, have been described previously (1, 20, 24, 25).

### Statistical analysis

GBD study provides a standardized methodology for estimating the determinations and their 95% uncertainty interval (UI) of deaths and DALYs in terms of number and ASRs. Based on the GBD framework, 95% UIs for all estimates were calculated by averaging the data from 1,000 draws, with the lower and upper bounds of the 95% UIs established by the 2.5th and 97.5th ranked values among all 1,000 draws.

To evaluate temporal trends, the estimated annual percentage change (EAPC) of ASRs was calculated. The formula *Y = αX + β* was used, where *Y* represents the *Log10 (ASR)* value, and *X* denotes the calendar year. EAPC values were derived from the formula *EAPC = 100 × (10^α^-1)*. Positive EAPC values with 95% confidence intervals (CI) above zero indicated increasing trends, while negative values signified decreases.

The estimation of substance use was stratified by sex, age, geographic location, level of SDI, and diseases. Based on sex, we used histogram plots to explore the change in the number of deaths and DALYs caused by substance use with age. We also used a line chart to analyze the change in the ASRs of deaths and DALYs caused by substance use with age. The world map was used for the geographic location to visualize the disease burden attributable to substance use in 204 countries and territories. For SDI, Pearson’s correlation analysis was performed to estimate the strength and direction of the correlation between SDI and both EAPC and ASRs. For diseases, we analyzed the burden of GBD level 2 causes attributable to substance use.

Bayesian age-period-cohort (BAPC) model was used to predict the number and ASRs of deaths and DALYs from 2022 to 2040. The detail of BACP model is as follows (26):

The APC model assumes there is a multiplicative effect of age, period, and cohort.

$$Y_{\mathrm{ap}}=\mu^{\prime }\alpha_{a}^{\prime }\beta_{p}^{\prime }\gamma_{c}^{\prime }$$

where $Y_{\mathrm{ap}}$ denotes the incident case counts, $\alpha_{a}^{\prime }$ denotes the age effect, $\beta_{p}^{\prime }$ denotes the period effect, and $\gamma_{c}^{\prime }$ denotes the cohort effect. We use a=1,…,A to represent age groups, p=1,…,P to represent observation periods, and c=5×(A−a)+p to represent the birth cohorts. By taking the logarithm, the above model can be transformed into an additive model:

$$\log(Y_{\mathrm{ap}})=\mu +\alpha_{a}+\beta_{p}+\gamma_{c}$$

where μ , $\alpha_{a}$ , $\beta_{p}$ , and $\gamma_{c}$ are the logarithms of $\mu^{\prime }$ , $\alpha_{a}^{\prime }$ , $\beta_{p}^{\prime }$ , and $\gamma_{c}^{\prime }$ , respectively. In this study, we focus on the prediction of $Y_{\mathrm{ap}}$ ; therefore, the identifiability problem of APC models does not affect the estimation. We conducted a BAPC analysis with integrated nested Laplace approximation (INLA). To ensure smoothing, BAPC models assume independent mean-zero normal distributions on the second differences of all effects. Specifically, the BAPC model assumes prior distribution of the age effect as follows:

$$f(\alpha |k_{\alpha })\propto k_{\alpha }^{\frac{t-2}{2}}\exp\{-\frac{k_{\alpha }}{2}\sum_{i=3}^{J}{\left[(\alpha_{i}-\alpha_{i-1})-(\alpha_{i-1}-\alpha_{i-2})\right]}^{2}\}$$

Considering that we are interested in the incident case counts for age group a , with a t period into the future, the following equation can be applied:

$$\log(Y_{\mathrm{ap}+t})=\mu +\alpha_{a}+\beta_{p+t}+\gamma_{c+t}+\delta_{a,p+t}$$

In this study, we add an independent random effect $\delta_{a,p+t}∼N(0,k_{\delta }^{-1})$ to adjust for overdispersion. Considering the smoothing assumption, the BAPC models assume prior distribution of the period effect as follows:

$$\beta_{p+t}|\beta_{1},\ldots ,\beta_{p},k_{\beta }∼N((1+t)\beta_{p}-t\beta_{p-1},k_{\beta }^{-1}(1+2^{2}+\cdots +t^{2}))$$

All analyses and visualizations were conducted in R version 4.3.2^2^ and JD_GBDR (V2.37, Jingding Medical Technology Co., Ltd.). All statistical tests were two-sided, and *p*-values < 0.05 were considered statistically significant.

## Results

### The burden of substance use in the global

**Figure 1:**
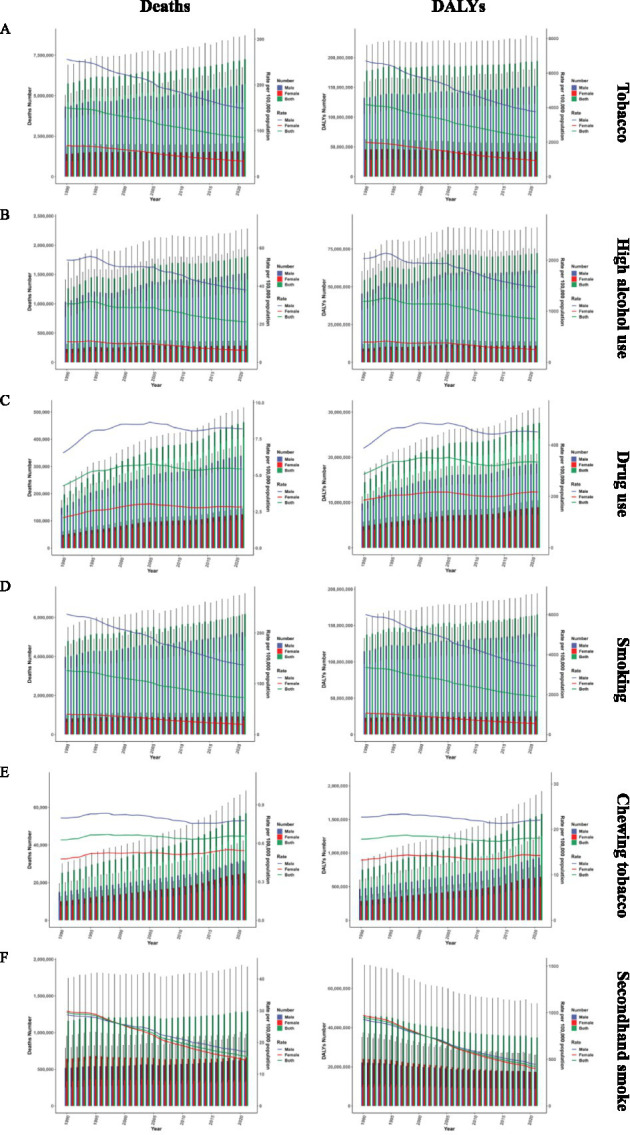
Global numbers and age-standardized rates of deaths and DALYs from 1990 to 2021. **(A)** Tobacco, **(B)** Smoking, **(C)** Chewing tobacco, **(D)** Secondhand smoke, **(E)** High alcohol use, **(F)** Drug use. Bar charts present numbers, broken line charts present age-standardized rates, and error bars present 95% uncertainty interval. DALYs, disability-adjusted life years.

Globally, the deaths and DALYs, and their change trends with substance use from 1990 and 2021 are presented in Table 1 and Figure 1. Between 1990 and 2021, the deaths and DALYs of all substance use increased, while the age-standardized death rates (ASDRs) and age-standardized DALY rates (ASDALYRs) per 100,000 population of all substance use declined. When examining substance use subtypes, tobacco was the leading component for the burden of all substance use. The ASDR and ASDALYR of tobacco and high alcohol use decreased consistently, while those for drug use increased slowly during the same period.

**Table 1:** Deaths and DALYs of substance use in 1990 and 2021 at global levels.

Types of substance use	Deaths (×1000, 95% UI)		ASDR per 100,000 (95% UI)		1990–2021 EAPC, (95% CI)	DALYs (×1000, 95% UI)		ASDALYR per 100,000 (95% UI)		1990–2021 EAPC, (95% CI)
	1990	2021	1990	2021		1990	2021	1990	2021	
All substance use										
Total	7208.17 (5737.52 to8836.76)	9523.09 (7572.1 to11499.82)	185.21 (147.99 to 226.30)	112.32 (89.10 to 135.80)	−1.75 (−1.83 to −1.68)	247461.49 (189588.87 to310106.81)	294573.40 (235220.45 to353490.81)	5661.11 (4400.70 to 7034.30)	3447.20 (2740.32 to 4149.49)	−1.74 (−1.82 to −1.67)
Female	1689.18 (1191.3 to2231.28)	1979.36 (1426.27 to2579.4)	80.78 (57.43 to 106.63)	43.07 (31.04 to 56.14)	−2.21 (−2.32 to −2.11)	59691.86 (39773.74 to80069.7)	62029.40 (44923.91 to80286.4)	2576.83 (1760.30 to 3420.15)	1408.77 (1015.00 to 1828.35)	−2.10 (−2.18 to −2.01)
Male	5518.99 (4526.92 to6623.13)	7543.72 (6084.4 to9033.69)	316.42 (260.07 to 379.10)	195.17 (156.41 to 234.67)	−1.68 (−1.75 to −1.61)	187769.63 (149128.2 to230802.74)	232544 0.00 (189329.72 to276809.92)	9121.58 (7379.14 to 11101.74)	5676.40 (4604.80 to 6765.32)	−1.67 (−1.74 to −1.59)
Tobacco										
Total	5746.56 (4619.49 to 6890.78)	7250.29 (5738.7 to 8703.27)	150.18 (121.46 to 179.42)	85.66 (67.58 to 102.93)	−1.95 (−1.99 to −1.90)	178364.24 (134781.81 to 221016.92)	194652.81 (152072.31 to 233417.37)	4178.73 (3227.26 to 5118.91)	2263.82 (1757.54 to 2725.76)	−2.09 (−2.13 to −2.05)
Female	1411.68 (984.93 to1858.86)	1568.61 (1094.26 to2064.25)	67.77 (47.91 to 88.91)	33.94 (23.62 to 44.76)	−2.42 (−2.48 to −2.35)	45808.71 (28716.91 to62384.75)	41962.99 (28334.88 to56107.18)	1993.06 (1295.40 to 2677.73)	937.89 (625.77 to 1259.72)	−2.55 (−2.60 to −2.50)
Male	4334.88 (3622.49 to5036.31)	5681.67 (4590.93 to6732.15)	256.25 (214.51 to 297.80)	149.02 (119.51 to 177.25)	−1.86 (−1.90 to −1.82)	132555.53 (105832.42 to159336.84)	152689.82 (123292.19 to180803.23)	6701.44 (5473.25 to 7969.16)	3753.24 (3015.84 to 4454.44)	−1.98 (−2.02 to −1.94)
Smoking										
Total	4784.43 (4087.94 to 5484.66)	6175.02 (5047.66 to 7226.38)	126.15 (107.10 to 145.41)	72.57 (59.31 to 85.08)	−1.91 (−1.96 to −1.86)	137534.82 (115962.11 to 160429.71)	165080.66 (135430.16 to 193938.45)	3360.67 (2833.61 to 3920.05)	1902.34 (1558.66 to 2234.96)	−1.95 (−1.99 to −1.91)
Female	811.87 (652.79 to988.66)	927.6 (739.48 to1160.37)	40.09 (32.15 to 49.05)	19.91 (15.88 to 24.95)	−2.44 (−2.51 to −2.36)	22887.5 (17853.71 to28349.21)	25047.5 (19360.38 to31532.7)	1072.43 (838.61 to 1329.16)	547.23 (423.04 to 688.77)	−2.28 (−2.33 to −2.23)
Male	3972.56 (3404 to4511.92)	5247.42 (4323.26 to6146.65)	237.06 (202.53 to 271.37)	137.08 (112.60 to 161.03)	−1.91 (−1.96 to −1.86)	114647.32 (97761.44 to132336.58)	140033.17 (115775.59 to163412.34)	6002.31 (5105.29 to 6925.75)	3424.67 (2829.09 to 4001.55)	−1.92 (−1.96 to −1.88)
Chewing tobacco										
Total	25.16 (19.53 to 30.08)	56.84 (45.75 to 68.79)	0.63 (0.49 to 0.75)	0.66 (0.53 to 0.79)	−0.01 (−0.07 to 0.04)	752.59 (586.36 to 906.82)	1581.85 (1264.08 to 1916.88)	17.86 (13.92 to 21.51)	18.11 (14.47 to 21.92)	−0.13 (−0.18 to −0.07)
Female	10.13 (7.72 to12.64)	24.96 (19.53 to30.96)	0.48 (0.36 to 0.60)	0.54 (0.43 to 0.67)	0.28 (0.22 to 0.34)	287.64 (218.7 to356.99)	646.64 (503.18 to814.62)	13.29 (10.10 to 16.52)	14.26 (11.11 to 17.94)	0.06 (−0.01 to 0.12)
Male	15.04 (10.65 to19.44)	31.88 (22.14 to42.23)	0.80 (0.57 to 1.04)	0.78 (0.54 to 1.03)	−0.28 (−0.34 to −0.22)	464.94 (328.02 to601.76)	935.21 (648.48 to1251.16)	22.73 (16.09 to 29.36)	22.10 (15.33 to 29.47)	−0.27 (−0.31 to −0.22)
Secondhand smoke										
Total	1162.72 (598.58 to 1741.48)	1292.10 (683.12 to 1896.16)	29.37 (15.87 to 43.55)	15.66 (8.25 to 23.01)	−2.21 (−2.26 to −2.15)	46260.09 (21049.94 to 71958.95)	34897.11 (17950.48 to 52207.79)	951.70 (452.48 to 1452.85)	423.17 (214.57 to 635.70)	−2.74 (−2.79 to −2.69)
Female	643.27 (342.74 to952.24)	664.27 (350.91 to981.28)	29.82 (16.44 to 43.82)	14.53 (7.66 to 21.47)	−2.53 (−2.59 to −2.47)	24005.55 (11164.73 to37325.73)	17426.4 (8908.8 to26078.54)	971.69 (468.41 to 1487.26)	401.65 (201.94 to 603.09)	−2.99 (−3.04 to −2.93)
Male	519.45 (255.84 to788.75)	627.83 (327.4 to920.71)	28.71 (14.80 to 43.48)	17.12 (8.83 to 25.24)	−1.80 (−1.85 to −1.74)	22254.54 (9875.59 to35012.56)	17470.7 (8941.31 to25919.71)	928.36 (432.83 to 1439.98)	447.88 (226.78 to 668.95)	−2.46 (−2.50 to −2.41)
High alcohol use										
Total	1264.2 (951.65 to 1713.5)	1809.44 (1424.95 to 2280.48)	30.74 (22.94 to 41.77)	21.21 (16.72 to 26.81)	−1.34 (−1.43 to −1.25)	54598.71 (42668.41 to 72289.11)	72254.24 (58930.61 to 89116.48)	1196.36 (932.50 to 1582.94)	850.23 (692.36 to 1050.02)	−1.28 (−1.37 to −1.18)
Female	228.49 (166.09 to311.66)	287.13 (227.3 to369.6)	10.92 (7.81 to 15.11)	6.30 (5.00 to 8.07)	−1.93 (−2.05 to −1.81)	9125.15 (7258.67 to12000.96)	11088.72 (9194.84 to13765.79)	399.23 (316.17 to 523.09)	254.20 (210.92 to 317.03)	−1.62 (−1.76 to −1.48)
Male	1035.72 (779.1 to1411.27)	1522.31 (1191.13 to1924.99)	53.63 (40.07 to 73.51)	37.95 (29.59 to 48.32)	−1.25 (−1.32 to −1.17)	45473.56 (34949.16 to60178.55)	61165.52 (49516.71 to75271.56)	2033.19 (1575.36 to 2685.58)	1472.60 (1191.45 to 1811.40)	−1.21 (−1.29 to −1.12)
Drug use										
Total	197.41 (166.38 to 232.48)	463.36 (408.45 to 516.08)	4.28 (3.58 to 5.11)	5.45 (4.80 to 6.06)	0.41 (0.25 to 0.57)	14498.54 (12138.64 to 16800.78)	27666.34 (24217.54 to 30956.95)	286.02 (240.94 to 332.45)	333.15 (290.42 to 373.71)	0.12 (−0.00 to 0.25)
Female	49.02 (40.28 to60.75)	123.62 (104.71 to145.55)	2.09 (1.71 to 2.61)	2.83 (2.42 to 3.31)	0.66 (0.46 to 0.85)	4758 (3798.16 to5683.99)	8977.69 (7394.18 to10413.43)	184.54 (148.73 to 219.33)	216.68 (178.31 to 251.60)	0.22 (0.12 to 0.32)
Male	148.39 (125.34 to175.54)	339.74 (302.34 to376.55)	6.54 (5.48 to 7.79)	8.20 (7.31 to 9.09)	0.35 (0.20 to 0.51)	9740.54 (8346.62 to11287.35)	18688.66 (16520.83 to20735.13)	386.95 (330.52 to 447.00)	450.55 (397.50 to 499.49)	0.09 (−0.05 to 0.24)

ASDR, age-standardized deaths rate; ASDALYR, age-standardized disability-adjusted life year rates; CI, confidence interval; DALYs, disability-adjusted life years; EAPC, estimated annual percentage change; UI, uncertainty interval.

Regarding specific tobacco subtypes, 85.17% of the tobacco-related deaths and 84.81% of the tobacco-related DALYs in 2021 were attributable to smoking, while the deaths and DALYs caused by chewing tobacco and secondhand smoke were relatively low. From 1990 to 2021, the trends of ASDR and ASDALY of smoking and secondhand smoke mirrored those of tobacco, while chewing tobacco was the subtype of tobacco with stable ADSR and ASDALYR.

### Tobacco burden in different regions and countries

**Figure 2:**
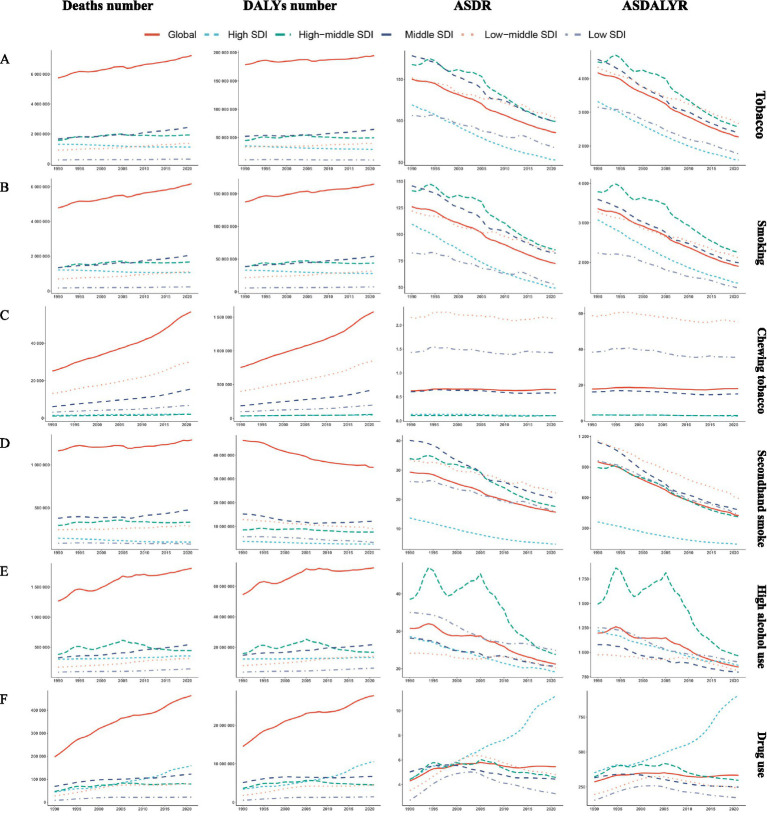
Global and regional trends of deaths and DALYs from 1990 to 2021. **(A)** Tobacco, **(B)** Smoking, **(C)** Chewing tobacco, **(D)** Secondhand smoke, **(E)** High alcohol use, **(F)** Drug use. ASDR, age-standardized death rate; ASDALYR, age-standardized disability-adjusted life year rate; DALYs, disability-adjusted life years.

**Figure 3:**
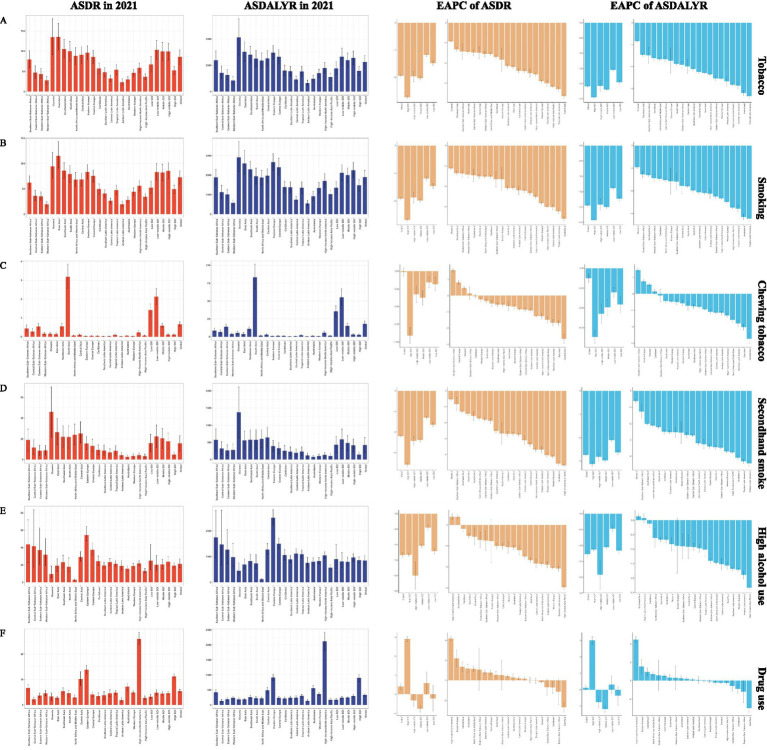
ASDR and ASDALYR in 2021 and the EAPC of ASDR and ASDALYR from 1990 to 2021 by SDI regions and 21 GBD regions. **(A)** Tobacco, **(B)** Smoking, **(C)** Chewing tobacco, **(D)** Secondhand smoke, **(E)** High alcohol use, **(F)** Drug use. ASDR, age-standardized death rate; ASDALYR, age-standardized disability-adjusted life year rate; EAPC, estimated annual percentage change.

**Figure 4:**
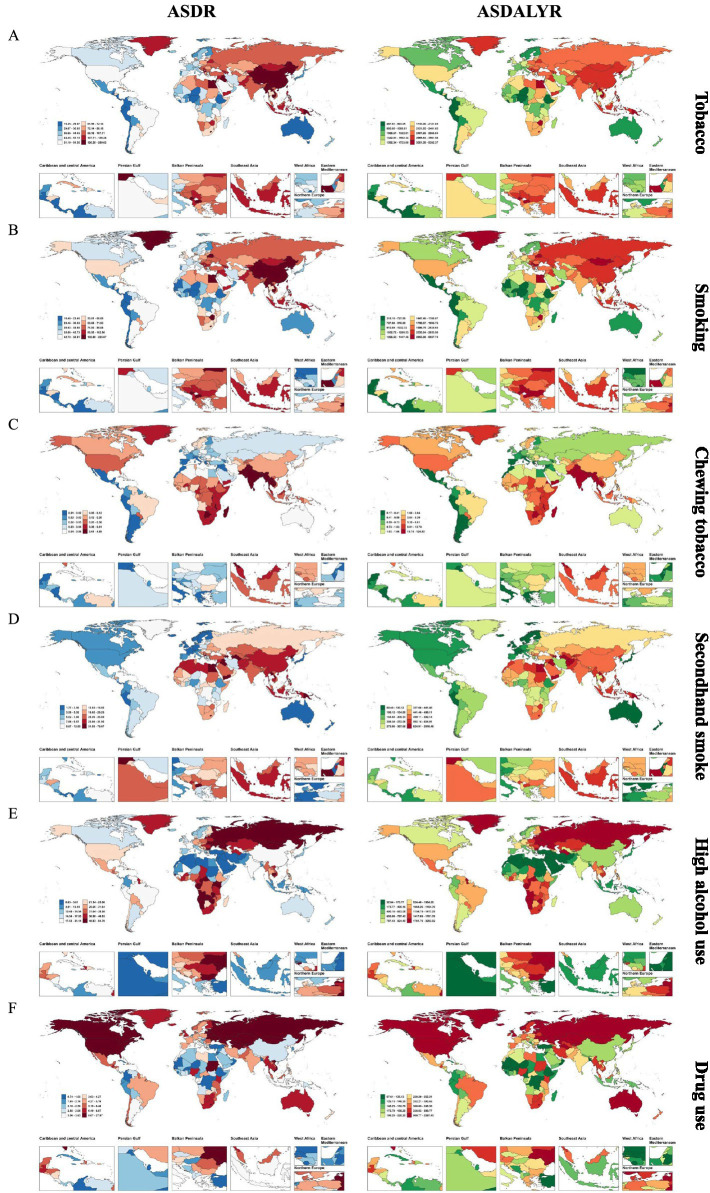
Global map of ASDR and ASDALYR by countries and territories in 2021. **(A)** Tobacco, **(B)** Smoking, **(C)** Chewing tobacco, **(D)** Secondhand smoke, **(E)** High alcohol use, **(F)** Drug use. ASDR, age-standardized death rate; ASDALYR, age-standardized disability-adjusted life year rate.

With regard to the SDI regions, low-middle SDI regions had the highest ASDR and ASDALYR in 2021. The ASDR and ASDALYR decreased in all SDI regions with the largest decline observed in high SDI regions (Supplementary Table S1; Figures 2A, 3A). Among the 21 global regions, the highest ASDR and ASDALYR in 2021 were observed in East Asia and Oceania (Supplementary Table S1; Figure 3A). All regions showed downward trends in ASDR and ASDALYR, with Australasia and Tropical Latin America experiencing the largest declines (Supplementary Table S1; Figure 3A). At the national level, in 2021, China had the highest deaths and DALYs, followed by India and the United States of America (Supplementary Table S2; Supplementary Figure S1A), collectively accounting for approximately 50% of global tobacco-related deaths and DALYs. The phenomenon that countries with larger populations have a greater absolute burden of disease was also observed for the other substance use subtypes. In 2021, the high ASDR and ASDALYR were mainly observed in low SDI countries (Supplementary Table S2; Figure 4A). In addition, high and high-middle SDI countries, such as Ireland, Singapore, Ethiopia, and Maldives, demonstrated the most significant downward trends in ASDR and ASDALYR (Supplementary Table S2; Supplementary Figure S2A).

### Smoking burden in different regions and countries

With regard to the SDI regions, high-middle SDI regions had the highest ASDR and ASDALYR in 2021. The ASDR and ASDALYR decreased in all SDI regions with the largest decline observed in high SDI regions (Supplementary Table S3; Figures 2B, 3B). Among the 21 global regions, the highest ASDR and ASDALYR were observed in East Asia, Oceania, and Eastern Europe (Supplementary Table S3; Figure 3B). All regions showed downward trends in ASDR and ASDALYR, with Australasia and Tropical Latin America experiencing the largest declines (Supplementary Table S3; Figure 3B). Nationally, in 2021, the highest ASDR and ASDALYR were mainly observed in low SDI countries (Supplementary Table S4; Figure 4B). Most countries showed downward trends in ASDR and ASDALYR, with high SDI countries demonstrating the most significant downward trends (Supplementary Table S4; Supplementary Figure S2B).

### Chewing tobacco burden in different regions and countries

With regard to the SDI regions, low and low-middle SDI regions had significantly higher ASDR and ASDALYR in 2021, while high and high-middle SDI regions had significantly lower ASDR and ASDALYR (Supplementary Table S5; Figures 2C, 3C). High SDI regions had the largest decrease in ASDR and ASDALYR (Supplementary Table S5; Figures 2C, 3C). Among the 21 global regions, South Asia had significantly higher ASDR and ASDALYR in 2021, which was 6–232 greater than other global regions (Supplementary Table S5; Figure 3C). Western Sub-Saharan Africa had the largest increase in ASDR and ASDALYR, while Central Asia had the largest decrease in ASDR and ASDALYR (Supplementary Table S5; Figure 3C). Nationally, in 2021, the highest ASDR and ASDALYR were mainly observed in South Asia countries (Supplementary Table S6; Figure 4C).

### Secondhand smoke burden in different regions and countries

With regard to the SDI regions, high SDI regions had lowest ASDR and ASDALYR in 2021, with largest decrease in ASDR and ASDALYR (Supplementary Table S7; Figures 2D, 3D). Among the 21 global regions, Oceania had the highest ASDR and ASDALYR, which was 2–18 times greater than other global regions (Supplementary Table S7; Figure 3D). All regions showed downward trends in ASDR and ASDALYR. High-income Asia Pacific and Australasia had the largest decline in ASDR, while Andean Latin America and Tropical Latin America had the largest decline in ASDALYR (Supplementary Table S7; Figure 3D). Nationally, in 2021, the highest ASDR and ASDALYR were mainly observed in South Asia, North Africa, and Middle East countries (Supplementary Table S8; Figure 4D).

### High alcohol use burden in different regions and countries

With regard to the SDI regions, high-middle SDI regions had the highest ASDR and ASDALYR in 2021. The ASDR and ASDALYR decreased in all SDI regions with the largest decline observed in high-middle SDI regions (Supplementary Table S9; Figures 2E, 3E). Among the 21 global regions, the highest ASDR and ASDALYR in 2021 were observed in Eastern Europe and Southern Sub-Saharan Africa (Supplementary Table S9; Figure 3E). Most regions showed downward trends in ASDR and ASDALYR, with high-income Asia Pacific experiencing the largest declines (Supplementary Table S9; Figure 3E). Nationally, in 2021, the highest ASDR and ASDALYR were mainly observed in cold region countries and African countries (Supplementary Table S10; Figure 1E).

### Drug use burden in different regions and countries

With regard to the SDI regions, high SDI regions had the highest ASDR and ASDALYR in 2021, with rates three to five times higher than those in other SDI regions (Supplementary Table S11; Figures 2F, 3F). High SDI regions also experienced the largest increase in ASDR and ASDALYR, while other SDI regions showed stable or downward trends (Supplementary Table S11; Figures 2F, 3F). Among the 21 global regions, the highest ASDR and ASDALYR in 2021 were observed in High-income North America, with rates 2–10 times higher than those in other global regions (Supplementary Table S11; Figure 3F). High-income North America also recorded the largest increase in ASDR and ASDALYR, while East Asia experienced the largest decrease in ASDR and ASDALYR (Supplementary Table S11; Figure 3F). Nationally, in 2021, the United States of America had the highest ASDR and ASDALYR (Supplementary Table S12; Figure 4F). In addition, the United States of America demonstrated the most significant upward trends in ASDALYR (Table S12, Figure S2F).

### Global burden of substance use by age and sex

**Figure 5:**
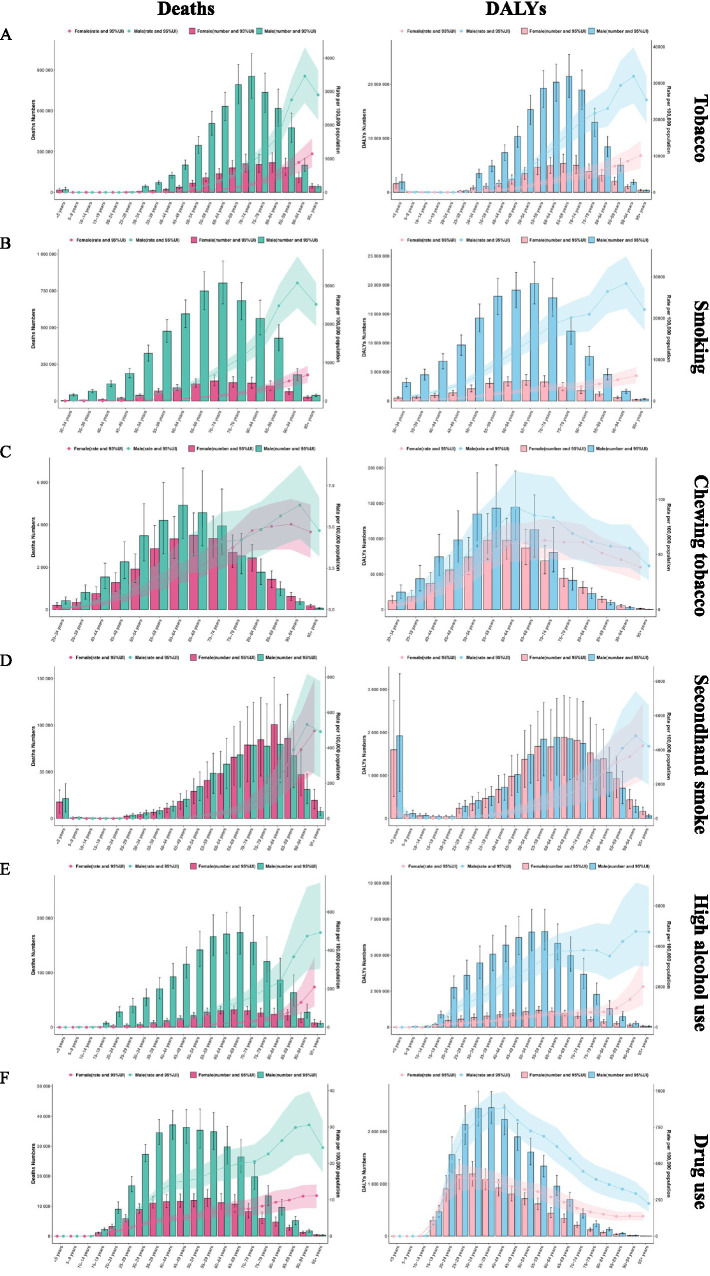
Age-specific counts and rates of deaths and DALYs by sex in 2021. **(A)** Tobacco, **(B)** Smoking, **(C)** Chewing tobacco, **(D)** Secondhand smoke, **(E)** High alcohol use, **(F)** Drug use. Bar charts present numbers, broken line charts present age-standardized rates, and error bars and shaded area present 95% uncertainty interval. DALYs, disability-adjusted life years.

For tobacco, smoking, and high alcohol use, the ASDR and ASDALYR increased with age (Figures 5A,B,E). For chewing tobacco and drug use, the ASDR also increased with age, while the ASDALYR showed a trend of initially increasing and then declining with age (Figures 5C,F). For secondhand smoke, the ASDR and ASDALYR displayed a bimodal distribution, with peaks in the < 5 years age group and ≥90 years age group (Figure 5D). In terms of sex, the ASDR and ASDALYR of all substance use subtypes are generally higher for male individuals than for female individuals, with the exception of secondhand smoke (Figure 5).

### Correlation between burden and SDI

**Figure 6:**
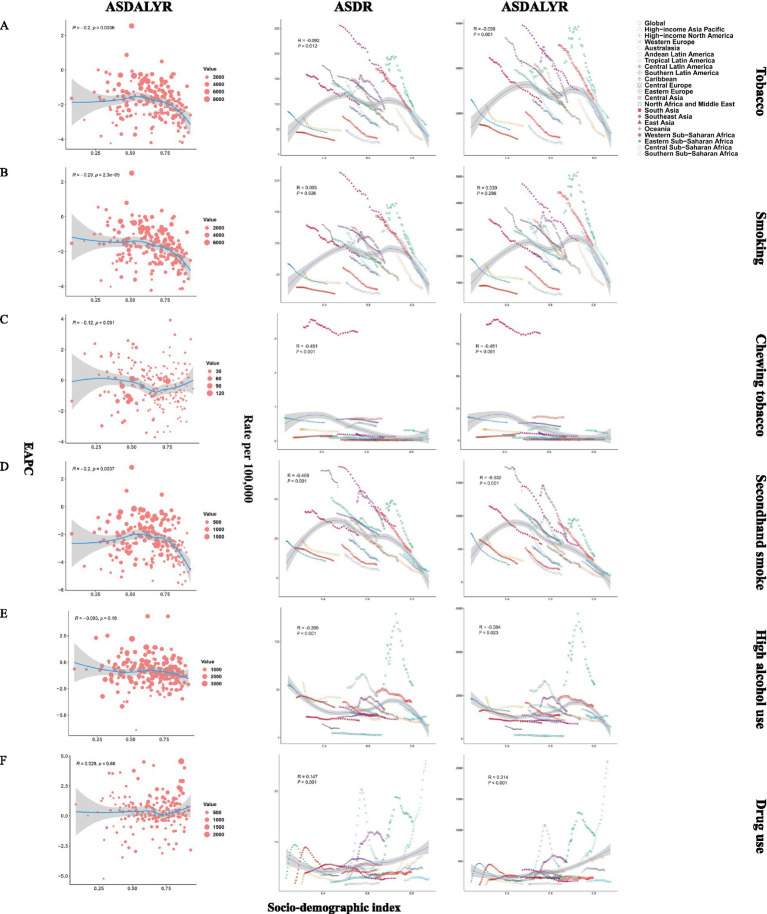
Relations between SDI and EAPC of 204 nations and territories from 1990 to 2021 and relations between SDI and both ASDR and ASDALYR of 21 GBD regions from 1990 to 2021. The *R* values indicate the strength of the correlation **(A)** Tobacco, **(B)** Smoking, **(C)** Chewing tobacco, **(D)** Secondhand smoke, **(E)** High alcohol use, **(F)** Drug use. ASDR, age-standardized death rate; ASDALYR, age-standardized disability-adjusted life year rate; EAPC, estimated annual percentage change.

To explore the role of the SDI in the burden of substance use, we generated scatter plots to illustrate the correlation between SDI and both the EAPC and ASDALYR over the past three decades (Figure 6). For high alcohol use, chewing tobacco, and secondhand smoke, negative correlations were observed between SDI and ASDALYR, while a positive correlation was observed between SDI and ASDALYR in drug use. For tobacco and smoking, the correlation between SDI and ASDALYR was more complex: While higher SDI was generally associated with higher ASDALYR, in regions where SDI exceeded 0.5, additional increases in SDI were associated with lower ASDALYR. For the correlation between SDI and EAPC, significant negative correlations were observed for tobacco, smoking, and secondhand smoke, while no significant correlations were observed for high alcohol use, drug use, and chewing tobacco.

### Burden due to substance use as risk factors for injuries and diseases

**Figure 7:**
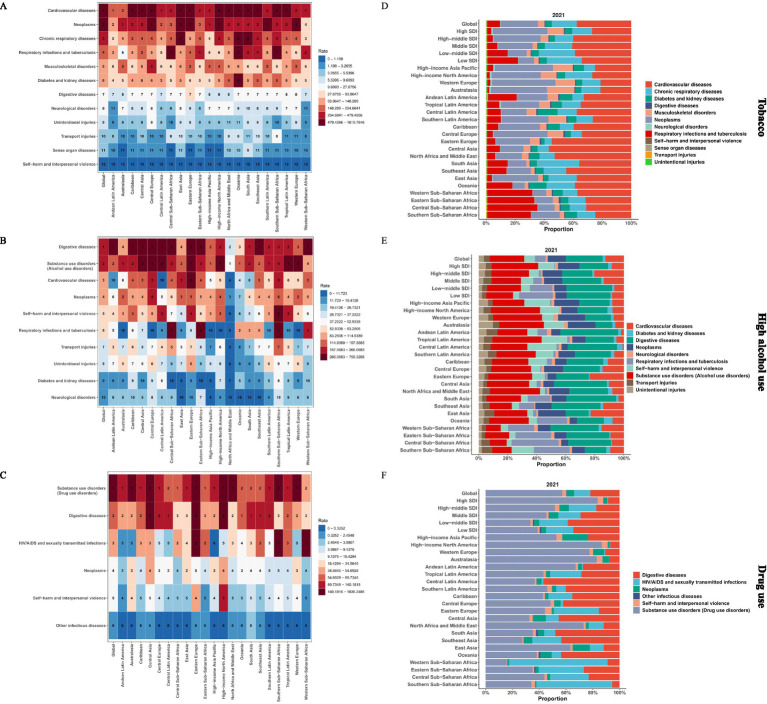
Ranking of different diseases or injuries ASDALYR attributed to **(A)** Tobacco, **(B)** High alcohol use, and **(C)** Drug use stratified by global and 21 GBD regions in 2021. Regional composition variation in different diseases or injuries ASDALYR attributed to **(D)** Tobacco, **(E)** High alcohol use, and **(F)** Drug use in 2021. ASDALYR, age-standardized disability-adjusted life year rate.

The distribution of diseases or injuries burden attributable to tobacco, high alcohol use, and drug use varied across GBD regions (Figure 7). Global ASDALYRs attributable to tobacco were highest for cardiovascular diseases, neoplasms, and chronic respiratory diseases. As SDI increased, the proportion of burden attributable to respiratory diseases decreased, while the proportion of burden attributable to neoplasms increased. Global ASDALYRs attributable to high alcohol use were highest for digestive diseases, alcohol use disorders, and cardiovascular diseases. High alcohol use burden was contributed to a wider variety of diseases and injuries, and all GBD regions showed a similar attributable disease pattern. Global ASDALYRs attributable to drug use were highest for drug use disorders, digestive diseases, and HIV/AIDS. HIV/AIDS accounted for a larger proportion of burden attributable to drug use in African regions. Drug use disorders were the largest contributor to the drug use-attributable burden in almost all regions, and their proportion increased as SDI increased.

### Predictions of burden

**Figure 8:**
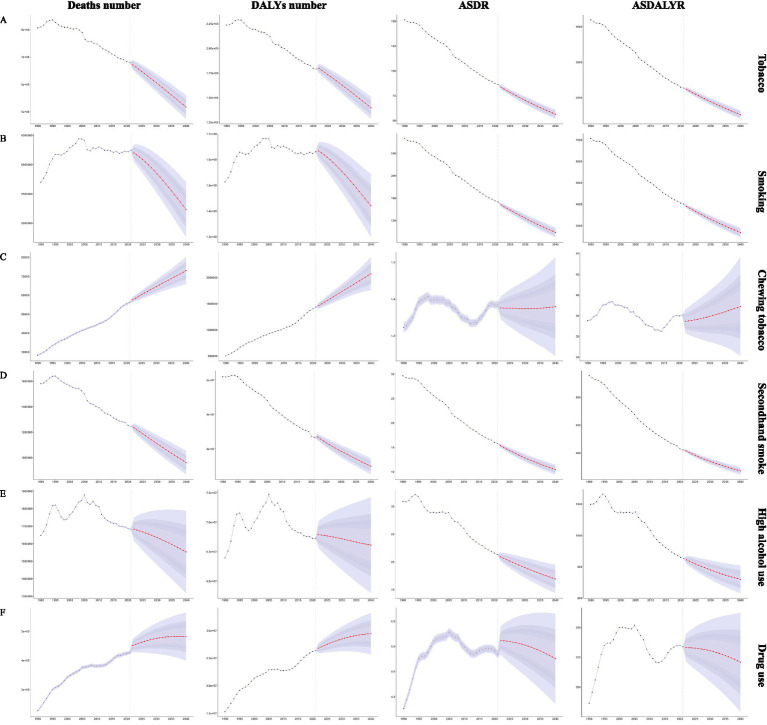
Global deaths, DALYs, and their age-standardized rates from 1990 to 2021 and their predictions to 2040. **(A)** Tobacco, **(B)** Smoking, **(C)** Chewing tobacco, **(D)** Secondhand smoke, **(E)** High alcohol use, **(F)** Drug use. The known data from 1990 to 2021 and predicted data from 2022 to 2040 were divided by a gray dashed line. The shaded area represents 95% uncertainty intervals. ASDR, age-standardized death rate; ASDALYR, age-standardized disability-adjusted life year rate; DALYs, disability-adjusted life years.

Based on the BAPC analysis, we projected that the deaths and DALYs of all substance use subtypes will decrease consistently from 2022 to 2040, with the exception of drug use and chewing tobacco. During the same period, the ASDR and ASDALYR of all substance use subtypes are projected to show declining trends, with the exception of chewing tobacco (Figure 8). The detailed annual estimates and their 95% UIs are presented in Supplementary Tables 13–18.

## Discussion

Substance use remains one of the world’s most severe public health issues, profoundly affecting individuals, families, and societies. This study presents a comprehensive analysis of the burden of substance use across regions and countries from 1990 to 2021. Over the past three decades, deaths and DALYs attributable to most substance use subtypes increased substantially, while the ASDR and ASDALYR decreased significantly during the same period. The burden varied considerably by substance use subtypes, geographic region, socioeconomic development level, sex, and age.

Tobacco use continues to be a leading risk factor for global disability and premature mortality, resulting in considerable economic costs (2). Although the ASRs for smoking and secondhand smoke declined significantly from 1990 to 2021, and those for chewing tobacco remained stable, the absolute number of deaths and DALYs of most tobacco subtypes remains high and continued to increase, indicating a persistent and substantial public health burden. Regional disparities were evident: The ASDALYR attributable to chewing tobacco was highest in South Asian countries, including Pakistan, Nepal, India, and Bangladesh, which collectively accounted for over 80% of global chewing tobacco-attributable DALYs. This aligns with existing reports indicating that more than 90% of smokeless tobacco users reside in Southeast Asia, where chewing tobacco is a predominant form (27). Previous findings from GBD 2017 suggested that high-SDI countries exhibit higher age-standardized tobacco exposure values (24). However, a clear negative correlation was identified between the SDI and the ASDALYR attributable to tobacco. Moreover, the EAPC of the ASDALYR for tobacco also showed a negative correlation with SDI. This observed disparity between higher tobacco exposure and lower burden of tobacco in high-SDI regions may be partly explained by stringent regulatory measures and advanced healthcare systems, which help mitigate tobacco-related health burden. Future projections suggest that while DALYs and ASDALYR related to smoking and secondhand smoke are expected to decline, those associated with chewing tobacco are likely to increase in both absolute number and ASRs. This projected trend may reflect the relative lag and insufficiency of targeted control policies of chewing tobacco than those for smoking, allowing its market and consumption to persist and even expand in certain regions (28).

In light of these findings, beyond conventional tobacco control measures such as the WHO Framework Convention on Tobacco Control (29), governments should adopt more tailored policy responses. First, countries bearing the highest burden from smoking and secondhand smoke, including China, India, the United States of America, and Indonesia, should intensify efforts to curb tobacco consumption through evidence-based measures such as taxation and price increases. Second, in South Asian countries with the highest burden of chewing tobacco, specific regulatory policies targeting these products are urgently needed. Third, research has shown that smoking prevalence is extremely high among people with serious mental illness, a subgroup that contributes substantially to tobacco burden (30–32). Such evidence highlights the importance of targeted interventions in vulnerable groups. Furthermore, as infants and young children endure the largest burden related to secondhand smoke, primarily due to household exposure (33), public health campaigns highlighting the harms of secondhand smoke to children should be promoted to reduce smoking among family members.

Since 1990, population growth and aging have contributed to a substantial increase in the number of people using alcohol (15). The burden attributable to high alcohol use also exhibited significant geographic variation. Eastern Europe recorded, mainly including high-SDI countries, the highest ASDALYR for high alcohol use, followed by Southern sub-Saharan Africa, mainly including low-SDI countries. Notably, considerable heterogeneity was observed even among high-SDI countries in 2021. Russia, Latvia, and Greenland had relatively high ASDALYR, whereas Kuwait, Saudi Arabia, and Singapore reported considerably lower ASDALYR. This pattern may be partly influenced by climatic factors, with colder regions generally demonstrating a higher high alcohol use-attributable burden (34). These findings suggest that policymakers should allocate more resources to control high alcohol use in colder regions. Across all GBD regions, a similar disease attribution pattern was observed, with alcohol use disorders, digestive diseases, and cardiovascular conditions accounting for the majority of high alcohol use-attributable DALYs.

Many high alcohol use-related harms are preventable or treatable. First, the global health community should advance efforts toward establishing a framework convention for alcohol control, analogous to the tobacco control framework (29). Second, taxation, along with regulation of availability and marketing, can significantly reduce alcohol-related harm. Third, reducing the alcohol content in beverages and implementing minimum unit pricing show considerable promise in decreasing alcohol-attributable harms (35, 36). Additionally, treatment and brief interventions have demonstrated efficacy and potential population-level impact (37).

Drug use is often characterized as a chronic relapsing condition with high rates of recurrence (2), leading to escalating social, health, and economic costs. In this study, drug use was the only substance use subtype that showed consistent increases in both the number of deaths and DALYs and their ASRs. High-income North America reported the highest ASDALYR for drug use, with rates 2–10 times higher than those in other regions. The United States of America recorded both the highest number and ASRs of DALYs related to drug use. A strong positive correlation was observed between SDI and ASDALYR attributable to drug use. Moreover, high-SDI regions experienced the most pronounced increases in ASDALYR from 1990 to 2021. Collectively, these findings indicate that high-SDI regions bear a disproportionately higher burden of drug use. This pattern may be driven by greater availability of both prescription and illicit opioids, alongside socioeconomic stressors despite overall wealth, leading to higher rates of misuse and fatal overdose (38).

HIV accounted for a substantial proportion of drug use-attributable burden in African regions, which is largely attributable to the high prevalence of HIV (39) and limited access to highly effective interventions for people who inject drugs (40). Scaling up needle and syringe programs and HIV antiretroviral therapy could alleviate much of this burden. Given that the burden of drug use is projected to continue rising, integrated prevention and treatment strategies should be widely implemented. Prevention should emphasize raising public awareness of the risks of drug use and addiction, while treatment efforts should focus on supporting sustained abstinence and preventing relapse.

In addition to geographic variation, substance use-related disease burden also differed significantly by sex and age. The health burden of nearly all substance use subtypes was higher in male individuals than in female individuals, with the exception of burden attributable to secondhand smoke. This phenomenon is consistent with previous GBD studies (4, 15, 17). The higher burden of substance use among male individual is mainly attributable to a greater prevalence of risk behaviors, such as heavier consumption and earlier initiation. This pattern is strongly influenced by socio-cultural norms of masculinity that promote such behaviors and discourage help-seeking (41). Therefore, government should implement men-specific prevention strategies, including higher alcohol/tobacco taxes, workplace brief interventions, and focused digital cessation apps. With regard to age, the burden of most substance use subtypes increased with age. The burden of drug use was highest among the 20–45 years age group for both sexes and then declined with advancing age. This may reflect the heightened life and social challenges, expectations, interpersonal alienation, and biological impulses in this demographic group, which may predispose individuals to drug experimentation as a form of self-medication (42). These findings underscore the need for focused attention on younger populations.

Despite previous reports on the limitations of GBD (1, 15, 20), it remains important to acknowledge the constraints of this analysis. First, the accuracy of our estimates depends on the quality and availability of underlying data, which vary across countries and regions. Data from low-SDI regions, in particular, may be less reliable due to limited health infrastructure and reporting systems. Second, GBD estimates rely on modeling techniques that may introduce uncertainty. Third, underreporting in substance use surveys, driven by stigma and legal fears, further deflates GBD estimates, especially where surveillance is weak. Fourth, in real word, the burden of different substance use subtypes is overlapping (people might use multiple substance), but GBD did not record the overlapping burden. Finally, although the new GBD 2023 database was released recently, it was not available at the time of our data analysis. Therefore, the time lag in the GBD 2021 database may affect the accuracy of predictions. Despite these limitations, our findings retain significant implications for public health policy and substance use control strategies.

In conclusion, substance use contributes substantially to the global disease burden, with its composition and magnitude varying widely across countries, sexes, and age groups and closely tracking socioeconomic development. There are several key insights. First, the burden of all tobacco subtypes showed a negative correlation with SDI, and chewing tobacco was the only substance use subtypes that showed increases in both absolute number and ASRs of deaths and DALYs. Second, at similar SDI levels, colder regions generally have higher burden attributable to high alcohol use. Third, high-SDI regions experienced higher and more rapidly growing burden attributable to drug use, and drug use primarily affects 20–45 years age group people. Fourth, the burden of nearly all substance use subtypes was higher in male individuals than that in female individuals and increased with age. Although current interventions have mitigated some of this burden, more targeted and population-specific approaches are needed, including region-specific actions, stronger monitoring systems, policy implementation, and evidence-based substance-use prevention.

## Data Availability

The original contributions presented in the study are included in the article/Supplementary material, further inquiries can be directed to the corresponding authors.

## Footnotes

1
https://vizhub.healthdata.org/gbd-results/

2
https://www.r-project.org
