# Supplementary material for: Burden of disease attributable to tobacco, high alcohol use, and drug use from 1990 to 2021
Source: Front Nutr. 2026 May 28;13:1701968. doi: 10.3389/fnut.2026.1701968 (PMC13253465; doi:10.3389/fnut.2026.1701968)
Supplement: Supplementary file 1 [file Table_1.docx]

**The detailed definitions of every substance use subtype**

Smoking: the prevalence of current smoking and the prevalence of former smoking were estimated using data from cross-sectional nationally representative household surveys. Current smokers were defined as individuals who currently use any smoked tobacco product on a daily or occasional basis. Former smokers were defined as individuals who quit using all smoked tobacco products for at least six months, where possible, or according to the definition used by the given survey.

Chewing tobacco: current chewing tobacco use is defined as current use (use within the last 30 days where possible, or according to the closest definition available from the survey) of any frequency (any, daily, or less than daily). Chewing tobacco includes local products, such as betel quid with tobacco.

Secondhand smoke: we define secondhand smoke exposure as current exposure to secondhand tobacco smoke at home or at work. We use household composition as a proxy for household secondhand smoke exposure and make the assumption that all persons living with a daily smoker are exposed to tobacco smoke. We use surveys to estimate the proportion of the population exposed to secondhand smoke at work. We only consider non-smokers to be exposed to secondhand smoke. Non-smokers are defined as all persons who are not daily smokers. Ex-smokers and occasional smokers are considered non-smokers in this analysis. Exposure is evaluated for both children and adults.

High alcohol use: it is defined as alcohol consumption in excess of the theoretical minimum risk exposure level (TMREL), the level of alcohol consumption at which all-cause risk is minimised. Prior to GBD 2021, this risk factor was simply “Alcohol use” and quantified the burden of alcohol consumption over the entire exposure range. We defined exposure as the grams per day of pure alcohol consumed among current drinkers.

Drug use: the drug use risk factor includes four dimensions of exposure. First, we include 100% attribution of drug use disorder estimates. Second, estimates of prevalence of opioid, amphetamine, and cocaine use disorder are used as exposures for risk of suicide. Third, instead of starting with an exposure model to estimate the proportion of HIV cases due to injection drug use (IDU), we model the population attributable fractions directly, alongside proportion of HIV cases due to sexual transmission and other routes of transmission, which mainly includes blood transfusions. Finally, prevalence of IDU is used to model risk of Hepatitis B and C viruses. Injecting drug users are at high risk of bloodborne infections due to the use of shared needles and injection equipment. Injecting drug use is defined as current IDU among individuals aged 15–64. The TMREL for drug use is defined as zero exposure to drug use.

Table S1 Deaths and DALYs of tobacco in 1990 and 2021 at regional levels

| Region | Deaths (×1000, 95% UI) | | ASDR per 100,000 (95% UI) | | 1990-2021 EAPC,  (95% CI) | DALYs (×1000, 95% UI) | | ASDALYR per 100,000 (95% UI) | | 1990-2021 EAPC,  (95% CI) |
| --- | --- | --- | --- | --- | --- | --- | --- | --- | --- | --- |
|  | 1990 | 2021 | 1990 | 2021 |  | 1990 | 2021 | 1990 | 2021 |  |
| High SDI | 1316.48 (1106.71 to 1538.39) | 1138.93 (922.22 to 1357.85) | 119.22 (100.15 to 139.43) | 52.80 (43.01 to 62.52) | -2.73 (-2.78 to -2.69) | 35540.55 (29248.69 to 42033.1) | 29745.77 (23485.29 to 36058.52) | 3320.06 (2725.70 to 3934.14) | 1582.19 (1239.97 to 1926.71) | -2.46 (-2.50 to -2.42) |
| High-middle SDI | 1576.32 (1312.73 to 1864.71) | 1942.41 (1547.93 to 2349.35) | 167.84 (138.59 to 199.44) | 98.89 (78.46 to 119.71) | -1.97 (-2.16 to -1.77) | 45153.51 (36842.85 to 53747.78) | 49698.2 (39746.98 to 59459.65) | 4499.96 (3648.55 to 5386.76) | 2563.01 (2036.93 to 3074.76) | -2.08 (-2.26 to -1.90) |
| Middle SDI | 1662.68 (1326.93 to 2011.46) | 2473.98 (1917.88 to 3023.11) | 178.57 (142.85 to 215.01) | 99.25 (75.91 to 121.67) | -2.04 (-2.10 to -1.97) | 52290.48 (39109.56 to 65662.94) | 64540.35 (49926.25 to 78431.07) | 4579.43 (3539.07 to 5604.85) | 2399.95 (1845.13 to 2924.90) | -2.20 (-2.24 to -2.15) |
| Low-middle SDI | 924.77 (681.36 to 1163.3) | 1373.02 (1067.54 to 1683.73) | 151.75 (115.36 to 188.75) | 103.11 (79.67 to 126.83) | -1.19 (-1.25 to -1.13) | 34070.92 (22949.69 to 45238.45) | 39889.59 (30069.2 to 49734.22) | 4346.95 (3179.23 to 5494.85) | 2659.61 (2016.63 to 3312.40) | -1.55 (-1.59 to -1.51) |
| Low SDI | 259.92 (177.12 to 352.55) | 315.99 (227.54 to 410.25) | 106.28 (78.30 to 137.48) | 67.31 (49.06 to 86.37) | -1.48 (-1.58 to -1.38) | 11111.14 (6406.04 to 15896.4) | 10604.58 (7250.31 to 14131.05) | 3152.69 (2190.19 to 4216.59) | 1765.73 (1270.25 to 2285.04) | -1.96 (-2.04 to -1.89) |
| Andean Latin America | 10.7 (7.31 to 14.28) | 13.5 (9.58 to 18.36) | 49.32 (35.02 to 64.09) | 23.22 (16.44 to 31.66) | -2.50 (-2.71 to -2.30) | 406.18 (245.67 to 577.28) | 400.28 (281.6 to 534.83) | 1479.35 (993.94 to 1990.37) | 657.58 (460.66 to 876.81) | -2.67 (-2.89 to -2.45) |
| Australasia | 21.52 (17.55 to 25.54) | 16.42 (12.91 to 20.29) | 91.11 (74.18 to 108.34) | 29.97 (23.78 to 36.77) | -3.66 (-3.77 to -3.54) | 603.66 (476.27 to 727.05) | 462.79 (351.02 to 587.45) | 2603.89 (2051.13 to 3141.61) | 975.35 (738.31 to 1238.53) | -3.20 (-3.31 to -3.09) |
| Caribbean | 24.03 (19.1 to 29.16) | 31.04 (23.63 to 38.37) | 95.02 (75.60 to 115.67) | 57.35 (43.64 to 70.96) | -1.70 (-1.79 to -1.62) | 711.19 (539.73 to 884.24) | 860.89 (661.72 to 1074.42) | 2607.38 (2014.83 to 3204.45) | 1610.13 (1233.00 to 2019.39) | -1.62 (-1.70 to -1.53) |
| Central Asia | 66.16 (51.65 to 81.09) | 70.43 (55.24 to 84.88) | 136.01 (108.75 to 165.26) | 90.37 (70.41 to 111.05) | -1.50 (-1.79 to -1.20) | 2374.16 (1710.59 to 3043.5) | 2222.08 (1706.32 to 2733.76) | 4274.16 (3247.19 to 5306.05) | 2519.94 (1930.10 to 3107.25) | -1.99 (-2.31 to -1.67) |
| Central Europe | 246.28 (207.32 to 284.72) | 187.54 (151.45 to 223.21) | 169.44 (141.16 to 197.55) | 85.54 (69.14 to 101.55) | -2.38 (-2.47 to -2.30) | 7416.49 (6157.68 to 8666.54) | 5243.1 (4227.99 to 6300.35) | 5036.53 (4141.01 to 5934.37) | 2644.37 (2126.88 to 3193.76) | -2.24 (-2.31 to -2.16) |
| Central Latin America | 60.47 (45.43 to 75.54) | 79.41 (58.94 to 100.01) | 75.29 (57.62 to 93.41) | 32.41 (24.02 to 40.91) | -3.01 (-3.16 to -2.86) | 2064.76 (1425.95 to 2680.74) | 2346.93 (1709.17 to 3025.95) | 2059.08 (1522.34 to 2592.55) | 920.31 (667.62 to 1188.33) | -2.86 (-3.03 to -2.70) |
| Central Sub-Saharan Africa | 19.88 (12.25 to 27.8) | 27.52 (18.61 to 37.58) | 73.81 (50.62 to 99.02) | 47.43 (32.47 to 64.33) | -1.48 (-1.63 to -1.33) | 908.31 (485.72 to 1353.86) | 1057.59 (693.18 to 1471.71) | 2365.03 (1526.70 to 3231.55) | 1434.56 (968.64 to 1952.35) | -1.66 (-1.78 to -1.54) |
| East Asia | 1790.54 (1412.37 to 2186.75) | 2734.82 (2077.34 to 3425.18) | 256.21 (202.58 to 313.19) | 135.15 (102.03 to 168.83) | -2.19 (-2.29 to -2.09) | 51335.73 (39521.14 to 63954.75) | 64573.81 (49501.88 to 81056.11) | 5913.25 (4602.47 to 7268.12) | 3025.23 (2320.28 to 3793.48) | -2.28 (-2.34 to -2.23) |
| Eastern Europe | 392.24 (335.05 to 452.99) | 331.55 (272.76 to 392.28) | 143.94 (121.53 to 167.34) | 95.86 (78.91 to 113.12) | -1.84 (-2.42 to -1.27) | 11685.14 (9889.71 to 13526.02) | 9661.17 (7932.27 to 11388.35) | 4243.94 (3559.50 to 4940.49) | 2956.51 (2423.33 to 3489.58) | -1.72 (-2.29 to -1.15) |
| Eastern Sub-Saharan Africa | 73.63 (48.3 to 101.45) | 75.26 (54.27 to 98.92) | 85.26 (61.49 to 112.92) | 43.68 (32.20 to 57.06) | -2.40 (-2.50 to -2.30) | 3310.69 (1823.76 to 4852.98) | 2814.9 (1909.84 to 3826.2) | 2609.82 (1780.74 to 3553.32) | 1271.32 (915.19 to 1677.43) | -2.54 (-2.63 to -2.45) |
| High-income Asia Pacific | 180.79 (148.41 to 213.92) | 189.24 (151.4 to 228.86) | 94.14 (77.01 to 112.20) | 36.86 (29.70 to 44.06) | -3.18 (-3.23 to -3.13) | 5113.52 (4063.44 to 6168.85) | 4374.73 (3408.41 to 5395.92) | 2534.79 (2006.89 to 3072.61) | 1108.03 (854.95 to 1372.95) | -2.82 (-2.87 to -2.76) |
| High-income North America | 433.81 (363.7 to 506.91) | 394.63 (319.3 to 475.36) | 123.68 (104.04 to 144.05) | 58.99 (47.99 to 70.75) | -2.56 (-2.64 to -2.49) | 11963.43 (9864.78 to 14080.41) | 10899.07 (8592.8 to 13282.93) | 3593.62 (2964.97 to 4229.36) | 1800.15 (1402.79 to 2201.13) | -2.35 (-2.40 to -2.30) |
| North Africa and Middle East | 232.79 (180.61 to 283.64) | 372.04 (283.59 to 458.57) | 141.16 (111.92 to 171.61) | 88.21 (67.12 to 108.73) | -1.56 (-1.63 to -1.49) | 8221.1 (5870.22 to 10353.67) | 11783.15 (8896.56 to 14566.91) | 3926.70 (3005.36 to 4791.04) | 2373.52 (1783.91 to 2939.96) | -1.66 (-1.72 to -1.60) |
| Oceania | 5.52 (3.59 to 7.5) | 10.41 (7.08 to 13.79) | 176.27 (120.25 to 236.85) | 134.53 (91.25 to 179.92) | -0.93 (-0.97 to -0.88) | 230 (140.45 to 319.93) | 410.45 (268.45 to 552.74) | 5349.48 (3515.60 to 7253.89) | 4124.21 (2774.41 to 5534.71) | -0.84 (-0.88 to -0.80) |
| South Asia | 885.74 (651.08 to 1125.06) | 1337.43 (1019.84 to 1649.02) | 158.24 (119.15 to 199.97) | 99.63 (75.52 to 123.81) | -1.46 (-1.55 to -1.37) | 32463.75 (22118.5 to 43169.16) | 37692.23 (28027.93 to 47058.19) | 4463.03 (3261.00 to 5700.32) | 2502.46 (1859.85 to 3119.73) | -1.87 (-1.93 to -1.81) |
| Southeast Asia | 382 (295.14 to 470.42) | 643.64 (509.17 to 783.44) | 155.39 (122.17 to 189.73) | 104.88 (82.63 to 128.83) | -1.33 (-1.43 to -1.24) | 13240.45 (9508.71 to 16974.15) | 19176.88 (15056.08 to 23398.84) | 4336.83 (3257.60 to 5391.85) | 2797.47 (2176.89 to 3432.42) | -1.46 (-1.52 to -1.40) |
| Southern Latin America | 46.07 (37.14 to 55.36) | 41.69 (32.14 to 51.62) | 100.66 (80.88 to 121.47) | 47.94 (37.08 to 59.14) | -2.14 (-2.24 to -2.04) | 1455.06 (1157.96 to 1746.95) | 1287.68 (970.98 to 1592.14) | 3109.54 (2469.91 to 3733.87) | 1552.04 (1168.95 to 1921.57) | -2.10 (-2.17 to -2.03) |
| Southern Sub-Saharan Africa | 35.29 (25.73 to 44.82) | 45.96 (34.11 to 58.12) | 124.22 (92.19 to 157.01) | 79.35 (58.34 to 100.74) | -1.43 (-1.82 to -1.04) | 1356.44 (927.11 to 1786.01) | 1571.07 (1144.04 to 2023.43) | 3905.06 (2834.26 to 4957.55) | 2398.65 (1749.81 to 3079.57) | -1.49 (-1.85 to -1.11) |
| Tropical Latin America | 126.38 (100.81 to 150.79) | 138.14 (106.35 to 171.48) | 144.08 (114.72 to 172.81) | 54.16 (41.57 to 67.43) | -3.30 (-3.41 to -3.19) | 4175.29 (3191.58 to 5109.24) | 4021.18 (3052.41 to 5022.65) | 4078.81 (3179.26 to 4942.60) | 1539.42 (1164.96 to 1927.90) | -3.35 (-3.46 to -3.25) |
| Western Europe | 663.24 (556.93 to 772.51) | 450.57 (365.09 to 539.48) | 114.40 (96.28 to 132.93) | 46.68 (38.26 to 55.24) | -2.91 (-2.97 to -2.84) | 16845.77 (13850.95 to 19952.17) | 11345.52 (8921.88 to 13821.96) | 3112.74 (2552.25 to 3688.52) | 1408.74 (1099.66 to 1718.13) | -2.55 (-2.59 to -2.50) |
| Western Sub-Saharan Africa | 49.47 (28.64 to 72.01) | 59.06 (37.36 to 81.84) | 44.54 (29.63 to 61.03) | 27.93 (18.72 to 37.76) | -1.56 (-1.70 to -1.42) | 2483.1 (1207.95 to 3864.28) | 2447.32 (1435.25 to 3548.1) | 1503.72 (917.38 to 2144.39) | 847.81 (552.64 to 1158.71) | -1.91 (-2.07 to -1.76) |

ASDR, age-standardized Deaths rates; ASDALYR, age-standardized disability-adjusted life years rates; CI, confidence interval; DALYs, disability-adjusted life years rates; EAPC, estimated annual percentage change; UI, uncertainty interval.

Table S2 Deaths and DALYs of tobacco in 1990 and 2021 at national levels

| Location | Deaths (95% UI) | | ASDR per 100,000 (95% UI) | | 1990-2021 EAPC,  (95% CI) | DALYs (95% UI) | | ASDALYR per 100,000 (95% UI) | | 1990-2021 EAPC,  (95% CI) |
| --- | --- | --- | --- | --- | --- | --- | --- | --- | --- | --- |
|  | 1990 | 2021 | 1990 | 2021 |  | 1990 | 2021 | 1990 | 2021 |  |
| Afghanistan | 7639.50 (5047.56 to 10781.75) | 9150.98 (5970.11 to 12744.25) | 108.15 (73.35 to 149.69) | 87.54 (58.81 to 119.58) | -0.66 (-0.97 to -0.34) | 272154.06 (166595.67 to 401855.16) | 359023.57 (227506.67 to 504628.30) | 3176.23 (2087.77 to 4526.59) | 2524.33 (1687.36 to 3481.17) | -0.71(-1.03,-0.40) |
| Albania | 3089.70 (2422.96 to 3733.89) | 4729.19 (3682.01 to 5821.27) | 165.35 (131.45 to 198.23) | 111.77 (87.01 to 137.23) | -1.03 (-1.29 to -0.76) | 91415.03 (67448.14 to 115580.63) | 110408.82 (85383.14 to 136358.23) | 4083.75 (3108.35 to 5027.64) | 2665.04 (2050.19 to 3296.07) | -1.16(-1.40,-0.91) |
| Algeria | 13085.82 (10031.25 to 16441.38) | 23083.46 (16505.30 to 30903.14) | 143.22 (108.04 to 180.71) | 84.62 (60.28 to 112.57) | -1.64 (-1.78 to -1.49) | 410895.62 (301635.42 to 524243.70) | 667934.90 (479761.96 to 877497.39) | 3216.14 (2417.07 to 4070.84) | 1908.44 (1369.75 to 2498.74) | -1.77(-1.90,-1.65) |
| American Samoa | 31.52 (24.14 to 39.48) | 54.23 (39.72 to 71.57) | 144.46 (108.77 to 181.15) | 116.07 (84.23 to 153.94) | -0.82 (-0.89 to -0.74) | 1137.24 (847.89 to 1444.74) | 1853.20 (1367.47 to 2398.51) | 4126.06 (3139.83 to 5175.86) | 3606.26 (2630.91 to 4681.09) | -0.52(-0.59,-0.46) |
| Andorra | 54.10 (39.24 to 73.87) | 70.14 (46.39 to 96.03) | 97.11 (70.79 to 130.86) | 44.13 (29.33 to 60.60) | -2.36 (-2.63 to -2.09) | 1566.28 (1143.53 to 2073.32) | 2014.01 (1364.14 to 2709.95) | 2662.28 (1947.93 to 3523.10) | 1350.10 (920.55 to 1823.21) | -2.07(-2.26,-1.87) |
| Angola | 4730.39 (3130.75 to 6497.17) | 7987.56 (5470.63 to 10877.01) | 103.96 (73.10 to 138.21) | 65.12 (45.14 to 86.41) | -1.57 (-1.75 to -1.39) | 202593.78 (120667.54 to 290078.90) | 300263.74 (199652.38 to 421509.83) | 3264.33 (2236.83 to 4388.70) | 1936.40 (1330.49 to 2605.69) | -1.73(-1.91,-1.56) |
| Antigua and Barbuda | 25.47 (19.37 to 32.26) | 31.30 (22.98 to 39.85) | 47.42 (36.17 to 59.78) | 30.36 (22.13 to 38.91) | -1.69 (-1.92 to -1.45) | 669.92 (499.00 to 850.41) | 934.19 (662.47 to 1213.34) | 1316.93 (981.43 to 1671.66) | 851.57 (600.17 to 1108.83) | -1.61(-1.81,-1.40) |
| Argentina | 33813.69 (27696.12 to 40678.84) | 30744.78 (23719.65 to 38163.10) | 105.58 (85.84 to 127.42) | 55.23 (42.78 to 68.28) | -1.78 (-1.90 to -1.65) | 1066160.99 (856399.58 to 1280735.29) | 928898.33 (706916.31 to 1147730.87) | 3287.04 (2640.84 to 3953.90) | 1737.73 (1322.00 to 2148.10) | -1.88(-1.97,-1.78) |
| Armenia | 4616.17 (3820.97 to 5422.96) | 5083.36 (4157.77 to 6135.72) | 174.29 (143.68 to 205.00) | 117.53 (95.83 to 141.93) | -1.55 (-1.71 to -1.39) | 144287.17 (115455.45 to 172850.72) | 130259.73 (106926.02 to 156150.28) | 4890.91 (3942.91 to 5826.11) | 3106.35 (2532.21 to 3739.54) | -1.71(-1.84,-1.57) |
| Australia | 17248.66 (14087.87 to 20484.75) | 12838.22 (10003.29 to 16010.51) | 87.55 (71.47 to 103.99) | 27.93 (21.95 to 34.52) | -3.74 (-3.85 to -3.64) | 486123.41 (380914.06 to 583102.22) | 367787.26 (276218.27 to 470210.15) | 2511.12 (1964.55 to 3011.93) | 929.28 (692.24 to 1191.12) | -3.21(-3.31,-3.12) |
| Austria | 9701.19 (8053.45 to 11503.95) | 8495.14 (6752.32 to 10339.82) | 83.38 (69.33 to 98.38) | 46.42 (37.34 to 55.66) | -1.88 (-1.98 to -1.77) | 261830.84 (214260.62 to 313831.36) | 230522.89 (180085.61 to 280102.66) | 2446.04 (1994.92 to 2923.40) | 1449.25 (1132.74 to 1770.00) | -1.68(-1.76,-1.59) |
| Azerbaijan | 8463.70 (6413.45 to 10572.83) | 11260.43 (8760.24 to 13617.27) | 162.31 (126.89 to 201.01) | 122.78 (95.15 to 150.03) | -0.73 (-0.99 to -0.47) | 325199.21 (218614.12 to 429245.56) | 338012.98 (260991.12 to 415309.34) | 5306.15 (3803.76 to 6752.83) | 3227.68 (2453.07 to 3990.32) | -1.64(-1.87,-1.42) |
| Bahamas | 80.80 (61.31 to 103.90) | 137.35 (92.76 to 183.51) | 51.74 (39.11 to 66.91) | 34.16 (22.99 to 45.47) | -1.25 (-1.34 to -1.16) | 2643.57 (1968.57 to 3357.39) | 4447.50 (3028.59 to 5871.82) | 1543.13 (1153.55 to 1968.35) | 1022.49 (694.12 to 1346.98) | -1.22(-1.32,-1.13) |
| Bahrain | 279.98 (220.52 to 347.46) | 581.01 (441.96 to 758.22) | 196.50 (152.58 to 245.63) | 90.06 (67.04 to 118.01) | -3.06 (-3.38 to -2.73) | 9109.78 (7080.89 to 11540.58) | 21618.24 (15974.32 to 28395.13) | 4555.26 (3526.91 to 5706.58) | 2122.33 (1583.50 to 2778.90) | -3.02(-3.26,-2.78) |
| Bangladesh | 96093.17 (70060.59 to 124891.94) | 130134.62 (93705.84 to 166745.72) | 188.53 (145.03 to 237.46) | 102.49 (73.56 to 130.39) | -2.05 (-2.26 to -1.84) | 3753402.35 (2396757.86 to 5148509.07) | 3742363.21 (2695382.40 to 4839905.08) | 5494.94 (4067.20 to 7069.84) | 2663.54 (1920.42 to 3439.82) | -2.33(-2.44,-2.22) |
| Barbados | 120.81 (90.34 to 154.93) | 118.74 (80.01 to 161.48) | 40.74 (30.86 to 52.09) | 23.01 (15.52 to 31.27) | -2.25 (-2.50 to -2.00) | 2950.92 (2224.04 to 3790.62) | 3005.24 (1984.92 to 4126.43) | 1086.77 (818.39 to 1394.86) | 618.54 (405.44 to 855.61) | -2.15(-2.36,-1.95) |
| Belarus | 19324.29 (16106.59 to 22643.39) | 19795.62 (15464.28 to 24492.69) | 151.46 (125.77 to 178.25) | 123.79 (96.92 to 152.72) | -1.18 (-1.52 to -0.84) | 555222.15 (462417.00 to 648246.39) | 543601.62 (424932.80 to 668104.58) | 4371.88 (3618.10 to 5128.12) | 3561.58 (2792.04 to 4392.49) | -1.26(-1.63,-0.89) |
| Belgium | 20323.32 (16866.10 to 23584.10) | 12474.37 (9931.75 to 15105.53) | 131.61 (109.49 to 152.46) | 51.95 (42.34 to 62.11) | -2.96 (-3.06 to -2.86) | 505754.93 (418040.02 to 590504.45) | 315327.35 (250303.88 to 385468.82) | 3495.72 (2890.49 to 4088.34) | 1544.72 (1220.37 to 1882.54) | -2.60(-2.68,-2.51) |
| Belize | 48.22 (35.96 to 60.84) | 105.94 (78.39 to 138.56) | 50.12 (38.20 to 62.40) | 36.68 (26.95 to 47.99) | -1.25 (-1.66 to -0.83) | 1553.27 (1091.80 to 2027.72) | 3417.53 (2493.33 to 4534.06) | 1456.03 (1088.36 to 1820.25) | 1052.39 (766.82 to 1395.41) | -1.23(-1.56,-0.90) |
| Benin | 1339.97 (775.00 to 1958.62) | 1282.19 (789.88 to 1825.93) | 52.69 (35.14 to 72.29) | 23.37 (14.98 to 32.40) | -2.80 (-2.94 to -2.66) | 63385.89 (30846.18 to 98947.37) | 50937.73 (28852.96 to 74890.82) | 1707.46 (1046.37 to 2431.98) | 707.33 (442.36 to 991.73) | -3.04(-3.18,-2.89) |
| Bermuda | 50.81 (39.68 to 62.77) | 50.64 (37.57 to 67.23) | 83.07 (64.51 to 103.12) | 36.23 (26.96 to 47.96) | -2.54 (-2.75 to -2.34) | 1369.65 (1069.63 to 1693.88) | 1275.82 (937.58 to 1678.34) | 2152.58 (1681.13 to 2670.19) | 1024.19 (753.52 to 1346.48) | -2.29(-2.50,-2.07) |
| Bhutan | 208.00 (130.45 to 307.55) | 329.21 (226.45 to 467.60) | 87.03 (56.44 to 126.63) | 58.52 (40.12 to 83.39) | -1.35 (-1.48 to -1.22) | 8392.88 (4741.90 to 12714.73) | 8519.29 (5766.33 to 12045.50) | 2419.86 (1549.03 to 3540.87) | 1387.91 (943.92 to 1967.39) | -1.92(-2.06,-1.78) |
| Bolivia (Plurinational State of) | 2796.10 (1817.71 to 3872.27) | 3089.64 (2133.64 to 4439.67) | 78.52 (54.56 to 105.19) | 36.10 (25.03 to 51.60) | -2.10 (-2.42 to -1.79) | 117513.11 (64714.34 to 174869.13) | 94601.14 (63495.47 to 136027.40) | 2438.51 (1598.64 to 3355.35) | 985.06 (667.03 to 1411.77) | -2.57(-2.91,-2.24) |
| Bosnia and Herzegovina | 5515.48 (4635.21 to 6438.61) | 6698.24 (5054.12 to 8490.32) | 140.57 (115.85 to 166.86) | 106.37 (79.63 to 134.78) | -0.98 (-1.13 to -0.84) | 173523.91 (143849.65 to 204393.90) | 179251.39 (136880.82 to 225505.17) | 3960.92 (3274.96 to 4686.15) | 3054.81 (2313.94 to 3838.41) | -0.88(-1.02,-0.74) |
| Botswana | 954.39 (658.46 to 1340.07) | 1187.61 (838.33 to 1590.46) | 172.58 (119.97 to 237.45) | 84.35 (59.33 to 113.99) | -2.49 (-2.77 to -2.21) | 32934.70 (22091.57 to 47448.37) | 40013.21 (27746.12 to 53747.04) | 4826.68 (3353.60 to 6730.05) | 2361.18 (1653.83 to 3145.27) | -2.56(-2.87,-2.24) |
| Brazil | 124299.79 (98943.39 to 148409.08) | 134181.84 (102857.54 to 166890.55) | 145.26 (115.52 to 174.35) | 53.80 (41.08 to 67.04) | -3.35 (-3.46 to -3.24) | 4113842.63 (3142599.57 to 5031241.43) | 3912707.15 (2960923.34 to 4897015.71) | 4118.31 (3208.10 to 4994.24) | 1533.28 (1156.69 to 1921.18) | -3.40(-3.51,-3.29) |
| Brunei Darussalam | 166.42 (130.61 to 203.07) | 206.40 (155.50 to 262.23) | 174.04 (135.66 to 214.32) | 65.62 (48.86 to 82.89) | -2.73 (-3.02 to -2.44) | 5231.25 (4019.02 to 6454.76) | 7339.44 (5510.50 to 9484.77) | 4420.12 (3439.43 to 5452.76) | 1836.93 (1357.87 to 2361.23) | -2.64(-2.93,-2.35) |
| Bulgaria | 21377.58 (17515.74 to 25043.80) | 14114.46 (10995.84 to 17445.53) | 192.14 (156.09 to 228.51) | 107.71 (84.26 to 133.17) | -2.20 (-2.40 to -2.00) | 652261.43 (534382.92 to 764044.44) | 416661.41 (321713.06 to 511487.67) | 5642.01 (4586.49 to 6674.71) | 3544.96 (2760.11 to 4340.61) | -1.84(-2.01,-1.66) |
| Burkina Faso | 3045.44 (1500.58 to 4714.70) | 3420.92 (1927.07 to 5120.67) | 48.13 (28.41 to 71.05) | 30.84 (18.40 to 45.09) | -1.35 (-1.48 to -1.22) | 175586.75 (72548.81 to 288980.83) | 160273.39 (82708.50 to 251765.79) | 1786.55 (962.14 to 2685.82) | 1010.33 (593.83 to 1485.41) | -1.72(-1.87,-1.58) |
| Burundi | 2959.50 (1998.17 to 4063.75) | 2006.97 (1380.51 to 2722.47) | 111.37 (79.98 to 148.68) | 37.99 (26.27 to 50.40) | -3.76 (-4.23 to -3.30) | 121339.45 (74385.23 to 176461.36) | 77762.10 (52471.88 to 108889.26) | 3519.79 (2454.22 to 4790.61) | 1168.72 (813.71 to 1581.39) | -3.81(-4.25,-3.38) |
| Côte d'Ivoire | 89.73 (62.17 to 122.24) | 137.23 (93.48 to 186.37) | 38.69 (27.29 to 52.35) | 30.77 (20.81 to 41.80) | -2.05 (-2.49 to -1.61) | 2873.31 (1927.44 to 3975.67) | 4235.12 (2836.84 to 5815.78) | 1220.02 (844.42 to 1655.21) | 878.59 (590.97 to 1200.54) | -2.12(-2.55,-1.69) |
| Cabo Verde | 11455.71 (8064.52 to 15189.30) | 17306.25 (12459.79 to 22151.90) | 228.35 (173.55 to 285.48) | 159.54 (115.71 to 203.02) | -1.23 (-1.67 to -0.78) | 503977.41 (299554.03 to 713242.31) | 513947.65 (360236.52 to 667300.84) | 6760.19 (4878.43 to 8752.05) | 3969.25 (2815.31 to 5088.25) | -1.45(-1.76,-1.15) |
| Cambodia | 2935.75 (1738.11 to 4287.34) | 4532.17 (2695.60 to 6400.00) | 53.85 (35.36 to 74.89) | 33.45 (20.40 to 47.40) | -1.36 (-1.52 to -1.21) | 140127.36 (71593.71 to 215804.76) | 182556.33 (105949.31 to 260496.66) | 1782.39 (1125.15 to 2531.30) | 1031.10 (624.23 to 1450.14) | -2.02(-2.21,-1.84) |
| Cameroon | 39826.44 (33640.88 to 46632.35) | 34171.14 (27332.53 to 41348.65) | 122.78 (103.65 to 143.56) | 45.30 (36.66 to 54.63) | -1.56 (-1.89 to -1.23) | 1046344.60 (866979.25 to 1234220.25) | 867665.53 (679837.97 to 1060238.26) | 3270.52 (2709.52 to 3855.78) | 1306.94 (1012.44 to 1606.09) | -1.82(-2.16,-1.47) |
| Canada | 1666.29 (1081.91 to 2312.82) | 1916.82 (1207.51 to 2689.82) | 118.80 (82.39 to 158.47) | 74.15 (48.47 to 102.49) | -3.30 (-3.39 to -3.22) | 73958.28 (41989.88 to 109811.37) | 77792.36 (46167.48 to 111201.21) | 3891.63 (2593.06 to 5352.47) | 2368.31 (1491.27 to 3322.56) | -3.05(-3.13,-2.98) |
| Central African Republic | 2180.28 (1241.86 to 3232.29) | 3295.68 (1879.05 to 4933.57) | 60.53 (38.61 to 85.30) | 47.13 (29.94 to 67.55) | -1.66 (-1.82 to -1.50) | 105631.76 (51156.63 to 168299.53) | 154336.76 (78793.89 to 251268.57) | 2002.08 (1192.43 to 2897.38) | 1437.11 (882.37 to 2097.69) | -1.75(-1.91,-1.59) |
| Chad | 7753.90 (5818.81 to 9687.89) | 7141.56 (5340.66 to 9009.36) | 79.17 (58.53 to 99.67) | 27.96 (21.01 to 35.17) | -1.04 (-1.29 to -0.79) | 260628.84 (191273.33 to 327534.44) | 255560.54 (185792.02 to 326423.06) | 2424.13 (1788.85 to 3036.18) | 1053.65 (763.76 to 1345.52) | -1.28(-1.55,-1.01) |
| Chile | 1748800.39 (1378793.54 to 2138287.53) | 2667104.41 (2026749.65 to 3345860.16) | 261.48 (206.53 to 319.91) | 137.04 (103.36 to 171.24) | -3.18 (-3.26 to -3.11) | 50055027.97 (38470578.78 to 62620164.43) | 62703459.12 (47833506.74 to 79040372.01) | 6001.96 (4664.31 to 7392.42) | 3045.27 (2322.40 to 3829.21) | -2.57(-2.65,-2.49) |
| China | 11356.87 (8947.54 to 14086.80) | 13225.73 (9435.03 to 17845.96) | 65.31 (51.61 to 81.24) | 23.72 (16.98 to 31.96) | -2.21 (-2.31 to -2.11) | 385061.98 (284111.27 to 494798.33) | 382503.48 (275070.01 to 518920.37) | 1891.09 (1446.25 to 2388.70) | 695.09 (498.92 to 943.85) | -2.31(-2.37,-2.25) |
| Colombia | 223.62 (139.86 to 329.54) | 249.63 (164.13 to 344.62) | 104.34 (69.64 to 144.60) | 55.18 (36.85 to 77.37) | -3.77 (-3.95 to -3.58) | 9549.49 (5309.31 to 14594.05) | 8096.53 (5157.06 to 11249.64) | 3047.51 (1902.19 to 4401.43) | 1492.60 (973.25 to 2069.60) | -3.64(-3.80,-3.48) |
| Comoros | 975.77 (676.17 to 1331.40) | 1624.86 (1098.85 to 2202.46) | 88.65 (62.86 to 121.70) | 62.16 (43.21 to 83.28) | -2.42 (-2.62 to -2.21) | 34675.80 (22581.72 to 48072.98) | 56832.41 (37547.41 to 78134.56) | 2581.62 (1764.30 to 3542.50) | 1733.00 (1175.29 to 2343.20) | -2.68(-2.91,-2.46) |
| Congo | 17.02 (12.10 to 22.02) | 20.00 (14.22 to 26.69) | 141.77 (99.60 to 184.26) | 79.20 (55.80 to 105.95) | -1.30 (-1.50 to -1.10) | 557.28 (388.46 to 725.22) | 594.12 (429.59 to 796.97) | 4051.81 (2836.21 to 5257.47) | 2445.89 (1754.33 to 3299.23) | -1.47(-1.68,-1.26) |
| Cook Islands | 1064.27 (827.70 to 1312.18) | 1574.66 (1155.86 to 2041.85) | 62.60 (48.78 to 77.17) | 28.43 (20.90 to 36.79) | -2.04 (-2.17 to -1.92) | 31135.89 (23081.81 to 39721.64) | 46235.57 (33381.07 to 60458.02) | 1662.74 (1255.97 to 2090.14) | 841.18 (605.63 to 1102.10) | -1.76(-1.87,-1.65) |
| Costa Rica | 3662.32 (2179.83 to 5265.59) | 5409.16 (3211.63 to 7709.81) | 74.25 (49.18 to 103.03) | 46.21 (28.49 to 65.07) | -2.81 (-3.02 to -2.59) | 181533.22 (93818.97 to 277061.10) | 222641.82 (130064.76 to 320887.33) | 2299.19 (1441.73 to 3219.72) | 1376.73 (842.38 to 1945.24) | -2.47(-2.63,-2.32) |
| Croatia | 11141.81 (9161.00 to 13083.85) | 8735.41 (6866.84 to 10662.21) | 195.69 (159.05 to 232.77) | 94.54 (74.52 to 114.66) | -2.16 (-2.26 to -2.07) | 303169.53 (250172.59 to 356159.69) | 208641.66 (162866.62 to 253313.68) | 4992.19 (4091.22 to 5885.35) | 2588.40 (2022.88 to 3158.07) | -1.96(-2.03,-1.88) |
| Cuba | 12476.81 (10183.15 to 14893.12) | 15271.12 (11718.23 to 18797.71) | 125.39 (101.87 to 150.25) | 76.63 (59.00 to 93.92) | -1.77 (-1.90 to -1.64) | 331750.43 (268575.55 to 393092.58) | 391014.06 (306382.06 to 480454.39) | 3266.28 (2641.42 to 3875.89) | 2066.23 (1613.80 to 2543.40) | -1.66(-1.78,-1.53) |
| Cyprus | 898.86 (716.80 to 1077.45) | 1169.48 (924.82 to 1439.64) | 141.07 (110.50 to 172.82) | 58.53 (45.95 to 72.07) | -2.84 (-2.93 to -2.76) | 23548.08 (18721.43 to 28421.32) | 32411.43 (24925.37 to 40441.53) | 3151.28 (2495.39 to 3817.51) | 1637.07 (1252.07 to 2043.88) | -2.07(-2.13,-2.02) |
| Czechia | 24332.20 (20086.30 to 28892.08) | 15763.62 (12230.73 to 19357.81) | 178.25 (147.12 to 212.11) | 72.14 (56.13 to 88.38) | -2.83 (-2.90 to -2.75) | 676646.41 (553935.85 to 796764.92) | 419957.12 (325142.53 to 521239.14) | 5086.95 (4168.97 to 6010.95) | 2171.80 (1670.97 to 2712.90) | -2.67(-2.75,-2.58) |
| Democratic People's Republic of Korea | 25230.50 (18161.37 to 32790.42) | 42320.87 (31106.88 to 54127.62) | 174.17 (125.35 to 224.53) | 133.04 (96.56 to 171.60) | -0.96 (-1.09 to -0.83) | 791140.87 (564598.76 to 1039596.62) | 1218467.67 (890587.02 to 1561784.77) | 4559.56 (3267.57 to 5968.14) | 3640.41 (2662.51 to 4671.48) | -0.83(-0.97,-0.70) |
| Democratic Republic of the Congo | 11895.38 (7016.36 to 17217.62) | 15231.57 (9875.76 to 21480.16) | 61.97 (40.79 to 86.71) | 39.05 (25.66 to 55.75) | -1.53 (-1.65 to -1.40) | 572582.31 (285229.87 to 876503.77) | 595568.13 (378435.53 to 845846.52) | 2006.52 (1237.04 to 2870.37) | 1193.03 (787.65 to 1668.28) | -1.70(-1.80,-1.61) |
| Denmark | 15738.65 (13494.96 to 18114.90) | 9368.80 (7568.65 to 11182.52) | 190.85 (163.96 to 218.86) | 73.33 (59.37 to 87.00) | -3.35 (-3.47 to -3.24) | 371520.37 (311887.93 to 430992.53) | 210845.92 (166452.20 to 253966.06) | 4906.74 (4100.73 to 5705.53) | 1898.75 (1485.33 to 2317.82) | -3.33(-3.44,-3.22) |
| Djibouti | 171.72 (111.71 to 248.44) | 493.18 (315.47 to 713.47) | 115.23 (78.67 to 163.45) | 84.71 (54.68 to 119.55) | -1.04 (-1.14 to -0.94) | 7625.62 (4624.50 to 11181.74) | 17712.25 (11104.91 to 25289.59) | 3376.31 (2257.82 to 4834.92) | 2267.10 (1472.95 to 3203.15) | -1.36(-1.51,-1.21) |
| Dominica | 31.35 (23.37 to 39.84) | 31.61 (23.12 to 42.62) | 53.33 (39.63 to 67.77) | 38.21 (27.65 to 51.67) | -1.08 (-1.33 to -0.84) | 822.26 (604.68 to 1052.00) | 913.56 (664.08 to 1229.67) | 1413.81 (1043.29 to 1804.67) | 1088.61 (778.66 to 1475.05) | -0.79(-1.03,-0.55) |
| Dominican Republic | 2799.34 (2109.75 to 3529.82) | 6361.99 (4636.01 to 8585.53) | 82.91 (63.39 to 105.08) | 64.58 (46.93 to 87.00) | -0.34 (-0.62 to -0.07) | 86805.99 (63054.74 to 111834.88) | 176750.77 (129850.75 to 235556.30) | 2110.26 (1575.43 to 2670.53) | 1731.79 (1273.18 to 2307.70) | -0.30(-0.48,-0.12) |
| Ecuador | 2977.67 (2256.21 to 3821.43) | 3605.49 (2539.84 to 4813.71) | 57.65 (43.89 to 73.77) | 23.02 (16.10 to 30.81) | -2.71 (-2.91 to -2.52) | 96736.86 (69149.62 to 127854.23) | 106718.07 (74859.98 to 142247.47) | 1559.70 (1165.43 to 2011.61) | 644.16 (452.24 to 858.19) | -2.70(-2.88,-2.52) |
| Egypt | 44278.35 (32646.62 to 55650.10) | 78336.83 (57969.56 to 98736.19) | 170.03 (129.07 to 212.43) | 142.95 (104.98 to 180.97) | -0.20 (-0.39 to -0.02) | 1727962.29 (1140691.53 to 2322108.08) | 2553427.80 (1912763.37 to 3239702.46) | 4642.82 (3393.88 to 5881.81) | 3644.76 (2685.73 to 4612.00) | -0.47(-0.62,-0.32) |
| El Salvador | 920.23 (656.31 to 1202.27) | 1548.75 (1056.24 to 2126.26) | 29.66 (21.43 to 38.46) | 24.80 (17.01 to 33.83) | -0.57 (-0.75 to -0.39) | 32858.94 (22191.58 to 44190.08) | 48853.22 (32696.91 to 66727.24) | 944.71 (667.63 to 1232.21) | 803.74 (539.24 to 1096.62) | -0.47(-0.65,-0.29) |
| Equatorial Guinea | 256.86 (153.81 to 369.39) | 256.85 (147.45 to 375.00) | 108.14 (70.22 to 150.70) | 50.65 (29.71 to 72.47) | -2.75 (-3.25 to -2.25) | 11810.99 (6135.95 to 17583.40) | 9769.75 (5490.22 to 14413.39) | 3553.13 (2171.98 to 5029.49) | 1436.01 (841.31 to 2068.44) | -3.33(-3.87,-2.79) |
| Eritrea | 1260.34 (794.57 to 1858.52) | 1439.51 (892.57 to 2122.77) | 79.24 (51.96 to 112.74) | 43.84 (27.44 to 63.61) | -2.13 (-2.24 to -2.03) | 59490.10 (35109.22 to 90438.89) | 60495.49 (36134.43 to 89270.78) | 2790.81 (1788.02 to 4022.91) | 1435.58 (891.37 to 2083.56) | -2.37(-2.47,-2.27) |
| Estonia | 2818.67 (2350.09 to 3343.99) | 1333.18 (1062.54 to 1607.18) | 139.55 (115.67 to 166.17) | 51.10 (41.26 to 61.15) | -3.78 (-4.16 to -3.40) | 82684.37 (68822.47 to 97355.46) | 37505.06 (29502.59 to 46406.40) | 4156.93 (3443.62 to 4904.93) | 1661.07 (1301.56 to 2070.06) | -3.52(-3.86,-3.18) |
| Eswatini | 281.41 (191.36 to 392.04) | 369.84 (230.18 to 526.03) | 97.79 (67.76 to 136.18) | 66.80 (43.42 to 92.98) | -0.86 (-1.43 to -0.29) | 10337.03 (6542.25 to 14890.54) | 12631.92 (7587.14 to 18204.24) | 2654.38 (1812.35 to 3657.32) | 1872.12 (1192.23 to 2653.76) | -0.80(-1.42,-0.17) |
| Ethiopia | 14798.02 (9419.41 to 20829.29) | 8394.62 (5487.42 to 11786.04) | 59.11 (40.87 to 79.84) | 18.65 (12.36 to 26.06) | -3.85 (-4.12 to -3.58) | 660894.94 (374396.90 to 984565.67) | 317087.55 (200498.43 to 455176.65) | 1966.47 (1291.70 to 2714.95) | 557.96 (365.10 to 784.41) | -4.23(-4.50,-3.97) |
| Fiji | 637.16 (473.67 to 809.86) | 915.46 (632.78 to 1289.42) | 171.76 (125.54 to 220.53) | 120.24 (81.84 to 170.29) | -1.38 (-1.52 to -1.24) | 22980.27 (16846.33 to 29515.74) | 30964.00 (21536.77 to 42849.65) | 5098.29 (3752.43 to 6505.02) | 3590.35 (2471.48 to 4977.22) | -1.28(-1.39,-1.17) |
| Finland | 5981.89 (4936.06 to 7151.83) | 4069.81 (3234.87 to 4978.56) | 85.14 (70.48 to 101.36) | 32.07 (25.63 to 38.91) | -3.06 (-3.10 to -3.01) | 172305.48 (140748.81 to 207538.44) | 109281.30 (84258.97 to 136773.70) | 2568.83 (2100.50 to 3093.38) | 1059.39 (814.08 to 1329.57) | -2.74(-2.79,-2.69) |
| France | 68793.47 (57067.38 to 81550.91) | 54939.74 (43448.93 to 65971.56) | 83.79 (70.05 to 98.78) | 39.64 (32.09 to 46.88) | -2.32 (-2.37 to -2.26) | 1815706.22 (1470907.20 to 2166806.46) | 1497201.16 (1175932.36 to 1824313.27) | 2401.85 (1942.05 to 2869.33) | 1322.81 (1036.27 to 1623.40) | -1.79(-1.83,-1.76) |
| Gabon | 358.24 (237.16 to 492.68) | 500.56 (320.78 to 683.36) | 61.02 (40.91 to 83.07) | 47.88 (30.50 to 64.34) | -0.84 (-0.94 to -0.74) | 12685.64 (7844.60 to 18197.82) | 17364.35 (10891.56 to 24067.97) | 1855.34 (1219.51 to 2557.16) | 1408.01 (902.74 to 1911.81) | -0.93(-1.03,-0.83) |
| Gambia | 409.69 (251.76 to 580.44) | 576.19 (358.13 to 808.54) | 97.47 (63.97 to 135.55) | 58.28 (35.97 to 81.87) | -1.83 (-1.99 to -1.68) | 18937.77 (10582.47 to 27897.12) | 20512.79 (12903.96 to 28915.89) | 3044.40 (1964.05 to 4253.70) | 1662.79 (1073.51 to 2306.22) | -2.17(-2.34,-1.99) |
| Georgia | 9078.71 (7294.51 to 10871.30) | 6533.84 (5263.29 to 7968.95) | 149.31 (118.42 to 179.52) | 110.01 (89.10 to 133.28) | -0.84 (-1.03 to -0.64) | 279724.10 (222213.50 to 339133.63) | 174187.88 (141613.26 to 209911.13) | 4608.99 (3588.61 to 5642.52) | 3178.60 (2586.51 to 3820.29) | -1.21(-1.40,-1.01) |
| Germany | 142091.00 (118050.31 to 167769.11) | 98990.51 (79833.72 to 119097.98) | 111.73 (92.89 to 131.19) | 50.90 (41.67 to 60.88) | -2.48 (-2.65 to -2.31) | 3728061.91 (3059018.87 to 4441597.58) | 2581741.65 (2038514.13 to 3163574.49) | 3159.39 (2590.74 to 3774.19) | 1584.51 (1242.94 to 1962.14) | -2.15(-2.27,-2.03) |
| Ghana | 2796.02 (1857.05 to 3836.12) | 5573.58 (3738.54 to 7724.96) | 43.74 (30.68 to 59.30) | 35.38 (23.55 to 48.64) | -0.10 (-0.28 to 0.08) | 111329.73 (69642.47 to 159300.49) | 193728.00 (128651.64 to 271090.10) | 1270.01 (861.06 to 1720.33) | 973.18 (666.11 to 1342.53) | -0.24(-0.43,-0.05) |
| Greece | 20050.75 (17024.47 to 23246.27) | 19853.84 (16288.48 to 23675.04) | 134.70 (113.72 to 156.85) | 79.92 (66.82 to 92.87) | -1.72 (-1.82 to -1.62) | 507703.23 (429762.93 to 590282.83) | 467302.36 (379247.67 to 556107.18) | 3497.42 (2945.30 to 4093.49) | 2333.32 (1893.55 to 2765.21) | -1.31(-1.38,-1.23) |
| Greenland | 93.37 (77.02 to 109.23) | 84.71 (67.26 to 103.95) | 287.68 (235.46 to 340.29) | 122.93 (96.87 to 152.21) | -2.75 (-2.83 to -2.68) | 3058.37 (2457.63 to 3616.17) | 2602.85 (2059.91 to 3180.47) | 7832.11 (6329.02 to 9216.36) | 3480.93 (2761.10 to 4276.83) | -2.65(-2.72,-2.59) |
| Grenada | 41.79 (31.16 to 54.86) | 39.20 (27.99 to 50.68) | 59.22 (44.67 to 76.87) | 34.60 (24.35 to 45.19) | -1.98 (-2.27 to -1.69) | 1167.51 (860.68 to 1522.66) | 1229.68 (882.04 to 1612.53) | 1769.94 (1313.38 to 2282.04) | 1026.56 (731.25 to 1353.76) | -1.89(-2.02,-1.75) |
| Guam | 66.62 (52.57 to 81.80) | 125.32 (97.17 to 157.93) | 89.92 (68.70 to 114.03) | 60.17 (46.63 to 75.56) | -0.87 (-1.12 to -0.62) | 2359.52 (1846.36 to 2935.94) | 4337.47 (3360.24 to 5438.18) | 2578.63 (2009.18 to 3226.55) | 2172.24 (1667.04 to 2738.07) | -0.23(-0.41,-0.06) |
| Guatemala | 2211.60 (1430.81 to 3041.34) | 3056.63 (1995.11 to 4206.80) | 63.69 (43.95 to 85.65) | 29.07 (19.05 to 39.81) | -2.83 (-3.02 to -2.65) | 92724.81 (52676.40 to 135556.90) | 101750.10 (64327.50 to 142942.89) | 1744.57 (1154.62 to 2386.98) | 858.84 (549.61 to 1194.44) | -2.53(-2.69,-2.37) |
| Guinea | 2850.17 (1557.16 to 4304.17) | 3056.00 (1869.67 to 4388.02) | 66.78 (42.82 to 95.23) | 51.24 (32.21 to 71.84) | -0.69 (-0.83 to -0.55) | 145378.01 (62891.93 to 235585.27) | 117405.96 (66000.88 to 175523.24) | 2330.09 (1312.60 to 3455.36) | 1502.03 (932.18 to 2129.31) | -1.24(-1.38,-1.10) |
| Guinea-Bissau | 255.02 (150.72 to 373.14) | 370.62 (238.32 to 525.16) | 54.47 (34.38 to 77.72) | 46.80 (30.62 to 65.80) | -0.01 (-0.24 to 0.23) | 10904.75 (5891.77 to 16731.81) | 14336.77 (8987.45 to 20515.54) | 1755.24 (1075.79 to 2525.59) | 1414.99 (921.09 to 1998.89) | -0.23(-0.45,-0.01) |
| Guyana | 351.17 (262.86 to 460.09) | 310.66 (208.07 to 438.09) | 89.07 (66.84 to 116.41) | 48.10 (32.27 to 67.82) | -1.73 (-1.83 to -1.62) | 12149.95 (8854.20 to 16044.76) | 10813.31 (7281.68 to 15010.40) | 2689.00 (2000.83 to 3527.10) | 1533.93 (1030.62 to 2125.14) | -1.54(-1.63,-1.46) |
| Haiti | 2891.69 (1945.04 to 3974.41) | 2933.51 (1911.99 to 4113.22) | 78.64 (54.81 to 105.44) | 40.55 (26.24 to 56.97) | -2.03 (-2.24 to -1.82) | 124426.09 (74170.46 to 182460.53) | 107562.65 (68365.94 to 152310.68) | 2581.36 (1744.24 to 3529.96) | 1203.79 (784.56 to 1689.97) | -2.37(-2.60,-2.13) |
| Honduras | 1417.77 (1013.19 to 1865.73) | 4139.19 (3047.15 to 5466.21) | 67.39 (49.20 to 87.48) | 71.70 (52.49 to 94.52) | 0.48 (0.29 to 0.67) | 53185.26 (34401.11 to 73279.79) | 119433.06 (85456.40 to 161178.07) | 1949.33 (1412.25 to 2548.34) | 1806.77 (1302.86 to 2428.72) | -0.07(-0.21,0.08) |
| Hungary | 25383.79 (21501.26 to 29489.52) | 17003.40 (13619.96 to 20817.71) | 178.02 (150.88 to 206.15) | 90.74 (73.11 to 110.42) | -2.31 (-2.41 to -2.20) | 768299.45 (644538.53 to 890774.13) | 487956.82 (390868.04 to 594461.25) | 5529.10 (4627.91 to 6423.21) | 2897.55 (2311.62 to 3537.13) | -2.26(-2.37,-2.15) |
| Iceland | 320.94 (260.70 to 390.16) | 258.01 (199.17 to 323.60) | 110.41 (90.30 to 133.49) | 42.70 (33.38 to 52.79) | -3.13 (-3.20 to -3.05) | 8204.17 (6576.42 to 9902.25) | 6781.33 (5216.09 to 8539.20) | 2978.00 (2389.56 to 3587.71) | 1267.89 (971.63 to 1607.24) | -2.87(-2.94,-2.81) |
| India | 668815.44 (487709.91 to 858332.58) | 1048266.44 (787047.61 to 1312373.19) | 151.10 (111.53 to 193.26) | 96.80 (72.16 to 121.78) | -1.34 (-1.46 to -1.22) | 24339373.34 (16648002.98 to 32470832.77) | 28926123.72 (21277795.81 to 36185564.46) | 4255.43 (3070.83 to 5481.49) | 2404.42 (1763.90 to 3028.25) | -1.81(-1.88,-1.75) |
| Indonesia | 119066.71 (89382.83 to 149635.63) | 268613.94 (200911.81 to 341175.44) | 124.35 (93.39 to 157.60) | 126.02 (93.69 to 159.33) | 0.14 (-0.03 to 0.31) | 4367440.73 (3135016.27 to 5620430.16) | 8315677.48 (6211884.61 to 10554870.73) | 3611.97 (2715.19 to 4589.34) | 3247.53 (2420.50 to 4129.14) | -0.24(-0.36,-0.11) |
| Iran (Islamic Republic of) | 19565.86 (15171.84 to 24020.77) | 34675.90 (27530.06 to 41888.74) | 76.84 (60.15 to 93.75) | 46.44 (36.38 to 57.00) | -1.49 (-1.58 to -1.40) | 701635.92 (515183.90 to 893979.76) | 1131188.64 (878169.50 to 1389422.79) | 2222.37 (1693.23 to 2768.85) | 1332.05 (1028.20 to 1632.17) | -1.50(-1.58,-1.42) |
| Iraq | 13932.13 (10536.76 to 17768.96) | 27305.28 (19772.37 to 35830.37) | 173.48 (132.18 to 217.68) | 130.96 (94.86 to 169.84) | -1.44 (-1.63 to -1.25) | 452553.09 (325117.72 to 585760.71) | 848110.79 (608513.16 to 1106473.43) | 4807.36 (3658.99 to 6068.45) | 3310.55 (2404.84 to 4289.68) | -1.64(-1.78,-1.51) |
| Ireland | 7772.62 (6449.61 to 9128.17) | 3836.36 (2990.95 to 4688.59) | 191.80 (158.11 to 225.64) | 46.69 (36.64 to 56.74) | -4.65 (-4.84 to -4.47) | 179895.07 (146237.93 to 212672.56) | 96032.32 (74744.85 to 118134.11) | 4493.56 (3643.89 to 5324.54) | 1272.07 (986.11 to 1574.84) | -4.18(-4.32,-4.04) |
| Israel | 4289.13 (3500.61 to 5137.54) | 3985.33 (3140.28 to 4869.59) | 91.31 (74.46 to 110.19) | 31.46 (25.00 to 38.18) | -3.53 (-3.61 to -3.46) | 116712.64 (93972.69 to 140604.56) | 113779.63 (86608.34 to 142000.80) | 2488.71 (2000.55 to 2990.25) | 991.61 (754.40 to 1247.30) | -3.06(-3.12,-3.00) |
| Italy | 89899.93 (74570.66 to 104806.95) | 61364.83 (48497.45 to 73948.33) | 101.72 (84.33 to 118.76) | 39.69 (32.05 to 47.03) | -3.07 (-3.12 to -3.02) | 2349407.76 (1920132.57 to 2773601.40) | 1468733.98 (1149614.08 to 1813350.10) | 2802.89 (2279.31 to 3310.34) | 1178.62 (912.28 to 1458.45) | -2.80(-2.84,-2.76) |
| Jamaica | 1022.97 (780.33 to 1281.75) | 1258.55 (861.45 to 1757.68) | 57.31 (43.98 to 71.68) | 40.13 (27.55 to 55.73) | -1.34 (-1.71 to -0.98) | 28258.82 (21388.36 to 35132.34) | 35074.39 (24080.71 to 48222.69) | 1627.80 (1246.47 to 2016.36) | 1138.73 (781.28 to 1566.89) | -1.42(-1.77,-1.08) |
| Japan | 140471.79 (116085.44 to 167328.73) | 142774.64 (113120.60 to 172675.35) | 85.57 (70.23 to 102.75) | 35.77 (29.17 to 42.54) | -2.93 (-2.98 to -2.89) | 3849424.19 (3083082.78 to 4669064.20) | 3171730.77 (2486318.10 to 3887456.80) | 2296.89 (1829.47 to 2799.79) | 1102.10 (852.22 to 1351.69) | -2.49(-2.54,-2.43) |
| Jordan | 1769.32 (1339.64 to 2230.73) | 4289.86 (3112.12 to 5454.36) | 140.70 (107.56 to 176.86) | 64.54 (47.18 to 82.81) | -2.90 (-3.14 to -2.67) | 63582.63 (46764.19 to 81703.83) | 167548.32 (124067.34 to 215654.68) | 3810.29 (2920.71 to 4782.04) | 1950.63 (1451.78 to 2521.22) | -2.49(-2.67,-2.31) |
| Kazakhstan | 19422.69 (15796.83 to 23562.05) | 15059.51 (11903.49 to 18215.48) | 150.32 (120.94 to 182.91) | 86.78 (67.87 to 106.03) | -2.46 (-2.93 to -1.99) | 664326.90 (521186.79 to 810968.96) | 474416.50 (370606.17 to 579156.23) | 4732.69 (3749.97 to 5762.73) | 2472.96 (1925.42 to 3029.67) | -2.87(-3.39,-2.34) |
| Kenya | 6140.36 (4011.10 to 8885.68) | 9418.10 (6551.17 to 12869.20) | 66.40 (46.52 to 94.01) | 42.24 (29.64 to 57.13) | -1.59 (-1.81 to -1.37) | 267627.36 (152069.69 to 403928.07) | 331967.41 (227139.75 to 460606.29) | 1952.51 (1333.60 to 2744.89) | 1189.23 (832.62 to 1630.75) | -1.68(-1.93,-1.44) |
| Kiribati | 111.04 (83.38 to 141.33) | 199.57 (145.95 to 257.28) | 293.39 (222.14 to 373.73) | 277.25 (202.16 to 358.83) | -0.32 (-0.51 to -0.12) | 4078.46 (2951.37 to 5223.70) | 7207.36 (5211.35 to 9366.05) | 8871.36 (6616.07 to 11214.64) | 8299.38 (6021.03 to 10723.19) | -0.33(-0.51,-0.15) |
| Kuwait | 552.50 (441.54 to 665.72) | 1548.64 (1129.02 to 1984.88) | 89.06 (69.99 to 107.95) | 53.39 (38.31 to 68.98) | -1.42 (-1.86 to -0.98) | 22223.55 (17149.51 to 27247.91) | 64760.09 (47566.23 to 82834.46) | 2591.04 (2019.13 to 3166.02) | 1614.86 (1192.20 to 2101.18) | -1.31(-1.67,-0.95) |
| Kyrgyzstan | 5095.27 (3849.47 to 6284.25) | 5322.24 (4203.89 to 6737.06) | 168.00 (131.48 to 204.50) | 119.28 (93.17 to 149.78) | -0.86 (-1.17 to -0.55) | 178606.98 (122468.63 to 228726.59) | 165665.12 (130704.90 to 209152.54) | 5074.46 (3768.71 to 6313.24) | 3149.54 (2484.73 to 3961.56) | -1.55(-1.85,-1.24) |
| Lao People's Democratic Republic | 6029.12 (4153.08 to 7983.92) | 6503.40 (4606.11 to 8370.98) | 265.81 (193.63 to 338.33) | 156.56 (111.92 to 200.26) | -1.80 (-1.87 to -1.73) | 250814.87 (149067.55 to 355435.69) | 203662.98 (143035.77 to 267419.08) | 7990.08 (5521.77 to 10570.84) | 4000.28 (2844.56 to 5157.07) | -2.32(-2.39,-2.25) |
| Latvia | 5218.46 (4378.90 to 6179.49) | 2735.22 (2143.05 to 3337.68) | 147.93 (123.69 to 175.53) | 72.66 (57.69 to 87.47) | -2.69 (-2.98 to -2.39) | 151635.37 (127014.78 to 178795.88) | 75264.35 (60036.64 to 91893.96) | 4380.65 (3655.27 to 5172.79) | 2288.80 (1824.72 to 2801.44) | -2.60(-2.91,-2.29) |
| Lebanon | 3269.31 (2476.19 to 4152.83) | 5689.35 (4295.29 to 7155.22) | 158.39 (120.70 to 200.32) | 90.14 (68.26 to 113.36) | -1.58 (-1.72 to -1.45) | 100953.13 (75839.58 to 128137.25) | 151228.07 (113567.90 to 189835.91) | 4336.86 (3274.24 to 5488.53) | 2519.98 (1894.62 to 3163.05) | -1.54(-1.67,-1.40) |
| Lesotho | 1062.02 (738.22 to 1468.17) | 2167.85 (1501.03 to 2865.21) | 125.21 (87.66 to 172.50) | 196.95 (136.01 to 264.54) | 2.37 (1.92 to 2.81) | 34800.36 (23438.95 to 49381.58) | 74124.13 (51306.24 to 97790.99) | 3490.94 (2412.86 to 4815.91) | 5845.20 (4066.62 to 7695.37) | 2.56(2.08,3.03) |
| Liberia | 686.94 (374.15 to 1043.70) | 659.57 (407.42 to 953.31) | 46.02 (29.21 to 64.68) | 28.66 (17.70 to 40.52) | -1.75 (-1.87 to -1.63) | 35257.02 (16221.07 to 58201.29) | 26524.74 (16111.42 to 38534.07) | 1625.41 (933.05 to 2391.61) | 893.05 (561.84 to 1264.16) | -2.14(-2.29,-1.99) |
| Libya | 1731.41 (1318.97 to 2231.44) | 4775.53 (3390.54 to 6253.00) | 92.55 (70.14 to 119.21) | 93.54 (66.59 to 122.10) | 0.55 (0.34 to 0.76) | 56774.17 (42556.81 to 73167.87) | 160929.42 (115513.24 to 210012.93) | 2576.47 (1974.78 to 3288.72) | 2630.66 (1890.28 to 3416.23) | 0.50(0.33,0.66) |
| Lithuania | 5933.73 (4940.07 to 7011.87) | 4019.52 (3236.42 to 4879.61) | 133.05 (110.49 to 157.39) | 71.89 (58.17 to 86.52) | -2.12 (-2.38 to -1.86) | 167628.80 (139623.62 to 196929.61) | 106209.24 (85424.22 to 129683.91) | 3811.18 (3169.85 to 4480.64) | 2196.62 (1765.43 to 2674.71) | -1.91(-2.19,-1.63) |
| Luxembourg | 621.56 (497.72 to 748.96) | 481.30 (370.33 to 600.01) | 114.73 (91.80 to 137.87) | 44.45 (34.48 to 55.06) | -3.04 (-3.11 to -2.96) | 16691.79 (13239.01 to 20157.69) | 13334.74 (9999.85 to 16577.18) | 3190.75 (2520.85 to 3843.58) | 1326.57 (990.39 to 1651.45) | -2.91(-2.97,-2.84) |
| Madagascar | 6755.48 (4268.71 to 9416.77) | 5484.85 (3474.08 to 7942.54) | 112.42 (78.98 to 151.48) | 46.66 (30.41 to 67.01) | -3.02 (-3.24 to -2.81) | 318397.42 (172930.33 to 465683.78) | 229631.26 (140308.64 to 333997.74) | 3562.48 (2341.89 to 4910.69) | 1381.34 (890.10 to 1988.51) | -3.20(-3.40,-2.99) |
| Malawi | 3901.35 (2450.53 to 5392.55) | 5419.43 (3790.37 to 7232.89) | 91.31 (64.65 to 120.85) | 73.67 (52.33 to 97.44) | -0.97 (-1.25 to -0.69) | 172308.68 (91978.16 to 259761.92) | 188103.61 (125831.03 to 255004.28) | 2643.54 (1755.39 to 3572.15) | 2047.43 (1437.22 to 2712.47) | -1.14(-1.43,-0.84) |
| Malaysia | 12006.99 (9444.93 to 14549.27) | 24106.43 (18717.66 to 29742.07) | 134.73 (106.07 to 164.46) | 91.34 (69.90 to 113.82) | -1.36 (-1.55 to -1.17) | 359865.71 (276625.53 to 443063.92) | 696414.24 (542977.49 to 855862.37) | 3525.61 (2761.96 to 4286.75) | 2371.87 (1836.19 to 2936.35) | -1.32(-1.44,-1.19) |
| Maldives | 163.87 (127.29 to 202.12) | 202.93 (151.85 to 260.36) | 211.12 (169.43 to 257.00) | 68.78 (51.54 to 87.83) | -3.97 (-4.15 to -3.80) | 5740.18 (4142.17 to 7346.52) | 6199.69 (4593.73 to 7886.90) | 5316.39 (4138.72 to 6527.70) | 1636.35 (1222.44 to 2084.19) | -4.20(-4.45,-3.94) |
| Mali | 2162.55 (1259.51 to 3161.77) | 4273.09 (2573.70 to 6108.39) | 50.11 (32.29 to 70.30) | 50.05 (32.03 to 69.47) | 0.32 (0.21 to 0.44) | 96719.52 (47913.15 to 149032.96) | 168823.98 (93107.26 to 249736.56) | 1485.17 (896.78 to 2142.11) | 1376.01 (859.85 to 1918.62) | 0.01(-0.11,0.12) |
| Malta | 450.15 (366.11 to 537.77) | 346.54 (271.52 to 429.76) | 106.68 (86.23 to 127.99) | 36.65 (29.07 to 44.90) | -3.48 (-3.56 to -3.40) | 12539.13 (10037.30 to 15031.79) | 9901.47 (7593.30 to 12388.24) | 2931.68 (2343.99 to 3522.12) | 1255.71 (956.27 to 1571.73) | -2.76(-2.86,-2.65) |
| Marshall Islands | 31.34 (22.55 to 40.68) | 57.54 (39.30 to 77.56) | 183.66 (130.98 to 239.06) | 162.20 (108.52 to 217.42) | -0.30 (-0.40 to -0.20) | 1165.21 (817.26 to 1525.83) | 2181.93 (1500.66 to 2937.90) | 5436.72 (3960.47 to 7040.67) | 4941.10 (3357.46 to 6615.21) | -0.25(-0.36,-0.14) |
| Mauritania | 860.55 (538.80 to 1230.41) | 831.84 (496.60 to 1188.12) | 77.17 (49.30 to 108.42) | 38.72 (23.45 to 55.76) | -2.42 (-2.60 to -2.24) | 36322.74 (21079.44 to 53029.95) | 29948.34 (17535.59 to 42550.76) | 2444.82 (1570.76 to 3423.89) | 1125.10 (673.58 to 1587.86) | -2.66(-2.79,-2.53) |
| Mauritius | 998.54 (782.29 to 1204.30) | 1301.48 (998.62 to 1606.82) | 143.79 (111.23 to 175.01) | 73.21 (55.98 to 90.81) | -2.31 (-2.58 to -2.03) | 30597.67 (23968.09 to 37257.38) | 38120.38 (28870.37 to 47244.54) | 3882.46 (3017.96 to 4724.64) | 2121.85 (1600.26 to 2638.58) | -2.02(-2.31,-1.73) |
| Mexico | 34246.80 (25349.04 to 42885.21) | 39508.80 (29268.46 to 50730.83) | 88.31 (66.32 to 109.33) | 32.74 (24.25 to 42.34) | -3.48 (-3.66 to -3.30) | 1158675.38 (778145.57 to 1518008.31) | 1160878.70 (840372.96 to 1507325.23) | 2266.07 (1648.72 to 2868.43) | 897.24 (649.65 to 1166.65) | -3.28(-3.49,-3.07) |
| Micronesia (Federated States of) | 120.01 (86.20 to 159.56) | 145.78 (105.03 to 194.34) | 240.60 (174.80 to 318.31) | 193.01 (136.21 to 255.34) | -0.74 (-0.78 to -0.70) | 4311.89 (3049.81 to 5847.74) | 5217.58 (3727.23 to 6879.21) | 7315.03 (5296.79 to 9749.83) | 5975.12 (4247.04 to 7866.15) | -0.65(-0.69,-0.61) |
| Monaco | 72.62 (53.17 to 92.49) | 74.22 (58.73 to 93.43) | 104.90 (77.02 to 131.52) | 76.23 (60.44 to 97.61) | -0.96 (-1.08 to -0.83) | 1736.02 (1301.58 to 2174.64) | 1760.32 (1368.99 to 2235.86) | 2957.62 (2224.88 to 3662.07) | 2180.85 (1684.29 to 2812.43) | -0.94(-1.05,-0.83) |
| Mongolia | 1972.16 (1406.84 to 2571.80) | 2690.81 (2055.31 to 3349.05) | 164.63 (125.81 to 206.41) | 116.77 (87.07 to 145.66) | -1.33 (-1.54 to -1.12) | 83065.99 (50016.57 to 116196.12) | 93615.63 (71223.48 to 116661.48) | 5317.23 (3764.39 to 6890.28) | 3406.38 (2585.41 to 4259.33) | -1.62(-1.82,-1.42) |
| Montenegro | 883.44 (737.55 to 1029.90) | 1325.04 (1054.14 to 1608.08) | 142.21 (118.09 to 167.06) | 139.34 (110.31 to 169.40) | -0.06 (-0.28 to 0.16) | 27888.90 (23244.92 to 32439.49) | 36516.82 (29159.60 to 44270.84) | 4329.19 (3602.03 to 5055.44) | 3843.58 (3063.20 to 4671.34) | -0.39(-0.66,-0.12) |
| Morocco | 15334.84 (11397.64 to 19512.92) | 21574.26 (15101.53 to 28041.31) | 104.61 (79.22 to 131.96) | 65.32 (45.26 to 85.55) | -1.61 (-1.77 to -1.45) | 551674.38 (384618.29 to 727476.62) | 666349.89 (480637.47 to 870673.60) | 3135.32 (2313.19 to 3998.92) | 1839.80 (1328.27 to 2420.13) | -1.82(-1.97,-1.66) |
| Mozambique | 5227.23 (3331.98 to 7482.70) | 7363.05 (5121.64 to 10098.38) | 75.27 (51.89 to 101.13) | 62.56 (43.49 to 85.86) | 0.01 (-0.21 to 0.22) | 239694.81 (127805.88 to 372393.45) | 278124.57 (184175.84 to 384858.78) | 2365.75 (1544.84 to 3317.57) | 1863.20 (1298.94 to 2532.89) | -0.13(-0.36,0.10) |
| Myanmar | 72952.48 (51887.23 to 94504.35) | 56841.35 (41488.73 to 74418.17) | 331.31 (243.07 to 425.21) | 131.80 (96.29 to 173.76) | -3.29 (-3.42 to -3.15) | 2544234.90 (1672093.79 to 3400034.75) | 1559219.98 (1124217.50 to 2047306.79) | 9170.68 (6361.05 to 11935.76) | 3179.36 (2297.90 to 4179.13) | -3.73(-3.88,-3.59) |
| Namibia | 868.41 (609.43 to 1170.32) | 1099.65 (732.65 to 1486.26) | 149.61 (106.15 to 199.17) | 90.33 (62.76 to 119.01) | -1.88 (-2.23 to -1.52) | 28874.16 (18697.98 to 40484.35) | 33326.56 (21522.48 to 46179.17) | 3804.69 (2672.51 to 5145.38) | 2252.77 (1504.21 to 3033.23) | -1.96(-2.32,-1.60) |
| Nauru | 14.72 (10.57 to 18.89) | 15.41 (10.85 to 20.43) | 306.15 (221.52 to 387.75) | 249.14 (174.76 to 325.66) | -0.81 (-1.12 to -0.50) | 561.27 (393.57 to 725.90) | 584.24 (405.12 to 771.30) | 9186.83 (6659.03 to 11635.45) | 7861.39 (5475.21 to 10379.29) | -0.65(-1.00,-0.29) |
| Nepal | 24727.52 (17590.64 to 33193.01) | 28110.20 (20351.73 to 36598.89) | 262.93 (193.54 to 344.34) | 140.22 (101.13 to 181.95) | -2.10 (-2.30 to -1.90) | 1010346.96 (645812.45 to 1408516.59) | 748485.95 (541101.30 to 968759.62) | 7404.31 (5311.18 to 9853.65) | 3250.50 (2352.61 to 4195.16) | -2.78(-2.94,-2.62) |
| Netherlands | 27008.71 (22974.49 to 31293.95) | 21305.74 (17268.82 to 25820.26) | 134.26 (114.30 to 155.19) | 57.06 (46.46 to 68.43) | -2.81 (-2.90 to -2.73) | 675327.21 (567070.99 to 784317.27) | 493948.16 (395719.75 to 597383.01) | 3513.00 (2945.81 to 4082.67) | 1502.34 (1186.25 to 1826.01) | -2.76(-2.83,-2.68) |
| New Zealand | 4274.27 (3487.68 to 5060.96) | 3580.41 (2864.29 to 4369.34) | 108.77 (88.76 to 129.14) | 41.15 (33.03 to 50.02) | -3.26 (-3.43 to -3.09) | 117541.57 (93578.57 to 142033.89) | 95004.18 (73951.41 to 118356.60) | 3069.42 (2437.59 to 3709.23) | 1219.14 (941.92 to 1526.74) | -3.11(-3.30,-2.93) |
| Nicaragua | 758.52 (497.72 to 1034.80) | 1280.49 (888.81 to 1719.07) | 43.22 (30.93 to 56.43) | 27.56 (19.07 to 37.14) | -1.05 (-1.25 to -0.84) | 34169.71 (18896.91 to 50391.53) | 44472.20 (30573.99 to 59986.84) | 1374.49 (928.17 to 1852.23) | 844.76 (581.39 to 1144.81) | -1.26(-1.43,-1.09) |
| Niger | 2253.72 (1017.75 to 3732.46) | 2250.40 (1185.67 to 3373.53) | 42.47 (24.77 to 62.94) | 25.13 (15.18 to 35.39) | -1.82 (-1.94 to -1.70) | 151911.85 (57699.67 to 263902.42) | 108239.63 (51450.11 to 169132.58) | 1630.83 (827.64 to 2547.05) | 721.45 (414.03 to 1049.18) | -2.87(-3.09,-2.66) |
| Nigeria | 17243.65 (9612.42 to 25887.43) | 15925.91 (9495.97 to 23334.53) | 30.82 (20.02 to 44.43) | 15.40 (10.09 to 21.89) | -2.40 (-2.60 to -2.21) | 883529.76 (402312.86 to 1417385.05) | 716752.03 (381094.31 to 1077491.95) | 1070.20 (611.11 to 1590.42) | 492.53 (303.25 to 703.05) | -2.64(-2.85,-2.43) |
| Niue | 2.89 (2.08 to 3.87) | 2.47 (1.79 to 3.33) | 131.29 (95.13 to 174.30) | 116.62 (83.54 to 158.25) | -0.66 (-0.76 to -0.56) | 81.79 (58.85 to 109.01) | 74.49 (53.56 to 101.18) | 3890.39 (2797.95 to 5177.92) | 3588.56 (2505.03 to 4869.35) | -0.64(-0.76,-0.52) |
| North Macedonia | 3376.11 (2685.99 to 4003.22) | 4145.96 (3182.18 to 5204.25) | 188.45 (147.63 to 225.69) | 142.72 (109.34 to 178.83) | -1.15 (-1.55 to -0.75) | 100928.97 (80359.15 to 120210.94) | 115925.63 (88145.91 to 144568.91) | 5227.52 (4116.40 to 6281.65) | 3620.10 (2761.51 to 4510.67) | -1.38(-1.64,-1.12) |
| Northern Mariana Islands | 24.87 (18.15 to 32.82) | 50.92 (39.64 to 63.12) | 143.37 (106.32 to 185.27) | 101.51 (76.55 to 127.55) | -1.13 (-1.19 to -1.07) | 980.89 (693.46 to 1294.42) | 1684.59 (1301.39 to 2116.23) | 3945.03 (2917.08 to 5079.49) | 2903.13 (2231.20 to 3658.14) | -0.97(-1.06,-0.88) |
| Norway | 7093.29 (5714.96 to 8479.22) | 3724.18 (2905.28 to 4655.10) | 102.81 (83.70 to 121.79) | 35.11 (27.71 to 43.36) | -3.78 (-3.95 to -3.60) | 176437.52 (141471.01 to 211905.32) | 93983.47 (72280.04 to 118373.15) | 2885.83 (2319.31 to 3447.45) | 1033.15 (792.86 to 1307.01) | -3.60(-3.74,-3.47) |
| Oman | 586.22 (408.75 to 818.64) | 756.45 (519.05 to 1021.62) | 84.13 (59.20 to 117.10) | 41.68 (28.65 to 57.17) | -1.80 (-1.97 to -1.62) | 21597.55 (14860.21 to 30499.46) | 30664.00 (21208.40 to 40716.92) | 2365.25 (1671.75 to 3303.57) | 1128.12 (771.57 to 1510.26) | -2.04(-2.16,-1.92) |
| Pakistan | 95893.47 (71106.31 to 120606.17) | 130593.43 (93139.05 to 170702.35) | 169.56 (125.19 to 211.92) | 115.99 (83.63 to 151.04) | -1.51 (-1.80 to -1.22) | 3352236.48 (2247984.79 to 4421801.95) | 4266738.09 (3023781.89 to 5631482.13) | 4679.24 (3462.92 to 5904.04) | 3069.28 (2216.33 to 4016.35) | -1.60(-1.88,-1.32) |
| Palau | 14.93 (10.84 to 19.68) | 26.33 (18.74 to 35.02) | 157.15 (113.76 to 207.30) | 123.99 (87.56 to 165.38) | -0.75 (-0.79 to -0.70) | 488.25 (347.55 to 647.70) | 881.50 (626.83 to 1179.90) | 4472.53 (3196.47 to 5897.77) | 3628.14 (2542.50 to 4801.50) | -0.68(-0.72,-0.63) |
| Palestine | 1273.91 (924.61 to 1642.49) | 2084.49 (1595.19 to 2627.27) | 158.11 (115.78 to 202.31) | 94.47 (70.92 to 120.87) | -1.89 (-2.15 to -1.63) | 37497.18 (26691.36 to 48783.63) | 66820.43 (49898.80 to 83773.30) | 3807.03 (2784.57 to 4903.60) | 2401.65 (1800.34 to 3009.97) | -1.68(-1.87,-1.49) |
| Panama | 726.80 (567.00 to 917.54) | 990.60 (689.81 to 1368.34) | 50.64 (39.45 to 63.84) | 22.12 (15.45 to 30.52) | -2.95 (-3.11 to -2.80) | 20792.30 (15383.46 to 26752.51) | 27781.77 (19375.95 to 38094.24) | 1319.10 (1000.21 to 1684.25) | 630.10 (438.61 to 864.07) | -2.64(-2.80,-2.49) |
| Papua New Guinea | 3400.45 (2079.72 to 4822.15) | 7067.74 (4586.87 to 9889.07) | 171.99 (110.11 to 241.69) | 134.84 (86.71 to 189.88) | -0.82 (-0.87 to -0.77) | 151772.72 (83077.16 to 220382.96) | 292771.90 (180709.72 to 412785.31) | 5317.89 (3305.85 to 7474.38) | 4078.88 (2631.16 to 5697.97) | -0.84(-0.90,-0.78) |
| Paraguay | 2079.52 (1636.78 to 2588.66) | 3955.11 (2742.06 to 5313.84) | 96.82 (76.23 to 120.15) | 70.79 (49.20 to 94.78) | -0.90 (-1.01 to -0.79) | 61448.30 (45516.87 to 78964.44) | 108470.35 (74474.16 to 146156.76) | 2514.35 (1948.46 to 3162.71) | 1819.25 (1249.97 to 2456.57) | -1.06(-1.14,-0.97) |
| Peru | 4929.61 (3185.32 to 6700.95) | 6799.94 (4711.14 to 9744.17) | 38.08 (25.74 to 50.26) | 20.18 (14.04 to 28.91) | -2.53 (-2.90 to -2.15) | 191933.68 (111207.69 to 276166.00) | 198963.84 (136449.06 to 278467.88) | 1171.66 (743.75 to 1600.45) | 574.11 (394.35 to 803.67) | -2.72(-3.08,-2.36) |
| Philippines | 47736.51 (36164.90 to 60230.95) | 88169.07 (65040.26 to 112151.87) | 171.87 (131.06 to 214.92) | 112.24 (82.66 to 142.42) | -1.16 (-1.31 to -1.00) | 1823304.32 (1275240.89 to 2391968.33) | 2834140.51 (2090208.71 to 3582486.44) | 4743.10 (3569.72 to 5981.27) | 3141.62 (2316.11 to 3969.64) | -1.21(-1.34,-1.09) |
| Poland | 80256.84 (68388.17 to 92165.24) | 56127.48 (45853.76 to 67900.04) | 186.39 (158.06 to 215.05) | 77.90 (63.78 to 93.71) | -2.93 (-3.01 to -2.85) | 2429472.66 (2042219.52 to 2823702.70) | 1588398.95 (1285323.12 to 1928372.74) | 5603.27 (4697.67 to 6527.52) | 2416.26 (1948.56 to 2942.40) | -2.77(-2.85,-2.68) |
| Portugal | 11869.69 (9432.85 to 14270.37) | 8086.73 (6164.25 to 10086.58) | 87.95 (69.40 to 106.12) | 34.28 (26.87 to 41.81) | -3.19 (-3.31 to -3.07) | 331339.34 (259231.75 to 401346.75) | 230958.47 (172713.21 to 289573.60) | 2523.65 (1957.75 to 3078.17) | 1204.55 (901.47 to 1505.33) | -2.48(-2.56,-2.40) |
| Puerto Rico | 2082.28 (1601.90 to 2675.84) | 1922.00 (1362.36 to 2625.86) | 59.13 (45.24 to 76.44) | 26.33 (18.99 to 35.61) | -2.96 (-3.11 to -2.81) | 55992.08 (42173.39 to 72464.03) | 50630.67 (36377.03 to 68700.61) | 1564.97 (1175.86 to 2028.17) | 846.03 (610.41 to 1148.70) | -2.30(-2.43,-2.16) |
| Qatar | 129.37 (95.65 to 167.64) | 401.07 (274.78 to 548.39) | 133.54 (96.37 to 174.51) | 49.16 (32.47 to 67.12) | -3.59 (-4.23 to -2.95) | 5189.31 (3787.21 to 6874.99) | 21997.62 (15019.88 to 29933.09) | 3243.28 (2346.72 to 4275.87) | 1360.28 (901.67 to 1854.30) | -3.04(-3.50,-2.58) |
| Republic of Korea | 38125.39 (29837.95 to 45535.30) | 44308.16 (34863.74 to 54266.55) | 148.87 (114.24 to 180.65) | 47.63 (37.41 to 58.44) | -4.01 (-4.17 to -3.86) | 1197190.19 (939982.13 to 1443671.77) | 1135442.04 (863468.64 to 1410547.77) | 3773.80 (2920.23 to 4566.26) | 1259.27 (949.64 to 1569.50) | -3.83(-3.95,-3.71) |
| Republic of Moldova | 5700.96 (4639.66 to 6860.97) | 5375.71 (4346.14 to 6467.56) | 133.65 (108.34 to 162.45) | 90.79 (73.23 to 109.06) | -1.49 (-1.80 to -1.19) | 178171.78 (142692.26 to 216233.52) | 159933.07 (129193.86 to 192690.71) | 3940.36 (3141.64 to 4804.03) | 2823.65 (2267.77 to 3405.09) | -1.23(-1.55,-0.92) |
| Romania | 37455.68 (30467.42 to 43911.99) | 29481.60 (23006.25 to 35791.04) | 144.28 (115.34 to 171.20) | 83.83 (65.82 to 101.01) | -2.29 (-2.51 to -2.07) | 1190611.85 (944439.89 to 1421869.26) | 865118.33 (667762.34 to 1051649.24) | 4474.63 (3456.28 to 5423.54) | 2750.76 (2113.73 to 3351.76) | -2.08(-2.30,-1.86) |
| Russian Federation | 242841.57 (207712.59 to 278082.74) | 224007.55 (184165.23 to 265289.47) | 137.02 (116.30 to 158.36) | 94.83 (78.11 to 112.17) | -1.72 (-2.37 to -1.06) | 7505912.19 (6373587.22 to 8623420.09) | 6667009.92 (5489318.27 to 7888658.00) | 4181.37 (3518.66 to 4840.41) | 2965.84 (2432.70 to 3511.79) | -1.68(-2.32,-1.03) |
| Rwanda | 5204.40 (3622.23 to 6945.60) | 4758.18 (3379.88 to 6378.48) | 181.25 (131.14 to 234.49) | 88.18 (63.18 to 118.66) | -3.26 (-3.63 to -2.89) | 196848.58 (120127.56 to 280338.76) | 148050.93 (102653.58 to 201785.43) | 5010.08 (3508.04 to 6658.48) | 2177.18 (1546.75 to 2919.60) | -3.70(-4.10,-3.30) |
| Saint Kitts and Nevis | 21.94 (16.27 to 28.41) | 19.19 (13.40 to 25.96) | 60.27 (44.29 to 78.00) | 29.94 (20.63 to 40.38) | -2.40 (-2.54 to -2.25) | 561.52 (411.47 to 722.76) | 586.56 (406.67 to 792.20) | 1621.73 (1207.40 to 2078.59) | 806.83 (559.56 to 1092.62) | -2.48(-2.66,-2.31) |
| Saint Lucia | 59.75 (45.42 to 75.80) | 76.41 (53.10 to 101.71) | 72.05 (54.44 to 91.59) | 32.09 (22.19 to 42.72) | -3.12 (-3.39 to -2.86) | 1727.87 (1305.11 to 2185.30) | 2262.23 (1586.93 to 3018.14) | 1962.97 (1493.13 to 2479.69) | 939.71 (656.77 to 1258.17) | -2.74(-2.92,-2.57) |
| Saint Vincent and the Grenadines | 34.92 (25.91 to 44.60) | 49.19 (35.28 to 64.03) | 50.14 (37.05 to 63.94) | 35.51 (25.39 to 46.29) | -1.31 (-1.44 to -1.17) | 1035.48 (753.64 to 1334.09) | 1485.11 (1058.46 to 1930.92) | 1438.94 (1063.21 to 1846.33) | 1047.74 (741.73 to 1368.92) | -1.21(-1.34,-1.09) |
| Samoa | 145.44 (107.30 to 186.63) | 204.39 (151.79 to 259.90) | 176.49 (130.60 to 226.27) | 146.55 (108.30 to 186.51) | -0.72 (-0.80 to -0.64) | 4778.81 (3434.16 to 6190.75) | 6652.92 (4836.21 to 8569.81) | 4925.49 (3656.33 to 6281.17) | 4241.93 (3104.94 to 5467.86) | -0.58(-0.66,-0.50) |
| San Marino | 26.96 (21.29 to 33.44) | 20.73 (13.41 to 29.94) | 75.36 (59.65 to 93.27) | 26.47 (16.69 to 38.58) | -2.56 (-2.87 to -2.25) | 689.28 (544.12 to 855.32) | 573.08 (389.17 to 803.39) | 2087.82 (1638.02 to 2586.59) | 915.25 (620.07 to 1278.39) | -2.17(-2.36,-1.98) |
| Sao Tome and Principe | 20.72 (13.15 to 29.18) | 31.59 (21.57 to 43.56) | 29.98 (19.94 to 41.53) | 28.22 (19.35 to 38.00) | -0.32 (-0.60 to -0.03) | 774.77 (451.69 to 1137.76) | 1089.33 (731.60 to 1537.19) | 949.80 (607.83 to 1335.91) | 824.17 (561.36 to 1131.15) | -0.65(-0.92,-0.38) |
| Saudi Arabia | 4646.21 (3131.04 to 6399.09) | 14238.04 (10063.96 to 18994.17) | 75.60 (51.38 to 103.97) | 61.56 (42.66 to 81.38) | -0.60 (-0.78 to -0.42) | 173700.39 (115549.51 to 240258.90) | 604757.05 (422194.08 to 802469.24) | 2181.29 (1493.42 to 2976.42) | 1918.24 (1345.68 to 2516.97) | -0.23(-0.41,-0.04) |
| Senegal | 3032.35 (1847.48 to 4321.49) | 3170.95 (1918.01 to 4482.17) | 74.86 (49.61 to 103.20) | 40.75 (24.67 to 57.96) | -2.15 (-2.26 to -2.05) | 145319.05 (75466.57 to 215631.45) | 113863.19 (68556.93 to 159183.92) | 2522.61 (1618.64 to 3494.51) | 1206.87 (735.86 to 1675.26) | -2.56(-2.67,-2.45) |
| Serbia | 17707.77 (14584.05 to 20920.52) | 17736.16 (13971.68 to 21777.85) | 172.88 (140.07 to 208.13) | 108.01 (85.62 to 132.36) | -1.84 (-2.14 to -1.53) | 532321.26 (434820.16 to 626043.75) | 481214.77 (377658.33 to 585672.80) | 4710.59 (3821.51 to 5569.15) | 3210.02 (2517.81 to 3903.19) | -1.50(-1.78,-1.23) |
| Seychelles | 78.63 (61.33 to 95.10) | 90.71 (69.75 to 113.96) | 139.71 (109.25 to 169.07) | 82.78 (63.19 to 104.81) | -1.54 (-1.70 to -1.37) | 2131.00 (1637.09 to 2585.76) | 2709.66 (2037.83 to 3419.96) | 3760.08 (2907.06 to 4533.54) | 2260.70 (1694.06 to 2854.46) | -1.53(-1.68,-1.38) |
| Sierra Leone | 2393.94 (1322.82 to 3489.33) | 1964.35 (1208.87 to 2844.76) | 86.84 (54.27 to 121.14) | 48.71 (30.75 to 69.75) | -1.72 (-1.92 to -1.52) | 125638.41 (56642.68 to 198995.95) | 78384.20 (45280.59 to 116759.85) | 3100.08 (1818.01 to 4409.40) | 1502.16 (953.22 to 2139.34) | -2.28(-2.46,-2.10) |
| Singapore | 2027.58 (1596.97 to 2465.40) | 1954.37 (1506.49 to 2422.55) | 96.14 (74.88 to 118.63) | 22.81 (17.52 to 28.41) | -4.51 (-4.57 to -4.45) | 61672.67 (47304.65 to 75577.40) | 60222.52 (45513.29 to 76820.56) | 2577.19 (1980.14 to 3164.41) | 692.95 (520.13 to 886.50) | -4.12(-4.18,-4.06) |
| Slovakia | 9396.51 (7629.97 to 11123.01) | 7020.90 (5485.67 to 8649.04) | 159.79 (129.23 to 189.48) | 74.02 (57.48 to 91.46) | -2.34 (-2.44 to -2.25) | 276116.47 (223608.79 to 328269.44) | 199702.50 (155266.21 to 247763.57) | 4735.71 (3820.63 to 5639.10) | 2235.39 (1723.27 to 2782.77) | -2.31(-2.40,-2.22) |
| Slovenia | 2429.27 (1956.07 to 2893.89) | 1926.52 (1476.47 to 2442.20) | 98.82 (79.18 to 117.91) | 43.56 (33.74 to 54.72) | -2.72 (-2.83 to -2.62) | 75233.38 (59672.69 to 90713.82) | 57029.35 (42542.79 to 72016.57) | 3102.50 (2442.15 to 3751.48) | 1502.54 (1107.32 to 1911.42) | -2.35(-2.43,-2.26) |
| Solomon Islands | 341.01 (217.22 to 460.67) | 727.78 (509.10 to 961.92) | 245.11 (164.44 to 325.05) | 208.47 (145.35 to 271.55) | -0.39 (-0.58 to -0.20) | 13007.75 (8056.86 to 17733.78) | 26318.88 (18226.50 to 34685.72) | 7009.73 (4533.93 to 9397.56) | 6105.56 (4278.32 to 8030.03) | -0.30(-0.49,-0.10) |
| Somalia | 3438.86 (2029.08 to 4979.77) | 4998.44 (3042.49 to 7239.88) | 110.22 (72.62 to 153.05) | 69.38 (44.44 to 97.77) | -1.53 (-1.58 to -1.48) | 168585.22 (89355.83 to 261003.95) | 218149.24 (124661.50 to 323853.62) | 3440.96 (2169.57 to 4933.36) | 2117.61 (1337.86 to 2986.59) | -1.63(-1.69,-1.58) |
| South Africa | 27558.47 (20194.81 to 34563.18) | 32441.76 (23965.76 to 40991.81) | 124.88 (93.24 to 156.23) | 70.22 (51.28 to 88.93) | -1.94 (-2.31 to -1.56) | 1088216.61 (743792.82 to 1419513.60) | 1100532.76 (803060.86 to 1409409.96) | 4119.30 (2975.99 to 5204.77) | 2147.05 (1556.43 to 2749.72) | -2.09(-2.44,-1.73) |
| South Sudan | 2643.88 (1659.64 to 3877.18) | 2531.87 (1568.51 to 3691.05) | 89.17 (59.32 to 126.88) | 61.30 (39.88 to 87.47) | -1.40 (-1.64 to -1.16) | 113432.53 (62089.28 to 176096.03) | 102479.06 (58158.87 to 151929.44) | 2774.18 (1777.16 to 3986.13) | 1786.65 (1113.62 to 2558.89) | -1.57(-1.86,-1.28) |
| Spain | 59302.36 (48727.39 to 69419.17) | 45137.05 (35888.06 to 54808.61) | 110.22 (90.47 to 129.33) | 44.55 (36.29 to 53.26) | -2.92 (-3.03 to -2.82) | 1517574.07 (1241840.42 to 1783577.95) | 1149425.34 (916922.05 to 1395568.54) | 2961.57 (2413.44 to 3481.12) | 1354.35 (1080.54 to 1643.82) | -2.51(-2.61,-2.41) |
| Sri Lanka | 10522.85 (8155.99 to 13100.07) | 11934.79 (7090.04 to 17303.08) | 115.09 (87.79 to 145.97) | 47.80 (29.01 to 69.03) | -2.52 (-2.66 to -2.38) | 300656.58 (231057.83 to 374774.55) | 322909.49 (202865.96 to 462196.84) | 2706.21 (2076.21 to 3372.89) | 1218.12 (771.52 to 1737.11) | -2.48(-2.60,-2.35) |
| Sudan | 12805.29 (8873.14 to 17049.75) | 15144.26 (10507.01 to 21535.71) | 129.45 (93.22 to 168.63) | 81.34 (56.85 to 114.88) | -1.67 (-1.74 to -1.60) | 496812.27 (305397.38 to 693901.70) | 495864.27 (340675.08 to 708097.06) | 3798.75 (2641.66 to 5040.21) | 2158.09 (1496.48 to 3044.34) | -1.99(-2.06,-1.92) |
| Suriname | 243.76 (189.18 to 300.87) | 332.29 (238.32 to 464.95) | 94.89 (73.56 to 117.64) | 51.95 (37.16 to 73.05) | -2.03 (-2.28 to -1.77) | 7928.19 (6077.01 to 9892.67) | 11033.79 (7840.74 to 15079.68) | 2824.04 (2175.86 to 3502.84) | 1674.72 (1191.15 to 2296.83) | -1.83(-2.06,-1.60) |
| Sweden | 13444.31 (10747.71 to 16324.42) | 8928.23 (6866.63 to 11213.26) | 86.10 (69.27 to 103.66) | 37.53 (29.31 to 46.93) | -2.55 (-2.64 to -2.46) | 313918.52 (249164.96 to 379820.54) | 209993.38 (158318.61 to 266597.97) | 2260.43 (1786.06 to 2757.23) | 1061.14 (799.41 to 1361.81) | -2.35(-2.41,-2.29) |
| Switzerland | 10141.38 (8229.16 to 12067.56) | 7347.92 (5936.82 to 8958.42) | 95.81 (78.28 to 113.68) | 37.20 (30.30 to 44.69) | -2.90 (-3.01 to -2.80) | 255680.52 (204921.38 to 307809.83) | 189096.84 (146554.99 to 235961.04) | 2638.57 (2114.38 to 3175.20) | 1157.63 (892.72 to 1443.10) | -2.59(-2.68,-2.51) |
| Syrian Arab Republic | 8545.54 (6353.57 to 11059.27) | 13133.10 (9029.13 to 17760.87) | 163.61 (123.05 to 211.22) | 112.35 (78.49 to 147.34) | -1.56 (-1.71 to -1.41) | 292548.55 (212542.25 to 382444.74) | 402969.93 (283102.79 to 541431.61) | 4541.94 (3414.44 to 5801.47) | 2893.39 (2043.13 to 3860.80) | -1.74(-1.90,-1.58) |
| Taiwan (Province of China) | 16505.66 (13290.61 to 19512.31) | 25393.55 (19850.46 to 31058.53) | 115.51 (91.32 to 138.14) | 58.97 (46.35 to 71.84) | -2.19 (-2.29 to -2.08) | 489556.72 (388012.76 to 591472.98) | 651884.65 (495765.23 to 793859.41) | 2986.53 (2360.58 to 3601.81) | 1616.16 (1227.31 to 1975.75) | -2.01(-2.08,-1.94) |
| Tajikistan | 4762.73 (3406.30 to 6166.45) | 3539.32 (2524.65 to 4853.11) | 156.33 (119.48 to 195.02) | 65.65 (47.12 to 89.67) | -2.95 (-3.26 to -2.65) | 188215.37 (116007.48 to 262360.95) | 125854.65 (87363.37 to 175004.71) | 4718.78 (3370.11 to 6037.74) | 1790.49 (1260.29 to 2465.12) | -3.35(-3.57,-3.12) |
| Thailand | 42786.85 (33352.43 to 52050.61) | 66326.91 (47160.61 to 91476.60) | 133.34 (103.54 to 161.47) | 61.70 (43.79 to 85.01) | -2.97 (-3.13 to -2.80) | 1276845.91 (971777.58 to 1563981.89) | 1765610.00 (1261357.04 to 2420387.11) | 3391.00 (2594.09 to 4132.81) | 1694.53 (1203.95 to 2320.40) | -2.67(-2.83,-2.52) |
| Timor-Leste | 582.71 (368.33 to 808.83) | 1001.63 (707.58 to 1323.31) | 160.55 (117.31 to 217.65) | 127.76 (90.48 to 169.22) | -0.60 (-0.79 to -0.40) | 31526.02 (16328.97 to 46292.91) | 30955.13 (21257.00 to 41059.94) | 5004.73 (3385.72 to 6776.88) | 3322.77 (2336.71 to 4385.37) | -1.22(-1.49,-0.95) |
| Togo | 1246.99 (809.97 to 1731.72) | 2296.30 (1493.61 to 3173.31) | 92.13 (64.05 to 124.55) | 58.90 (39.27 to 80.18) | -1.52 (-1.63 to -1.40) | 51853.44 (29357.03 to 73924.56) | 82697.85 (53011.70 to 115223.25) | 2692.53 (1805.10 to 3650.50) | 1715.16 (1121.92 to 2357.02) | -1.51(-1.65,-1.38) |
| Tokelau | 1.91 (1.35 to 2.52) | 1.57 (1.08 to 2.17) | 146.22 (103.37 to 191.61) | 107.98 (74.24 to 149.76) | -1.20 (-1.28 to -1.12) | 56.34 (38.95 to 74.84) | 48.23 (33.29 to 66.70) | 4172.87 (2907.43 to 5498.06) | 3454.65 (2359.04 to 4787.97) | -0.96(-1.08,-0.85) |
| Tonga | 86.87 (65.12 to 111.21) | 107.53 (77.30 to 145.41) | 168.44 (126.53 to 216.18) | 136.93 (98.47 to 184.35) | -0.72 (-0.88 to -0.56) | 2641.78 (1894.93 to 3443.74) | 2996.39 (2131.74 to 4038.82) | 4376.41 (3230.72 to 5642.04) | 3619.54 (2598.75 to 4842.22) | -0.66(-0.79,-0.53) |
| Trinidad and Tobago | 786.10 (596.48 to 973.42) | 892.27 (586.11 to 1226.82) | 97.49 (73.08 to 121.63) | 46.33 (30.27 to 63.80) | -2.84 (-3.08 to -2.60) | 23839.65 (17825.99 to 29527.89) | 27903.75 (18443.33 to 37830.57) | 2739.16 (2047.22 to 3389.57) | 1465.27 (964.44 to 1986.68) | -2.46(-2.69,-2.23) |
| Tunisia | 6061.59 (4754.06 to 7499.37) | 11046.70 (7941.30 to 15215.04) | 135.40 (106.69 to 168.80) | 88.44 (63.89 to 120.64) | -1.67 (-1.80 to -1.54) | 185288.99 (138661.23 to 235183.09) | 305986.98 (226754.30 to 411967.03) | 3409.72 (2620.26 to 4244.23) | 2281.36 (1685.92 to 3066.75) | -1.53(-1.62,-1.45) |
| Türkiye | 67233.36 (52036.48 to 81031.23) | 85332.39 (64084.60 to 110193.06) | 198.28 (154.13 to 237.36) | 94.49 (70.66 to 122.26) | -2.52 (-2.83 to -2.22) | 2220407.01 (1647220.07 to 2739487.63) | 2394409.26 (1816366.35 to 3065298.10) | 5576.54 (4260.64 to 6724.70) | 2512.70 (1903.05 to 3213.30) | -2.77(-3.04,-2.50) |
| Turkmenistan | 3517.59 (2630.15 to 4429.38) | 3940.72 (2811.18 to 5179.41) | 169.05 (133.35 to 206.67) | 99.41 (70.92 to 130.73) | -2.34 (-2.68 to -2.00) | 142200.42 (93222.51 to 192320.15) | 131193.23 (93441.19 to 174398.00) | 5276.16 (3971.33 to 6648.42) | 2904.16 (2091.42 to 3836.02) | -2.49(-2.85,-2.14) |
| Tuvalu | 15.57 (11.09 to 20.39) | 16.53 (12.29 to 21.33) | 225.70 (162.35 to 295.51) | 162.98 (119.78 to 209.64) | -1.00 (-1.06 to -0.95) | 574.91 (385.42 to 774.24) | 540.96 (398.48 to 708.78) | 6994.24 (4921.14 to 9243.44) | 4903.12 (3613.90 to 6394.73) | -1.07(-1.15,-0.99) |
| Uganda | 4130.58 (2652.35 to 6010.39) | 5284.43 (3526.28 to 7422.08) | 58.50 (40.37 to 82.43) | 35.85 (24.41 to 49.86) | -2.47 (-2.92 to -2.01) | 172597.01 (95631.13 to 262533.80) | 195194.16 (122305.74 to 273648.62) | 1687.64 (1130.51 to 2384.52) | 1015.58 (678.19 to 1408.59) | -2.55(-3.02,-2.08) |
| Ukraine | 110403.71 (91483.79 to 129883.58) | 74278.71 (52312.44 to 100581.72) | 159.17 (131.64 to 188.35) | 97.89 (69.03 to 133.05) | -2.19 (-2.72 to -1.66) | 3043884.89 (2541280.24 to 3547518.42) | 2071644.39 (1467813.04 to 2795121.65) | 4382.85 (3627.13 to 5126.86) | 2927.22 (2066.27 to 3960.14) | -1.90(-2.39,-1.41) |
| United Arab Emirates | 509.41 (358.19 to 690.29) | 1692.65 (1166.51 to 2274.90) | 116.73 (82.16 to 159.37) | 65.71 (44.49 to 90.24) | -0.55 (-1.09 to -0.00) | 21145.49 (14665.04 to 28611.58) | 90828.94 (61015.03 to 123058.07) | 3068.11 (2159.26 to 4142.51) | 1559.05 (1078.89 to 2101.36) | -1.42(-1.84,-0.99) |
| United Kingdom | 146743.12 (123958.73 to 169348.51) | 75847.93 (61005.04 to 91871.91) | 159.45 (134.73 to 183.41) | 55.05 (44.69 to 66.35) | -3.57 (-3.70 to -3.44) | 3487773.03 (2891416.77 to 4074554.55) | 1810575.79 (1404671.91 to 2215649.98) | 4124.33 (3401.76 to 4834.41) | 1525.82 (1177.56 to 1878.06) | -3.31(-3.42,-3.20) |
| United Republic of Tanzania | 13388.57 (8344.80 to 18859.33) | 13534.11 (9444.82 to 18647.53) | 101.98 (70.28 to 137.15) | 52.42 (37.02 to 71.83) | -2.62 (-2.79 to -2.44) | 641053.03 (336155.05 to 970225.89) | 500305.24 (338308.91 to 689748.29) | 3259.84 (2128.97 to 4506.09) | 1542.14 (1076.27 to 2104.05) | -2.86(-3.02,-2.69) |
| United States of America | 393882.76 (329926.24 to 460284.88) | 360369.51 (290297.49 to 433606.01) | 123.94 (104.24 to 144.32) | 60.64 (49.09 to 72.80) | -2.49 (-2.57 to -2.41) | 10913754.74 (8983097.96 to 12850160.02) | 10028633.80 (7905778.36 to 12210024.39) | 3631.06 (2990.99 to 4267.72) | 1858.88 (1447.71 to 2270.38) | -2.28(-2.33,-2.23) |
| United States Virgin Islands | 45.81 (32.64 to 62.39) | 49.94 (34.34 to 70.29) | 56.12 (39.80 to 77.22) | 28.30 (19.62 to 39.68) | -2.16 (-2.34 to -1.97) | 1456.32 (1028.82 to 1984.67) | 1413.22 (974.13 to 1956.55) | 1562.07 (1095.93 to 2123.47) | 879.11 (607.26 to 1219.28) | -1.80(-1.94,-1.66) |
| Uruguay | 4501.60 (3710.12 to 5230.71) | 3800.88 (2962.91 to 4559.75) | 117.15 (96.63 to 136.02) | 70.10 (55.52 to 83.06) | -1.88 (-1.98 to -1.78) | 128201.00 (104785.12 to 149769.25) | 103148.36 (81004.50 to 123994.47) | 3480.99 (2841.45 to 4070.79) | 2132.89 (1673.04 to 2570.86) | -1.80(-1.89,-1.71) |
| Uzbekistan | 9232.87 (6526.96 to 12108.28) | 16996.48 (12684.62 to 21544.85) | 72.79 (54.40 to 93.09) | 67.36 (49.39 to 86.25) | -0.28 (-0.81 to 0.24) | 368535.96 (234159.58 to 503910.27) | 588877.93 (425964.65 to 749432.78) | 2397.98 (1703.78 to 3129.72) | 1969.89 (1431.53 to 2501.33) | -0.74(-1.24,-0.23) |
| Vanuatu | 103.67 (75.39 to 138.13) | 199.45 (147.80 to 254.90) | 163.16 (119.73 to 216.79) | 113.11 (82.72 to 143.82) | -1.41 (-1.52 to -1.29) | 3794.68 (2664.35 to 5163.78) | 7109.30 (5181.35 to 9121.91) | 4657.24 (3411.22 to 6188.37) | 3364.03 (2462.96 to 4292.07) | -1.28(-1.39,-1.16) |
| Venezuela (Bolivarian Republic of) | 7765.14 (5914.68 to 9528.39) | 14085.41 (9484.65 to 19083.14) | 81.01 (61.62 to 98.91) | 47.62 (31.96 to 64.59) | -2.04 (-2.24 to -1.85) | 256155.37 (189222.61 to 321585.09) | 415026.04 (279326.68 to 559605.30) | 2293.63 (1737.00 to 2840.23) | 1351.91 (908.02 to 1829.60) | -2.01(-2.19,-1.83) |
| Viet Nam | 57068.58 (42412.99 to 75461.32) | 100341.56 (74363.63 to 123082.35) | 145.34 (108.30 to 193.38) | 106.98 (79.32 to 131.65) | -1.09 (-1.15 to -1.02) | 1724163.91 (1227546.82 to 2274729.86) | 2860560.17 (2119214.92 to 3575896.76) | 3843.65 (2850.47 to 5031.60) | 2765.09 (2038.88 to 3439.69) | -1.07(-1.10,-1.05) |
| Yemen | 9433.64 (6468.84 to 12685.18) | 16857.55 (11429.94 to 22794.19) | 172.05 (126.10 to 227.80) | 126.28 (85.94 to 169.98) | -1.16 (-1.25 to -1.08) | 392901.99 (241481.13 to 557223.40) | 565742.83 (383311.94 to 765347.76) | 5117.31 (3662.63 to 6802.74) | 3374.84 (2318.89 to 4520.45) | -1.52(-1.62,-1.43) |
| Zambia | 3332.62 (2063.78 to 4710.87) | 3814.40 (2461.26 to 5318.16) | 95.84 (67.55 to 129.06) | 56.86 (37.12 to 78.67) | -2.28 (-2.58 to -1.97) | 158884.60 (79441.66 to 244895.93) | 139287.55 (85174.46 to 196945.85) | 2899.33 (1916.09 to 3954.35) | 1549.61 (1009.67 to 2118.89) | -2.62(-2.94,-2.30) |
| Zimbabwe | 4568.25 (3234.73 to 6244.98) | 8695.80 (5914.36 to 11630.44) | 114.73 (82.53 to 154.68) | 124.90 (86.52 to 165.87) | 0.75 (0.20 to 1.30) | 161273.76 (106543.89 to 226838.29) | 310439.03 (203080.27 to 421687.60) | 3095.42 (2217.71 to 4201.71) | 3561.87 (2421.13 to 4763.95) | 0.87(0.31,1.44) |

ASDR, age-standardized Deaths rates; ASDALYR, age-standardized disability-adjusted life years rates; CI, confidence interval; DALYs, disability-adjusted life years rates; EAPC, estimated annual percentage change; UI, uncertainty interval.

Table S3 Deaths and DALYs of smoking in 1990 and 2021 at regional levels

| Region | Deaths (×1000, 95% UI) | | ASDR per 100,000 (95% UI) | | 1990-2021 EAPC,  (95% CI) | DALYs (×1000, 95% UI) | | ASDALYR per 100,000 (95% UI) | | 1990-2021 EAPC,  (95% CI) |
| --- | --- | --- | --- | --- | --- | --- | --- | --- | --- | --- |
|  | 1990 | 2021 | 1990 | 2021 |  | 1990 | 2021 | 1990 | 2021 |  |
| High SDI | 1212.59 (1039.58 to 1393.78) | 1061.57 (877.86 to 1248.89) | 109.59 (93.98 to 125.98) | 49.31 (41.01 to 57.55) | -2.68 (-2.72 to -2.63) | 33056.46 (27693.21 to 38694.91) | 27838.15 (22484.86 to 33301.17) | 3079.00 (2576.13 to 3611.91) | 1477.18 (1184.98 to 1773.25) | -2.43 (-2.46 to -2.40) |
| High-middle SDI | 1348.71 (1165.83 to 1544.15) | 1685.25 (1378.93 to 2006.55) | 141.15 (121.56 to 162.12) | 85.39 (69.79 to 101.65) | -1.87 (-2.07 to -1.68) | 38451.66 (33013.01 to 44305.82) | 43940.33 (36601.57 to 52069.34) | 3786.88 (3241.84 to 4367.97) | 2250.72 (1872.92 to 2661.29) | -1.93 (-2.11 to -1.74) |
| Middle SDI | 1344.8 (1128.63 to 1580.89) | 2073.57 (1674.75 to 2494.31) | 145.82 (121.74 to 171.38) | 82.17 (65.98 to 98.60) | -1.98 (-2.05 to -1.91) | 38702.32 (32742.98 to 45809.93) | 54276.33 (43958.25 to 64578.33) | 3598.21 (3029.81 to 4246.91) | 1993.02 (1609.87 to 2372.35) | -2.00 (-2.04 to -1.96) |
| Low-middle SDI | 699.71 (565 to 840.5) | 1109.9 (902.23 to 1325.61) | 122.30 (98.07 to 147.30) | 83.04 (67.21 to 99.91) | -1.18 (-1.25 to -1.12) | 21625.86 (17528.71 to 25917.76) | 31485.07 (25547.14 to 37544.45) | 3282.33 (2638.36 to 3938.66) | 2113.55 (1710.48 to 2525.23) | -1.39 (-1.43 to -1.35) |
| Low SDI | 173.23 (137.18 to 215.27) | 239.64 (186.87 to 295.02) | 82.39 (64.75 to 102.35) | 52.39 (40.53 to 65.17) | -1.46 (-1.56 to -1.36) | 5534.56 (4369.27 to 6898.14) | 7391.46 (5795.58 to 9080.17) | 2236.02 (1760.79 to 2789.16) | 1350.61 (1057.16 to 1664.63) | -1.73 (-1.81 to -1.65) |
| Andean Latin America | 7.58 (6.01 to 9.23) | 11.31 (8.6 to 14.72) | 38.01 (30.12 to 46.28) | 19.43 (14.78 to 25.27) | -2.20 (-2.41 to -1.99) | 230 (181.44 to 280.27) | 334.29 (252.24 to 431.12) | 1039.70 (817.35 to 1268.07) | 549.17 (414.57 to 707.14) | -2.12 (-2.34 to -1.90) |
| Australasia | 19.83 (16.6 to 23.16) | 15.32 (12.33 to 18.76) | 83.56 (69.87 to 97.64) | 28.00 (22.73 to 34.02) | -3.60 (-3.72 to -3.48) | 562.77 (452.87 to 671.22) | 435.81 (337.15 to 549.95) | 2420.62 (1945.84 to 2889.34) | 918.59 (706.42 to 1161.19) | -3.16 (-3.27 to -3.05) |
| Caribbean | 19.87 (16.64 to 23.39) | 26.86 (21.34 to 32.36) | 78.61 (65.55 to 92.91) | 49.57 (39.39 to 59.73) | -1.53 (-1.60 to -1.46) | 570.13 (470.16 to 672.41) | 748.64 (599.76 to 907.21) | 2151.26 (1774.05 to 2534.62) | 1390.47 (1113.32 to 1685.51) | -1.47 (-1.54 to -1.39) |
| Central Asia | 48.63 (41.81 to 55.43) | 55.02 (45.38 to 64.12) | 102.29 (87.67 to 117.39) | 68.37 (56.31 to 80.03) | -1.46 (-1.77 to -1.16) | 1560.75 (1337.57 to 1784.65) | 1758.71 (1437.98 to 2074.27) | 3109.78 (2662.25 to 3559.77) | 1965.28 (1608.20 to 2319.11) | -1.78 (-2.11 to -1.45) |
| Central Europe | 211.98 (183.79 to 239.45) | 164.46 (137.34 to 190.85) | 142.95 (123.48 to 161.97) | 75.45 (63.26 to 87.31) | -2.22 (-2.30 to -2.15) | 6576.42 (5622.27 to 7539.95) | 4765.69 (3929.14 to 5664.25) | 4392.38 (3747.42 to 5054.05) | 2407.59 (1977.46 to 2871.77) | -2.09 (-2.16 to -2.03) |
| Central Latin America | 46.87 (38.61 to 55.26) | 64.58 (51.38 to 78.32) | 60.78 (49.69 to 72.09) | 26.23 (20.80 to 31.90) | -3.03 (-3.16 to -2.89) | 1419.59 (1162.25 to 1690.57) | 1913.89 (1494.15 to 2357.01) | 1601.18 (1312.78 to 1908.07) | 745.50 (582.97 to 918.55) | -2.79 (-2.93 to -2.64) |
| Central Sub-Saharan Africa | 13 (9.84 to 16.61) | 21.46 (15.66 to 27.73) | 55.40 (41.84 to 70.37) | 36.21 (26.88 to 47.10) | -1.37 (-1.56 to -1.19) | 454.19 (341.05 to 585.18) | 792.07 (569.59 to 1036.28) | 1679.58 (1261.77 to 2148.36) | 1127.79 (822.48 to 1466.09) | -1.27 (-1.43 to -1.12) |
| East Asia | 1497.42 (1233.28 to 1799.58) | 2360.63 (1870.73 to 2957.49) | 212.71 (175.07 to 254.13) | 115.03 (90.85 to 142.91) | -2.09 (-2.19 to -1.99) | 40205.47 (33066.16 to 48005.18) | 56296.31 (44571.48 to 70508.55) | 4712.59 (3877.98 to 5653.84) | 2598.18 (2057.21 to 3260.32) | -1.98 (-2.04 to -1.92) |
| Eastern Europe | 329.89 (292.17 to 369.36) | 287.41 (240.33 to 336.24) | 118.19 (104.47 to 132.73) | 83.22 (69.67 to 97.30) | -1.65 (-2.22 to -1.09) | 10211.33 (8930.15 to 11495.86) | 8699.88 (7270.14 to 10162.55) | 3647.50 (3186.21 to 4118.29) | 2659.92 (2222.21 to 3105.74) | -1.57 (-2.14 to -1.00) |
| Eastern Sub-Saharan Africa | 48.45 (37.9 to 61.54) | 58.75 (46.12 to 73.03) | 67.28 (52.53 to 86.07) | 35.22 (27.74 to 44.06) | -2.34 (-2.44 to -2.24) | 1610.41 (1251.62 to 2055.86) | 2032.43 (1578.61 to 2575.54) | 1892.71 (1466.56 to 2407.36) | 1012.43 (792.41 to 1276.98) | -2.25 (-2.34 to -2.16) |
| High-income Asia Pacific | 162.14 (137.25 to 187.84) | 175.3 (143.44 to 208.66) | 83.47 (70.32 to 97.16) | 34.42 (28.36 to 40.34) | -3.01 (-3.06 to -2.96) | 4664.43 (3818.68 to 5518.5) | 4059.1 (3275.05 to 4912.43) | 2292.57 (1874.01 to 2719.09) | 1027.80 (807.97 to 1249.11) | -2.73 (-2.79 to -2.68) |
| High-income North America | 406.6 (345.96 to 469.74) | 374.91 (307.11 to 446.73) | 115.92 (98.89 to 133.46) | 55.97 (46.08 to 66.52) | -2.52 (-2.59 to -2.44) | 11307.05 (9437.18 to 13217.94) | 10351.19 (8303.13 to 12508.54) | 3394.07 (2836.13 to 3959.67) | 1705.27 (1352.53 to 2066.35) | -2.33 (-2.38 to -2.29) |
| North Africa and Middle East | 172.06 (144.07 to 198.97) | 293.55 (236.5 to 352.91) | 107.22 (89.19 to 124.79) | 68.15 (54.70 to 82.25) | -1.53 (-1.59 to -1.46) | 5435.62 (4535.6 to 6339.08) | 9391.77 (7463.02 to 11239.56) | 2938.50 (2446.20 to 3437.91) | 1874.78 (1491.07 to 2241.65) | -1.52 (-1.57 to -1.46) |
| Oceania | 3.87 (2.9 to 4.95) | 7.42 (5.62 to 9.48) | 129.81 (97.75 to 167.37) | 94.30 (71.21 to 121.31) | -1.12 (-1.17 to -1.07) | 134.82 (99.21 to 171.3) | 270.14 (204.3 to 344.14) | 3815.88 (2846.75 to 4842.04) | 2908.95 (2180.25 to 3713.16) | -0.95 (-1.00 to -0.91) |
| South Asia | 677.58 (545.28 to 824.82) | 1065.69 (843.42 to 1295.68) | 128.30 (101.23 to 157.12) | 79.03 (62.32 to 96.09) | -1.51 (-1.60 to -1.42) | 21031.33 (16893.71 to 25514.88) | 29233.38 (23125.39 to 35409.99) | 3384.42 (2696.85 to 4115.66) | 1940.77 (1540.64 to 2356.34) | -1.80 (-1.86 to -1.74) |
| Southeast Asia | 303.23 (249.39 to 360) | 535.68 (440.76 to 635.24) | 127.44 (104.00 to 152.10) | 85.92 (70.99 to 101.27) | -1.36 (-1.46 to -1.26) | 9274.52 (7674.58 to 11092.59) | 15932.99 (13150.2 to 18840.79) | 3364.75 (2764.15 to 4011.42) | 2294.14 (1898.44 to 2710.66) | -1.30 (-1.37 to -1.24) |
| Southern Latin America | 38.87 (33.18 to 44.78) | 35.43 (29.14 to 41.91) | 83.72 (71.32 to 96.92) | 40.91 (33.76 to 48.33) | -2.09 (-2.19 to -1.98) | 1265.01 (1052.81 to 1471.44) | 1139.88 (907.61 to 1378.21) | 2696.65 (2241.90 to 3135.60) | 1373.64 (1089.27 to 1661.92) | -2.06 (-2.13 to -1.99) |
| Southern Sub-Saharan Africa | 27.76 (22.4 to 33.6) | 36.73 (29.33 to 44.54) | 101.97 (81.85 to 124.13) | 62.24 (49.81 to 75.96) | -1.65 (-2.04 to -1.26) | 954.76 (764.78 to 1157.89) | 1234.89 (983.44 to 1501.58) | 3088.09 (2481.13 to 3741.47) | 1885.04 (1499.09 to 2282.26) | -1.59 (-1.95 to -1.23) |
| Tropical Latin America | 106.92 (89.85 to 123.34) | 120.79 (97.53 to 146.17) | 122.04 (101.55 to 143.21) | 47.21 (38.07 to 57.18) | -3.23 (-3.35 to -3.12) | 3384.68 (2805.16 to 3933.74) | 3540.52 (2794.87 to 4322.22) | 3408.54 (2824.70 to 3983.04) | 1349.80 (1065.08 to 1649.69) | -3.23 (-3.34 to -3.12) |
| Western Europe | 616.26 (525.82 to 707.51) | 425.44 (351.41 to 501.67) | 106.10 (90.85 to 121.48) | 44.28 (36.87 to 51.75) | -2.83 (-2.89 to -2.77) | 15811.36 (13219.98 to 18509.86) | 10788.92 (8671.98 to 12985.71) | 2915.19 (2429.29 to 3420.58) | 1339.80 (1066.53 to 1616.70) | -2.49 (-2.53 to -2.45) |
| Western Sub-Saharan Africa | 25.64 (19.28 to 32.43) | 38.3 (28.26 to 48.3) | 29.72 (22.47 to 37.83) | 19.41 (14.44 to 24.59) | -1.42 (-1.56 to -1.29) | 870.16 (654.13 to 1114.29) | 1360.16 (1004.9 to 1722.87) | 879.40 (663.17 to 1121.05) | 577.30 (429.83 to 730.62) | -1.44 (-1.60 to -1.28) |

ASDR, age-standardized Deaths rates; ASDALYR, age-standardized disability-adjusted life years rates; CI, confidence interval; DALYs, disability-adjusted life years rates; EAPC, estimated annual percentage change; UI, uncertainty interval.

Table S4 Deaths and DALYs of smoking in 1990 and 2021 at national levels

| Location | Deaths (95% UI) | | ASDR per 100,000 (95% UI) | | 1990-2021 EAPC,  (95% CI) | DALYs (95% UI) | | ASDALYR per 100,000 (95% UI) | | 1990-2021 EAPC,  (95% CI) |
| --- | --- | --- | --- | --- | --- | --- | --- | --- | --- | --- |
|  | 1990 | 2021 | 1990 | 2021 |  | 1990 | 2021 | 1990 | 2021 |  |
| Afghanistan | 4904.42 (3386.92 to 6703.08) | 6285.56 (4456.69 to 8507.37) | 73.08 (51.77 to 99.03) | 60.68 (43.60 to 80.14) | -0.63 (-1.01 to -0.25) | 145286.71 (100119.69 to 198986.04) | 232076.85 (166193.75 to 314137.88) | 2013.61 (1386.17 to 2762.85) | 1763.40 (1284.61 to 2319.58) | -0.50 (-0.87 to -0.13) |
| Albania | 2523.93 (2092.74 to 2940.97) | 4057.05 (3232.47 to 5016.96) | 137.51 (113.46 to 161.19) | 94.68 (75.28 to 117.38) | -0.95 (-1.22 to -0.69) | 67841.76 (55716.93 to 79636.59) | 97812.88 (77487.53 to 119602.52) | 3257.44 (2674.34 to 3843.94) | 2334.73 (1847.56 to 2850.17) | -0.80 (-1.03 to -0.57) |
| Algeria | 9761.52 (7680.75 to 12063.32) | 16777.76 (12524.15 to 22125.04) | 109.53 (85.68 to 137.18) | 61.50 (45.48 to 80.98) | -1.86 (-2.02 to -1.69) | 282648.77 (225692.62 to 345679.09) | 491528.91 (375471.53 to 629609.98) | 2412.27 (1900.99 to 2960.83) | 1403.12 (1068.08 to 1801.70) | -1.89 (-2.02 to -1.76) |
| American Samoa | 25.41 (20.36 to 31.03) | 43.00 (33.71 to 54.20) | 113.34 (89.50 to 136.63) | 88.46 (69.72 to 111.50) | -0.92 (-1.00 to -0.83) | 896.47 (722.05 to 1101.08) | 1500.02 (1177.56 to 1880.29) | 3339.04 (2682.38 to 4017.76) | 2853.98 (2241.71 to 3570.86) | -0.59 (-0.66 to -0.52) |
| Andorra | 51.28 (37.73 to 69.75) | 66.61 (44.75 to 90.73) | 91.52 (67.32 to 123.99) | 42.01 (28.11 to 57.24) | -2.33 (-2.61 to -2.05) | 1488.19 (1093.64 to 1959.03) | 1921.18 (1311.53 to 2573.86) | 2519.15 (1848.70 to 3327.32) | 1287.20 (884.78 to 1726.39) | -2.04 (-2.24 to -1.84) |
| Angola | 3713.64 (2698.52 to 4835.07) | 6882.93 (4877.90 to 9093.27) | 91.09 (66.59 to 118.28) | 56.34 (40.54 to 74.59) | -1.61 (-1.80 to -1.42) | 131018.26 (94768.21 to 170770.05) | 248541.40 (176582.73 to 329557.82) | 2720.18 (1979.83 to 3529.49) | 1682.18 (1208.02 to 2211.69) | -1.59 (-1.77 to -1.40) |
| Antigua and Barbuda | 20.50 (15.93 to 24.99) | 25.97 (20.34 to 32.14) | 38.55 (30.02 to 46.97) | 24.68 (19.35 to 30.64) | -1.69 (-1.94 to -1.45) | 553.28 (428.65 to 687.78) | 783.89 (596.65 to 971.56) | 1098.35 (850.12 to 1367.04) | 704.02 (534.57 to 870.15) | -1.63 (-1.85 to -1.42) |
| Argentina | 29237.84 (24759.11 to 33665.69) | 26477.40 (21579.50 to 31555.41) | 90.20 (76.44 to 104.27) | 47.67 (38.91 to 56.75) | -1.78 (-1.91 to -1.65) | 942142.41 (784087.07 to 1097408.36) | 826099.60 (654473.13 to 1002290.79) | 2896.05 (2411.17 to 3376.76) | 1543.59 (1222.04 to 1872.52) | -1.88 (-1.97 to -1.78) |
| Armenia | 3656.25 (3214.29 to 4088.04) | 4191.93 (3566.37 to 4893.37) | 135.70 (118.12 to 152.71) | 96.23 (81.86 to 112.46) | -1.32 (-1.49 to -1.15) | 110688.80 (96721.58 to 124371.37) | 111646.26 (94991.15 to 129503.43) | 3780.08 (3294.36 to 4245.09) | 2621.95 (2229.53 to 3042.04) | -1.40 (-1.56 to -1.24) |
| Australia | 15837.44 (13257.46 to 18500.83) | 11916.18 (9470.49 to 14678.95) | 80.00 (66.96 to 93.58) | 25.99 (20.88 to 31.76) | -3.68 (-3.79 to -3.58) | 452674.37 (364888.81 to 540826.64) | 345651.88 (265214.19 to 438547.31) | 2332.05 (1877.43 to 2784.62) | 874.31 (664.69 to 1113.64) | -3.17 (-3.26 to -3.08) |
| Austria | 8820.34 (7447.43 to 10303.30) | 7898.15 (6399.66 to 9562.08) | 75.99 (64.44 to 87.97) | 43.41 (35.63 to 51.56) | -1.77 (-1.86 to -1.68) | 242652.23 (201524.09 to 287800.59) | 217241.88 (172347.42 to 262034.00) | 2269.88 (1883.88 to 2691.98) | 1368.88 (1083.55 to 1658.77) | -1.60 (-1.68 to -1.52) |
| Azerbaijan | 5873.45 (4892.72 to 6811.41) | 8658.24 (7061.97 to 10236.55) | 115.45 (95.88 to 134.31) | 89.98 (73.68 to 107.50) | -0.63 (-0.92 to -0.34) | 187395.59 (157993.91 to 217717.11) | 263652.69 (214412.61 to 309735.37) | 3427.61 (2871.87 to 3982.10) | 2384.23 (1942.89 to 2820.64) | -1.22 (-1.49 to -0.95) |
| Bahamas | 68.00 (53.62 to 83.83) | 117.38 (82.63 to 153.10) | 43.37 (34.00 to 53.91) | 28.79 (20.22 to 37.26) | -1.22 (-1.32 to -1.12) | 2211.01 (1733.47 to 2717.78) | 3808.02 (2685.53 to 4904.56) | 1303.59 (1019.07 to 1605.35) | 866.08 (610.76 to 1114.84) | -1.21 (-1.32 to -1.10) |
| Bahrain | 231.53 (187.35 to 281.41) | 469.43 (371.60 to 592.46) | 158.65 (125.86 to 195.68) | 69.38 (53.91 to 90.02) | -3.26 (-3.59 to -2.92) | 7425.09 (5976.71 to 9126.95) | 17585.57 (13721.13 to 22491.76) | 3749.91 (2984.99 to 4618.35) | 1678.14 (1310.66 to 2147.15) | -3.18 (-3.43 to -2.94) |
| Bangladesh | 69786.92 (56555.62 to 85813.64) | 104021.40 (77592.89 to 133520.06) | 154.45 (124.58 to 189.44) | 81.22 (60.99 to 103.13) | -2.14 (-2.35 to -1.93) | 2106166.19 (1715198.38 to 2575426.74) | 2954639.78 (2227401.08 to 3750026.68) | 4167.53 (3393.89 to 5106.60) | 2094.01 (1579.56 to 2661.04) | -2.16 (-2.28 to -2.05) |
| Barbados | 103.05 (80.00 to 127.71) | 104.17 (73.07 to 139.48) | 34.59 (27.14 to 42.74) | 20.12 (14.09 to 26.88) | -2.18 (-2.43 to -1.92) | 2528.49 (1968.45 to 3118.52) | 2620.97 (1845.10 to 3486.51) | 928.77 (723.95 to 1146.17) | 535.77 (377.15 to 711.76) | -2.12 (-2.32 to -1.91) |
| Belarus | 16825.57 (14504.78 to 19170.23) | 17307.78 (13394.76 to 21324.43) | 130.73 (112.41 to 149.47) | 108.36 (84.22 to 133.24) | -1.16 (-1.53 to -0.80) | 497142.32 (422685.78 to 567957.78) | 492788.69 (387848.94 to 604057.82) | 3879.47 (3298.52 to 4454.56) | 3226.96 (2540.58 to 3951.49) | -1.20 (-1.59 to -0.82) |
| Belgium | 19085.27 (16129.77 to 22052.26) | 11872.85 (9587.81 to 14240.98) | 123.38 (104.72 to 142.07) | 49.61 (40.97 to 58.65) | -2.90 (-3.00 to -2.80) | 478660.61 (401386.22 to 556057.69) | 301582.17 (242826.29 to 364597.52) | 3300.21 (2772.24 to 3834.29) | 1476.35 (1187.02 to 1784.75) | -2.56 (-2.64 to -2.47) |
| Belize | 37.98 (30.95 to 45.25) | 89.98 (70.23 to 113.28) | 41.08 (33.44 to 48.92) | 31.04 (23.93 to 39.19) | -1.14 (-1.56 to -0.72) | 1122.15 (898.69 to 1335.06) | 2880.58 (2236.68 to 3662.85) | 1177.42 (944.70 to 1397.40) | 889.55 (690.70 to 1130.23) | -1.10 (-1.44 to -0.75) |
| Benin | 752.85 (556.80 to 986.73) | 877.10 (611.35 to 1183.26) | 37.92 (27.87 to 49.65) | 16.98 (11.84 to 22.84) | -2.74 (-2.89 to -2.60) | 23742.05 (17441.13 to 31317.99) | 30220.27 (21152.52 to 41392.81) | 1106.11 (815.44 to 1459.44) | 510.09 (360.12 to 688.59) | -2.68 (-2.84 to -2.53) |
| Bermuda | 44.81 (35.89 to 54.57) | 44.69 (34.44 to 57.84) | 72.55 (57.69 to 88.83) | 32.24 (24.85 to 41.63) | -2.45 (-2.66 to -2.23) | 1229.45 (976.25 to 1498.02) | 1148.10 (881.21 to 1468.81) | 1922.14 (1527.51 to 2335.84) | 923.59 (704.16 to 1183.81) | -2.23 (-2.45 to -2.01) |
| Bhutan | 140.26 (93.58 to 206.21) | 250.61 (172.40 to 350.35) | 66.16 (44.22 to 97.24) | 44.62 (30.70 to 62.30) | -1.32 (-1.44 to -1.19) | 4521.31 (2983.98 to 6575.82) | 6348.04 (4382.50 to 8942.34) | 1711.21 (1157.92 to 2477.70) | 1036.66 (719.28 to 1449.60) | -1.69 (-1.83 to -1.56) |
| Bolivia (Plurinational State of) | 1881.70 (1421.03 to 2417.51) | 2521.49 (1861.09 to 3584.70) | 60.15 (45.23 to 77.56) | 29.13 (21.76 to 41.09) | -1.97 (-2.28 to -1.66) | 58373.28 (44682.95 to 74937.08) | 75405.28 (56337.64 to 104323.22) | 1667.98 (1268.95 to 2142.74) | 789.97 (594.60 to 1089.82) | -2.11 (-2.44 to -1.79) |
| Bosnia and Herzegovina | 4886.00 (4191.16 to 5597.57) | 5829.03 (4455.23 to 7290.84) | 122.16 (103.71 to 141.59) | 92.63 (70.59 to 115.96) | -0.97 (-1.11 to -0.83) | 157093.85 (133185.96 to 182062.92) | 161291.94 (124512.15 to 199223.81) | 3545.04 (2990.18 to 4122.29) | 2756.47 (2111.88 to 3410.04) | -0.84 (-0.98 to -0.70) |
| Botswana | 786.74 (568.09 to 1072.03) | 985.22 (716.70 to 1332.64) | 143.82 (104.38 to 194.05) | 68.96 (51.53 to 92.30) | -2.62 (-2.91 to -2.33) | 25340.97 (18045.29 to 34895.02) | 32551.02 (23532.50 to 43314.02) | 4017.97 (2899.06 to 5479.07) | 1928.53 (1435.73 to 2561.64) | -2.70 (-3.03 to -2.37) |
| Brazil | 105147.09 (88335.50 to 121277.55) | 117324.43 (94510.03 to 142015.36) | 122.91 (101.99 to 144.30) | 46.89 (37.74 to 56.89) | -3.29 (-3.40 to -3.17) | 3335510.53 (2759093.43 to 3872335.02) | 3445800.94 (2720817.70 to 4205260.87) | 3438.29 (2843.26 to 4019.40) | 1344.53 (1061.51 to 1642.88) | -3.28 (-3.39 to -3.17) |
| Brunei Darussalam | 147.66 (119.46 to 176.90) | 185.61 (143.91 to 231.41) | 154.24 (123.86 to 186.54) | 58.49 (44.60 to 72.52) | -2.72 (-3.01 to -2.44) | 4587.11 (3696.34 to 5513.63) | 6521.64 (5005.09 to 8288.11) | 3939.80 (3162.25 to 4745.18) | 1630.73 (1242.49 to 2059.12) | -2.66 (-2.95 to -2.37) |
| Bulgaria | 17740.20 (15009.64 to 20368.76) | 11889.28 (9556.01 to 14410.93) | 149.12 (126.93 to 171.45) | 91.26 (73.33 to 110.16) | -1.90 (-2.09 to -1.72) | 565670.70 (477029.69 to 649013.30) | 370936.22 (297613.93 to 447503.77) | 4715.85 (3983.85 to 5422.28) | 3167.41 (2554.63 to 3827.23) | -1.61 (-1.77 to -1.44) |
| Burkina Faso | 1115.91 (799.88 to 1506.81) | 1774.45 (1251.44 to 2405.93) | 24.89 (17.97 to 33.27) | 17.91 (12.79 to 24.16) | -0.97 (-1.16 to -0.78) | 40298.62 (29158.21 to 54556.09) | 67137.73 (47149.08 to 90886.26) | 815.90 (591.36 to 1101.45) | 590.25 (420.35 to 799.53) | -0.99 (-1.18 to -0.80) |
| Burundi | 2199.05 (1648.24 to 2863.56) | 1624.48 (1171.63 to 2136.16) | 92.69 (69.84 to 120.60) | 31.69 (22.71 to 41.59) | -3.75 (-4.26 to -3.24) | 73455.33 (54653.56 to 96650.85) | 59315.54 (42742.67 to 79303.35) | 2768.45 (2066.29 to 3640.09) | 965.87 (696.10 to 1289.33) | -3.66 (-4.16 to -3.17) |
| Côte d'Ivoire | 65.93 (48.68 to 85.94) | 99.21 (72.66 to 131.78) | 29.72 (21.98 to 38.66) | 21.96 (16.21 to 28.88) | -2.25 (-2.66 to -1.85) | 1976.78 (1485.21 to 2571.96) | 3194.30 (2286.77 to 4263.78) | 938.84 (704.03 to 1223.03) | 656.17 (474.66 to 868.01) | -2.17 (-2.58 to -1.76) |
| Cabo Verde | 7762.29 (6273.70 to 9425.77) | 14428.27 (10746.96 to 17917.98) | 186.36 (150.24 to 225.41) | 131.07 (97.42 to 163.04) | -1.51 (-1.99 to -1.03) | 237815.65 (192031.05 to 291268.74) | 415462.08 (310065.30 to 522001.23) | 4890.82 (3961.07 to 5955.47) | 3227.97 (2437.31 to 4030.53) | -1.54 (-1.87 to -1.22) |
| Cambodia | 1751.57 (1281.51 to 2298.35) | 3266.82 (2084.59 to 4588.33) | 37.98 (27.85 to 49.31) | 24.59 (16.04 to 34.49) | -1.32 (-1.47 to -1.16) | 63021.70 (45859.59 to 82655.23) | 120671.60 (77862.31 to 166110.97) | 1189.62 (875.99 to 1558.30) | 765.26 (496.81 to 1054.88) | -1.60 (-1.77 to -1.43) |
| Cameroon | 36913.81 (31599.87 to 42676.95) | 32295.06 (26183.09 to 38537.14) | 113.57 (97.27 to 131.49) | 42.73 (35.08 to 50.71) | -1.41 (-1.77 to -1.05) | 977247.31 (822524.48 to 1139996.51) | 816448.78 (652468.26 to 990088.92) | 3048.97 (2566.02 to 3553.36) | 1224.99 (968.04 to 1492.43) | -1.49 (-1.87 to -1.10) |
| Canada | 1199.67 (878.99 to 1607.50) | 1548.53 (1011.50 to 2168.20) | 96.14 (70.94 to 126.66) | 59.93 (41.46 to 81.82) | -3.23 (-3.31 to -3.15) | 42210.87 (30811.86 to 57480.53) | 58397.38 (37965.50 to 82164.73) | 2969.65 (2162.39 to 3990.55) | 1912.68 (1293.98 to 2632.30) | -3.03 (-3.10 to -2.96) |
| Central African Republic | 1217.75 (851.23 to 1672.54) | 2001.78 (1324.12 to 2836.00) | 44.02 (30.69 to 60.57) | 35.36 (23.55 to 51.04) | -1.66 (-1.83 to -1.50) | 38235.17 (27048.17 to 52218.05) | 68235.84 (45266.98 to 96652.97) | 1267.10 (898.70 to 1732.62) | 1006.43 (671.72 to 1419.40) | -1.57 (-1.73 to -1.41) |
| Chad | 5703.59 (4747.56 to 6793.60) | 5523.88 (4485.41 to 6610.51) | 55.96 (46.44 to 66.88) | 21.76 (17.74 to 26.03) | -0.97 (-1.22 to -0.73) | 207615.69 (165793.39 to 251872.62) | 218371.35 (166453.05 to 272634.96) | 1919.44 (1533.77 to 2321.64) | 904.28 (685.93 to 1132.86) | -1.00 (-1.27 to -0.73) |
| Chile | 1462998.63 (1200178.60 to 1760241.47) | 2304209.18 (1814317.23 to 2896304.86) | 217.27 (178.48 to 260.26) | 116.69 (91.86 to 145.56) | -2.90 (-2.95 to -2.84) | 39159330.36 (32147078.46 to 46908740.74) | 54701548.52 (43108562.78 to 68748853.71) | 4782.06 (3924.43 to 5752.16) | 2616.41 (2061.62 to 3293.19) | -2.33 (-2.39 to -2.28) |
| China | 9187.89 (7654.47 to 10966.65) | 10772.32 (8076.45 to 14269.77) | 53.21 (44.15 to 63.56) | 19.35 (14.53 to 25.54) | -2.11 (-2.21 to -2.01) | 298994.19 (243362.06 to 363149.34) | 320592.89 (240138.30 to 422947.32) | 1545.99 (1259.42 to 1877.79) | 580.01 (433.62 to 766.04) | -2.00 (-2.07 to -1.94) |
| Colombia | 140.68 (94.91 to 192.97) | 176.49 (122.91 to 236.53) | 77.25 (53.79 to 103.97) | 39.44 (27.79 to 52.91) | -3.79 (-3.98 to -3.61) | 4407.79 (2958.55 to 6074.27) | 5408.71 (3741.80 to 7222.94) | 2053.31 (1396.28 to 2794.81) | 1032.72 (720.47 to 1379.42) | -3.61 (-3.77 to -3.44) |
| Comoros | 746.95 (557.40 to 989.64) | 1317.73 (952.22 to 1746.68) | 69.26 (52.19 to 90.97) | 49.67 (36.90 to 64.80) | -2.55 (-2.80 to -2.31) | 24167.40 (17636.66 to 32254.58) | 45493.67 (32314.79 to 61034.40) | 2000.59 (1478.23 to 2656.17) | 1398.45 (1017.90 to 1824.61) | -2.63 (-2.92 to -2.35) |
| Congo | 13.07 (10.05 to 16.06) | 15.34 (11.83 to 19.67) | 106.38 (81.50 to 130.71) | 60.30 (46.29 to 77.56) | -1.15 (-1.41 to -0.90) | 422.06 (323.57 to 525.01) | 471.25 (364.05 to 610.62) | 3109.32 (2379.52 to 3848.41) | 1928.19 (1480.27 to 2508.45) | -1.26 (-1.52 to -1.00) |
| Cook Islands | 904.25 (731.58 to 1081.22) | 1371.16 (1045.18 to 1720.82) | 53.55 (43.10 to 64.35) | 24.79 (18.94 to 31.10) | -1.96 (-2.08 to -1.84) | 25919.76 (20459.35 to 31841.57) | 40357.71 (30483.32 to 51412.71) | 1423.74 (1124.95 to 1740.89) | 731.47 (552.80 to 932.60) | -1.61 (-1.72 to -1.50) |
| Costa Rica | 2127.84 (1535.99 to 2821.03) | 3581.47 (2458.24 to 4961.25) | 52.11 (38.06 to 69.19) | 31.15 (21.62 to 42.32) | -2.73 (-2.95 to -2.51) | 78927.00 (57054.53 to 104331.67) | 134136.28 (90486.31 to 183692.43) | 1532.80 (1107.72 to 2008.62) | 938.04 (643.30 to 1273.00) | -2.42 (-2.57 to -2.27) |
| Croatia | 9454.09 (8023.07 to 10961.88) | 7537.69 (6100.57 to 9023.39) | 162.56 (136.73 to 190.53) | 82.13 (66.84 to 97.87) | -1.99 (-2.09 to -1.89) | 268045.41 (226302.49 to 310885.55) | 186925.99 (148957.16 to 225257.40) | 4350.86 (3655.89 to 5071.26) | 2335.14 (1860.14 to 2821.11) | -1.84 (-1.91 to -1.76) |
| Cuba | 10549.83 (8889.73 to 12318.62) | 13319.21 (10614.98 to 16157.38) | 104.98 (88.28 to 123.12) | 67.19 (53.66 to 81.20) | -1.57 (-1.68 to -1.45) | 289684.94 (243612.18 to 338881.11) | 351287.82 (283355.90 to 424433.15) | 2843.20 (2391.50 to 3326.85) | 1851.43 (1491.50 to 2238.00) | -1.53 (-1.64 to -1.42) |
| Cyprus | 791.45 (651.91 to 928.61) | 1065.31 (853.94 to 1293.02) | 121.45 (98.56 to 146.37) | 52.28 (41.89 to 63.77) | -2.72 (-2.80 to -2.64) | 21212.27 (17323.69 to 25184.83) | 30016.07 (23651.64 to 37366.45) | 2803.95 (2276.64 to 3357.88) | 1506.65 (1179.65 to 1872.74) | -1.95 (-2.01 to -1.90) |
| Czechia | 21363.84 (18085.90 to 24903.73) | 13823.20 (11186.85 to 16684.43) | 155.30 (131.61 to 181.07) | 63.56 (51.44 to 76.76) | -2.80 (-2.88 to -2.72) | 613116.14 (509638.08 to 709239.10) | 382931.43 (303505.42 to 468303.59) | 4585.43 (3828.17 to 5313.35) | 1985.23 (1566.66 to 2444.15) | -2.64 (-2.72 to -2.55) |
| Democratic People's Republic of Korea | 20297.43 (15038.44 to 25890.45) | 34351.80 (25486.74 to 42925.96) | 136.23 (101.40 to 171.51) | 105.52 (78.92 to 132.42) | -0.90 (-1.00 to -0.79) | 624752.72 (468051.42 to 799431.24) | 1021486.21 (768333.10 to 1276793.98) | 3603.59 (2711.47 to 4549.41) | 3004.36 (2262.11 to 3759.00) | -0.69 (-0.82 to -0.56) |
| Democratic Republic of the Congo | 6927.80 (5083.07 to 9101.72) | 11143.33 (7855.44 to 15233.07) | 42.73 (31.53 to 55.84) | 27.50 (19.58 to 37.17) | -1.38 (-1.55 to -1.21) | 243277.11 (176188.47 to 321538.31) | 419788.90 (300260.89 to 570107.91) | 1300.19 (949.58 to 1708.09) | 883.43 (632.91 to 1187.66) | -1.19 (-1.32 to -1.06) |
| Denmark | 14876.65 (12902.53 to 17020.58) | 9075.88 (7376.22 to 10778.93) | 180.28 (156.69 to 205.07) | 71.00 (58.01 to 83.77) | -3.27 (-3.39 to -3.15) | 353372.18 (300662.11 to 409724.37) | 204607.02 (163041.11 to 245354.99) | 4659.57 (3959.08 to 5401.88) | 1838.67 (1452.89 to 2230.72) | -3.26 (-3.37 to -3.15) |
| Djibouti | 122.30 (86.36 to 173.52) | 393.14 (263.60 to 549.01) | 91.33 (65.28 to 126.38) | 66.21 (45.43 to 90.98) | -1.10 (-1.17 to -1.02) | 4460.21 (3141.12 to 6324.27) | 13666.17 (9064.61 to 18905.40) | 2559.46 (1831.29 to 3567.32) | 1779.46 (1203.99 to 2456.67) | -1.25 (-1.36 to -1.14) |
| Dominica | 25.33 (20.12 to 30.75) | 26.26 (19.94 to 34.45) | 42.86 (34.06 to 52.07) | 31.18 (23.77 to 40.97) | -1.02 (-1.29 to -0.75) | 673.26 (532.02 to 827.38) | 768.71 (585.55 to 1013.01) | 1168.31 (921.38 to 1433.02) | 897.78 (685.53 to 1183.75) | -0.80 (-1.05 to -0.54) |
| Dominican Republic | 2430.75 (1881.85 to 3055.03) | 5729.65 (4312.72 to 7615.03) | 73.17 (56.62 to 92.81) | 58.16 (43.74 to 77.51) | -0.28 (-0.55 to -0.01) | 71010.34 (54604.83 to 88604.52) | 158152.15 (119854.92 to 210023.03) | 1831.97 (1409.59 to 2286.34) | 1551.16 (1177.45 to 2060.82) | -0.19 (-0.38 to -0.01) |
| Ecuador | 2406.39 (1945.75 to 2953.60) | 3028.07 (2193.56 to 3957.04) | 47.52 (38.33 to 58.70) | 19.15 (13.87 to 25.09) | -2.69 (-2.89 to -2.49) | 71992.05 (57983.75 to 88860.45) | 89878.31 (65361.77 to 115507.64) | 1268.67 (1017.52 to 1561.57) | 540.99 (393.92 to 694.99) | -2.59 (-2.78 to -2.40) |
| Egypt | 29405.80 (24336.40 to 34805.29) | 61815.98 (48173.75 to 77148.79) | 120.51 (97.75 to 145.42) | 110.51 (84.97 to 137.97) | 0.03 (-0.16 to 0.22) | 939853.51 (775017.67 to 1109649.38) | 2000226.74 (1555165.39 to 2475668.79) | 3116.20 (2550.29 to 3705.36) | 2858.82 (2231.95 to 3540.16) | -0.02 (-0.18 to 0.15) |
| El Salvador | 724.33 (556.46 to 913.13) | 1224.60 (885.63 to 1615.26) | 24.10 (18.42 to 30.58) | 19.84 (14.34 to 26.14) | -0.66 (-0.85 to -0.46) | 24345.37 (18405.47 to 30743.81) | 39741.13 (28750.30 to 52057.27) | 761.38 (577.10 to 963.18) | 657.07 (475.79 to 860.98) | -0.44 (-0.64 to -0.25) |
| Equatorial Guinea | 155.49 (108.15 to 213.45) | 174.60 (111.69 to 246.42) | 75.70 (52.82 to 103.83) | 33.60 (22.04 to 46.19) | -2.92 (-3.50 to -2.34) | 5335.38 (3686.95 to 7254.87) | 6411.74 (4124.26 to 9073.33) | 2332.57 (1610.70 to 3178.16) | 990.53 (644.06 to 1373.44) | -3.09 (-3.69 to -2.49) |
| Eritrea | 885.33 (593.86 to 1267.00) | 1051.04 (687.89 to 1504.49) | 59.70 (40.89 to 86.53) | 30.78 (20.40 to 43.74) | -2.47 (-2.61 to -2.32) | 36099.39 (24248.43 to 51594.62) | 43045.65 (28104.64 to 62063.90) | 2072.34 (1415.80 to 2967.53) | 1044.55 (698.09 to 1484.51) | -2.56 (-2.70 to -2.42) |
| Estonia | 2384.52 (2053.23 to 2741.58) | 1202.77 (979.38 to 1426.30) | 116.83 (100.37 to 134.27) | 46.81 (38.33 to 55.25) | -3.47 (-3.83 to -3.11) | 73391.46 (62348.79 to 84488.73) | 34918.75 (28097.70 to 42846.90) | 3665.00 (3108.04 to 4226.65) | 1554.65 (1240.17 to 1918.16) | -3.31 (-3.64 to -2.98) |
| Eswatini | 205.87 (151.53 to 274.04) | 284.00 (191.54 to 393.48) | 77.22 (57.13 to 104.71) | 52.07 (35.56 to 71.20) | -0.91 (-1.46 to -0.35) | 6390.53 (4670.77 to 8543.72) | 9192.63 (6154.94 to 12734.95) | 2031.07 (1485.57 to 2695.46) | 1425.42 (968.26 to 1950.14) | -0.82 (-1.43 to -0.21) |
| Ethiopia | 9681.62 (7298.91 to 12600.94) | 5932.81 (4249.93 to 7958.91) | 44.51 (33.67 to 58.40) | 13.75 (9.77 to 18.51) | -3.80 (-4.13 to -3.46) | 336335.83 (252000.94 to 441098.02) | 205683.45 (149895.48 to 277142.96) | 1386.50 (1045.08 to 1810.03) | 405.71 (297.30 to 546.92) | -4.02 (-4.34 to -3.69) |
| Fiji | 524.70 (403.99 to 655.08) | 715.59 (515.66 to 972.46) | 136.13 (104.38 to 170.72) | 87.92 (64.57 to 119.07) | -1.66 (-1.81 to -1.51) | 18806.69 (14621.01 to 23316.45) | 24764.36 (18273.60 to 33344.65) | 4178.45 (3205.15 to 5215.62) | 2788.34 (2060.61 to 3742.46) | -1.49 (-1.61 to -1.36) |
| Finland | 5506.78 (4620.80 to 6480.87) | 3854.34 (3092.81 to 4679.06) | 78.24 (65.98 to 91.70) | 30.46 (24.63 to 36.53) | -2.95 (-2.99 to -2.90) | 160594.64 (132931.21 to 190117.04) | 104080.33 (81742.45 to 128697.79) | 2390.55 (1979.65 to 2832.52) | 1007.15 (785.60 to 1252.15) | -2.66 (-2.71 to -2.61) |
| France | 64925.70 (54968.66 to 75740.90) | 52331.65 (42227.23 to 62095.66) | 79.00 (67.31 to 91.51) | 38.06 (31.43 to 44.26) | -2.25 (-2.30 to -2.20) | 1731778.49 (1426296.41 to 2046766.82) | 1440754.29 (1148869.40 to 1746526.00) | 2286.59 (1881.36 to 2706.27) | 1274.02 (1013.60 to 1550.60) | -1.75 (-1.79 to -1.71) |
| Gabon | 256.69 (189.61 to 332.33) | 389.64 (266.60 to 519.42) | 44.75 (32.83 to 57.87) | 35.92 (24.88 to 47.27) | -0.72 (-0.80 to -0.65) | 8176.59 (5960.41 to 10718.92) | 13439.20 (9162.02 to 18120.06) | 1352.61 (994.11 to 1758.75) | 1089.25 (747.86 to 1451.13) | -0.71 (-0.79 to -0.63) |
| Gambia | 245.78 (171.96 to 328.43) | 377.00 (256.94 to 511.51) | 67.12 (47.39 to 89.22) | 37.21 (25.54 to 49.82) | -2.14 (-2.31 to -1.97) | 8795.73 (6339.07 to 11827.86) | 13300.43 (9084.69 to 17763.93) | 2071.30 (1483.78 to 2756.92) | 1136.66 (784.29 to 1515.45) | -2.20 (-2.38 to -2.02) |
| Georgia | 7015.32 (5993.73 to 7964.91) | 5341.83 (4501.39 to 6293.01) | 111.84 (95.78 to 127.56) | 91.12 (76.93 to 106.85) | -0.46 (-0.66 to -0.26) | 217476.59 (186405.34 to 248010.03) | 148962.32 (125411.98 to 174151.18) | 3441.60 (2951.60 to 3923.27) | 2729.75 (2303.17 to 3183.98) | -0.70 (-0.91 to -0.49) |
| Germany | 132907.00 (111776.98 to 155708.86) | 94195.99 (76715.48 to 112177.97) | 104.36 (88.01 to 121.84) | 48.57 (40.28 to 57.28) | -2.41 (-2.58 to -2.24) | 3520588.67 (2920826.12 to 4171716.37) | 2471534.25 (1974409.76 to 3010235.15) | 2978.07 (2467.54 to 3533.02) | 1516.41 (1207.13 to 1864.56) | -2.10 (-2.22 to -1.98) |
| Ghana | 2011.94 (1487.23 to 2710.49) | 4541.90 (3230.83 to 6102.75) | 34.83 (25.69 to 46.03) | 29.38 (20.59 to 39.01) | 0.09 (-0.11 to 0.29) | 67416.12 (49343.49 to 89084.33) | 151239.44 (107587.51 to 204177.01) | 950.94 (700.17 to 1252.63) | 791.97 (562.53 to 1054.12) | 0.05 (-0.15 to 0.24) |
| Greece | 17911.96 (15559.09 to 20533.64) | 18251.25 (15385.37 to 21437.91) | 119.55 (103.42 to 137.08) | 74.44 (63.81 to 85.41) | -1.54 (-1.63 to -1.46) | 465640.01 (402354.19 to 537670.87) | 437617.45 (362547.79 to 516081.43) | 3200.12 (2756.27 to 3706.76) | 2197.14 (1824.92 to 2578.05) | -1.20 (-1.27 to -1.13) |
| Greenland | 87.14 (73.16 to 101.18) | 80.46 (64.74 to 98.15) | 265.76 (222.40 to 314.54) | 115.88 (92.39 to 141.98) | -2.69 (-2.76 to -2.61) | 2848.73 (2336.45 to 3342.98) | 2475.49 (1983.14 to 2997.61) | 7293.72 (6054.44 to 8521.07) | 3295.88 (2645.35 to 4003.23) | -2.60 (-2.67 to -2.53) |
| Grenada | 32.60 (26.01 to 41.32) | 32.08 (24.47 to 40.06) | 47.53 (38.16 to 59.44) | 27.38 (20.78 to 34.51) | -2.06 (-2.36 to -1.77) | 924.09 (734.10 to 1153.90) | 1023.34 (782.15 to 1291.47) | 1450.87 (1148.96 to 1807.14) | 838.15 (639.71 to 1057.35) | -1.93 (-2.07 to -1.79) |
| Guam | 54.57 (44.82 to 65.54) | 105.67 (84.42 to 130.74) | 68.47 (55.59 to 82.46) | 50.81 (40.68 to 62.77) | -0.56 (-0.79 to -0.33) | 1946.11 (1577.77 to 2372.82) | 3706.30 (2968.37 to 4586.12) | 2091.01 (1689.25 to 2529.27) | 1850.10 (1489.80 to 2292.18) | -0.08 (-0.25 to 0.09) |
| Guatemala | 1446.87 (1118.38 to 1837.03) | 2222.66 (1628.56 to 2909.20) | 47.60 (36.17 to 61.29) | 21.26 (15.46 to 27.81) | -2.97 (-3.16 to -2.79) | 46507.29 (35131.89 to 59165.55) | 69751.91 (51311.75 to 91020.97) | 1221.07 (925.59 to 1570.31) | 604.19 (444.69 to 786.91) | -2.66 (-2.83 to -2.49) |
| Guinea | 1332.20 (959.21 to 1809.79) | 1995.52 (1364.43 to 2722.25) | 41.62 (29.94 to 56.54) | 35.96 (24.73 to 49.56) | -0.28 (-0.45 to -0.11) | 41626.41 (30264.72 to 55628.00) | 65002.36 (44391.11 to 87546.20) | 1189.45 (869.56 to 1596.29) | 1027.42 (707.02 to 1383.01) | -0.29 (-0.49 to -0.09) |
| Guinea-Bissau | 166.22 (115.01 to 226.09) | 266.38 (186.43 to 365.08) | 39.29 (27.08 to 53.10) | 32.48 (23.06 to 44.19) | -0.11 (-0.38 to 0.15) | 5776.09 (4027.57 to 7873.45) | 9985.35 (6850.19 to 14054.00) | 1223.17 (851.90 to 1667.21) | 1024.80 (717.16 to 1408.10) | -0.07 (-0.33 to 0.18) |
| Guyana | 269.13 (214.61 to 337.61) | 251.41 (174.15 to 348.29) | 67.88 (53.80 to 84.92) | 37.97 (26.62 to 52.30) | -1.52 (-1.62 to -1.42) | 9173.68 (7252.11 to 11498.30) | 8782.26 (6150.11 to 12046.29) | 2096.46 (1669.95 to 2639.33) | 1233.62 (865.85 to 1688.35) | -1.37 (-1.47 to -1.28) |
| Haiti | 1958.61 (1488.88 to 2532.80) | 2271.27 (1540.78 to 3168.38) | 57.80 (43.97 to 73.35) | 31.16 (21.24 to 42.67) | -1.88 (-2.08 to -1.68) | 68250.71 (51493.58 to 87862.18) | 79009.81 (53690.42 to 109054.58) | 1804.93 (1367.81 to 2321.49) | 916.95 (627.45 to 1268.06) | -2.12 (-2.34 to -1.90) |
| Honduras | 1061.74 (823.02 to 1326.15) | 3344.09 (2598.88 to 4242.84) | 53.94 (41.39 to 67.41) | 57.08 (43.85 to 71.82) | 0.52 (0.32 to 0.73) | 33814.83 (26374.58 to 42389.55) | 95944.57 (73104.18 to 124374.72) | 1507.84 (1169.61 to 1890.46) | 1455.24 (1120.96 to 1875.20) | 0.09 (-0.06 to 0.25) |
| Hungary | 22326.98 (19362.74 to 25606.62) | 15320.39 (12615.73 to 18392.25) | 154.84 (134.32 to 176.86) | 82.44 (68.23 to 98.35) | -2.15 (-2.25 to -2.05) | 697729.29 (596709.99 to 799614.71) | 451628.83 (365744.61 to 545174.44) | 4994.46 (4265.17 to 5729.70) | 2692.71 (2170.35 to 3244.06) | -2.17 (-2.28 to -2.06) |
| Iceland | 298.02 (245.32 to 359.52) | 243.90 (191.80 to 304.89) | 102.61 (84.98 to 122.87) | 40.42 (32.09 to 49.71) | -3.07 (-3.14 to -2.99) | 7687.04 (6262.29 to 9239.45) | 6440.97 (5031.91 to 8081.92) | 2790.22 (2271.86 to 3345.15) | 1201.95 (935.05 to 1513.89) | -2.83 (-2.89 to -2.77) |
| India | 515814.21 (408670.02 to 633963.42) | 836056.50 (657237.99 to 1028420.62) | 121.88 (93.88 to 150.92) | 76.76 (60.13 to 94.92) | -1.38 (-1.50 to -1.25) | 16252735.43 (12851692.05 to 19913679.43) | 22540404.09 (17566203.71 to 27529015.49) | 3228.30 (2550.27 to 3967.30) | 1863.30 (1460.42 to 2283.96) | -1.74 (-1.82 to -1.67) |
| Indonesia | 92940.78 (73966.07 to 113283.06) | 223481.13 (169965.17 to 283927.38) | 100.28 (78.68 to 123.35) | 102.80 (78.25 to 129.53) | 0.18 (-0.01 to 0.37) | 3052189.75 (2422856.77 to 3673728.84) | 6897785.55 (5276084.15 to 8739278.55) | 2758.44 (2193.16 to 3345.66) | 2659.14 (2056.66 to 3344.52) | -0.01 (-0.17 to 0.14) |
| Iran (Islamic Republic of) | 15611.57 (12678.93 to 18595.34) | 28859.88 (23744.85 to 34397.41) | 61.19 (49.08 to 73.73) | 38.12 (30.80 to 45.71) | -1.34 (-1.43 to -1.25) | 530043.71 (425532.42 to 642122.88) | 963115.33 (773107.41 to 1162920.79) | 1786.89 (1431.10 to 2168.97) | 1122.98 (898.18 to 1356.33) | -1.33 (-1.41 to -1.25) |
| Iraq | 10433.49 (8319.05 to 12835.45) | 21408.11 (15413.67 to 27370.26) | 134.96 (107.05 to 166.23) | 100.40 (73.44 to 126.63) | -1.48 (-1.67 to -1.30) | 311623.83 (249135.22 to 380081.78) | 663880.35 (493899.70 to 855333.75) | 3716.92 (2962.98 to 4552.09) | 2580.20 (1914.49 to 3286.90) | -1.63 (-1.77 to -1.49) |
| Ireland | 7202.48 (6079.95 to 8354.04) | 3616.76 (2872.97 to 4360.03) | 177.05 (148.86 to 205.84) | 44.01 (35.19 to 52.76) | -4.59 (-4.76 to -4.41) | 167717.59 (138438.82 to 197299.99) | 90982.83 (71861.47 to 111241.69) | 4177.59 (3441.87 to 4919.09) | 1203.23 (942.25 to 1478.87) | -4.12 (-4.25 to -3.99) |
| Israel | 3883.16 (3223.75 to 4622.40) | 3697.85 (3001.50 to 4449.43) | 82.25 (68.05 to 97.54) | 29.26 (23.85 to 34.97) | -3.42 (-3.49 to -3.36) | 106792.95 (87397.70 to 128025.00) | 106245.69 (83414.79 to 131756.70) | 2274.48 (1866.55 to 2720.16) | 926.52 (727.72 to 1146.62) | -2.99 (-3.04 to -2.93) |
| Italy | 82620.03 (70060.98 to 94903.54) | 56647.87 (45722.16 to 67390.54) | 93.15 (79.17 to 106.95) | 36.95 (30.49 to 43.21) | -3.02 (-3.07 to -2.97) | 2185210.97 (1823247.64 to 2551188.94) | 1373749.62 (1092887.93 to 1678594.54) | 2597.97 (2159.26 to 3037.47) | 1104.80 (877.94 to 1354.51) | -2.77 (-2.80 to -2.73) |
| Jamaica | 835.32 (676.44 to 999.77) | 1054.00 (745.33 to 1453.77) | 47.17 (38.21 to 56.31) | 33.96 (24.09 to 46.76) | -1.22 (-1.60 to -0.83) | 23434.01 (18932.17 to 28243.74) | 30158.99 (21674.33 to 40847.54) | 1372.36 (1111.60 to 1646.95) | 979.69 (704.32 to 1324.77) | -1.33 (-1.69 to -0.98) |
| Japan | 126239.88 (107610.68 to 146854.93) | 132466.72 (107263.33 to 158034.05) | 76.13 (64.72 to 88.71) | 33.55 (27.85 to 39.25) | -2.77 (-2.81 to -2.72) | 3533270.84 (2893120.81 to 4200772.72) | 2960653.57 (2378379.30 to 3584619.31) | 2092.73 (1707.35 to 2493.98) | 1031.37 (810.23 to 1248.21) | -2.40 (-2.46 to -2.34) |
| Jordan | 1335.06 (1077.24 to 1637.53) | 3572.34 (2730.49 to 4473.82) | 108.76 (86.22 to 134.18) | 52.67 (40.25 to 65.51) | -2.68 (-2.86 to -2.49) | 44898.23 (36376.23 to 54758.69) | 139697.72 (109007.46 to 174152.61) | 2967.67 (2399.11 to 3624.87) | 1611.46 (1252.35 to 2007.56) | -2.26 (-2.41 to -2.12) |
| Kazakhstan | 15596.04 (13390.46 to 17992.38) | 12415.26 (10168.66 to 14888.58) | 118.62 (101.33 to 137.86) | 68.16 (55.50 to 81.45) | -2.47 (-2.97 to -1.98) | 519132.13 (449693.40 to 601443.53) | 404160.21 (327794.21 to 485913.15) | 3749.33 (3243.30 to 4345.28) | 2061.81 (1667.36 to 2473.15) | -2.70 (-3.24 to -2.15) |
| Kenya | 4197.32 (2963.51 to 5780.36) | 7720.95 (5623.71 to 10379.46) | 53.67 (38.13 to 74.85) | 34.33 (24.80 to 46.12) | -1.60 (-1.81 to -1.39) | 137164.90 (98634.59 to 187985.04) | 264693.90 (194146.32 to 352773.13) | 1476.92 (1074.05 to 2024.19) | 975.88 (717.85 to 1306.47) | -1.46 (-1.72 to -1.20) |
| Kiribati | 92.07 (72.92 to 113.94) | 167.46 (127.42 to 213.48) | 243.82 (192.50 to 300.06) | 223.43 (169.83 to 282.69) | -0.42 (-0.63 to -0.22) | 3192.40 (2534.03 to 3978.46) | 6048.62 (4559.79 to 7755.50) | 7391.74 (5889.96 to 9131.59) | 6888.85 (5254.71 to 8706.16) | -0.35 (-0.54 to -0.16) |
| Kuwait | 438.62 (362.77 to 518.73) | 1262.16 (953.10 to 1605.30) | 69.17 (55.51 to 82.70) | 42.35 (31.30 to 54.73) | -1.39 (-1.84 to -0.93) | 17616.07 (14358.70 to 20943.79) | 52930.59 (40365.15 to 66363.83) | 2066.26 (1666.33 to 2460.91) | 1282.84 (984.07 to 1620.14) | -1.38 (-1.75 to -1.00) |
| Kyrgyzstan | 3727.34 (3132.47 to 4335.71) | 4265.05 (3391.47 to 5254.98) | 127.41 (107.25 to 148.61) | 92.20 (74.72 to 113.36) | -0.81 (-1.15 to -0.47) | 113724.15 (95354.91 to 132575.38) | 136487.00 (109554.32 to 168055.40) | 3670.19 (3074.89 to 4274.37) | 2556.47 (2052.01 to 3153.03) | -1.20 (-1.56 to -0.84) |
| Lao People's Democratic Republic | 4119.51 (3092.88 to 5165.09) | 5280.90 (3881.98 to 6665.95) | 206.85 (156.90 to 257.34) | 126.63 (94.34 to 158.78) | -1.66 (-1.72 to -1.60) | 126226.74 (93885.20 to 158783.55) | 157689.19 (116400.84 to 198419.27) | 5596.06 (4199.30 to 7013.88) | 3178.61 (2353.00 to 3967.62) | -1.91 (-1.97 to -1.85) |
| Latvia | 4298.70 (3710.10 to 4926.42) | 2342.57 (1904.01 to 2785.98) | 120.96 (104.22 to 138.38) | 63.79 (52.22 to 75.43) | -2.48 (-2.78 to -2.18) | 132048.24 (113439.88 to 151800.76) | 67644.57 (55527.79 to 81230.22) | 3795.86 (3256.03 to 4352.16) | 2081.62 (1704.16 to 2509.31) | -2.45 (-2.76 to -2.13) |
| Lebanon | 2787.01 (2175.83 to 3489.15) | 4893.44 (3820.54 to 6084.24) | 134.38 (105.40 to 168.61) | 77.95 (61.08 to 96.66) | -1.47 (-1.63 to -1.32) | 84511.97 (66085.78 to 104737.95) | 132069.05 (103780.19 to 162210.67) | 3691.13 (2872.99 to 4580.34) | 2201.77 (1731.72 to 2707.04) | -1.41 (-1.56 to -1.26) |
| Lesotho | 874.05 (629.39 to 1170.64) | 1814.28 (1323.24 to 2353.69) | 106.76 (77.32 to 144.04) | 164.14 (118.11 to 214.68) | 2.21 (1.79 to 2.64) | 25577.50 (18525.57 to 34208.00) | 60000.08 (44008.50 to 77689.12) | 2865.73 (2076.23 to 3832.75) | 4823.55 (3521.13 to 6217.23) | 2.51 (2.04 to 2.98) |
| Liberia | 356.05 (252.25 to 471.40) | 476.78 (314.05 to 666.71) | 31.35 (22.42 to 41.25) | 20.45 (13.53 to 28.19) | -1.65 (-1.80 to -1.50) | 12001.77 (8497.19 to 15895.61) | 18569.03 (12411.00 to 25763.06) | 964.89 (685.77 to 1273.75) | 660.50 (441.76 to 904.01) | -1.48 (-1.63 to -1.32) |
| Libya | 1321.30 (1038.33 to 1674.84) | 3662.76 (2728.24 to 4758.51) | 72.01 (56.43 to 91.65) | 70.82 (52.68 to 91.34) | 0.40 (0.20 to 0.60) | 41587.71 (32978.94 to 51888.16) | 123688.84 (91873.60 to 158390.13) | 2004.07 (1595.62 to 2500.06) | 1996.75 (1495.38 to 2544.64) | 0.35 (0.19 to 0.51) |
| Lithuania | 5145.62 (4374.63 to 5929.68) | 3578.05 (2930.26 to 4272.19) | 114.99 (97.95 to 132.58) | 64.95 (53.69 to 77.02) | -1.96 (-2.23 to -1.69) | 150301.59 (127366.26 to 172408.28) | 97641.84 (79972.79 to 116977.48) | 3407.65 (2889.65 to 3913.02) | 2032.86 (1664.55 to 2438.58) | -1.78 (-2.06 to -1.50) |
| Luxembourg | 572.34 (470.74 to 682.02) | 454.28 (357.28 to 563.90) | 105.33 (86.84 to 125.42) | 42.10 (33.35 to 52.06) | -2.93 (-3.00 to -2.86) | 15591.34 (12620.64 to 18699.56) | 12682.67 (9711.16 to 15720.63) | 2975.03 (2408.06 to 3563.68) | 1261.87 (963.12 to 1567.56) | -2.84 (-2.90 to -2.77) |
| Madagascar | 3789.06 (2971.45 to 4779.97) | 3513.93 (2375.89 to 5024.48) | 78.10 (60.53 to 100.56) | 31.88 (21.81 to 44.72) | -3.08 (-3.35 to -2.82) | 128608.92 (99072.34 to 161825.63) | 132685.57 (91724.61 to 187953.91) | 2217.97 (1717.61 to 2804.24) | 910.89 (636.35 to 1276.55) | -3.04 (-3.29 to -2.80) |
| Malawi | 2604.28 (1957.90 to 3323.80) | 4482.64 (3221.41 to 5889.82) | 75.34 (56.40 to 97.19) | 62.47 (45.45 to 81.36) | -0.88 (-1.17 to -0.60) | 80853.57 (60696.23 to 102768.62) | 145688.42 (104830.79 to 193429.83) | 1956.41 (1465.63 to 2503.78) | 1708.07 (1239.12 to 2230.25) | -0.75 (-1.07 to -0.43) |
| Malaysia | 9760.92 (8187.14 to 11466.35) | 19258.69 (15933.89 to 22758.78) | 110.21 (91.62 to 129.94) | 71.93 (59.02 to 85.70) | -1.59 (-1.78 to -1.39) | 285319.68 (238770.80 to 336372.13) | 560695.38 (464357.22 to 658984.74) | 2870.44 (2397.77 to 3379.88) | 1893.70 (1562.51 to 2241.87) | -1.45 (-1.58 to -1.32) |
| Maldives | 127.92 (105.68 to 152.26) | 165.89 (129.32 to 208.03) | 176.69 (145.91 to 211.13) | 55.96 (43.68 to 69.71) | -4.08 (-4.25 to -3.90) | 3969.40 (3263.40 to 4740.62) | 5074.06 (3910.35 to 6298.81) | 4271.37 (3543.65 to 5130.03) | 1325.59 (1036.21 to 1648.76) | -4.22 (-4.48 to -3.96) |
| Mali | 1199.34 (831.87 to 1585.29) | 2742.83 (1917.04 to 3708.35) | 33.31 (23.05 to 44.06) | 35.68 (24.63 to 47.78) | 0.67 (0.49 to 0.84) | 38863.96 (27472.68 to 51950.43) | 89273.72 (63187.32 to 119750.92) | 899.71 (635.15 to 1192.57) | 937.45 (665.75 to 1259.17) | 0.55 (0.37 to 0.73) |
| Malta | 402.79 (334.40 to 475.22) | 317.06 (253.84 to 386.51) | 94.82 (78.55 to 112.79) | 33.72 (27.10 to 40.78) | -3.38 (-3.46 to -3.30) | 11449.85 (9349.10 to 13647.98) | 9217.52 (7179.89 to 11431.31) | 2666.61 (2174.94 to 3185.15) | 1170.80 (911.97 to 1447.82) | -2.68 (-2.78 to -2.58) |
| Marshall Islands | 24.04 (18.67 to 30.30) | 45.52 (32.95 to 60.62) | 139.99 (107.04 to 177.66) | 121.18 (87.97 to 160.41) | -0.31 (-0.40 to -0.22) | 844.71 (661.25 to 1051.96) | 1714.22 (1241.91 to 2274.49) | 4242.13 (3334.23 to 5312.63) | 3794.47 (2758.85 to 4998.30) | -0.27 (-0.35 to -0.18) |
| Mauritania | 451.69 (327.17 to 594.23) | 466.84 (311.33 to 630.08) | 43.84 (31.75 to 57.91) | 21.43 (14.44 to 28.79) | -2.47 (-2.74 to -2.21) | 16207.05 (11757.33 to 21383.30) | 16880.37 (11260.48 to 22812.54) | 1429.75 (1038.73 to 1878.98) | 678.34 (451.90 to 908.31) | -2.55 (-2.79 to -2.31) |
| Mauritius | 805.51 (673.46 to 945.94) | 991.11 (824.78 to 1163.98) | 112.99 (93.42 to 134.38) | 55.01 (45.79 to 64.70) | -2.51 (-2.82 to -2.20) | 24760.43 (20726.93 to 29203.17) | 29481.83 (24420.59 to 34681.78) | 3124.34 (2601.79 to 3695.36) | 1617.27 (1340.63 to 1895.28) | -2.26 (-2.58 to -1.93) |
| Mexico | 26153.55 (21385.97 to 31010.51) | 32298.95 (25184.15 to 40098.42) | 71.37 (57.68 to 85.38) | 26.64 (20.68 to 33.08) | -3.47 (-3.64 to -3.31) | 755517.64 (614483.46 to 902239.53) | 946734.73 (738035.91 to 1184909.64) | 1723.09 (1400.11 to 2064.50) | 727.88 (566.98 to 908.11) | -3.11 (-3.29 to -2.93) |
| Micronesia (Federated States of) | 93.73 (72.03 to 121.18) | 119.45 (88.35 to 158.11) | 188.03 (145.04 to 241.56) | 148.62 (110.29 to 193.11) | -0.79 (-0.84 to -0.74) | 3196.23 (2454.73 to 4136.29) | 4326.74 (3232.02 to 5595.09) | 5832.59 (4468.05 to 7546.19) | 4828.60 (3616.64 to 6185.20) | -0.61 (-0.65 to -0.56) |
| Monaco | 67.98 (50.54 to 86.36) | 71.10 (56.56 to 88.93) | 98.48 (73.51 to 123.87) | 73.19 (58.44 to 92.96) | -0.89 (-1.02 to -0.75) | 1643.50 (1232.64 to 2048.64) | 1693.69 (1334.52 to 2144.36) | 2797.90 (2108.49 to 3475.83) | 2093.53 (1638.37 to 2684.36) | -0.89 (-1.01 to -0.78) |
| Mongolia | 1361.13 (1092.37 to 1649.65) | 2367.69 (1850.95 to 2918.62) | 129.43 (104.10 to 156.55) | 99.48 (76.06 to 122.36) | -1.00 (-1.23 to -0.77) | 42203.23 (33588.44 to 50963.75) | 82067.55 (64104.02 to 100825.58) | 3754.38 (2995.15 to 4532.07) | 2966.35 (2302.93 to 3660.36) | -0.90 (-1.16 to -0.64) |
| Montenegro | 771.30 (659.60 to 884.95) | 1120.03 (918.48 to 1342.48) | 122.66 (104.48 to 141.83) | 114.39 (93.48 to 137.17) | -0.23 (-0.48 to 0.01) | 25286.37 (21546.12 to 29057.65) | 32676.02 (26674.86 to 39296.54) | 3895.81 (3310.28 to 4490.39) | 3410.83 (2784.14 to 4092.87) | -0.44 (-0.71 to -0.16) |
| Morocco | 9996.67 (8092.92 to 12102.41) | 14558.55 (10782.90 to 18054.90) | 70.22 (56.28 to 85.49) | 42.14 (31.36 to 52.81) | -1.73 (-1.92 to -1.53) | 325317.76 (262917.81 to 391616.73) | 469626.16 (352410.01 to 590847.23) | 2089.29 (1693.12 to 2517.26) | 1266.94 (951.18 to 1597.27) | -1.71 (-1.89 to -1.53) |
| Mozambique | 3412.21 (2473.59 to 4515.17) | 5937.82 (4385.65 to 7940.30) | 59.18 (42.80 to 77.40) | 51.56 (37.41 to 68.90) | 0.18 (-0.04 to 0.40) | 113134.90 (81711.07 to 151616.99) | 210143.38 (153934.58 to 283633.53) | 1666.58 (1214.39 to 2213.37) | 1534.43 (1135.24 to 2061.85) | 0.40 (0.15 to 0.65) |
| Myanmar | 57952.16 (44299.12 to 72511.64) | 47057.69 (35380.25 to 60207.39) | 277.38 (213.78 to 348.16) | 108.83 (81.43 to 139.73) | -3.35 (-3.49 to -3.21) | 1687678.17 (1274395.03 to 2118829.35) | 1241786.97 (930849.78 to 1590375.77) | 6992.99 (5329.98 to 8763.00) | 2535.64 (1902.46 to 3234.29) | -3.61 (-3.76 to -3.47) |
| Namibia | 679.96 (506.19 to 881.37) | 874.55 (630.59 to 1134.86) | 123.77 (91.20 to 159.77) | 73.06 (53.52 to 94.24) | -1.98 (-2.34 to -1.61) | 19855.41 (14824.84 to 25686.68) | 25329.89 (17731.19 to 33573.53) | 3016.25 (2262.37 to 3882.52) | 1778.90 (1271.17 to 2307.02) | -2.03 (-2.40 to -1.65) |
| Nauru | 11.87 (9.03 to 14.97) | 12.37 (8.95 to 16.10) | 242.21 (183.12 to 307.62) | 191.69 (140.90 to 246.99) | -0.90 (-1.19 to -0.60) | 436.08 (328.98 to 543.98) | 466.21 (340.61 to 599.36) | 7404.25 (5637.08 to 9263.01) | 6264.03 (4589.68 to 8101.03) | -0.68 (-1.01 to -0.35) |
| Nepal | 18412.93 (13745.71 to 24515.72) | 24097.21 (17656.46 to 31057.83) | 226.11 (168.33 to 295.64) | 120.53 (89.06 to 155.19) | -2.11 (-2.31 to -1.91) | 558846.92 (422599.05 to 736228.18) | 623680.22 (458904.62 to 794862.91) | 5715.02 (4325.00 to 7493.11) | 2739.55 (2038.07 to 3493.74) | -2.50 (-2.67 to -2.33) |
| Netherlands | 25731.95 (22212.78 to 29476.31) | 20611.96 (16878.56 to 24816.05) | 127.71 (110.40 to 145.93) | 55.16 (45.41 to 65.66) | -2.75 (-2.84 to -2.67) | 645482.42 (547425.45 to 743650.67) | 477434.53 (386410.47 to 572708.77) | 3350.28 (2844.88 to 3872.30) | 1447.46 (1160.90 to 1743.66) | -2.72 (-2.79 to -2.65) |
| New Zealand | 3990.12 (3325.83 to 4669.82) | 3400.12 (2757.09 to 4123.19) | 101.20 (84.47 to 118.51) | 39.01 (31.90 to 47.05) | -3.20 (-3.37 to -3.03) | 110099.13 (89075.98 to 131459.18) | 90159.02 (71350.28 to 111616.80) | 2865.95 (2319.94 to 3425.47) | 1153.43 (904.87 to 1443.53) | -3.08 (-3.26 to -2.89) |
| Nicaragua | 473.40 (374.88 to 583.74) | 972.77 (724.77 to 1255.55) | 31.73 (24.98 to 39.44) | 20.67 (15.30 to 26.54) | -1.01 (-1.22 to -0.80) | 16272.76 (12408.79 to 20706.76) | 33582.41 (24964.80 to 43736.43) | 935.82 (721.50 to 1188.31) | 635.99 (471.99 to 822.91) | -0.98 (-1.16 to -0.80) |
| Niger | 607.62 (425.16 to 838.50) | 1121.78 (756.29 to 1543.89) | 23.30 (16.36 to 31.62) | 16.23 (10.96 to 22.16) | -1.19 (-1.26 to -1.12) | 22096.20 (15157.57 to 30716.01) | 39743.39 (26398.23 to 55552.80) | 655.22 (463.83 to 902.58) | 428.67 (287.09 to 585.65) | -1.40 (-1.49 to -1.31) |
| Nigeria | 8720.41 (6196.79 to 11947.08) | 9684.45 (6570.70 to 13429.65) | 20.66 (14.66 to 28.09) | 10.58 (7.41 to 14.60) | -2.36 (-2.59 to -2.12) | 289077.56 (203696.69 to 393259.56) | 353164.36 (241247.92 to 490753.52) | 596.57 (421.88 to 803.04) | 321.38 (223.74 to 443.34) | -2.23 (-2.49 to -1.96) |
| Niue | 2.17 (1.66 to 2.79) | 1.89 (1.43 to 2.53) | 100.74 (77.11 to 129.22) | 86.99 (66.12 to 116.51) | -0.70 (-0.80 to -0.60) | 63.63 (48.28 to 81.76) | 57.66 (43.64 to 77.52) | 3080.37 (2330.33 to 3977.21) | 2664.29 (2008.21 to 3599.06) | -0.72 (-0.82 to -0.62) |
| North Macedonia | 2738.94 (2286.02 to 3174.50) | 3344.92 (2637.23 to 4120.25) | 148.99 (123.36 to 174.74) | 108.96 (84.83 to 134.22) | -1.22 (-1.59 to -0.86) | 84883.60 (69800.77 to 99745.14) | 99378.15 (78068.08 to 121127.04) | 4307.22 (3533.92 to 5053.73) | 3036.91 (2388.77 to 3716.40) | -1.33 (-1.59 to -1.07) |
| Northern Mariana Islands | 21.19 (15.90 to 27.38) | 42.89 (35.32 to 51.97) | 116.36 (91.16 to 147.15) | 81.41 (65.68 to 98.98) | -1.16 (-1.23 to -1.08) | 833.51 (620.51 to 1080.70) | 1437.81 (1161.77 to 1754.00) | 3312.16 (2570.81 to 4195.39) | 2413.55 (1940.20 to 2933.46) | -1.00 (-1.10 to -0.90) |
| Norway | 6529.50 (5364.76 to 7744.59) | 3508.27 (2768.06 to 4339.39) | 94.81 (78.80 to 111.48) | 33.15 (26.67 to 40.50) | -3.71 (-3.89 to -3.53) | 164715.24 (134545.25 to 196492.56) | 89092.33 (70005.15 to 111341.66) | 2694.72 (2208.99 to 3197.71) | 979.61 (760.58 to 1231.84) | -3.56 (-3.70 to -3.42) |
| Oman | 429.69 (310.93 to 577.25) | 555.10 (410.16 to 729.13) | 62.26 (44.20 to 84.22) | 28.81 (21.14 to 37.88) | -2.02 (-2.19 to -1.85) | 15163.09 (11105.78 to 20085.03) | 23494.85 (17189.08 to 30276.62) | 1789.42 (1289.01 to 2408.07) | 823.21 (603.48 to 1068.73) | -2.19 (-2.31 to -2.07) |
| Pakistan | 73424.79 (56825.06 to 90467.45) | 101259.79 (74279.76 to 134472.75) | 139.70 (105.55 to 173.15) | 91.07 (68.15 to 120.27) | -1.68 (-1.98 to -1.37) | 2109064.60 (1659406.06 to 2568634.05) | 3108306.67 (2295780.39 to 4089675.23) | 3612.81 (2818.93 to 4399.13) | 2354.57 (1748.23 to 3082.82) | -1.69 (-2.00 to -1.38) |
| Palau | 11.96 (9.12 to 15.68) | 21.19 (15.70 to 28.07) | 123.54 (94.18 to 162.44) | 94.60 (69.37 to 125.07) | -0.87 (-0.92 to -0.82) | 382.59 (287.28 to 491.97) | 713.89 (527.00 to 940.48) | 3529.21 (2666.94 to 4561.68) | 2839.13 (2098.73 to 3725.50) | -0.73 (-0.79 to -0.68) |
| Palestine | 969.90 (738.90 to 1222.74) | 1681.42 (1338.21 to 2064.87) | 123.25 (94.77 to 154.71) | 73.85 (58.42 to 91.72) | -1.90 (-2.17 to -1.63) | 26256.71 (20139.88 to 33068.75) | 54121.55 (42618.79 to 65880.94) | 2979.31 (2285.23 to 3738.77) | 1939.03 (1523.92 to 2378.45) | -1.59 (-1.80 to -1.39) |
| Panama | 612.41 (488.86 to 748.41) | 844.22 (608.28 to 1133.67) | 43.00 (34.16 to 52.84) | 18.89 (13.60 to 25.30) | -2.95 (-3.10 to -2.80) | 16934.46 (13507.28 to 20905.63) | 23474.19 (17191.14 to 31092.41) | 1106.43 (881.36 to 1372.11) | 530.82 (388.47 to 702.56) | -2.65 (-2.80 to -2.49) |
| Papua New Guinea | 2204.42 (1530.11 to 2976.71) | 4799.60 (3403.42 to 6504.20) | 121.34 (84.01 to 164.65) | 90.56 (63.68 to 122.92) | -1.02 (-1.08 to -0.97) | 77587.85 (53634.87 to 103821.16) | 178675.92 (128063.05 to 238466.05) | 3565.18 (2481.93 to 4811.44) | 2762.19 (1977.42 to 3707.53) | -0.90 (-0.95 to -0.85) |
| Paraguay | 1770.41 (1432.66 to 2159.36) | 3462.39 (2467.73 to 4581.95) | 84.13 (67.85 to 102.44) | 61.94 (44.36 to 81.72) | -0.90 (-1.00 to -0.79) | 49167.62 (39657.27 to 60823.12) | 94714.07 (67680.42 to 125243.92) | 2143.64 (1735.43 to 2639.14) | 1590.39 (1138.53 to 2102.99) | -0.99 (-1.08 to -0.90) |
| Peru | 3294.58 (2538.59 to 4127.97) | 5757.80 (4161.44 to 8018.94) | 28.07 (21.65 to 35.28) | 17.13 (12.39 to 23.84) | -1.99 (-2.36 to -1.61) | 99635.04 (75817.87 to 125023.73) | 169009.56 (124598.03 to 232884.90) | 768.02 (582.40 to 965.00) | 487.50 (358.95 to 670.87) | -1.85 (-2.20 to -1.50) |
| Philippines | 37379.34 (30705.77 to 44766.15) | 72992.93 (56154.68 to 91601.48) | 141.43 (113.05 to 172.33) | 90.94 (69.88 to 113.56) | -1.28 (-1.44 to -1.11) | 1249441.18 (1027035.72 to 1503586.65) | 2348779.64 (1809319.41 to 2911631.19) | 3759.16 (3077.66 to 4523.66) | 2591.76 (1988.38 to 3207.79) | -1.17 (-1.33 to -1.02) |
| Poland | 70384.53 (61460.64 to 79475.52) | 51198.30 (43032.77 to 60697.64) | 161.72 (141.09 to 183.56) | 71.26 (60.06 to 84.27) | -2.74 (-2.81 to -2.67) | 2203407.08 (1894586.16 to 2527461.95) | 1479458.85 (1219427.30 to 1775701.55) | 5054.57 (4348.92 to 5797.44) | 2249.90 (1847.62 to 2708.23) | -2.65 (-2.73 to -2.57) |
| Portugal | 10104.19 (8401.29 to 11805.75) | 6996.83 (5649.24 to 8433.24) | 73.32 (60.85 to 85.72) | 30.53 (25.18 to 36.04) | -2.96 (-3.07 to -2.85) | 292729.38 (237859.43 to 346669.18) | 208975.17 (163778.42 to 253687.03) | 2205.79 (1788.03 to 2620.68) | 1103.93 (859.66 to 1344.43) | -2.31 (-2.39 to -2.24) |
| Puerto Rico | 1869.08 (1456.35 to 2359.64) | 1726.80 (1246.20 to 2308.02) | 52.66 (40.96 to 66.79) | 23.91 (17.65 to 31.85) | -2.90 (-3.06 to -2.75) | 50685.91 (39191.11 to 64301.55) | 45837.66 (34025.32 to 61295.93) | 1412.43 (1091.21 to 1794.13) | 767.26 (567.93 to 1031.07) | -2.28 (-2.41 to -2.15) |
| Qatar | 102.18 (78.11 to 131.36) | 317.36 (222.70 to 424.93) | 96.60 (72.81 to 124.03) | 33.18 (22.99 to 44.44) | -3.80 (-4.41 to -3.18) | 4180.02 (3164.24 to 5398.16) | 18043.04 (12756.46 to 24008.20) | 2493.10 (1907.22 to 3204.67) | 997.96 (705.14 to 1322.94) | -3.18 (-3.62 to -2.74) |
| Republic of Korea | 33963.86 (27496.89 to 39905.21) | 40926.58 (33305.53 to 49498.56) | 129.74 (103.36 to 156.35) | 43.89 (35.61 to 53.08) | -3.82 (-3.97 to -3.67) | 1072375.82 (866238.71 to 1270542.35) | 1038682.71 (820837.49 to 1264261.50) | 3347.76 (2680.73 to 3994.40) | 1148.73 (900.40 to 1407.89) | -3.72 (-3.84 to -3.61) |
| Republic of Moldova | 4774.55 (3985.16 to 5669.80) | 4672.45 (3828.04 to 5528.94) | 107.82 (89.68 to 128.74) | 78.67 (64.56 to 92.82) | -1.19 (-1.50 to -0.88) | 149928.08 (124813.94 to 177480.14) | 143084.98 (118414.23 to 169371.99) | 3249.91 (2707.04 to 3856.79) | 2501.17 (2073.61 to 2953.31) | -0.96 (-1.27 to -0.64) |
| Romania | 31528.06 (26770.05 to 36128.86) | 25483.42 (20636.79 to 30359.69) | 116.79 (98.69 to 135.17) | 73.19 (59.40 to 86.76) | -2.07 (-2.30 to -1.85) | 1012823.56 (845631.42 to 1170255.13) | 778067.71 (614417.34 to 932666.52) | 3635.06 (3028.16 to 4216.99) | 2470.89 (1951.21 to 2963.09) | -1.81 (-2.04 to -1.59) |
| Russian Federation | 208008.26 (183991.69 to 231247.13) | 196882.93 (163798.72 to 229031.87) | 114.28 (100.82 to 127.54) | 83.33 (69.50 to 96.86) | -1.54 (-2.18 to -0.90) | 6626659.37 (5807755.78 to 7463116.72) | 6053298.62 (5047379.80 to 7102033.30) | 3624.00 (3173.73 to 4085.77) | 2686.95 (2246.44 to 3146.82) | -1.53 (-2.17 to -0.88) |
| Rwanda | 4129.46 (3088.74 to 5288.93) | 4250.44 (3054.61 to 5631.11) | 160.53 (118.90 to 205.36) | 80.40 (58.09 to 106.89) | -3.17 (-3.54 to -2.79) | 126714.19 (93839.30 to 164853.46) | 126470.24 (90087.80 to 168703.37) | 4156.21 (3086.25 to 5378.79) | 1945.30 (1406.70 to 2582.87) | -3.46 (-3.86 to -3.06) |
| Saint Kitts and Nevis | 16.96 (13.13 to 21.49) | 15.55 (11.43 to 20.53) | 46.22 (36.22 to 58.09) | 23.46 (17.10 to 30.61) | -2.35 (-2.50 to -2.21) | 436.67 (341.67 to 546.01) | 477.97 (346.10 to 631.88) | 1275.73 (998.56 to 1586.49) | 641.28 (463.97 to 841.89) | -2.49 (-2.67 to -2.31) |
| Saint Lucia | 50.32 (39.94 to 61.87) | 66.73 (48.15 to 87.75) | 59.80 (47.10 to 74.24) | 27.83 (20.07 to 36.48) | -2.90 (-3.14 to -2.66) | 1457.94 (1158.57 to 1781.71) | 1983.81 (1467.58 to 2587.30) | 1667.26 (1323.28 to 2037.67) | 816.61 (604.55 to 1065.82) | -2.63 (-2.79 to -2.46) |
| Saint Vincent and the Grenadines | 26.75 (21.07 to 32.82) | 40.20 (30.71 to 50.78) | 37.96 (29.83 to 46.41) | 28.46 (21.71 to 35.89) | -1.10 (-1.24 to -0.97) | 803.67 (632.96 to 979.61) | 1235.73 (953.95 to 1563.48) | 1136.77 (896.58 to 1381.73) | 861.17 (662.08 to 1088.85) | -1.06 (-1.18 to -0.94) |
| Samoa | 113.97 (89.44 to 141.07) | 156.00 (122.09 to 194.83) | 137.47 (107.67 to 170.61) | 108.89 (84.83 to 134.51) | -0.90 (-0.98 to -0.81) | 3585.77 (2800.53 to 4441.96) | 5162.65 (3933.69 to 6475.35) | 3917.60 (3051.42 to 4848.68) | 3291.24 (2525.23 to 4101.57) | -0.69 (-0.77 to -0.60) |
| San Marino | 25.32 (20.18 to 31.12) | 19.68 (12.86 to 28.27) | 70.76 (56.73 to 86.95) | 25.28 (15.99 to 36.68) | -2.50 (-2.81 to -2.19) | 653.87 (523.97 to 809.13) | 547.00 (375.61 to 762.44) | 1976.62 (1569.65 to 2445.93) | 874.13 (599.17 to 1215.38) | -2.14 (-2.34 to -1.95) |
| Sao Tome and Principe | 15.16 (10.80 to 20.24) | 26.49 (19.30 to 35.51) | 23.37 (16.63 to 31.31) | 23.55 (17.28 to 30.88) | -0.12 (-0.46 to 0.23) | 478.30 (344.17 to 632.03) | 896.65 (651.35 to 1218.88) | 709.05 (511.10 to 934.42) | 690.83 (506.33 to 918.87) | -0.29 (-0.63 to 0.06) |
| Saudi Arabia | 3153.91 (2171.72 to 4295.62) | 10860.32 (7853.50 to 14200.58) | 50.77 (34.91 to 69.43) | 43.03 (31.70 to 55.08) | -0.41 (-0.60 to -0.22) | 114809.31 (79936.49 to 155686.46) | 473067.06 (346168.97 to 619660.52) | 1534.01 (1072.06 to 2056.86) | 1433.65 (1057.74 to 1842.61) | 0.01 (-0.19 to 0.22) |
| Senegal | 1516.55 (1123.16 to 1956.91) | 1922.53 (1355.53 to 2582.92) | 43.71 (32.18 to 56.30) | 23.43 (16.44 to 31.48) | -2.11 (-2.27 to -1.95) | 55164.98 (41164.34 to 71384.85) | 69357.09 (48311.56 to 90806.54) | 1463.03 (1095.23 to 1895.38) | 763.48 (530.50 to 1011.91) | -2.20 (-2.33 to -2.07) |
| Serbia | 14937.86 (12597.57 to 17311.56) | 14821.54 (12017.78 to 17885.66) | 139.40 (116.80 to 162.73) | 91.04 (73.93 to 109.24) | -1.62 (-1.92 to -1.31) | 469156.64 (393472.69 to 541464.54) | 425766.19 (343019.42 to 515301.71) | 4038.02 (3397.31 to 4701.27) | 2867.97 (2306.68 to 3471.62) | -1.34 (-1.62 to -1.06) |
| Seychelles | 66.85 (54.64 to 78.36) | 76.81 (62.57 to 93.59) | 119.10 (97.41 to 139.63) | 68.88 (56.06 to 84.62) | -1.66 (-1.84 to -1.49) | 1798.93 (1470.63 to 2102.86) | 2291.24 (1840.70 to 2792.00) | 3216.48 (2627.34 to 3756.12) | 1880.00 (1510.61 to 2298.16) | -1.68 (-1.84 to -1.53) |
| Sierra Leone | 1097.37 (791.51 to 1435.48) | 1243.38 (868.08 to 1681.78) | 53.25 (38.75 to 69.77) | 31.00 (21.91 to 42.00) | -1.52 (-1.69 to -1.35) | 37139.99 (27214.57 to 48714.70) | 46237.10 (32563.99 to 62356.18) | 1657.86 (1207.54 to 2170.35) | 1006.51 (711.08 to 1349.61) | -1.37 (-1.56 to -1.18) |
| Singapore | 1792.52 (1458.97 to 2140.46) | 1716.55 (1370.48 to 2083.96) | 84.41 (68.02 to 101.94) | 19.92 (15.93 to 24.23) | -4.55 (-4.62 to -4.49) | 54198.09 (43259.43 to 64869.50) | 53242.66 (41332.05 to 65956.83) | 2261.83 (1812.60 to 2699.93) | 607.41 (473.47 to 753.81) | -4.14 (-4.19 to -4.08) |
| Slovakia | 7868.13 (6674.74 to 9060.97) | 5969.52 (4806.92 to 7201.11) | 132.63 (112.86 to 152.64) | 62.65 (50.40 to 75.55) | -2.29 (-2.38 to -2.20) | 239377.57 (202160.56 to 276650.99) | 177477.36 (141368.29 to 216361.16) | 4075.59 (3449.47 to 4700.79) | 1978.05 (1570.53 to 2416.88) | -2.23 (-2.32 to -2.14) |
| Slovenia | 2063.84 (1730.19 to 2412.76) | 1674.49 (1330.93 to 2061.18) | 83.40 (69.54 to 97.13) | 38.63 (30.74 to 47.47) | -2.56 (-2.66 to -2.45) | 66823.45 (54369.91 to 79199.46) | 51969.59 (40238.69 to 64523.84) | 2732.69 (2222.44 to 3240.86) | 1379.00 (1051.79 to 1721.23) | -2.20 (-2.29 to -2.11) |
| Solomon Islands | 259.31 (172.42 to 339.00) | 572.75 (425.87 to 749.42) | 189.76 (132.09 to 242.90) | 157.42 (117.24 to 203.09) | -0.46 (-0.67 to -0.24) | 8719.08 (5699.28 to 11535.98) | 20395.85 (15173.61 to 26319.77) | 5459.53 (3683.67 to 7124.25) | 4797.06 (3591.52 to 6194.24) | -0.26 (-0.47 to -0.04) |
| Somalia | 2205.29 (1488.30 to 3247.77) | 3644.13 (2420.89 to 5228.57) | 86.03 (59.82 to 122.53) | 53.92 (36.65 to 75.41) | -1.60 (-1.66 to -1.53) | 81505.61 (53871.58 to 120592.08) | 138528.04 (91590.87 to 199935.68) | 2517.57 (1709.56 to 3608.18) | 1639.52 (1109.28 to 2308.56) | -1.52 (-1.60 to -1.45) |
| South Africa | 21523.57 (17484.87 to 25780.48) | 25561.81 (20399.57 to 30650.14) | 101.34 (82.08 to 122.32) | 53.95 (42.89 to 65.13) | -2.20 (-2.57 to -1.83) | 766512.22 (619407.32 to 923241.11) | 867634.80 (691295.04 to 1051907.91) | 3203.00 (2586.63 to 3862.85) | 1670.84 (1334.94 to 2025.48) | -2.18 (-2.52 to -1.84) |
| South Sudan | 1770.68 (1225.71 to 2586.50) | 1818.43 (1194.58 to 2503.10) | 70.36 (49.18 to 102.26) | 47.84 (32.05 to 66.29) | -1.49 (-1.74 to -1.25) | 55219.10 (38061.21 to 80095.00) | 61821.49 (40920.84 to 85201.46) | 1991.12 (1370.43 to 2897.55) | 1333.33 (895.93 to 1832.65) | -1.56 (-1.82 to -1.29) |
| Spain | 52414.71 (44622.34 to 59823.52) | 41161.23 (33691.93 to 48893.54) | 96.93 (82.49 to 110.60) | 41.34 (34.43 to 48.24) | -2.76 (-2.85 to -2.66) | 1380114.39 (1165378.03 to 1587035.38) | 1067551.02 (870199.49 to 1270382.91) | 2690.19 (2265.25 to 3102.77) | 1266.53 (1035.35 to 1518.99) | -2.42 (-2.52 to -2.32) |
| Sri Lanka | 8562.17 (6922.74 to 10473.39) | 8997.94 (5527.41 to 12772.74) | 93.04 (74.67 to 115.36) | 35.64 (22.27 to 49.97) | -2.80 (-2.94 to -2.66) | 241756.01 (197456.86 to 293001.92) | 245280.43 (158702.23 to 339549.67) | 2192.58 (1768.53 to 2684.39) | 918.46 (599.50 to 1264.42) | -2.74 (-2.87 to -2.60) |
| Sudan | 8434.11 (6362.60 to 10807.04) | 11287.46 (7882.56 to 15977.54) | 93.69 (70.17 to 119.57) | 60.79 (43.22 to 85.51) | -1.56 (-1.63 to -1.48) | 265840.07 (200242.26 to 338963.99) | 363214.50 (256628.21 to 507391.86) | 2614.93 (1966.76 to 3333.84) | 1623.05 (1181.17 to 2258.17) | -1.71 (-1.80 to -1.63) |
| Suriname | 197.79 (161.14 to 236.76) | 278.44 (202.53 to 378.83) | 76.80 (62.33 to 92.22) | 43.00 (31.14 to 58.94) | -1.97 (-2.23 to -1.72) | 6360.89 (5185.27 to 7573.17) | 9312.72 (6869.46 to 12478.09) | 2304.76 (1866.00 to 2759.63) | 1397.53 (1028.78 to 1875.47) | -1.77 (-2.00 to -1.54) |
| Sweden | 12509.99 (10151.55 to 15128.81) | 8528.85 (6644.10 to 10647.97) | 80.02 (65.84 to 95.85) | 35.87 (28.43 to 44.54) | -2.46 (-2.56 to -2.36) | 294105.36 (236010.56 to 355254.74) | 201184.66 (153884.32 to 253986.82) | 2112.52 (1687.46 to 2550.89) | 1014.22 (772.77 to 1294.92) | -2.28 (-2.34 to -2.22) |
| Switzerland | 9375.78 (7723.46 to 11035.12) | 7042.49 (5751.21 to 8531.94) | 88.63 (73.93 to 103.94) | 35.77 (29.54 to 42.59) | -2.76 (-2.86 to -2.66) | 239813.61 (194916.13 to 286703.63) | 181443.08 (142838.67 to 222936.71) | 2472.61 (2011.04 to 2955.18) | 1109.52 (865.36 to 1365.42) | -2.51 (-2.59 to -2.43) |
| Syrian Arab Republic | 6457.67 (5002.79 to 8128.40) | 10688.30 (7561.84 to 14444.86) | 126.80 (97.89 to 160.48) | 89.52 (64.02 to 119.11) | -1.48 (-1.62 to -1.33) | 207141.47 (160064.89 to 258146.57) | 330768.26 (236747.42 to 437289.05) | 3536.93 (2738.64 to 4404.73) | 2337.98 (1678.81 to 3071.92) | -1.67 (-1.83 to -1.51) |
| Taiwan (Province of China) | 14120.75 (11836.05 to 16253.85) | 22070.71 (18128.83 to 25801.38) | 96.41 (80.05 to 111.89) | 51.34 (42.30 to 59.94) | -2.07 (-2.19 to -1.95) | 421388.18 (349063.45 to 492128.19) | 573275.54 (458168.49 to 679279.05) | 2540.09 (2104.88 to 2963.21) | 1416.30 (1134.06 to 1681.71) | -1.92 (-1.99 to -1.85) |
| Tajikistan | 3059.03 (2462.32 to 3685.70) | 2544.09 (1868.25 to 3440.79) | 113.15 (90.33 to 136.98) | 47.21 (34.53 to 65.05) | -2.91 (-3.22 to -2.60) | 92520.36 (75523.74 to 111135.84) | 84672.35 (62046.90 to 113146.53) | 3176.66 (2595.79 to 3801.19) | 1277.52 (940.48 to 1706.57) | -3.17 (-3.43 to -2.90) |
| Thailand | 37325.26 (29804.38 to 44476.76) | 56850.93 (40885.71 to 76757.92) | 115.97 (91.67 to 137.81) | 52.67 (38.00 to 71.06) | -3.05 (-3.22 to -2.88) | 1083096.95 (871544.19 to 1295204.38) | 1528827.74 (1122610.96 to 2051614.79) | 2907.96 (2332.85 to 3466.61) | 1448.05 (1068.25 to 1935.08) | -2.71 (-2.87 to -2.55) |
| Timor-Leste | 307.36 (229.52 to 416.54) | 780.88 (567.11 to 1017.27) | 119.51 (92.06 to 159.88) | 100.64 (73.05 to 132.04) | -0.44 (-0.61 to -0.28) | 10508.21 (7545.65 to 14226.15) | 22228.43 (16441.53 to 29080.81) | 3122.34 (2372.97 to 4184.01) | 2562.86 (1890.78 to 3335.60) | -0.54 (-0.76 to -0.32) |
| Togo | 887.32 (645.00 to 1173.29) | 1829.02 (1244.61 to 2548.72) | 74.95 (54.77 to 99.10) | 47.17 (32.62 to 65.20) | -1.56 (-1.67 to -1.45) | 29290.26 (21199.54 to 38786.45) | 62896.69 (43088.19 to 87123.57) | 2079.23 (1506.42 to 2741.94) | 1368.31 (938.54 to 1890.99) | -1.40 (-1.53 to -1.26) |
| Tokelau | 1.48 (1.10 to 1.94) | 1.16 (0.85 to 1.53) | 111.75 (83.62 to 145.96) | 78.67 (57.59 to 104.49) | -1.28 (-1.35 to -1.22) | 43.05 (31.54 to 56.31) | 35.49 (26.64 to 47.12) | 3232.44 (2351.79 to 4233.17) | 2457.90 (1848.93 to 3274.11) | -1.03 (-1.08 to -0.97) |
| Tonga | 71.91 (56.52 to 89.64) | 87.28 (65.61 to 112.49) | 140.94 (110.26 to 176.74) | 111.16 (83.58 to 142.69) | -0.84 (-1.01 to -0.68) | 2049.38 (1604.64 to 2571.47) | 2401.29 (1804.07 to 3118.86) | 3604.72 (2809.47 to 4528.57) | 2938.31 (2211.19 to 3796.38) | -0.71 (-0.84 to -0.59) |
| Trinidad and Tobago | 620.88 (506.28 to 740.47) | 715.85 (494.92 to 967.72) | 75.24 (61.57 to 90.25) | 36.86 (25.45 to 49.79) | -2.74 (-2.99 to -2.49) | 19040.32 (15438.15 to 22760.10) | 22842.94 (15931.48 to 30425.64) | 2183.33 (1771.91 to 2613.61) | 1190.62 (831.79 to 1585.19) | -2.38 (-2.61 to -2.15) |
| Tunisia | 4728.47 (3914.43 to 5651.69) | 9128.93 (6625.49 to 12467.60) | 105.96 (87.30 to 127.99) | 72.24 (52.20 to 98.58) | -1.54 (-1.67 to -1.40) | 132989.76 (108904.58 to 158504.15) | 254579.75 (193109.97 to 341091.66) | 2608.28 (2136.76 to 3130.59) | 1880.97 (1424.18 to 2511.97) | -1.32 (-1.42 to -1.22) |
| Türkiye | 54583.68 (44961.38 to 64365.67) | 70737.91 (54651.81 to 90630.54) | 160.77 (132.08 to 189.13) | 76.78 (59.08 to 99.03) | -2.57 (-2.86 to -2.28) | 1699908.59 (1383275.24 to 2009477.39) | 2068002.16 (1611601.26 to 2600442.37) | 4493.70 (3647.05 to 5302.77) | 2142.33 (1666.66 to 2692.08) | -2.62 (-2.87 to -2.37) |
| Turkmenistan | 2280.55 (1949.36 to 2632.15) | 2802.39 (2061.00 to 3657.26) | 118.38 (100.53 to 137.99) | 67.92 (50.38 to 88.10) | -2.39 (-2.73 to -2.05) | 74874.10 (63426.55 to 87052.10) | 95429.51 (71282.43 to 123450.60) | 3543.55 (3008.37 to 4118.71) | 2075.95 (1545.09 to 2677.60) | -2.32 (-2.68 to -1.96) |
| Tuvalu | 11.79 (9.15 to 14.98) | 12.97 (10.08 to 16.64) | 170.79 (131.63 to 218.66) | 123.46 (96.13 to 156.27) | -1.01 (-1.06 to -0.95) | 389.45 (299.57 to 499.28) | 431.39 (331.29 to 548.79) | 5183.76 (3996.67 to 6626.27) | 3870.33 (2969.08 to 4924.05) | -0.89 (-0.95 to -0.82) |
| Uganda | 2868.07 (2086.72 to 3936.73) | 4167.13 (2936.49 to 5647.64) | 47.30 (34.78 to 64.96) | 29.03 (20.61 to 39.88) | -2.52 (-2.99 to -2.04) | 91546.91 (65140.49 to 125521.19) | 143567.62 (99835.45 to 196538.33) | 1299.79 (938.79 to 1762.62) | 828.85 (580.51 to 1121.75) | -2.42 (-2.91 to -1.94) |
| Ukraine | 88450.19 (77378.30 to 100241.68) | 61420.61 (41756.16 to 85109.85) | 124.89 (109.01 to 141.72) | 81.42 (55.13 to 112.76) | -1.98 (-2.49 to -1.47) | 2581853.99 (2232467.18 to 2937304.83) | 1810502.45 (1240374.78 to 2486688.86) | 3673.21 (3173.74 to 4187.34) | 2567.24 (1754.16 to 3524.90) | -1.75 (-2.24 to -1.27) |
| United Arab Emirates | 391.69 (286.98 to 521.76) | 1316.05 (939.33 to 1769.55) | 85.99 (63.39 to 114.93) | 46.13 (33.05 to 61.85) | -0.87 (-1.41 to -0.33) | 16487.71 (11970.70 to 21865.81) | 73756.41 (52534.95 to 98593.57) | 2335.86 (1725.39 to 3120.51) | 1142.97 (822.94 to 1511.88) | -1.69 (-2.13 to -1.25) |
| United Kingdom | 139138.58 (119170.86 to 159522.97) | 73532.65 (59755.16 to 88460.53) | 150.65 (129.03 to 172.09) | 53.23 (43.64 to 63.79) | -3.49 (-3.62 to -3.37) | 3308674.78 (2777996.65 to 3836886.52) | 1742824.45 (1373155.73 to 2115807.61) | 3895.30 (3251.31 to 4533.26) | 1460.60 (1147.76 to 1781.23) | -3.26 (-3.36 to -3.16) |
| United Republic of Tanzania | 8419.98 (6354.56 to 10955.14) | 11119.87 (8089.29 to 15210.65) | 80.79 (60.80 to 106.02) | 44.06 (32.50 to 59.37) | -2.44 (-2.62 to -2.26) | 277651.40 (206750.05 to 365105.17) | 386216.79 (282380.51 to 519240.71) | 2304.18 (1726.96 to 3006.02) | 1291.31 (941.17 to 1747.11) | -2.32 (-2.49 to -2.15) |
| United States of America | 369589.53 (314448.58 to 427628.74) | 342532.07 (279694.91 to 408001.88) | 116.32 (99.30 to 133.98) | 57.56 (47.20 to 68.24) | -2.45 (-2.53 to -2.37) | 10326699.64 (8604003.95 to 12064764.63) | 9532101.91 (7630777.07 to 11512956.25) | 3433.76 (2864.63 to 4002.64) | 1762.46 (1397.46 to 2134.38) | -2.27 (-2.31 to -2.22) |
| United States Virgin Islands | 38.83 (28.61 to 52.47) | 41.76 (29.16 to 57.65) | 46.23 (33.82 to 62.53) | 23.37 (16.35 to 32.18) | -2.17 (-2.36 to -1.97) | 1248.17 (916.81 to 1684.36) | 1191.46 (858.55 to 1614.76) | 1323.57 (975.90 to 1782.36) | 735.94 (529.55 to 994.23) | -1.85 (-2.00 to -1.71) |
| Uruguay | 3931.45 (3338.59 to 4467.87) | 3431.66 (2787.19 to 4009.50) | 101.94 (86.75 to 115.66) | 64.06 (52.69 to 74.26) | -1.68 (-1.77 to -1.60) | 115190.44 (97394.08 to 131490.70) | 95348.31 (76883.95 to 112224.00) | 3119.43 (2635.53 to 3574.29) | 1978.33 (1593.26 to 2331.08) | -1.66 (-1.74 to -1.59) |
| Uzbekistan | 6059.85 (4873.74 to 7265.67) | 12435.55 (9647.93 to 15262.70) | 51.81 (41.40 to 62.15) | 47.16 (36.60 to 58.13) | -0.33 (-0.82 to 0.16) | 202732.83 (162672.76 to 243363.62) | 431633.12 (339305.10 to 528827.84) | 1647.25 (1315.54 to 1972.47) | 1426.80 (1119.64 to 1750.96) | -0.59 (-1.05 to -0.12) |
| Vanuatu | 83.53 (63.78 to 106.84) | 163.28 (126.04 to 203.68) | 132.76 (101.49 to 168.48) | 90.55 (69.51 to 112.19) | -1.47 (-1.58 to -1.35) | 2822.04 (2140.19 to 3652.29) | 5705.40 (4412.14 to 7185.93) | 3789.29 (2883.34 to 4841.43) | 2724.69 (2101.96 to 3401.58) | -1.30 (-1.41 to -1.19) |
| Venezuela (Bolivarian Republic of) | 6301.62 (5136.88 to 7501.26) | 11526.28 (8135.50 to 15380.27) | 66.19 (53.73 to 79.32) | 38.57 (27.25 to 51.44) | -2.12 (-2.31 to -1.94) | 201284.64 (160522.45 to 240379.28) | 343707.07 (242892.26 to 458207.83) | 1888.98 (1521.83 to 2257.94) | 1106.77 (785.26 to 1476.81) | -2.10 (-2.27 to -1.93) |
| Viet Nam | 45679.71 (34987.20 to 58733.71) | 84571.24 (65309.38 to 101771.67) | 118.40 (90.79 to 152.97) | 88.08 (68.69 to 105.38) | -1.05 (-1.11 to -0.98) | 1256547.20 (954820.84 to 1607346.33) | 2455386.44 (1877889.72 to 3005915.91) | 3053.51 (2321.80 to 3911.90) | 2332.34 (1789.24 to 2820.27) | -0.88 (-0.92 to -0.85) |
| Yemen | 6483.20 (4789.90 to 8623.74) | 13137.73 (9148.09 to 17664.37) | 130.93 (97.59 to 171.42) | 96.88 (67.55 to 129.30) | -1.15 (-1.24 to -1.06) | 219052.16 (161071.95 to 289571.17) | 437538.75 (307347.65 to 587004.46) | 3845.09 (2849.10 to 5072.83) | 2664.38 (1874.01 to 3507.35) | -1.39 (-1.48 to -1.30) |
| Zambia | 1992.48 (1519.96 to 2560.51) | 2870.15 (1951.80 to 3783.63) | 74.65 (56.55 to 96.72) | 45.14 (31.15 to 60.14) | -2.29 (-2.62 to -1.96) | 62100.74 (47412.44 to 79949.75) | 93727.09 (64544.15 to 124160.11) | 2002.51 (1523.57 to 2566.85) | 1197.51 (828.92 to 1561.52) | -2.40 (-2.77 to -2.03) |
| Zimbabwe | 3685.71 (2806.38 to 4802.95) | 7205.17 (5157.32 to 9446.29) | 98.28 (74.80 to 129.11) | 105.78 (76.50 to 137.16) | 0.65 (0.12 to 1.19) | 111083.49 (84127.54 to 144488.62) | 240180.92 (171456.25 to 315603.39) | 2568.54 (1971.55 to 3363.83) | 2951.49 (2107.58 to 3830.87) | 0.80 (0.23 to 1.37) |

ASDR, age-standardized Deaths rates; ASDALYR, age-standardized disability-adjusted life years rates; CI, confidence interval; DALYs, disability-adjusted life years rates; EAPC, estimated annual percentage change; UI, uncertainty interval.

Table S5 Deaths and DALYs of chewing tobacco in 1990 and 2021 at regional levels

| Region | Deaths (×1000, 95% UI) | | ASDR per 100,000 (95% UI) | | 1990-2021 EAPC,  (95% CI) | DALYs (×1000, 95% UI) | | ASDALYR per 100,000 (95% UI) | | 1990-2021 EAPC,  (95% CI) |
| --- | --- | --- | --- | --- | --- | --- | --- | --- | --- | --- |
|  | 1990 | 2021 | 1990 | 2021 |  | 1990 | 2021 | 1990 | 2021 |  |
| High SDI | 1.48 (1.02 to 2.03) | 2.2 (1.48 to 3.04) | 0.13 (0.09 to 0.18) | 0.11 (0.07 to 0.14) | -0.91 (-1.02 to -0.80) | 35.99 (24.71 to 49.08) | 49.52 (33.5 to 67.18) | 3.36 (2.30 to 4.59) | 2.61 (1.78 to 3.57) | -0.87 (-0.95 to -0.78) |
| High-middle SDI | 1.1 (0.74 to 1.54) | 2.07 (1.35 to 2.95) | 0.11 (0.07 to 0.15) | 0.11 (0.07 to 0.15) | -0.33 (-0.44 to -0.21) | 33.9 (22.51 to 48.05) | 57.13 (37.31 to 82.77) | 3.29 (2.18 to 4.69) | 2.96 (1.94 to 4.28) | -0.57 (-0.69 to -0.45) |
| Middle SDI | 6.15 (4.79 to 7.62) | 15.54 (12.47 to 19.21) | 0.61 (0.48 to 0.74) | 0.59 (0.47 to 0.72) | -0.37 (-0.48 to -0.27) | 184.36 (142.31 to 232.16) | 423.41 (328.83 to 533.43) | 16.17 (12.53 to 20.29) | 15.10 (11.80 to 18.93) | -0.49 (-0.59 to -0.39) |
| Low-middle SDI | 13.23 (10.39 to 15.89) | 30.15 (24.34 to 36.45) | 2.16 (1.69 to 2.59) | 2.13 (1.72 to 2.57) | -0.16 (-0.23 to -0.08) | 400.92 (313.59 to 481.77) | 853.3 (681.65 to 1035.39) | 58.79 (46.26 to 70.70) | 55.31 (44.37 to 67.10) | -0.30 (-0.37 to -0.24) |
| Low SDI | 3.2 (2.42 to 3.91) | 6.87 (5.25 to 8.44) | 1.43 (1.08 to 1.73) | 1.42 (1.09 to 1.74) | -0.18 (-0.28 to -0.09) | 97.31 (73.43 to 119.29) | 198.27 (150.91 to 245.33) | 38.37 (29.08 to 46.99) | 35.49 (27.20 to 43.65) | -0.46 (-0.56 to -0.36) |
| Andean Latin America | 0 (0 to 0.01) | 0.01 (0.01 to 0.01) | 0.02 (0.01 to 0.03) | 0.02 (0.01 to 0.02) | -0.91 (-1.01 to -0.80) | 0.11 (0.08 to 0.15) | 0.22 (0.15 to 0.31) | 0.50 (0.35 to 0.67) | 0.36 (0.24 to 0.50) | -1.15 (-1.26 to -1.04) |
| Australasia | 0.01 (0.01 to 0.02) | 0.03 (0.02 to 0.04) | 0.06 (0.04 to 0.09) | 0.05 (0.03 to 0.08) | -0.65 (-0.92 to -0.37) | 0.35 (0.23 to 0.5) | 0.6 (0.39 to 0.85) | 1.51 (1.01 to 2.16) | 1.17 (0.78 to 1.64) | -0.73 (-1.01 to -0.45) |
| Caribbean | 0.01 (0.01 to 0.02) | 0.02 (0.02 to 0.03) | 0.05 (0.03 to 0.06) | 0.04 (0.03 to 0.06) | -0.04 (-0.16 to 0.08) | 0.32 (0.23 to 0.42) | 0.62 (0.43 to 0.84) | 1.20 (0.86 to 1.59) | 1.15 (0.81 to 1.55) | 0.14 (0.00 to 0.28) |
| Central Asia | 0.11 (0.07 to 0.15) | 0.08 (0.06 to 0.12) | 0.22 (0.15 to 0.30) | 0.10 (0.07 to 0.13) | -2.67 (-2.96 to -2.37) | 3.2 (2.17 to 4.49) | 2.6 (1.8 to 3.58) | 6.32 (4.29 to 8.75) | 2.78 (1.93 to 3.81) | -2.73 (-3.03 to -2.44) |
| Central Europe | 0.05 (0.04 to 0.07) | 0.09 (0.07 to 0.12) | 0.03 (0.02 to 0.05) | 0.04 (0.03 to 0.06) | 0.81 (0.69 to 0.94) | 1.44 (1.01 to 1.93) | 2.38 (1.68 to 3.25) | 0.98 (0.68 to 1.31) | 1.24 (0.87 to 1.72) | 0.76 (0.66 to 0.86) |
| Central Latin America | 0.03 (0.02 to 0.04) | 0.06 (0.05 to 0.09) | 0.04 (0.03 to 0.05) | 0.03 (0.02 to 0.03) | -1.26 (-1.33 to -1.18) | 0.77 (0.56 to 1.01) | 1.63 (1.15 to 2.17) | 0.87 (0.64 to 1.15) | 0.64 (0.45 to 0.84) | -1.15 (-1.22 to -1.07) |
| Central Sub-Saharan Africa | 0.07 (0.05 to 0.11) | 0.14 (0.08 to 0.21) | 0.34 (0.21 to 0.50) | 0.28 (0.16 to 0.42) | -0.86 (-0.98 to -0.74) | 2.12 (1.33 to 3.24) | 3.93 (2.35 to 6) | 8.59 (5.41 to 12.80) | 6.57 (3.90 to 10.01) | -1.09 (-1.21 to -0.97) |
| East Asia | 1.84 (1.1 to 2.75) | 2.88 (1.61 to 4.45) | 0.20 (0.12 to 0.30) | 0.13 (0.07 to 0.20) | -1.69 (-1.85 to -1.53) | 58.51 (33.94 to 88.31) | 78.43 (43.21 to 122.9) | 5.97 (3.53 to 8.97) | 3.52 (1.96 to 5.54) | -2.02 (-2.20 to -1.85) |
| Eastern Europe | 0.09 (0.06 to 0.12) | 0.1 (0.07 to 0.14) | 0.03 (0.02 to 0.04) | 0.03 (0.02 to 0.04) | -0.68 (-0.83 to -0.52) | 2.54 (1.81 to 3.48) | 2.79 (1.94 to 3.82) | 0.91 (0.64 to 1.24) | 0.85 (0.59 to 1.16) | -0.58 (-0.75 to -0.41) |
| Eastern Sub-Saharan Africa | 0.51 (0.37 to 0.67) | 0.9 (0.63 to 1.15) | 0.68 (0.50 to 0.91) | 0.54 (0.38 to 0.70) | -0.83 (-0.87 to -0.80) | 15.08 (10.82 to 20.03) | 26.65 (18.8 to 34.12) | 18.28 (13.15 to 24.28) | 14.06 (9.92 to 18.00) | -0.94 (-0.98 to -0.89) |
| High-income Asia Pacific | 0.15 (0.09 to 0.22) | 0.3 (0.2 to 0.45) | 0.07 (0.05 to 0.11) | 0.06 (0.04 to 0.09) | -0.56 (-0.68 to -0.44) | 3.66 (2.28 to 5.38) | 5.77 (3.84 to 8.24) | 1.77 (1.10 to 2.60) | 1.39 (0.93 to 1.97) | -0.79 (-0.90 to -0.69) |
| High-income North America | 1.11 (0.76 to 1.52) | 1.49 (0.96 to 2.14) | 0.32 (0.22 to 0.44) | 0.23 (0.15 to 0.32) | -1.26 (-1.43 to -1.09) | 26.42 (17.74 to 36.49) | 33.85 (21.91 to 48.24) | 8.00 (5.35 to 11.03) | 5.51 (3.60 to 7.70) | -1.32 (-1.43 to -1.20) |
| North Africa and Middle East | 0.15 (0.1 to 0.2) | 0.28 (0.19 to 0.37) | 0.09 (0.06 to 0.12) | 0.06 (0.04 to 0.08) | -1.49 (-1.67 to -1.32) | 4.25 (2.85 to 5.86) | 7.89 (5.4 to 10.7) | 2.32 (1.58 to 3.15) | 1.56 (1.07 to 2.10) | -1.58 (-1.74 to -1.43) |
| Oceania | 0 (0 to 0.01) | 0.01 (0.01 to 0.02) | 0.13 (0.09 to 0.19) | 0.15 (0.10 to 0.21) | 0.47 (0.38 to 0.56) | 0.19 (0.12 to 0.28) | 0.58 (0.39 to 0.84) | 4.62 (3.01 to 6.70) | 5.34 (3.59 to 7.64) | 0.56 (0.47 to 0.65) |
| South Asia | 19.2 (15.04 to 23.22) | 46.36 (37.3 to 56.15) | 3.33 (2.59 to 4.01) | 3.19 (2.57 to 3.85) | -0.33 (-0.43 to -0.24) | 588.24 (457.64 to 713.45) | 1320.03 (1047.11 to 1609) | 90.17 (70.43 to 109.03) | 83.43 (66.74 to 101.38) | -0.44 (-0.53 to -0.35) |
| Southeast Asia | 1.37 (1.05 to 1.71) | 3.09 (2.32 to 3.92) | 0.63 (0.48 to 0.79) | 0.55 (0.41 to 0.70) | -0.56 (-0.68 to -0.44) | 34.13 (25.84 to 42.93) | 70.49 (53.05 to 89.61) | 13.56 (10.34 to 16.88) | 10.98 (8.26 to 13.93) | -0.76 (-0.88 to -0.65) |
| Southern Latin America | 0.01 (0.01 to 0.02) | 0.02 (0.01 to 0.02) | 0.03 (0.02 to 0.04) | 0.02 (0.01 to 0.02) | -1.67 (-1.86 to -1.48) | 0.32 (0.23 to 0.44) | 0.34 (0.24 to 0.45) | 0.70 (0.50 to 0.94) | 0.39 (0.28 to 0.52) | -1.80 (-1.99 to -1.61) |
| Southern Sub-Saharan Africa | 0.09 (0.06 to 0.13) | 0.21 (0.15 to 0.29) | 0.40 (0.26 to 0.58) | 0.43 (0.30 to 0.62) | 0.14 (-0.25 to 0.52) | 2.26 (1.56 to 3.01) | 4.76 (3.45 to 6.45) | 8.37 (5.74 to 11.36) | 8.57 (6.19 to 11.79) | -0.10 (-0.47 to 0.28) |
| Tropical Latin America | 0.1 (0.07 to 0.14) | 0.25 (0.17 to 0.35) | 0.12 (0.09 to 0.17) | 0.10 (0.07 to 0.14) | -0.49 (-0.67 to -0.31) | 2.41 (1.67 to 3.27) | 5.66 (3.88 to 7.7) | 2.66 (1.86 to 3.59) | 2.18 (1.50 to 2.98) | -0.50 (-0.67 to -0.34) |
| Western Europe | 0.16 (0.11 to 0.21) | 0.24 (0.17 to 0.31) | 0.03 (0.02 to 0.04) | 0.03 (0.02 to 0.03) | -0.24 (-0.33 to -0.14) | 3.89 (2.72 to 5.13) | 5.1 (3.58 to 6.54) | 0.73 (0.51 to 0.96) | 0.61 (0.43 to 0.79) | -0.47 (-0.55 to -0.40) |
| Western Sub-Saharan Africa | 0.09 (0.07 to 0.12) | 0.28 (0.19 to 0.38) | 0.11 (0.08 to 0.14) | 0.16 (0.11 to 0.21) | 1.56 (1.45 to 1.68) | 2.36 (1.76 to 3.2) | 7.53 (5.2 to 10.34) | 2.59 (1.93 to 3.48) | 3.64 (2.51 to 4.98) | 1.43 (1.32 to 1.55) |

ASDR, age-standardized Deaths rates; ASDALYR, age-standardized disability-adjusted life years rates; CI, confidence interval; DALYs, disability-adjusted life years rates; EAPC, estimated annual percentage change; UI, uncertainty interval.

Table S6 Deaths and DALYs of chewing tobacco in 1990 and 2021 at national levels

| Location | Deaths (95% UI) | | ASDR per 100,000 (95% UI) | | 1990-2021 EAPC,  (95% CI) | DALYs (95% UI) | | ASDALYR per 100,000 (95% UI) | | 1990-2021 EAPC,  (95% CI) |
| --- | --- | --- | --- | --- | --- | --- | --- | --- | --- | --- |
|  | 1990 | 2021 | 1990 | 2021 |  | 1990 | 2021 | 1990 | 2021 |  |
| Afghanistan | 17.35 (9.67 to 28.38) | 26.35 (14.13 to 41.98) | 0.24 (0.14 to 0.38) | 0.18 (0.11 to 0.29) | -1.10 (-1.23 to -0.98) | 17.35 (9.67 to 28.38) | 26.35 (14.13 to 41.98) | 0.24 (0.14 to 0.38) | 0.18 (0.11 to 0.29) | -1.10 (-1.23 to -0.98) |
| Albania | 0.66 (0.42 to 0.99) | 1.26 (0.75 to 2.10) | 0.03 (0.02 to 0.05) | 0.03 (0.02 to 0.05) | 0.53 (0.31 to 0.74) | 0.66 (0.42 to 0.99) | 1.26 (0.75 to 2.10) | 0.03 (0.02 to 0.05) | 0.03 (0.02 to 0.05) | 0.53 (0.31 to 0.74) |
| Algeria | 8.62 (5.56 to 12.68) | 19.88 (12.24 to 30.54) | 0.08 (0.05 to 0.12) | 0.06 (0.04 to 0.09) | -0.91 (-0.99 to -0.83) | 8.62 (5.56 to 12.68) | 19.88 (12.24 to 30.54) | 0.08 (0.05 to 0.12) | 0.06 (0.04 to 0.09) | -0.91 (-0.99 to -0.83) |
| American Samoa | 0.01 (0.01 to 0.02) | 0.03 (0.02 to 0.04) | 0.05 (0.03 to 0.06) | 0.06 (0.04 to 0.09) | 1.71 (1.21 to 2.21) | 0.01 (0.01 to 0.02) | 0.03 (0.02 to 0.04) | 0.05 (0.03 to 0.06) | 0.06 (0.04 to 0.09) | 1.71 (1.21 to 2.21) |
| Andorra | 0.01 (0.00 to 0.01) | 0.02 (0.01 to 0.03) | 0.01 (0.01 to 0.02) | 0.01 (0.01 to 0.02) | -0.64 (-0.95 to -0.34) | 0.01 (0.00 to 0.01) | 0.02 (0.01 to 0.03) | 0.01 (0.01 to 0.02) | 0.01 (0.01 to 0.02) | -0.64 (-0.95 to -0.34) |
| Angola | 4.69 (2.89 to 7.20) | 11.15 (6.67 to 17.08) | 0.13 (0.08 to 0.20) | 0.11 (0.06 to 0.16) | -0.78 (-0.87 to -0.70) | 4.69 (2.89 to 7.20) | 11.15 (6.67 to 17.08) | 0.13 (0.08 to 0.20) | 0.11 (0.06 to 0.16) | -0.78 (-0.87 to -0.70) |
| Antigua and Barbuda | 0.01 (0.01 to 0.02) | 0.03 (0.02 to 0.03) | 0.03 (0.02 to 0.04) | 0.02 (0.02 to 0.03) | -0.28 (-0.52 to -0.04) | 0.01 (0.01 to 0.02) | 0.03 (0.02 to 0.03) | 0.03 (0.02 to 0.04) | 0.02 (0.02 to 0.03) | -0.28 (-0.52 to -0.04) |
| Argentina | 8.18 (5.70 to 11.76) | 9.81 (6.60 to 14.10) | 0.03 (0.02 to 0.04) | 0.02 (0.01 to 0.02) | -1.10 (-1.34 to -0.86) | 8.18 (5.70 to 11.76) | 9.81 (6.60 to 14.10) | 0.03 (0.02 to 0.04) | 0.02 (0.01 to 0.02) | -1.10 (-1.34 to -0.86) |
| Armenia | 0.41 (0.28 to 0.58) | 0.46 (0.31 to 0.66) | 0.02 (0.01 to 0.02) | 0.01 (0.01 to 0.01) | -1.07 (-1.32 to -0.81) | 0.41 (0.28 to 0.58) | 0.46 (0.31 to 0.66) | 0.02 (0.01 to 0.02) | 0.01 (0.01 to 0.01) | -1.07 (-1.32 to -0.81) |
| Australia | 12.35 (7.91 to 18.06) | 24.63 (15.05 to 37.93) | 0.06 (0.04 to 0.09) | 0.05 (0.03 to 0.08) | -0.57 (-0.84 to -0.30) | 12.35 (7.91 to 18.06) | 24.63 (15.05 to 37.93) | 0.06 (0.04 to 0.09) | 0.05 (0.03 to 0.08) | -0.57 (-0.84 to -0.30) |
| Austria | 2.92 (1.89 to 4.13) | 5.41 (3.35 to 7.77) | 0.03 (0.02 to 0.04) | 0.03 (0.02 to 0.04) | 0.81 (0.70 to 0.93) | 2.92 (1.89 to 4.13) | 5.41 (3.35 to 7.77) | 0.03 (0.02 to 0.04) | 0.03 (0.02 to 0.04) | 0.81 (0.70 to 0.93) |
| Azerbaijan | 1.94 (1.25 to 2.90) | 2.71 (1.61 to 4.04) | 0.04 (0.03 to 0.06) | 0.03 (0.02 to 0.04) | -0.81 (-1.05 to -0.57) | 1.94 (1.25 to 2.90) | 2.71 (1.61 to 4.04) | 0.04 (0.03 to 0.06) | 0.03 (0.02 to 0.04) | -0.81 (-1.05 to -0.57) |
| Bahamas | 0.11 (0.08 to 0.15) | 0.25 (0.16 to 0.37) | 0.07 (0.05 to 0.09) | 0.06 (0.04 to 0.09) | -0.17 (-0.37 to 0.03) | 0.11 (0.08 to 0.15) | 0.25 (0.16 to 0.37) | 0.07 (0.05 to 0.09) | 0.06 (0.04 to 0.09) | -0.17 (-0.37 to 0.03) |
| Bahrain | 0.15 (0.10 to 0.22) | 0.40 (0.25 to 0.59) | 0.11 (0.07 to 0.16) | 0.05 (0.03 to 0.08) | -2.91 (-3.29 to -2.54) | 0.15 (0.10 to 0.22) | 0.40 (0.25 to 0.59) | 0.11 (0.07 to 0.16) | 0.05 (0.03 to 0.08) | -2.91 (-3.29 to -2.54) |
| Bangladesh | 2192.30 (1609.32 to 2837.04) | 4489.52 (3114.20 to 6350.85) | 4.71 (3.46 to 6.12) | 3.34 (2.34 to 4.70) | -1.09 (-1.22 to -0.96) | 2192.30 (1609.32 to 2837.04) | 4489.52 (3114.20 to 6350.85) | 4.71 (3.46 to 6.12) | 3.34 (2.34 to 4.70) | -1.09 (-1.22 to -0.96) |
| Barbados | 0.10 (0.07 to 0.15) | 0.15 (0.10 to 0.22) | 0.04 (0.02 to 0.05) | 0.03 (0.02 to 0.04) | -0.35 (-0.48 to -0.23) | 0.10 (0.07 to 0.15) | 0.15 (0.10 to 0.22) | 0.04 (0.02 to 0.05) | 0.03 (0.02 to 0.04) | -0.35 (-0.48 to -0.23) |
| Belarus | 4.05 (2.70 to 5.97) | 4.49 (2.77 to 6.65) | 0.03 (0.02 to 0.05) | 0.03 (0.02 to 0.04) | -0.64 (-0.88 to -0.39) | 4.05 (2.70 to 5.97) | 4.49 (2.77 to 6.65) | 0.03 (0.02 to 0.05) | 0.03 (0.02 to 0.04) | -0.64 (-0.88 to -0.39) |
| Belgium | 3.08 (2.07 to 4.42) | 4.79 (3.31 to 6.81) | 0.02 (0.01 to 0.03) | 0.02 (0.01 to 0.03) | -0.01 (-0.17 to 0.16) | 3.08 (2.07 to 4.42) | 4.79 (3.31 to 6.81) | 0.02 (0.01 to 0.03) | 0.02 (0.01 to 0.03) | -0.01 (-0.17 to 0.16) |
| Belize | 0.02 (0.01 to 0.02) | 0.06 (0.04 to 0.08) | 0.02 (0.01 to 0.03) | 0.02 (0.01 to 0.03) | 0.31 (-0.11 to 0.72) | 0.02 (0.01 to 0.02) | 0.06 (0.04 to 0.08) | 0.02 (0.01 to 0.03) | 0.02 (0.01 to 0.03) | 0.31 (-0.11 to 0.72) |
| Benin | 3.10 (1.96 to 4.56) | 10.72 (6.58 to 16.35) | 0.17 (0.11 to 0.25) | 0.24 (0.15 to 0.38) | 1.43 (1.31 to 1.55) | 3.10 (1.96 to 4.56) | 10.72 (6.58 to 16.35) | 0.17 (0.11 to 0.25) | 0.24 (0.15 to 0.38) | 1.43 (1.31 to 1.55) |
| Bermuda | 0.04 (0.03 to 0.05) | 0.04 (0.03 to 0.07) | 0.06 (0.04 to 0.09) | 0.03 (0.02 to 0.05) | -1.75 (-2.04 to -1.45) | 0.04 (0.03 to 0.05) | 0.04 (0.03 to 0.07) | 0.06 (0.04 to 0.09) | 0.03 (0.02 to 0.05) | -1.75 (-2.04 to -1.45) |
| Bhutan | 8.54 (5.83 to 11.50) | 17.48 (12.51 to 23.63) | 3.67 (2.58 to 4.90) | 2.97 (2.12 to 3.94) | -0.78 (-0.92 to -0.63) | 8.54 (5.83 to 11.50) | 17.48 (12.51 to 23.63) | 3.67 (2.58 to 4.90) | 2.97 (2.12 to 3.94) | -0.78 (-0.92 to -0.63) |
| Bolivia (Plurinational State of) | 0.97 (0.64 to 1.46) | 2.19 (1.31 to 3.29) | 0.03 (0.02 to 0.05) | 0.03 (0.02 to 0.04) | -0.67 (-0.71 to -0.64) | 0.97 (0.64 to 1.46) | 2.19 (1.31 to 3.29) | 0.03 (0.02 to 0.05) | 0.03 (0.02 to 0.04) | -0.67 (-0.71 to -0.64) |
| Bosnia and Herzegovina | 1.02 (0.69 to 1.46) | 1.83 (1.16 to 2.64) | 0.02 (0.02 to 0.03) | 0.03 (0.02 to 0.04) | 0.98 (0.75 to 1.21) | 1.02 (0.69 to 1.46) | 1.83 (1.16 to 2.64) | 0.02 (0.02 to 0.03) | 0.03 (0.02 to 0.04) | 0.98 (0.75 to 1.21) |
| Botswana | 6.94 (4.14 to 10.78) | 13.22 (8.86 to 18.43) | 1.30 (0.79 to 2.00) | 0.95 (0.62 to 1.33) | -0.82 (-1.11 to -0.53) | 6.94 (4.14 to 10.78) | 13.22 (8.86 to 18.43) | 1.30 (0.79 to 2.00) | 0.95 (0.62 to 1.33) | -0.82 (-1.11 to -0.53) |
| Brazil | 95.84 (66.56 to 130.41) | 236.74 (161.08 to 332.03) | 0.12 (0.08 to 0.17) | 0.10 (0.07 to 0.14) | -0.55 (-0.73 to -0.36) | 95.84 (66.56 to 130.41) | 236.74 (161.08 to 332.03) | 0.12 (0.08 to 0.17) | 0.10 (0.07 to 0.14) | -0.55 (-0.73 to -0.36) |
| Brunei Darussalam | 0.15 (0.10 to 0.21) | 0.39 (0.27 to 0.56) | 0.15 (0.10 to 0.22) | 0.12 (0.08 to 0.18) | -0.16 (-0.42 to 0.11) | 0.15 (0.10 to 0.21) | 0.39 (0.27 to 0.56) | 0.15 (0.10 to 0.22) | 0.12 (0.08 to 0.18) | -0.16 (-0.42 to 0.11) |
| Bulgaria | 4.92 (3.23 to 7.01) | 8.92 (6.11 to 12.90) | 0.04 (0.03 to 0.06) | 0.07 (0.05 to 0.10) | 1.49 (1.18 to 1.81) | 4.92 (3.23 to 7.01) | 8.92 (6.11 to 12.90) | 0.04 (0.03 to 0.06) | 0.07 (0.05 to 0.10) | 1.49 (1.18 to 1.81) |
| Burkina Faso | 14.21 (9.26 to 20.14) | 44.80 (27.97 to 63.11) | 0.33 (0.22 to 0.47) | 0.49 (0.31 to 0.70) | 1.63 (1.52 to 1.74) | 14.21 (9.26 to 20.14) | 44.80 (27.97 to 63.11) | 0.33 (0.22 to 0.47) | 0.49 (0.31 to 0.70) | 1.63 (1.52 to 1.74) |
| Burundi | 34.82 (22.65 to 49.57) | 36.95 (23.05 to 53.93) | 1.46 (0.95 to 2.08) | 0.71 (0.45 to 1.04) | -2.84 (-3.09 to -2.59) | 34.82 (22.65 to 49.57) | 36.95 (23.05 to 53.93) | 1.46 (0.95 to 2.08) | 0.71 (0.45 to 1.04) | -2.84 (-3.09 to -2.59) |
| Côte d'Ivoire | 0.98 (0.61 to 1.42) | 4.14 (2.55 to 6.04) | 0.43 (0.26 to 0.61) | 0.94 (0.58 to 1.38) | 0.48 (0.40 to 0.56) | 0.98 (0.61 to 1.42) | 4.14 (2.55 to 6.04) | 0.43 (0.26 to 0.61) | 0.94 (0.58 to 1.38) | 0.48 (0.40 to 0.56) |
| Cabo Verde | 61.03 (44.69 to 81.37) | 137.53 (96.19 to 190.57) | 1.55 (1.15 to 2.01) | 1.31 (0.93 to 1.77) | 2.12 (1.55 to 2.68) | 61.03 (44.69 to 81.37) | 137.53 (96.19 to 190.57) | 1.55 (1.15 to 2.01) | 1.31 (0.93 to 1.77) | 2.12 (1.55 to 2.68) |
| Cambodia | 9.07 (6.24 to 13.09) | 36.71 (22.84 to 56.99) | 0.25 (0.17 to 0.36) | 0.37 (0.23 to 0.57) | -0.73 (-0.81 to -0.65) | 9.07 (6.24 to 13.09) | 36.71 (22.84 to 56.99) | 0.25 (0.17 to 0.36) | 0.37 (0.23 to 0.57) | -0.73 (-0.81 to -0.65) |
| Cameroon | 77.02 (51.89 to 112.15) | 100.09 (62.65 to 155.69) | 0.24 (0.16 to 0.35) | 0.14 (0.09 to 0.21) | 1.60 (1.46 to 1.75) | 77.02 (51.89 to 112.15) | 100.09 (62.65 to 155.69) | 0.24 (0.16 to 0.35) | 0.14 (0.09 to 0.21) | 1.60 (1.46 to 1.75) |
| Canada | 3.32 (2.04 to 4.86) | 5.37 (3.21 to 8.48) | 0.32 (0.20 to 0.45) | 0.28 (0.16 to 0.45) | -1.76 (-1.83 to -1.69) | 3.32 (2.04 to 4.86) | 5.37 (3.21 to 8.48) | 0.32 (0.20 to 0.45) | 0.28 (0.16 to 0.45) | -1.76 (-1.83 to -1.69) |
| Central African Republic | 4.86 (3.17 to 7.01) | 18.53 (11.80 to 26.98) | 0.18 (0.12 to 0.27) | 0.36 (0.23 to 0.52) | -0.46 (-0.55 to -0.37) | 4.86 (3.17 to 7.01) | 18.53 (11.80 to 26.98) | 0.18 (0.12 to 0.27) | 0.36 (0.23 to 0.52) | -0.46 (-0.55 to -0.37) |
| Chad | 3.87 (2.48 to 5.53) | 4.07 (2.66 to 5.88) | 0.04 (0.03 to 0.06) | 0.02 (0.01 to 0.02) | 2.53 (2.36 to 2.71) | 3.87 (2.48 to 5.53) | 4.07 (2.66 to 5.88) | 0.04 (0.03 to 0.06) | 0.02 (0.01 to 0.02) | 2.53 (2.36 to 2.71) |
| Chile | 1817.77 (1083.81 to 2721.61) | 2812.95 (1560.13 to 4362.49) | 0.21 (0.13 to 0.31) | 0.13 (0.07 to 0.20) | -3.24 (-3.36 to -3.11) | 1817.77 (1083.81 to 2721.61) | 2812.95 (1560.13 to 4362.49) | 0.21 (0.13 to 0.31) | 0.13 (0.07 to 0.20) | -3.24 (-3.36 to -3.11) |
| China | 7.79 (5.34 to 11.13) | 13.62 (8.80 to 20.51) | 0.05 (0.03 to 0.07) | 0.02 (0.02 to 0.04) | -1.73 (-1.90 to -1.57) | 7.79 (5.34 to 11.13) | 13.62 (8.80 to 20.51) | 0.05 (0.03 to 0.07) | 0.02 (0.02 to 0.04) | -1.73 (-1.90 to -1.57) |
| Colombia | 5.72 (3.64 to 8.15) | 10.14 (6.66 to 14.75) | 2.81 (1.82 to 4.04) | 2.03 (1.33 to 2.93) | -2.42 (-2.58 to -2.26) | 5.72 (3.64 to 8.15) | 10.14 (6.66 to 14.75) | 2.81 (1.82 to 4.04) | 2.03 (1.33 to 2.93) | -2.42 (-2.58 to -2.26) |
| Comoros | 3.28 (2.01 to 5.12) | 6.35 (4.02 to 10.11) | 0.33 (0.20 to 0.53) | 0.25 (0.16 to 0.42) | -1.34 (-1.46 to -1.23) | 3.28 (2.01 to 5.12) | 6.35 (4.02 to 10.11) | 0.33 (0.20 to 0.53) | 0.25 (0.16 to 0.42) | -1.34 (-1.46 to -1.23) |
| Congo | 0.01 (0.01 to 0.02) | 0.02 (0.01 to 0.03) | 0.09 (0.06 to 0.13) | 0.09 (0.05 to 0.13) | -1.07 (-1.19 to -0.95) | 0.01 (0.01 to 0.02) | 0.02 (0.01 to 0.03) | 0.09 (0.06 to 0.13) | 0.09 (0.05 to 0.13) | -1.07 (-1.19 to -0.95) |
| Cook Islands | 0.39 (0.25 to 0.55) | 0.87 (0.56 to 1.26) | 0.02 (0.01 to 0.03) | 0.02 (0.01 to 0.02) | 0.18 (0.06 to 0.30) | 0.39 (0.25 to 0.55) | 0.87 (0.56 to 1.26) | 0.02 (0.01 to 0.03) | 0.02 (0.01 to 0.02) | 0.18 (0.06 to 0.30) |
| Costa Rica | 3.56 (2.26 to 5.42) | 11.12 (6.77 to 17.97) | 0.10 (0.06 to 0.17) | 0.12 (0.07 to 0.19) | -1.43 (-1.58 to -1.27) | 3.56 (2.26 to 5.42) | 11.12 (6.77 to 17.97) | 0.10 (0.06 to 0.17) | 0.12 (0.07 to 0.19) | -1.43 (-1.58 to -1.27) |
| Croatia | 2.06 (1.33 to 2.91) | 2.10 (1.35 to 3.14) | 0.03 (0.02 to 0.05) | 0.02 (0.02 to 0.04) | -0.95 (-1.07 to -0.83) | 2.06 (1.33 to 2.91) | 2.10 (1.35 to 3.14) | 0.03 (0.02 to 0.05) | 0.02 (0.02 to 0.04) | -0.95 (-1.07 to -0.83) |
| Cuba | 3.94 (2.64 to 5.42) | 8.46 (5.48 to 12.05) | 0.04 (0.03 to 0.05) | 0.04 (0.03 to 0.06) | 0.65 (0.48 to 0.82) | 3.94 (2.64 to 5.42) | 8.46 (5.48 to 12.05) | 0.04 (0.03 to 0.05) | 0.04 (0.03 to 0.06) | 0.65 (0.48 to 0.82) |
| Cyprus | 0.11 (0.07 to 0.15) | 0.23 (0.15 to 0.35) | 0.02 (0.01 to 0.03) | 0.01 (0.01 to 0.02) | -0.98 (-1.20 to -0.77) | 0.11 (0.07 to 0.15) | 0.23 (0.15 to 0.35) | 0.02 (0.01 to 0.03) | 0.01 (0.01 to 0.02) | -0.98 (-1.20 to -0.77) |
| Czechia | 4.95 (3.35 to 6.97) | 10.20 (6.49 to 15.01) | 0.04 (0.02 to 0.05) | 0.05 (0.03 to 0.07) | 1.26 (0.98 to 1.54) | 4.95 (3.35 to 6.97) | 10.20 (6.49 to 15.01) | 0.04 (0.02 to 0.05) | 0.05 (0.03 to 0.07) | 1.26 (0.98 to 1.54) |
| Democratic People's Republic of Korea | 6.39 (3.72 to 9.86) | 11.26 (6.46 to 17.50) | 0.04 (0.02 to 0.06) | 0.03 (0.02 to 0.05) | -0.46 (-0.55 to -0.37) | 6.39 (3.72 to 9.86) | 11.26 (6.46 to 17.50) | 0.04 (0.02 to 0.06) | 0.03 (0.02 to 0.05) | -0.46 (-0.55 to -0.37) |
| Democratic Republic of the Congo | 59.50 (36.31 to 90.86) | 111.61 (62.61 to 173.64) | 0.40 (0.25 to 0.61) | 0.34 (0.18 to 0.54) | -0.78 (-0.90 to -0.65) | 59.50 (36.31 to 90.86) | 111.61 (62.61 to 173.64) | 0.40 (0.25 to 0.61) | 0.34 (0.18 to 0.54) | -0.78 (-0.90 to -0.65) |
| Denmark | 3.80 (2.53 to 5.65) | 6.91 (4.43 to 9.94) | 0.05 (0.03 to 0.07) | 0.06 (0.04 to 0.08) | 0.32 (0.07 to 0.56) | 3.80 (2.53 to 5.65) | 6.91 (4.43 to 9.94) | 0.05 (0.03 to 0.07) | 0.06 (0.04 to 0.08) | 0.32 (0.07 to 0.56) |
| Djibouti | 0.74 (0.46 to 1.12) | 2.77 (1.64 to 4.53) | 0.55 (0.35 to 0.81) | 0.45 (0.27 to 0.73) | -0.87 (-0.98 to -0.75) | 0.74 (0.46 to 1.12) | 2.77 (1.64 to 4.53) | 0.55 (0.35 to 0.81) | 0.45 (0.27 to 0.73) | -0.87 (-0.98 to -0.75) |
| Dominica | 0.02 (0.01 to 0.03) | 0.03 (0.02 to 0.04) | 0.04 (0.02 to 0.06) | 0.04 (0.02 to 0.05) | 0.01 (-0.13 to 0.15) | 0.02 (0.01 to 0.03) | 0.03 (0.02 to 0.04) | 0.04 (0.02 to 0.06) | 0.04 (0.02 to 0.05) | 0.01 (-0.13 to 0.15) |
| Dominican Republic | 1.74 (1.16 to 2.55) | 5.42 (3.29 to 8.12) | 0.05 (0.03 to 0.07) | 0.05 (0.03 to 0.08) | 0.80 (0.64 to 0.96) | 1.74 (1.16 to 2.55) | 5.42 (3.29 to 8.12) | 0.05 (0.03 to 0.07) | 0.05 (0.03 to 0.08) | 0.80 (0.64 to 0.96) |
| Ecuador | 0.58 (0.39 to 0.80) | 1.64 (1.01 to 2.53) | 0.01 (0.01 to 0.02) | 0.01 (0.01 to 0.02) | -0.02 (-0.18 to 0.13) | 0.58 (0.39 to 0.80) | 1.64 (1.01 to 2.53) | 0.01 (0.01 to 0.02) | 0.01 (0.01 to 0.02) | -0.02 (-0.18 to 0.13) |
| Egypt | 4.75 (3.17 to 6.93) | 11.72 (7.45 to 17.16) | 0.02 (0.02 to 0.04) | 0.03 (0.02 to 0.04) | 0.53 (0.24 to 0.83) | 4.75 (3.17 to 6.93) | 11.72 (7.45 to 17.16) | 0.02 (0.02 to 0.04) | 0.03 (0.02 to 0.04) | 0.53 (0.24 to 0.83) |
| El Salvador | 0.81 (0.55 to 1.16) | 1.55 (0.97 to 2.30) | 0.03 (0.02 to 0.04) | 0.02 (0.02 to 0.04) | -0.45 (-0.56 to -0.35) | 0.81 (0.55 to 1.16) | 1.55 (0.97 to 2.30) | 0.03 (0.02 to 0.04) | 0.02 (0.02 to 0.04) | -0.45 (-0.56 to -0.35) |
| Equatorial Guinea | 0.53 (0.33 to 0.78) | 1.08 (0.63 to 1.85) | 0.29 (0.18 to 0.43) | 0.25 (0.15 to 0.42) | -0.60 (-0.75 to -0.46) | 0.53 (0.33 to 0.78) | 1.08 (0.63 to 1.85) | 0.29 (0.18 to 0.43) | 0.25 (0.15 to 0.42) | -0.60 (-0.75 to -0.46) |
| Eritrea | 10.47 (5.79 to 17.15) | 15.02 (8.31 to 24.55) | 0.77 (0.44 to 1.23) | 0.49 (0.28 to 0.79) | -1.87 (-2.07 to -1.67) | 10.47 (5.79 to 17.15) | 15.02 (8.31 to 24.55) | 0.77 (0.44 to 1.23) | 0.49 (0.28 to 0.79) | -1.87 (-2.07 to -1.67) |
| Estonia | 0.53 (0.35 to 0.75) | 0.69 (0.45 to 1.01) | 0.03 (0.02 to 0.04) | 0.03 (0.02 to 0.04) | -0.09 (-0.45 to 0.27) | 0.53 (0.35 to 0.75) | 0.69 (0.45 to 1.01) | 0.03 (0.02 to 0.04) | 0.03 (0.02 to 0.04) | -0.09 (-0.45 to 0.27) |
| Eswatini | 1.14 (0.67 to 1.70) | 2.50 (1.49 to 3.99) | 0.43 (0.26 to 0.64) | 0.48 (0.29 to 0.74) | 0.63 (0.12 to 1.14) | 1.14 (0.67 to 1.70) | 2.50 (1.49 to 3.99) | 0.43 (0.26 to 0.64) | 0.48 (0.29 to 0.74) | 0.63 (0.12 to 1.14) |
| Ethiopia | 68.67 (45.71 to 99.76) | 87.99 (55.70 to 132.87) | 0.37 (0.24 to 0.54) | 0.23 (0.14 to 0.35) | -1.80 (-1.92 to -1.67) | 68.67 (45.71 to 99.76) | 87.99 (55.70 to 132.87) | 0.37 (0.24 to 0.54) | 0.23 (0.14 to 0.35) | -1.80 (-1.92 to -1.67) |
| Fiji | 0.53 (0.36 to 0.76) | 1.05 (0.68 to 1.57) | 0.12 (0.08 to 0.17) | 0.13 (0.08 to 0.19) | 0.33 (0.19 to 0.48) | 0.53 (0.36 to 0.76) | 1.05 (0.68 to 1.57) | 0.12 (0.08 to 0.17) | 0.13 (0.08 to 0.19) | 0.33 (0.19 to 0.48) |
| Finland | 1.37 (0.94 to 1.88) | 2.46 (1.70 to 3.52) | 0.02 (0.01 to 0.03) | 0.02 (0.01 to 0.03) | 0.33 (0.18 to 0.48) | 1.37 (0.94 to 1.88) | 2.46 (1.70 to 3.52) | 0.02 (0.01 to 0.03) | 0.02 (0.01 to 0.03) | 0.33 (0.18 to 0.48) |
| France | 29.04 (18.69 to 43.04) | 27.02 (17.69 to 38.99) | 0.04 (0.02 to 0.05) | 0.02 (0.01 to 0.03) | -2.14 (-2.26 to -2.01) | 29.04 (18.69 to 43.04) | 27.02 (17.69 to 38.99) | 0.04 (0.02 to 0.05) | 0.02 (0.01 to 0.03) | -2.14 (-2.26 to -2.01) |
| Gabon | 0.79 (0.50 to 1.21) | 1.22 (0.79 to 1.88) | 0.15 (0.09 to 0.25) | 0.14 (0.09 to 0.21) | -0.53 (-0.68 to -0.38) | 0.79 (0.50 to 1.21) | 1.22 (0.79 to 1.88) | 0.15 (0.09 to 0.25) | 0.14 (0.09 to 0.21) | -0.53 (-0.68 to -0.38) |
| Gambia | 0.30 (0.19 to 0.45) | 1.11 (0.67 to 1.63) | 0.10 (0.06 to 0.15) | 0.13 (0.07 to 0.19) | 0.75 (0.63 to 0.87) | 0.30 (0.19 to 0.45) | 1.11 (0.67 to 1.63) | 0.10 (0.06 to 0.15) | 0.13 (0.07 to 0.19) | 0.75 (0.63 to 0.87) |
| Georgia | 1.10 (0.74 to 1.56) | 1.15 (0.74 to 1.65) | 0.02 (0.01 to 0.02) | 0.02 (0.01 to 0.03) | 1.04 (0.51 to 1.58) | 1.10 (0.74 to 1.56) | 1.15 (0.74 to 1.65) | 0.02 (0.01 to 0.02) | 0.02 (0.01 to 0.03) | 1.04 (0.51 to 1.58) |
| Germany | 28.75 (18.90 to 40.81) | 54.09 (35.04 to 78.27) | 0.02 (0.02 to 0.03) | 0.03 (0.02 to 0.04) | 0.65 (0.44 to 0.87) | 28.75 (18.90 to 40.81) | 54.09 (35.04 to 78.27) | 0.02 (0.02 to 0.03) | 0.03 (0.02 to 0.04) | 0.65 (0.44 to 0.87) |
| Ghana | 3.75 (2.39 to 5.50) | 13.27 (7.89 to 19.52) | 0.06 (0.04 to 0.09) | 0.09 (0.05 to 0.13) | 1.32 (1.18 to 1.46) | 3.75 (2.39 to 5.50) | 13.27 (7.89 to 19.52) | 0.06 (0.04 to 0.09) | 0.09 (0.05 to 0.13) | 1.32 (1.18 to 1.46) |
| Greece | 2.28 (1.47 to 3.26) | 3.66 (2.18 to 5.71) | 0.02 (0.01 to 0.02) | 0.01 (0.01 to 0.02) | -0.45 (-0.65 to -0.25) | 2.28 (1.47 to 3.26) | 3.66 (2.18 to 5.71) | 0.02 (0.01 to 0.02) | 0.01 (0.01 to 0.02) | -0.45 (-0.65 to -0.25) |
| Greenland | 0.29 (0.18 to 0.45) | 0.39 (0.24 to 0.60) | 0.84 (0.54 to 1.29) | 0.54 (0.33 to 0.82) | -1.26 (-1.32 to -1.20) | 0.29 (0.18 to 0.45) | 0.39 (0.24 to 0.60) | 0.84 (0.54 to 1.29) | 0.54 (0.33 to 0.82) | -1.26 (-1.32 to -1.20) |
| Grenada | 0.05 (0.03 to 0.07) | 0.06 (0.04 to 0.09) | 0.07 (0.05 to 0.10) | 0.05 (0.03 to 0.08) | -0.43 (-0.77 to -0.08) | 0.05 (0.03 to 0.07) | 0.06 (0.04 to 0.09) | 0.07 (0.05 to 0.10) | 0.05 (0.03 to 0.08) | -0.43 (-0.77 to -0.08) |
| Guam | 0.11 (0.07 to 0.15) | 0.33 (0.22 to 0.46) | 0.11 (0.07 to 0.15) | 0.17 (0.11 to 0.24) | 2.52 (2.16 to 2.88) | 0.11 (0.07 to 0.15) | 0.33 (0.22 to 0.46) | 0.11 (0.07 to 0.15) | 0.17 (0.11 to 0.24) | 2.52 (2.16 to 2.88) |
| Guatemala | 0.87 (0.59 to 1.27) | 2.07 (1.32 to 2.97) | 0.03 (0.02 to 0.05) | 0.02 (0.01 to 0.03) | -1.69 (-1.88 to -1.50) | 0.87 (0.59 to 1.27) | 2.07 (1.32 to 2.97) | 0.03 (0.02 to 0.05) | 0.02 (0.01 to 0.03) | -1.69 (-1.88 to -1.50) |
| Guinea | 5.14 (3.20 to 7.90) | 9.16 (5.71 to 14.59) | 0.16 (0.10 to 0.25) | 0.18 (0.11 to 0.29) | 0.34 (0.22 to 0.46) | 5.14 (3.20 to 7.90) | 9.16 (5.71 to 14.59) | 0.16 (0.10 to 0.25) | 0.18 (0.11 to 0.29) | 0.34 (0.22 to 0.46) |
| Guinea-Bissau | 0.65 (0.41 to 0.95) | 1.74 (1.11 to 2.50) | 0.17 (0.11 to 0.26) | 0.27 (0.17 to 0.40) | 1.80 (1.67 to 1.93) | 0.65 (0.41 to 0.95) | 1.74 (1.11 to 2.50) | 0.17 (0.11 to 0.26) | 0.27 (0.17 to 0.40) | 1.80 (1.67 to 1.93) |
| Guyana | 0.09 (0.06 to 0.12) | 0.14 (0.09 to 0.21) | 0.02 (0.02 to 0.03) | 0.02 (0.01 to 0.03) | 0.10 (-0.04 to 0.25) | 0.09 (0.06 to 0.12) | 0.14 (0.09 to 0.21) | 0.02 (0.02 to 0.03) | 0.02 (0.01 to 0.03) | 0.10 (-0.04 to 0.25) |
| Haiti | 2.51 (1.59 to 3.85) | 4.33 (2.58 to 6.89) | 0.08 (0.05 to 0.11) | 0.06 (0.03 to 0.09) | -0.76 (-0.85 to -0.67) | 2.51 (1.59 to 3.85) | 4.33 (2.58 to 6.89) | 0.08 (0.05 to 0.11) | 0.06 (0.03 to 0.09) | -0.76 (-0.85 to -0.67) |
| Honduras | 0.41 (0.27 to 0.60) | 1.71 (1.11 to 2.49) | 0.02 (0.01 to 0.03) | 0.03 (0.02 to 0.04) | 1.11 (0.95 to 1.27) | 0.41 (0.27 to 0.60) | 1.71 (1.11 to 2.49) | 0.02 (0.01 to 0.03) | 0.03 (0.02 to 0.04) | 1.11 (0.95 to 1.27) |
| Hungary | 5.67 (3.74 to 8.14) | 6.48 (4.23 to 9.65) | 0.04 (0.03 to 0.06) | 0.04 (0.02 to 0.05) | -0.76 (-1.00 to -0.52) | 5.67 (3.74 to 8.14) | 6.48 (4.23 to 9.65) | 0.04 (0.03 to 0.06) | 0.04 (0.02 to 0.05) | -0.76 (-1.00 to -0.52) |
| Iceland | 0.18 (0.12 to 0.27) | 0.59 (0.37 to 0.88) | 0.06 (0.04 to 0.09) | 0.10 (0.06 to 0.15) | 1.87 (1.55 to 2.19) | 0.18 (0.12 to 0.27) | 0.59 (0.37 to 0.88) | 0.06 (0.04 to 0.09) | 0.10 (0.06 to 0.15) | 1.87 (1.55 to 2.19) |
| India | 14333.78 (11108.44 to 17381.04) | 35339.78 (28136.61 to 43427.94) | 3.04 (2.37 to 3.70) | 3.00 (2.40 to 3.67) | -0.25 (-0.36 to -0.14) | 14333.78 (11108.44 to 17381.04) | 35339.78 (28136.61 to 43427.94) | 3.04 (2.37 to 3.70) | 3.00 (2.40 to 3.67) | -0.25 (-0.36 to -0.14) |
| Indonesia | 273.35 (168.48 to 401.06) | 594.54 (366.20 to 904.14) | 0.36 (0.22 to 0.53) | 0.36 (0.22 to 0.54) | -0.27 (-0.35 to -0.18) | 273.35 (168.48 to 401.06) | 594.54 (366.20 to 904.14) | 0.36 (0.22 to 0.53) | 0.36 (0.22 to 0.54) | -0.27 (-0.35 to -0.18) |
| Iran (Islamic Republic of) | 20.04 (13.04 to 28.95) | 39.61 (26.30 to 56.97) | 0.09 (0.06 to 0.13) | 0.05 (0.04 to 0.08) | -1.80 (-1.97 to -1.63) | 20.04 (13.04 to 28.95) | 39.61 (26.30 to 56.97) | 0.09 (0.06 to 0.13) | 0.05 (0.04 to 0.08) | -1.80 (-1.97 to -1.63) |
| Iraq | 1.54 (1.00 to 2.32) | 3.74 (2.32 to 5.42) | 0.02 (0.01 to 0.03) | 0.02 (0.01 to 0.02) | -1.11 (-1.25 to -0.97) | 1.54 (1.00 to 2.32) | 3.74 (2.32 to 5.42) | 0.02 (0.01 to 0.03) | 0.02 (0.01 to 0.02) | -1.11 (-1.25 to -0.97) |
| Ireland | 1.27 (0.85 to 1.76) | 1.78 (1.09 to 2.58) | 0.03 (0.02 to 0.04) | 0.02 (0.01 to 0.03) | -0.99 (-1.12 to -0.85) | 1.27 (0.85 to 1.76) | 1.78 (1.09 to 2.58) | 0.03 (0.02 to 0.04) | 0.02 (0.01 to 0.03) | -0.99 (-1.12 to -0.85) |
| Israel | 0.69 (0.46 to 0.98) | 1.68 (1.09 to 2.47) | 0.01 (0.01 to 0.02) | 0.01 (0.01 to 0.02) | -0.40 (-0.59 to -0.21) | 0.69 (0.46 to 0.98) | 1.68 (1.09 to 2.47) | 0.01 (0.01 to 0.02) | 0.01 (0.01 to 0.02) | -0.40 (-0.59 to -0.21) |
| Italy | 20.43 (13.37 to 28.55) | 24.57 (15.95 to 36.27) | 0.02 (0.02 to 0.03) | 0.02 (0.01 to 0.02) | -1.15 (-1.26 to -1.04) | 20.43 (13.37 to 28.55) | 24.57 (15.95 to 36.27) | 0.02 (0.02 to 0.03) | 0.02 (0.01 to 0.02) | -1.15 (-1.26 to -1.04) |
| Jamaica | 0.42 (0.29 to 0.59) | 0.75 (0.46 to 1.10) | 0.02 (0.02 to 0.03) | 0.02 (0.01 to 0.03) | 0.20 (-0.12 to 0.53) | 0.42 (0.29 to 0.59) | 0.75 (0.46 to 1.10) | 0.02 (0.02 to 0.03) | 0.02 (0.01 to 0.03) | 0.20 (-0.12 to 0.53) |
| Japan | 120.89 (74.09 to 180.57) | 261.02 (162.40 to 395.55) | 0.07 (0.04 to 0.11) | 0.07 (0.04 to 0.10) | -0.13 (-0.24 to -0.02) | 120.89 (74.09 to 180.57) | 261.02 (162.40 to 395.55) | 0.07 (0.04 to 0.11) | 0.07 (0.04 to 0.10) | -0.13 (-0.24 to -0.02) |
| Jordan | 0.60 (0.40 to 0.88) | 2.11 (1.29 to 3.18) | 0.05 (0.03 to 0.07) | 0.03 (0.02 to 0.05) | -1.95 (-2.21 to -1.69) | 0.60 (0.40 to 0.88) | 2.11 (1.29 to 3.18) | 0.05 (0.03 to 0.07) | 0.03 (0.02 to 0.05) | -1.95 (-2.21 to -1.69) |
| Kazakhstan | 13.61 (8.88 to 19.23) | 7.22 (4.86 to 10.16) | 0.11 (0.07 to 0.16) | 0.04 (0.03 to 0.06) | -3.23 (-3.41 to -3.06) | 13.61 (8.88 to 19.23) | 7.22 (4.86 to 10.16) | 0.11 (0.07 to 0.16) | 0.04 (0.03 to 0.06) | -3.23 (-3.41 to -3.06) |
| Kenya | 32.42 (21.13 to 47.09) | 118.22 (80.58 to 167.95) | 0.41 (0.27 to 0.59) | 0.54 (0.36 to 0.80) | 1.34 (1.13 to 1.54) | 32.42 (21.13 to 47.09) | 118.22 (80.58 to 167.95) | 0.41 (0.27 to 0.59) | 0.54 (0.36 to 0.80) | 1.34 (1.13 to 1.54) |
| Kiribati | 0.08 (0.05 to 0.11) | 0.15 (0.09 to 0.25) | 0.17 (0.11 to 0.25) | 0.17 (0.11 to 0.28) | -0.02 (-0.09 to 0.05) | 0.08 (0.05 to 0.11) | 0.15 (0.09 to 0.25) | 0.17 (0.11 to 0.25) | 0.17 (0.11 to 0.28) | -0.02 (-0.09 to 0.05) |
| Kuwait | 0.25 (0.17 to 0.34) | 0.43 (0.27 to 0.62) | 0.05 (0.03 to 0.06) | 0.02 (0.01 to 0.02) | -2.43 (-2.95 to -1.91) | 0.25 (0.17 to 0.34) | 0.43 (0.27 to 0.62) | 0.05 (0.03 to 0.06) | 0.02 (0.01 to 0.02) | -2.43 (-2.95 to -1.91) |
| Kyrgyzstan | 22.19 (14.35 to 32.76) | 12.21 (7.87 to 17.61) | 0.71 (0.46 to 1.05) | 0.23 (0.15 to 0.33) | -3.18 (-3.41 to -2.94) | 22.19 (14.35 to 32.76) | 12.21 (7.87 to 17.61) | 0.71 (0.46 to 1.05) | 0.23 (0.15 to 0.33) | -3.18 (-3.41 to -2.94) |
| Lao People's Democratic Republic | 13.51 (8.72 to 19.81) | 22.50 (14.46 to 32.99) | 0.79 (0.54 to 1.13) | 0.61 (0.39 to 0.87) | -1.00 (-1.08 to -0.92) | 13.51 (8.72 to 19.81) | 22.50 (14.46 to 32.99) | 0.79 (0.54 to 1.13) | 0.61 (0.39 to 0.87) | -1.00 (-1.08 to -0.92) |
| Latvia | 0.77 (0.54 to 1.10) | 0.94 (0.60 to 1.35) | 0.02 (0.02 to 0.03) | 0.02 (0.02 to 0.04) | 0.35 (0.18 to 0.53) | 0.77 (0.54 to 1.10) | 0.94 (0.60 to 1.35) | 0.02 (0.02 to 0.03) | 0.02 (0.02 to 0.04) | 0.35 (0.18 to 0.53) |
| Lebanon | 0.97 (0.65 to 1.49) | 1.99 (1.31 to 2.93) | 0.05 (0.03 to 0.07) | 0.03 (0.02 to 0.05) | -1.14 (-1.27 to -1.01) | 0.97 (0.65 to 1.49) | 1.99 (1.31 to 2.93) | 0.05 (0.03 to 0.07) | 0.03 (0.02 to 0.05) | -1.14 (-1.27 to -1.01) |
| Lesotho | 3.00 (1.91 to 4.53) | 6.26 (3.84 to 9.30) | 0.40 (0.25 to 0.62) | 0.68 (0.42 to 1.02) | 2.33 (1.92 to 2.73) | 3.00 (1.91 to 4.53) | 6.26 (3.84 to 9.30) | 0.40 (0.25 to 0.62) | 0.68 (0.42 to 1.02) | 2.33 (1.92 to 2.73) |
| Liberia | 1.02 (0.66 to 1.54) | 2.92 (1.76 to 4.69) | 0.10 (0.06 to 0.14) | 0.15 (0.09 to 0.24) | 1.90 (1.72 to 2.09) | 1.02 (0.66 to 1.54) | 2.92 (1.76 to 4.69) | 0.10 (0.06 to 0.14) | 0.15 (0.09 to 0.24) | 1.90 (1.72 to 2.09) |
| Libya | 0.85 (0.54 to 1.29) | 2.30 (1.44 to 3.64) | 0.05 (0.03 to 0.07) | 0.05 (0.03 to 0.07) | -0.03 (-0.17 to 0.11) | 0.85 (0.54 to 1.29) | 2.30 (1.44 to 3.64) | 0.05 (0.03 to 0.07) | 0.05 (0.03 to 0.07) | -0.03 (-0.17 to 0.11) |
| Lithuania | 0.95 (0.65 to 1.31) | 1.42 (0.91 to 2.00) | 0.02 (0.01 to 0.03) | 0.03 (0.02 to 0.04) | 0.55 (0.38 to 0.73) | 0.95 (0.65 to 1.31) | 1.42 (0.91 to 2.00) | 0.02 (0.01 to 0.03) | 0.03 (0.02 to 0.04) | 0.55 (0.38 to 0.73) |
| Luxembourg | 0.14 (0.09 to 0.19) | 0.20 (0.13 to 0.28) | 0.03 (0.02 to 0.04) | 0.02 (0.01 to 0.03) | -1.00 (-1.10 to -0.90) | 0.14 (0.09 to 0.19) | 0.20 (0.13 to 0.28) | 0.03 (0.02 to 0.04) | 0.02 (0.01 to 0.03) | -1.00 (-1.10 to -0.90) |
| Madagascar | 146.11 (101.89 to 205.89) | 237.58 (153.85 to 346.26) | 2.65 (1.83 to 3.78) | 1.74 (1.11 to 2.51) | -1.42 (-1.48 to -1.36) | 146.11 (101.89 to 205.89) | 237.58 (153.85 to 346.26) | 2.65 (1.83 to 3.78) | 1.74 (1.11 to 2.51) | -1.42 (-1.48 to -1.36) |
| Malawi | 27.72 (17.34 to 41.27) | 56.81 (35.04 to 92.19) | 0.77 (0.49 to 1.17) | 0.85 (0.50 to 1.37) | 0.27 (-0.03 to 0.58) | 27.72 (17.34 to 41.27) | 56.81 (35.04 to 92.19) | 0.77 (0.49 to 1.17) | 0.85 (0.50 to 1.37) | 0.27 (-0.03 to 0.58) |
| Malaysia | 30.59 (20.71 to 42.49) | 104.65 (74.19 to 142.64) | 0.34 (0.23 to 0.48) | 0.38 (0.26 to 0.53) | 0.27 (0.03 to 0.51) | 30.59 (20.71 to 42.49) | 104.65 (74.19 to 142.64) | 0.34 (0.23 to 0.48) | 0.38 (0.26 to 0.53) | 0.27 (0.03 to 0.51) |
| Maldives | 0.78 (0.56 to 1.09) | 1.69 (1.17 to 2.28) | 0.95 (0.69 to 1.30) | 0.56 (0.39 to 0.77) | -2.14 (-2.30 to -1.98) | 0.78 (0.56 to 1.09) | 1.69 (1.17 to 2.28) | 0.95 (0.69 to 1.30) | 0.56 (0.39 to 0.77) | -2.14 (-2.30 to -1.98) |
| Mali | 7.31 (4.98 to 10.54) | 16.46 (10.79 to 24.08) | 0.19 (0.13 to 0.26) | 0.19 (0.12 to 0.27) | 0.35 (0.21 to 0.49) | 7.31 (4.98 to 10.54) | 16.46 (10.79 to 24.08) | 0.19 (0.13 to 0.26) | 0.19 (0.12 to 0.27) | 0.35 (0.21 to 0.49) |
| Malta | 0.07 (0.05 to 0.10) | 0.15 (0.10 to 0.22) | 0.02 (0.01 to 0.02) | 0.02 (0.01 to 0.02) | -0.62 (-0.70 to -0.54) | 0.07 (0.05 to 0.10) | 0.15 (0.10 to 0.22) | 0.02 (0.01 to 0.02) | 0.02 (0.01 to 0.02) | -0.62 (-0.70 to -0.54) |
| Marshall Islands | 0.02 (0.01 to 0.03) | 0.04 (0.03 to 0.07) | 0.09 (0.06 to 0.13) | 0.10 (0.06 to 0.15) | 0.53 (0.45 to 0.61) | 0.02 (0.01 to 0.03) | 0.04 (0.03 to 0.07) | 0.09 (0.06 to 0.13) | 0.10 (0.06 to 0.15) | 0.53 (0.45 to 0.61) |
| Mauritania | 1.45 (0.97 to 2.09) | 4.04 (2.57 to 6.26) | 0.16 (0.10 to 0.23) | 0.20 (0.13 to 0.31) | 0.91 (0.77 to 1.05) | 1.45 (0.97 to 2.09) | 4.04 (2.57 to 6.26) | 0.16 (0.10 to 0.23) | 0.20 (0.13 to 0.31) | 0.91 (0.77 to 1.05) |
| Mauritius | 4.41 (3.20 to 5.86) | 8.12 (5.88 to 10.69) | 0.74 (0.53 to 1.00) | 0.46 (0.33 to 0.62) | -1.36 (-1.74 to -0.98) | 4.41 (3.20 to 5.86) | 8.12 (5.88 to 10.69) | 0.74 (0.53 to 1.00) | 0.46 (0.33 to 0.62) | -1.36 (-1.74 to -0.98) |
| Mexico | 7.59 (5.06 to 10.49) | 17.27 (12.19 to 23.96) | 0.02 (0.01 to 0.03) | 0.01 (0.01 to 0.02) | -1.09 (-1.18 to -0.99) | 7.59 (5.06 to 10.49) | 17.27 (12.19 to 23.96) | 0.02 (0.01 to 0.03) | 0.01 (0.01 to 0.02) | -1.09 (-1.18 to -0.99) |
| Micronesia (Federated States of) | 0.07 (0.05 to 0.11) | 0.12 (0.07 to 0.18) | 0.13 (0.08 to 0.20) | 0.14 (0.09 to 0.21) | 0.14 (0.09 to 0.19) | 0.07 (0.05 to 0.11) | 0.12 (0.07 to 0.18) | 0.13 (0.08 to 0.20) | 0.14 (0.09 to 0.21) | 0.14 (0.09 to 0.19) |
| Monaco | 0.02 (0.01 to 0.02) | 0.03 (0.02 to 0.04) | 0.02 (0.01 to 0.03) | 0.03 (0.02 to 0.04) | 1.11 (0.96 to 1.26) | 0.02 (0.01 to 0.02) | 0.03 (0.02 to 0.04) | 0.02 (0.01 to 0.03) | 0.03 (0.02 to 0.04) | 1.11 (0.96 to 1.26) |
| Mongolia | 1.41 (0.89 to 2.13) | 1.84 (1.18 to 2.68) | 0.14 (0.09 to 0.22) | 0.09 (0.06 to 0.14) | -1.93 (-2.10 to -1.75) | 1.41 (0.89 to 2.13) | 1.84 (1.18 to 2.68) | 0.14 (0.09 to 0.22) | 0.09 (0.06 to 0.14) | -1.93 (-2.10 to -1.75) |
| Montenegro | 0.15 (0.10 to 0.21) | 0.31 (0.20 to 0.45) | 0.02 (0.02 to 0.03) | 0.03 (0.02 to 0.05) | 1.39 (1.18 to 1.61) | 0.15 (0.10 to 0.21) | 0.31 (0.20 to 0.45) | 0.02 (0.02 to 0.03) | 0.03 (0.02 to 0.05) | 1.39 (1.18 to 1.61) |
| Morocco | 1.82 (1.12 to 2.67) | 4.27 (2.70 to 6.47) | 0.01 (0.01 to 0.02) | 0.01 (0.01 to 0.02) | -0.10 (-0.15 to -0.05) | 1.82 (1.12 to 2.67) | 4.27 (2.70 to 6.47) | 0.01 (0.01 to 0.02) | 0.01 (0.01 to 0.02) | -0.10 (-0.15 to -0.05) |
| Mozambique | 25.10 (16.27 to 35.55) | 48.71 (30.15 to 72.57) | 0.46 (0.30 to 0.65) | 0.48 (0.29 to 0.72) | 0.44 (0.28 to 0.60) | 25.10 (16.27 to 35.55) | 48.71 (30.15 to 72.57) | 0.46 (0.30 to 0.65) | 0.48 (0.29 to 0.72) | 0.44 (0.28 to 0.60) |
| Myanmar | 266.09 (179.90 to 388.15) | 394.37 (276.28 to 559.25) | 1.15 (0.80 to 1.64) | 0.81 (0.57 to 1.18) | -1.39 (-1.49 to -1.28) | 266.09 (179.90 to 388.15) | 394.37 (276.28 to 559.25) | 1.15 (0.80 to 1.64) | 0.81 (0.57 to 1.18) | -1.39 (-1.49 to -1.28) |
| Namibia | 1.98 (1.29 to 2.87) | 4.97 (2.96 to 7.68) | 0.37 (0.23 to 0.54) | 0.43 (0.26 to 0.68) | 0.33 (0.14 to 0.53) | 1.98 (1.29 to 2.87) | 4.97 (2.96 to 7.68) | 0.37 (0.23 to 0.54) | 0.43 (0.26 to 0.68) | 0.33 (0.14 to 0.53) |
| Nauru | 0.00 (0.00 to 0.01) | 0.01 (0.00 to 0.01) | 0.08 (0.05 to 0.11) | 0.08 (0.05 to 0.13) | 0.08 (-0.01 to 0.18) | 0.00 (0.00 to 0.01) | 0.01 (0.00 to 0.01) | 0.08 (0.05 to 0.11) | 0.08 (0.05 to 0.13) | 0.08 (-0.01 to 0.18) |
| Nepal | 352.50 (248.98 to 471.92) | 771.60 (541.95 to 1032.17) | 3.87 (2.77 to 5.07) | 3.44 (2.41 to 4.54) | -0.29 (-0.56 to -0.02) | 352.50 (248.98 to 471.92) | 771.60 (541.95 to 1032.17) | 3.87 (2.77 to 5.07) | 3.44 (2.41 to 4.54) | -0.29 (-0.56 to -0.02) |
| Netherlands | 4.22 (2.85 to 5.94) | 9.23 (5.76 to 13.17) | 0.02 (0.01 to 0.03) | 0.03 (0.02 to 0.04) | 0.65 (0.46 to 0.83) | 4.22 (2.85 to 5.94) | 9.23 (5.76 to 13.17) | 0.02 (0.01 to 0.03) | 0.03 (0.02 to 0.04) | 0.65 (0.46 to 0.83) |
| New Zealand | 2.64 (1.74 to 3.80) | 4.10 (2.44 to 6.22) | 0.07 (0.04 to 0.10) | 0.05 (0.03 to 0.07) | -1.04 (-1.38 to -0.70) | 2.64 (1.74 to 3.80) | 4.10 (2.44 to 6.22) | 0.07 (0.04 to 0.10) | 0.05 (0.03 to 0.07) | -1.04 (-1.38 to -0.70) |
| Nicaragua | 0.21 (0.14 to 0.29) | 0.56 (0.37 to 0.80) | 0.01 (0.01 to 0.02) | 0.01 (0.01 to 0.02) | -0.40 (-0.60 to -0.19) | 0.21 (0.14 to 0.29) | 0.56 (0.37 to 0.80) | 0.01 (0.01 to 0.02) | 0.01 (0.01 to 0.02) | -0.40 (-0.60 to -0.19) |
| Niger | 5.70 (3.60 to 8.32) | 24.43 (14.43 to 38.34) | 0.21 (0.14 to 0.30) | 0.30 (0.18 to 0.47) | 1.72 (1.54 to 1.90) | 5.70 (3.60 to 8.32) | 24.43 (14.43 to 38.34) | 0.21 (0.14 to 0.30) | 0.30 (0.18 to 0.47) | 1.72 (1.54 to 1.90) |
| Nigeria | 19.08 (12.36 to 28.67) | 53.53 (31.23 to 81.09) | 0.05 (0.03 to 0.07) | 0.06 (0.04 to 0.10) | 1.35 (1.18 to 1.53) | 19.08 (12.36 to 28.67) | 53.53 (31.23 to 81.09) | 0.05 (0.03 to 0.07) | 0.06 (0.04 to 0.10) | 1.35 (1.18 to 1.53) |
| Niue | 0.00 (0.00 to 0.00) | 0.00 (0.00 to 0.00) | 0.04 (0.03 to 0.06) | 0.05 (0.03 to 0.07) | 0.20 (0.17 to 0.24) | 0.00 (0.00 to 0.00) | 0.00 (0.00 to 0.00) | 0.04 (0.03 to 0.06) | 0.05 (0.03 to 0.07) | 0.20 (0.17 to 0.24) |
| North Macedonia | 0.36 (0.24 to 0.51) | 0.78 (0.49 to 1.12) | 0.02 (0.01 to 0.03) | 0.03 (0.02 to 0.04) | 1.04 (0.85 to 1.22) | 0.36 (0.24 to 0.51) | 0.78 (0.49 to 1.12) | 0.02 (0.01 to 0.03) | 0.03 (0.02 to 0.04) | 1.04 (0.85 to 1.22) |
| Northern Mariana Islands | 0.04 (0.02 to 0.06) | 0.18 (0.12 to 0.26) | 0.14 (0.09 to 0.20) | 0.32 (0.21 to 0.46) | 3.93 (3.32 to 4.53) | 0.04 (0.02 to 0.06) | 0.18 (0.12 to 0.26) | 0.14 (0.09 to 0.20) | 0.32 (0.21 to 0.46) | 3.93 (3.32 to 4.53) |
| Norway | 3.33 (2.25 to 4.76) | 10.41 (6.66 to 14.49) | 0.05 (0.03 to 0.07) | 0.10 (0.07 to 0.14) | 2.82 (2.36 to 3.28) | 3.33 (2.25 to 4.76) | 10.41 (6.66 to 14.49) | 0.05 (0.03 to 0.07) | 0.10 (0.07 to 0.14) | 2.82 (2.36 to 3.28) |
| Oman | 0.31 (0.19 to 0.47) | 0.68 (0.42 to 1.01) | 0.04 (0.03 to 0.07) | 0.03 (0.02 to 0.05) | -0.79 (-0.97 to -0.61) | 0.31 (0.19 to 0.47) | 0.68 (0.42 to 1.01) | 0.04 (0.03 to 0.07) | 0.03 (0.02 to 0.05) | -0.79 (-0.97 to -0.61) |
| Pakistan | 2311.69 (1684.20 to 3042.78) | 5744.99 (4184.90 to 7681.30) | 4.24 (3.08 to 5.54) | 4.84 (3.61 to 6.47) | 0.14 (-0.05 to 0.34) | 2311.69 (1684.20 to 3042.78) | 5744.99 (4184.90 to 7681.30) | 4.24 (3.08 to 5.54) | 4.84 (3.61 to 6.47) | 0.14 (-0.05 to 0.34) |
| Palau | 0.36 (0.25 to 0.51) | 0.73 (0.49 to 1.04) | 3.28 (2.24 to 4.72) | 3.08 (2.06 to 4.37) | -0.06 (-0.15 to 0.04) | 0.36 (0.25 to 0.51) | 0.73 (0.49 to 1.04) | 3.28 (2.24 to 4.72) | 3.08 (2.06 to 4.37) | -0.06 (-0.15 to 0.04) |
| Palestine | 0.31 (0.19 to 0.45) | 0.51 (0.34 to 0.74) | 0.04 (0.03 to 0.06) | 0.02 (0.01 to 0.03) | -2.05 (-2.30 to -1.79) | 0.31 (0.19 to 0.45) | 0.51 (0.34 to 0.74) | 0.04 (0.03 to 0.06) | 0.02 (0.01 to 0.03) | -2.05 (-2.30 to -1.79) |
| Panama | 0.53 (0.36 to 0.74) | 1.25 (0.80 to 1.93) | 0.04 (0.03 to 0.05) | 0.03 (0.02 to 0.04) | -0.77 (-0.86 to -0.68) | 0.53 (0.36 to 0.74) | 1.25 (0.80 to 1.93) | 0.04 (0.03 to 0.05) | 0.03 (0.02 to 0.04) | -0.77 (-0.86 to -0.68) |
| Papua New Guinea | 3.02 (1.73 to 4.73) | 10.45 (6.60 to 15.71) | 0.13 (0.07 to 0.20) | 0.14 (0.09 to 0.21) | 0.46 (0.35 to 0.58) | 3.02 (1.73 to 4.73) | 10.45 (6.60 to 15.71) | 0.13 (0.07 to 0.20) | 0.14 (0.09 to 0.21) | 0.46 (0.35 to 0.58) |
| Paraguay | 4.00 (2.52 to 5.64) | 13.47 (8.54 to 19.91) | 0.19 (0.12 to 0.26) | 0.23 (0.15 to 0.35) | 0.81 (0.65 to 0.97) | 4.00 (2.52 to 5.64) | 13.47 (8.54 to 19.91) | 0.19 (0.12 to 0.26) | 0.23 (0.15 to 0.35) | 0.81 (0.65 to 0.97) |
| Peru | 2.41 (1.59 to 3.42) | 4.95 (3.06 to 7.76) | 0.02 (0.01 to 0.03) | 0.01 (0.01 to 0.02) | -1.23 (-1.43 to -1.04) | 2.41 (1.59 to 3.42) | 4.95 (3.06 to 7.76) | 0.02 (0.01 to 0.03) | 0.01 (0.01 to 0.02) | -1.23 (-1.43 to -1.04) |
| Philippines | 63.36 (41.85 to 87.22) | 169.42 (118.09 to 236.26) | 0.25 (0.16 to 0.36) | 0.23 (0.16 to 0.33) | -0.21 (-0.31 to -0.11) | 63.36 (41.85 to 87.22) | 169.42 (118.09 to 236.26) | 0.25 (0.16 to 0.36) | 0.23 (0.16 to 0.33) | -0.21 (-0.31 to -0.11) |
| Poland | 15.28 (10.18 to 21.01) | 30.74 (20.94 to 43.42) | 0.04 (0.02 to 0.05) | 0.05 (0.03 to 0.06) | 0.77 (0.60 to 0.94) | 15.28 (10.18 to 21.01) | 30.74 (20.94 to 43.42) | 0.04 (0.02 to 0.05) | 0.05 (0.03 to 0.06) | 0.77 (0.60 to 0.94) |
| Portugal | 3.33 (2.23 to 4.78) | 4.40 (2.90 to 6.40) | 0.02 (0.02 to 0.03) | 0.02 (0.01 to 0.03) | -0.85 (-0.95 to -0.76) | 3.33 (2.23 to 4.78) | 4.40 (2.90 to 6.40) | 0.02 (0.02 to 0.03) | 0.02 (0.01 to 0.03) | -0.85 (-0.95 to -0.76) |
| Puerto Rico | 1.84 (1.23 to 2.64) | 1.57 (0.97 to 2.37) | 0.05 (0.03 to 0.08) | 0.02 (0.01 to 0.03) | -2.77 (-2.92 to -2.63) | 1.84 (1.23 to 2.64) | 1.57 (0.97 to 2.37) | 0.05 (0.03 to 0.08) | 0.02 (0.01 to 0.03) | -2.77 (-2.92 to -2.63) |
| Qatar | 0.03 (0.02 to 0.05) | 0.19 (0.12 to 0.28) | 0.04 (0.02 to 0.06) | 0.02 (0.01 to 0.03) | -1.70 (-2.35 to -1.05) | 0.03 (0.02 to 0.05) | 0.19 (0.12 to 0.28) | 0.04 (0.02 to 0.06) | 0.02 (0.01 to 0.03) | -1.70 (-2.35 to -1.05) |
| Republic of Korea | 23.38 (14.58 to 36.48) | 40.27 (24.92 to 61.21) | 0.08 (0.05 to 0.12) | 0.04 (0.03 to 0.06) | -2.43 (-2.74 to -2.13) | 23.38 (14.58 to 36.48) | 40.27 (24.92 to 61.21) | 0.08 (0.05 to 0.12) | 0.04 (0.03 to 0.06) | -2.43 (-2.74 to -2.13) |
| Republic of Moldova | 1.29 (0.84 to 1.90) | 1.50 (0.92 to 2.24) | 0.03 (0.02 to 0.04) | 0.03 (0.02 to 0.04) | -0.66 (-0.93 to -0.38) | 1.29 (0.84 to 1.90) | 1.50 (0.92 to 2.24) | 0.03 (0.02 to 0.04) | 0.03 (0.02 to 0.04) | -0.66 (-0.93 to -0.38) |
| Romania | 7.81 (5.08 to 10.91) | 18.33 (12.20 to 27.39) | 0.03 (0.02 to 0.04) | 0.06 (0.04 to 0.08) | 2.10 (1.94 to 2.26) | 7.81 (5.08 to 10.91) | 18.33 (12.20 to 27.39) | 0.03 (0.02 to 0.04) | 0.06 (0.04 to 0.08) | 2.10 (1.94 to 2.26) |
| Russian Federation | 63.98 (45.15 to 89.29) | 73.25 (48.69 to 101.41) | 0.04 (0.03 to 0.05) | 0.03 (0.02 to 0.04) | -0.92 (-1.11 to -0.73) | 63.98 (45.15 to 89.29) | 73.25 (48.69 to 101.41) | 0.04 (0.03 to 0.05) | 0.03 (0.02 to 0.04) | -0.92 (-1.11 to -0.73) |
| Rwanda | 28.72 (16.87 to 44.46) | 37.43 (20.15 to 62.07) | 1.03 (0.61 to 1.60) | 0.62 (0.35 to 1.02) | -2.40 (-2.71 to -2.09) | 28.72 (16.87 to 44.46) | 37.43 (20.15 to 62.07) | 1.03 (0.61 to 1.60) | 0.62 (0.35 to 1.02) | -2.40 (-2.71 to -2.09) |
| Saint Kitts and Nevis | 0.01 (0.01 to 0.02) | 0.02 (0.01 to 0.02) | 0.03 (0.02 to 0.04) | 0.02 (0.02 to 0.03) | -0.30 (-0.56 to -0.05) | 0.01 (0.01 to 0.02) | 0.02 (0.01 to 0.02) | 0.03 (0.02 to 0.04) | 0.02 (0.02 to 0.03) | -0.30 (-0.56 to -0.05) |
| Saint Lucia | 0.06 (0.04 to 0.08) | 0.12 (0.08 to 0.18) | 0.07 (0.05 to 0.10) | 0.05 (0.03 to 0.07) | -1.13 (-1.41 to -0.84) | 0.06 (0.04 to 0.08) | 0.12 (0.08 to 0.18) | 0.07 (0.05 to 0.10) | 0.05 (0.03 to 0.07) | -1.13 (-1.41 to -0.84) |
| Saint Vincent and the Grenadines | 0.03 (0.02 to 0.04) | 0.05 (0.03 to 0.08) | 0.04 (0.03 to 0.06) | 0.04 (0.02 to 0.05) | -0.14 (-0.39 to 0.10) | 0.03 (0.02 to 0.04) | 0.05 (0.03 to 0.08) | 0.04 (0.03 to 0.06) | 0.04 (0.02 to 0.05) | -0.14 (-0.39 to 0.10) |
| Samoa | 0.03 (0.02 to 0.04) | 0.05 (0.03 to 0.08) | 0.03 (0.02 to 0.05) | 0.03 (0.02 to 0.05) | -0.11 (-0.19 to -0.03) | 0.03 (0.02 to 0.04) | 0.05 (0.03 to 0.08) | 0.03 (0.02 to 0.05) | 0.03 (0.02 to 0.05) | -0.11 (-0.19 to -0.03) |
| San Marino | 0.01 (0.00 to 0.01) | 0.01 (0.00 to 0.02) | 0.02 (0.01 to 0.03) | 0.01 (0.01 to 0.02) | -0.43 (-0.80 to -0.05) | 0.01 (0.00 to 0.01) | 0.01 (0.00 to 0.02) | 0.02 (0.01 to 0.03) | 0.01 (0.01 to 0.02) | -0.43 (-0.80 to -0.05) |
| Sao Tome and Principe | 0.02 (0.01 to 0.04) | 0.07 (0.04 to 0.10) | 0.04 (0.02 to 0.06) | 0.07 (0.04 to 0.10) | 2.18 (2.01 to 2.34) | 0.02 (0.01 to 0.04) | 0.07 (0.04 to 0.10) | 0.04 (0.02 to 0.06) | 0.07 (0.04 to 0.10) | 2.18 (2.01 to 2.34) |
| Saudi Arabia | 2.86 (1.82 to 4.27) | 8.14 (5.26 to 11.82) | 0.05 (0.03 to 0.08) | 0.04 (0.03 to 0.06) | -0.99 (-1.09 to -0.90) | 2.86 (1.82 to 4.27) | 8.14 (5.26 to 11.82) | 0.05 (0.03 to 0.08) | 0.04 (0.03 to 0.06) | -0.99 (-1.09 to -0.90) |
| Senegal | 2.66 (1.64 to 4.11) | 10.05 (6.28 to 16.09) | 0.10 (0.06 to 0.15) | 0.15 (0.09 to 0.24) | 1.69 (1.58 to 1.80) | 2.66 (1.64 to 4.11) | 10.05 (6.28 to 16.09) | 0.10 (0.06 to 0.15) | 0.15 (0.09 to 0.24) | 1.69 (1.58 to 1.80) |
| Serbia | 3.44 (2.13 to 5.13) | 5.31 (3.50 to 7.54) | 0.03 (0.02 to 0.05) | 0.03 (0.02 to 0.05) | 0.18 (0.01 to 0.35) | 3.44 (2.13 to 5.13) | 5.31 (3.50 to 7.54) | 0.03 (0.02 to 0.05) | 0.03 (0.02 to 0.05) | 0.18 (0.01 to 0.35) |
| Seychelles | 0.33 (0.23 to 0.45) | 0.65 (0.44 to 0.89) | 0.60 (0.41 to 0.81) | 0.62 (0.41 to 0.84) | 0.07 (-0.29 to 0.43) | 0.33 (0.23 to 0.45) | 0.65 (0.44 to 0.89) | 0.60 (0.41 to 0.81) | 0.62 (0.41 to 0.84) | 0.07 (-0.29 to 0.43) |
| Sierra Leone | 3.23 (1.96 to 4.92) | 8.80 (5.43 to 13.34) | 0.17 (0.10 to 0.25) | 0.25 (0.15 to 0.39) | 1.76 (1.54 to 1.97) | 3.23 (1.96 to 4.92) | 8.80 (5.43 to 13.34) | 0.17 (0.10 to 0.25) | 0.25 (0.15 to 0.39) | 1.76 (1.54 to 1.97) |
| Singapore | 1.42 (0.89 to 2.07) | 3.10 (2.13 to 4.49) | 0.07 (0.04 to 0.10) | 0.04 (0.02 to 0.05) | -1.84 (-2.29 to -1.39) | 1.42 (0.89 to 2.07) | 3.10 (2.13 to 4.49) | 0.07 (0.04 to 0.10) | 0.04 (0.02 to 0.05) | -1.84 (-2.29 to -1.39) |
| Slovakia | 2.28 (1.48 to 3.17) | 2.77 (1.64 to 4.24) | 0.04 (0.03 to 0.06) | 0.03 (0.02 to 0.05) | -0.74 (-0.80 to -0.68) | 2.28 (1.48 to 3.17) | 2.77 (1.64 to 4.24) | 0.04 (0.03 to 0.06) | 0.03 (0.02 to 0.05) | -0.74 (-0.80 to -0.68) |
| Slovenia | 0.81 (0.55 to 1.14) | 1.14 (0.74 to 1.68) | 0.03 (0.02 to 0.05) | 0.03 (0.02 to 0.04) | -0.86 (-1.03 to -0.68) | 0.81 (0.55 to 1.14) | 1.14 (0.74 to 1.68) | 0.03 (0.02 to 0.05) | 0.03 (0.02 to 0.04) | -0.86 (-1.03 to -0.68) |
| Solomon Islands | 0.13 (0.07 to 0.20) | 0.42 (0.27 to 0.67) | 0.08 (0.04 to 0.12) | 0.10 (0.06 to 0.15) | 0.53 (0.46 to 0.61) | 0.13 (0.07 to 0.20) | 0.42 (0.27 to 0.67) | 0.08 (0.04 to 0.12) | 0.10 (0.06 to 0.15) | 0.53 (0.46 to 0.61) |
| Somalia | 16.32 (9.72 to 25.14) | 28.89 (16.72 to 44.74) | 0.67 (0.40 to 1.00) | 0.49 (0.28 to 0.74) | -1.17 (-1.27 to -1.07) | 16.32 (9.72 to 25.14) | 28.89 (16.72 to 44.74) | 0.67 (0.40 to 1.00) | 0.49 (0.28 to 0.74) | -1.17 (-1.27 to -1.07) |
| South Africa | 71.32 (44.87 to 105.12) | 153.67 (102.44 to 221.25) | 0.39 (0.24 to 0.59) | 0.39 (0.25 to 0.60) | -0.24 (-0.67 to 0.19) | 71.32 (44.87 to 105.12) | 153.67 (102.44 to 221.25) | 0.39 (0.24 to 0.59) | 0.39 (0.25 to 0.60) | -0.24 (-0.67 to 0.19) |
| South Sudan | 14.82 (9.17 to 22.47) | 18.77 (11.40 to 29.08) | 0.59 (0.36 to 0.88) | 0.49 (0.30 to 0.74) | -0.90 (-1.06 to -0.74) | 14.82 (9.17 to 22.47) | 18.77 (11.40 to 29.08) | 0.59 (0.36 to 0.88) | 0.49 (0.30 to 0.74) | -0.90 (-1.06 to -0.74) |
| Spain | 11.70 (8.00 to 16.20) | 15.23 (9.87 to 22.36) | 0.02 (0.01 to 0.03) | 0.02 (0.01 to 0.02) | -1.19 (-1.26 to -1.11) | 11.70 (8.00 to 16.20) | 15.23 (9.87 to 22.36) | 0.02 (0.01 to 0.03) | 0.02 (0.01 to 0.02) | -1.19 (-1.26 to -1.11) |
| Sri Lanka | 238.64 (179.12 to 304.47) | 541.96 (329.24 to 770.56) | 2.48 (1.87 to 3.16) | 2.05 (1.24 to 2.92) | -0.23 (-0.48 to 0.02) | 238.64 (179.12 to 304.47) | 541.96 (329.24 to 770.56) | 2.48 (1.87 to 3.16) | 2.05 (1.24 to 2.92) | -0.23 (-0.48 to 0.02) |
| Sudan | 13.68 (8.34 to 21.71) | 30.34 (17.16 to 51.13) | 0.15 (0.09 to 0.24) | 0.15 (0.08 to 0.26) | 0.12 (0.05 to 0.20) | 13.68 (8.34 to 21.71) | 30.34 (17.16 to 51.13) | 0.15 (0.09 to 0.24) | 0.15 (0.08 to 0.26) | 0.12 (0.05 to 0.20) |
| Suriname | 0.05 (0.04 to 0.08) | 0.12 (0.08 to 0.18) | 0.02 (0.01 to 0.03) | 0.02 (0.01 to 0.03) | -0.00 (-0.18 to 0.18) | 0.05 (0.04 to 0.08) | 0.12 (0.08 to 0.18) | 0.02 (0.01 to 0.03) | 0.02 (0.01 to 0.03) | -0.00 (-0.18 to 0.18) |
| Sweden | 13.50 (8.73 to 19.67) | 23.12 (14.36 to 34.15) | 0.10 (0.07 to 0.15) | 0.12 (0.07 to 0.17) | 0.91 (0.75 to 1.08) | 13.50 (8.73 to 19.67) | 23.12 (14.36 to 34.15) | 0.10 (0.07 to 0.15) | 0.12 (0.07 to 0.17) | 0.91 (0.75 to 1.08) |
| Switzerland | 2.69 (1.66 to 3.85) | 4.27 (2.72 to 6.03) | 0.03 (0.02 to 0.04) | 0.02 (0.01 to 0.03) | -0.17 (-0.30 to -0.04) | 2.69 (1.66 to 3.85) | 4.27 (2.72 to 6.03) | 0.03 (0.02 to 0.04) | 0.02 (0.01 to 0.03) | -0.17 (-0.30 to -0.04) |
| Syrian Arab Republic | 0.33 (0.20 to 0.48) | 0.88 (0.55 to 1.37) | 0.01 (0.00 to 0.01) | 0.01 (0.00 to 0.01) | 0.23 (0.09 to 0.36) | 0.33 (0.20 to 0.48) | 0.88 (0.55 to 1.37) | 0.01 (0.00 to 0.01) | 0.01 (0.00 to 0.01) | 0.23 (0.09 to 0.36) |
| Taiwan (Province of China) | 16.99 (10.78 to 24.02) | 52.19 (33.72 to 74.82) | 0.10 (0.07 to 0.15) | 0.13 (0.08 to 0.18) | 0.76 (0.33 to 1.19) | 16.99 (10.78 to 24.02) | 52.19 (33.72 to 74.82) | 0.10 (0.07 to 0.15) | 0.13 (0.08 to 0.18) | 0.76 (0.33 to 1.19) |
| Tajikistan | 9.97 (5.99 to 15.31) | 12.39 (6.82 to 20.69) | 0.36 (0.22 to 0.55) | 0.21 (0.12 to 0.35) | -1.68 (-1.85 to -1.52) | 9.97 (5.99 to 15.31) | 12.39 (6.82 to 20.69) | 0.36 (0.22 to 0.55) | 0.21 (0.12 to 0.35) | -1.68 (-1.85 to -1.52) |
| Thailand | 281.99 (196.44 to 373.27) | 817.73 (564.94 to 1172.83) | 1.00 (0.68 to 1.34) | 0.75 (0.52 to 1.08) | -1.21 (-1.48 to -0.94) | 281.99 (196.44 to 373.27) | 817.73 (564.94 to 1172.83) | 1.00 (0.68 to 1.34) | 0.75 (0.52 to 1.08) | -1.21 (-1.48 to -0.94) |
| Timor-Leste | 0.87 (0.53 to 1.24) | 2.60 (1.65 to 3.67) | 0.41 (0.26 to 0.58) | 0.35 (0.22 to 0.49) | -0.67 (-0.82 to -0.52) | 0.87 (0.53 to 1.24) | 2.60 (1.65 to 3.67) | 0.41 (0.26 to 0.58) | 0.35 (0.22 to 0.49) | -0.67 (-0.82 to -0.52) |
| Togo | 1.75 (1.10 to 2.65) | 8.67 (5.31 to 13.02) | 0.16 (0.10 to 0.24) | 0.25 (0.16 to 0.40) | 1.84 (1.69 to 1.99) | 1.75 (1.10 to 2.65) | 8.67 (5.31 to 13.02) | 0.16 (0.10 to 0.24) | 0.25 (0.16 to 0.40) | 1.84 (1.69 to 1.99) |
| Tokelau | 0.00 (0.00 to 0.00) | 0.00 (0.00 to 0.00) | 0.09 (0.06 to 0.14) | 0.10 (0.06 to 0.14) | 0.04 (0.00 to 0.07) | 0.00 (0.00 to 0.00) | 0.00 (0.00 to 0.00) | 0.09 (0.06 to 0.14) | 0.10 (0.06 to 0.14) | 0.04 (0.00 to 0.07) |
| Tonga | 0.05 (0.03 to 0.07) | 0.08 (0.05 to 0.11) | 0.08 (0.05 to 0.11) | 0.09 (0.06 to 0.13) | 0.53 (0.43 to 0.64) | 0.05 (0.03 to 0.07) | 0.08 (0.05 to 0.11) | 0.08 (0.05 to 0.11) | 0.09 (0.06 to 0.13) | 0.53 (0.43 to 0.64) |
| Trinidad and Tobago | 0.26 (0.18 to 0.38) | 0.46 (0.28 to 0.67) | 0.03 (0.02 to 0.05) | 0.02 (0.01 to 0.04) | -0.99 (-1.18 to -0.81) | 0.26 (0.18 to 0.38) | 0.46 (0.28 to 0.67) | 0.03 (0.02 to 0.05) | 0.02 (0.01 to 0.04) | -0.99 (-1.18 to -0.81) |
| Tunisia | 7.43 (4.73 to 10.30) | 14.88 (8.83 to 22.38) | 0.17 (0.11 to 0.23) | 0.12 (0.07 to 0.18) | -1.42 (-1.55 to -1.28) | 7.43 (4.73 to 10.30) | 14.88 (8.83 to 22.38) | 0.17 (0.11 to 0.23) | 0.12 (0.07 to 0.18) | -1.42 (-1.55 to -1.28) |
| Türkiye | 14.14 (9.32 to 20.23) | 24.34 (15.98 to 34.92) | 0.04 (0.03 to 0.06) | 0.03 (0.02 to 0.04) | -1.58 (-1.80 to -1.36) | 14.14 (9.32 to 20.23) | 24.34 (15.98 to 34.92) | 0.04 (0.03 to 0.06) | 0.03 (0.02 to 0.04) | -1.58 (-1.80 to -1.36) |
| Turkmenistan | 3.39 (2.16 to 4.86) | 2.56 (1.65 to 3.89) | 0.18 (0.11 to 0.26) | 0.06 (0.04 to 0.10) | -3.46 (-3.95 to -2.97) | 3.39 (2.16 to 4.86) | 2.56 (1.65 to 3.89) | 0.18 (0.11 to 0.26) | 0.06 (0.04 to 0.10) | -3.46 (-3.95 to -2.97) |
| Tuvalu | 0.01 (0.01 to 0.01) | 0.01 (0.01 to 0.02) | 0.11 (0.07 to 0.15) | 0.11 (0.07 to 0.16) | 0.18 (0.14 to 0.21) | 0.01 (0.01 to 0.01) | 0.01 (0.01 to 0.02) | 0.11 (0.07 to 0.15) | 0.11 (0.07 to 0.16) | 0.18 (0.14 to 0.21) |
| Uganda | 27.55 (16.99 to 39.63) | 69.88 (43.41 to 106.01) | 0.46 (0.28 to 0.68) | 0.52 (0.32 to 0.81) | 0.00 (-0.26 to 0.26) | 27.55 (16.99 to 39.63) | 69.88 (43.41 to 106.01) | 0.46 (0.28 to 0.68) | 0.52 (0.32 to 0.81) | 0.00 (-0.26 to 0.26) |
| Ukraine | 18.45 (12.27 to 27.06) | 19.27 (11.71 to 31.67) | 0.03 (0.02 to 0.04) | 0.03 (0.02 to 0.04) | -0.12 (-0.25 to 0.01) | 18.45 (12.27 to 27.06) | 19.27 (11.71 to 31.67) | 0.03 (0.02 to 0.04) | 0.03 (0.02 to 0.04) | -0.12 (-0.25 to 0.01) |
| United Arab Emirates | 0.40 (0.24 to 0.62) | 2.05 (1.24 to 3.06) | 0.09 (0.06 to 0.14) | 0.06 (0.04 to 0.09) | -0.04 (-0.58 to 0.50) | 0.40 (0.24 to 0.62) | 2.05 (1.24 to 3.06) | 0.09 (0.06 to 0.14) | 0.06 (0.04 to 0.09) | -0.04 (-0.58 to 0.50) |
| United Kingdom | 26.08 (17.37 to 36.38) | 39.36 (26.11 to 56.71) | 0.03 (0.02 to 0.04) | 0.03 (0.02 to 0.04) | -0.01 (-0.11 to 0.09) | 26.08 (17.37 to 36.38) | 39.36 (26.11 to 56.71) | 0.03 (0.02 to 0.04) | 0.03 (0.02 to 0.04) | -0.01 (-0.11 to 0.09) |
| United Republic of Tanzania | 53.04 (32.06 to 81.09) | 94.51 (57.20 to 138.62) | 0.52 (0.31 to 0.80) | 0.40 (0.24 to 0.59) | -1.07 (-1.15 to -0.98) | 53.04 (32.06 to 81.09) | 94.51 (57.20 to 138.62) | 0.52 (0.31 to 0.80) | 0.40 (0.24 to 0.59) | -1.07 (-1.15 to -0.98) |
| United States of America | 1033.93 (704.59 to 1413.65) | 1388.57 (884.80 to 2001.90) | 0.33 (0.22 to 0.45) | 0.24 (0.15 to 0.34) | -1.20 (-1.39 to -1.02) | 1033.93 (704.59 to 1413.65) | 1388.57 (884.80 to 2001.90) | 0.33 (0.22 to 0.45) | 0.24 (0.15 to 0.34) | -1.20 (-1.39 to -1.02) |
| United States Virgin Islands | 0.02 (0.01 to 0.02) | 0.02 (0.01 to 0.03) | 0.02 (0.01 to 0.03) | 0.01 (0.01 to 0.02) | -1.06 (-1.22 to -0.90) | 0.02 (0.01 to 0.02) | 0.02 (0.01 to 0.03) | 0.02 (0.01 to 0.03) | 0.01 (0.01 to 0.02) | -1.06 (-1.22 to -0.90) |
| Uruguay | 1.51 (1.00 to 2.15) | 1.60 (1.09 to 2.32) | 0.04 (0.03 to 0.06) | 0.03 (0.02 to 0.04) | -1.15 (-1.36 to -0.94) | 1.51 (1.00 to 2.15) | 1.60 (1.09 to 2.32) | 0.04 (0.03 to 0.06) | 0.03 (0.02 to 0.04) | -1.15 (-1.36 to -0.94) |
| Uzbekistan | 51.23 (31.33 to 76.19) | 43.48 (27.59 to 64.19) | 0.43 (0.26 to 0.63) | 0.15 (0.10 to 0.22) | -3.64 (-4.22 to -3.07) | 51.23 (31.33 to 76.19) | 43.48 (27.59 to 64.19) | 0.43 (0.26 to 0.63) | 0.15 (0.10 to 0.22) | -3.64 (-4.22 to -3.07) |
| Vanuatu | 0.07 (0.04 to 0.11) | 0.22 (0.14 to 0.32) | 0.09 (0.06 to 0.15) | 0.10 (0.07 to 0.15) | 0.37 (0.30 to 0.45) | 0.07 (0.04 to 0.11) | 0.22 (0.14 to 0.32) | 0.09 (0.06 to 0.15) | 0.10 (0.07 to 0.15) | 0.37 (0.30 to 0.45) |
| Venezuela (Bolivarian Republic of) | 10.39 (7.35 to 14.49) | 25.53 (16.05 to 38.00) | 0.11 (0.08 to 0.15) | 0.08 (0.05 to 0.13) | -1.04 (-1.17 to -0.92) | 10.39 (7.35 to 14.49) | 25.53 (16.05 to 38.00) | 0.11 (0.08 to 0.15) | 0.08 (0.05 to 0.13) | -1.04 (-1.17 to -0.92) |
| Viet Nam | 137.50 (89.00 to 205.44) | 285.25 (183.51 to 403.34) | 0.38 (0.24 to 0.57) | 0.34 (0.21 to 0.47) | -0.81 (-1.01 to -0.60) | 137.50 (89.00 to 205.44) | 285.25 (183.51 to 403.34) | 0.38 (0.24 to 0.57) | 0.34 (0.21 to 0.47) | -0.81 (-1.01 to -0.60) |
| Yemen | 51.72 (30.18 to 78.85) | 81.63 (48.60 to 123.35) | 1.09 (0.63 to 1.63) | 0.61 (0.36 to 0.92) | -2.49 (-2.88 to -2.09) | 51.72 (30.18 to 78.85) | 81.63 (48.60 to 123.35) | 1.09 (0.63 to 1.63) | 0.61 (0.36 to 0.92) | -2.49 (-2.88 to -2.09) |
| Zambia | 14.54 (9.61 to 20.93) | 34.86 (20.46 to 58.96) | 0.56 (0.37 to 0.81) | 0.57 (0.34 to 0.95) | -0.25 (-0.42 to -0.08) | 14.54 (9.61 to 20.93) | 34.86 (20.46 to 58.96) | 0.56 (0.37 to 0.81) | 0.57 (0.34 to 0.95) | -0.25 (-0.42 to -0.08) |
| Zimbabwe | 9.63 (6.29 to 14.49) | 28.62 (17.92 to 44.55) | 0.31 (0.19 to 0.50) | 0.56 (0.33 to 0.89) | 2.48 (2.07 to 2.88) | 9.63 (6.29 to 14.49) | 28.62 (17.92 to 44.55) | 0.31 (0.19 to 0.50) | 0.56 (0.33 to 0.89) | 2.48 (2.07 to 2.88) |

ASDR, age-standardized Deaths rates; ASDALYR, age-standardized disability-adjusted life years rates; CI, confidence interval; DALYs, disability-adjusted life years rates; EAPC, estimated annual percentage change; UI, uncertainty interval.

Table S7 Deaths and DALYs of secondhand smoke in 1990 and 2021 at regional levels

| Region | Deaths (×1000, 95% UI) | | ASDR per 100,000 (95% UI) | | 1990-2021 EAPC,  (95% CI) | DALYs (×1000, 95% UI) | | ASDALYR per 100,000 (95% UI) | | 1990-2021 EAPC,  (95% CI) |
| --- | --- | --- | --- | --- | --- | --- | --- | --- | --- | --- |
|  | 1990 | 2021 | 1990 | 2021 |  | 1990 | 2021 | 1990 | 2021 |  |
| High SDI | 147.88 (83.27 to 216.51) | 102.63 (49.78 to 158.06) | 13.72 (7.71 to 20.16) | 4.76 (2.36 to 7.30) | -3.58 (-3.68 to -3.49) | 3756.99 (2072.14 to 5505.94) | 2595.39 (1290.01 to 3992.62) | 362.87 (198.80 to 533.10) | 143.58 (71.76 to 220.65) | -3.13 (-3.23 to -3.03) |
| High-middle SDI | 295.5 (169.24 to 431.28) | 336.7 (185.4 to 488.44) | 33.93 (19.58 to 49.81) | 17.57 (9.65 to 25.54) | -2.43 (-2.64 to -2.22) | 8535.36 (4554.38 to 12522.55) | 7703.39 (4078.74 to 11134.33) | 895.37 (473.16 to 1319.00) | 412.59 (216.54 to 598.58) | -2.82 (-2.99 to -2.65) |
| Middle SDI | 381.49 (192.4 to 568.4) | 483.56 (253.97 to 709.13) | 40.12 (21.20 to 59.22) | 20.50 (10.67 to 30.22) | -2.38 (-2.47 to -2.30) | 15243.36 (6991.05 to 23437.55) | 12241.49 (6468.4 to 17693.7) | 1142.79 (553.78 to 1727.77) | 481.30 (250.26 to 700.23) | -2.98 (-3.07 to -2.88) |
| Low-middle SDI | 246.15 (116.41 to 378.46) | 288.61 (153.78 to 428.06) | 33.61 (17.46 to 50.52) | 22.18 (11.69 to 33.19) | -1.31 (-1.37 to -1.24) | 13005.99 (5388.52 to 20874.62) | 9028.93 (4626.42 to 13563.78) | 1158.39 (540.40 to 1786.77) | 591.46 (304.03 to 888.13) | -2.10 (-2.16 to -2.05) |
| Low SDI | 90.42 (36.68 to 145.4) | 79.53 (37.23 to 122.62) | 26.06 (12.34 to 40.64) | 15.81 (7.65 to 24.27) | -1.64 (-1.74 to -1.54) | 5676.34 (2047.05 to 9528.98) | 3297.54 (1360.08 to 5248.53) | 963.51 (402.52 to 1531.73) | 433.38 (201.61 to 666.91) | -2.63 (-2.73 to -2.54) |
| Andean Latin America | 3.29 (1.32 to 5.35) | 2.37 (1 to 3.96) | 12.16 (5.36 to 19.35) | 4.09 (1.72 to 6.87) | -3.70 (-3.91 to -3.50) | 181.21 (66.29 to 301.94) | 71.35 (29.42 to 117.28) | 462.14 (182.22 to 752.17) | 117.09 (48.17 to 193.37) | -4.45 (-4.63 to -4.26) |
| Australasia | 2.34 (1.16 to 3.7) | 1.37 (0.61 to 2.27) | 10.36 (5.14 to 16.30) | 2.52 (1.12 to 4.17) | -4.59 (-4.69 to -4.49) | 59.8 (29.52 to 93.41) | 34.94 (15.66 to 57.14) | 266.96 (132.01 to 415.82) | 74.49 (33.59 to 120.47) | -4.10 (-4.22 to -3.98) |
| Caribbean | 4.95 (2.62 to 7.35) | 4.94 (2.54 to 7.54) | 19.56 (10.66 to 28.72) | 9.17 (4.71 to 14.04) | -2.71 (-2.87 to -2.55) | 162.51 (75.81 to 256.35) | 132.24 (64.65 to 209.77) | 537.23 (264.73 to 824.08) | 256.76 (123.85 to 409.54) | -2.51 (-2.65 to -2.36) |
| Central Asia | 19.82 (10.77 to 29.62) | 17.94 (11.17 to 25.91) | 38.46 (22.60 to 56.61) | 25.09 (15.93 to 36.33) | -1.58 (-1.87 to -1.29) | 885.32 (399.66 to 1396.6) | 541.41 (304.86 to 809.75) | 1306.19 (663.73 to 1998.16) | 640.79 (367.77 to 952.51) | -2.53 (-2.84 to -2.23) |
| Central Europe | 46.44 (28.57 to 65.23) | 29.46 (16.64 to 43.89) | 34.71 (21.44 to 48.62) | 13.14 (7.39 to 19.68) | -3.37 (-3.50 to -3.24) | 1217.23 (714.27 to 1734.17) | 656.34 (345.22 to 999.31) | 898.19 (514.69 to 1288.88) | 329.52 (171.17 to 501.77) | -3.41 (-3.52 to -3.29) |
| Central Latin America | 15.42 (7.18 to 23.94) | 16.5 (7.91 to 25.41) | 16.90 (8.40 to 25.89) | 6.85 (3.28 to 10.58) | -3.11 (-3.39 to -2.82) | 696.21 (290.17 to 1108.92) | 480.24 (219.38 to 752.62) | 516.08 (236.09 to 808.18) | 193.14 (87.53 to 301.98) | -3.27 (-3.56 to -2.98) |
| Central Sub-Saharan Africa | 7.05 (2.52 to 11.99) | 6.33 (2.71 to 10.26) | 19.08 (8.46 to 31.56) | 11.59 (5.09 to 19.07) | -1.81 (-1.90 to -1.71) | 460.26 (144.55 to 811.55) | 276.54 (112.88 to 453.84) | 707.31 (268.63 to 1197.63) | 320.72 (139.40 to 519.76) | -2.73 (-2.82 to -2.64) |
| East Asia | 374.85 (188.73 to 566.98) | 496.96 (262.8 to 743.79) | 56.42 (28.33 to 85.73) | 26.29 (14.01 to 39.57) | -2.71 (-2.81 to -2.61) | 13114 (6084.24 to 19912.6) | 10997.83 (5746.31 to 16200.41) | 1450.42 (685.01 to 2183.06) | 555.31 (288.09 to 826.28) | -3.45 (-3.58 to -3.32) |
| Eastern Europe | 74.12 (48.26 to 104.04) | 53.55 (33.97 to 76.47) | 30.00 (19.55 to 42.06) | 15.47 (9.75 to 22.05) | -2.69 (-3.35 to -2.01) | 1839.06 (1126.72 to 2586.52) | 1254.7 (769.42 to 1796.5) | 728.01 (439.80 to 1029.51) | 389.92 (237.03 to 556.72) | -2.56 (-3.22 to -1.90) |
| Eastern Sub-Saharan Africa | 25.73 (9.73 to 42.67) | 16.78 (7.36 to 27) | 18.82 (8.40 to 30.29) | 8.60 (3.94 to 13.65) | -2.76 (-2.86 to -2.66) | 1718.62 (593.34 to 2910.52) | 795.44 (312.09 to 1310.39) | 739.04 (294.98 to 1204.00) | 264.33 (116.88 to 424.93) | -3.49 (-3.57 to -3.40) |
| High-income Asia Pacific | 24.09 (12.58 to 35.98) | 17.91 (7.61 to 29.28) | 13.46 (7.05 to 20.24) | 3.28 (1.45 to 5.29) | -4.69 (-4.76 to -4.62) | 602.94 (309.74 to 902.16) | 409.14 (178.73 to 662.62) | 317.56 (162.42 to 475.08) | 104.99 (47.24 to 170.06) | -3.76 (-3.84 to -3.68) |
| High-income North America | 41.69 (22.88 to 61.27) | 26.98 (13.72 to 41.48) | 12.13 (6.66 to 17.78) | 4.19 (2.15 to 6.44) | -3.70 (-3.82 to -3.57) | 1087.61 (579.07 to 1614.61) | 766.43 (379.47 to 1180.98) | 335.19 (177.82 to 497.20) | 133.06 (66.53 to 204.60) | -3.21 (-3.32 to -3.10) |
| North Africa and Middle East | 71.67 (40.32 to 102.14) | 95.26 (55.24 to 136.96) | 40.55 (24.91 to 56.89) | 23.80 (14.08 to 34.21) | -1.73 (-1.83 to -1.63) | 3127.13 (1528.69 to 4713.44) | 2918.93 (1655.18 to 4215.01) | 1168.73 (646.72 to 1666.70) | 601.28 (338.49 to 869.08) | -2.12 (-2.20 to -2.05) |
| Oceania | 1.86 (0.77 to 2.93) | 3.42 (1.55 to 5.24) | 54.15 (25.03 to 84.46) | 45.98 (21.64 to 69.95) | -0.53 (-0.60 to -0.47) | 101.96 (39.82 to 160.57) | 154.62 (65.81 to 242.78) | 1736.98 (724.09 to 2724.96) | 1376.83 (619.73 to 2113.13) | -0.62 (-0.73 to -0.51) |
| South Asia | 222.82 (102.62 to 344.73) | 281.67 (146.96 to 418.46) | 33.58 (16.86 to 51.52) | 21.70 (11.02 to 32.48) | -1.44 (-1.55 to -1.34) | 11771.51 (4709.88 to 18802.81) | 8576.23 (4346.3 to 12913.39) | 1149.15 (518.21 to 1784.52) | 575.62 (288.75 to 869.56) | -2.22 (-2.31 to -2.13) |
| Southeast Asia | 89.97 (45.51 to 135.57) | 125.94 (66.76 to 188.47) | 32.84 (17.69 to 48.97) | 21.89 (11.56 to 32.46) | -1.30 (-1.37 to -1.23) | 4286.47 (1916.4 to 6590.8) | 3762.58 (1964.42 to 5588.27) | 1091.98 (534.14 to 1648.70) | 578.68 (297.36 to 865.92) | -2.02 (-2.06 to -1.98) |
| Southern Latin America | 9.15 (4.89 to 13.92) | 7.62 (3.51 to 12.33) | 21.14 (11.36 to 32.18) | 8.63 (3.99 to 13.96) | -2.52 (-2.64 to -2.41) | 251.85 (130.7 to 384.78) | 189.36 (86.63 to 304.5) | 544.99 (284.10 to 831.65) | 228.90 (104.35 to 367.88) | -2.52 (-2.62 to -2.43) |
| Southern Sub-Saharan Africa | 8.73 (3.71 to 13.74) | 10.32 (4.64 to 16.02) | 26.62 (12.08 to 41.41) | 18.86 (8.51 to 29.41) | -0.83 (-1.25 to -0.41) | 442.58 (172.33 to 711.84) | 375.06 (162.43 to 595.58) | 948.14 (397.92 to 1492.24) | 571.33 (251.78 to 899.70) | -1.26 (-1.67 to -0.86) |
| Tropical Latin America | 25.8 (13.66 to 38.13) | 21.23 (10.19 to 33.29) | 29.22 (15.68 to 43.08) | 8.46 (4.04 to 13.29) | -4.05 (-4.16 to -3.93) | 983.15 (469.48 to 1483.53) | 591.23 (286.5 to 929.71) | 862.56 (435.23 to 1285.52) | 231.62 (111.36 to 364.35) | -4.32 (-4.44 to -4.21) |
| Western Europe | 68.56 (37.12 to 100.3) | 34.17 (15.81 to 54.84) | 12.27 (6.62 to 18.00) | 3.44 (1.57 to 5.51) | -4.14 (-4.23 to -4.05) | 1639.79 (893.21 to 2406.2) | 801.52 (362.18 to 1285.65) | 315.62 (170.87 to 464.11) | 101.55 (45.57 to 161.57) | -3.71 (-3.79 to -3.63) |
| Western Sub-Saharan Africa | 24.36 (9.21 to 40.52) | 21.37 (9.32 to 34.51) | 15.41 (6.99 to 24.36) | 8.79 (4.24 to 13.80) | -1.88 (-2.05 to -1.72) | 1630.9 (542.54 to 2805.93) | 1110.97 (427.22 to 1877.79) | 642.16 (251.42 to 1055.99) | 279.78 (123.50 to 452.08) | -2.71 (-2.88 to -2.54) |

ASDR, age-standardized Deaths rates; ASDALYR, age-standardized disability-adjusted life years rates; CI, confidence interval; DALYs, disability-adjusted life years rates; EAPC, estimated annual percentage change; UI, uncertainty interval.

Table S8 Deaths and DALYs of secondhand smoke in 1990 and 2021 at national levels

| Location | Deaths (95% UI) | | ASDR per 100,000 (95% UI) | | 1990-2021 EAPC,  (95% CI) | DALYs (95% UI) | | ASDALYR per 100,000 (95% UI) | | 1990-2021 EAPC,  (95% CI) |
| --- | --- | --- | --- | --- | --- | --- | --- | --- | --- | --- |
|  | 1990 | 2021 | 1990 | 2021 |  | 1990 | 2021 | 1990 | 2021 |  |
| Afghanistan | 2945.08 (1537.42 to 4372.38) | 3144.08 (1481.11 to 4963.02) | 38.10 (21.36 to 55.61) | 29.35 (14.97 to 45.22) | -0.68 (-0.89 to -0.48) | 133252.64 (59329.20 to 216941.85) | 137328.88 (61606.06 to 222472.58) | 1248.97 (639.14 to 1869.92) | 835.67 (398.78 to 1314.24) | -1.08 (-1.30 to -0.87) |
| Albania | 707.93 (384.97 to 1047.52) | 857.77 (498.53 to 1226.60) | 35.44 (20.20 to 51.57) | 21.52 (12.54 to 30.79) | -1.43 (-1.71 to -1.15) | 27152.10 (12606.50 to 42812.63) | 16768.51 (9689.42 to 23976.33) | 997.33 (506.20 to 1528.64) | 431.25 (245.06 to 621.11) | -2.68 (-3.01 to -2.35) |
| Algeria | 3956.12 (2464.77 to 5641.44) | 7384.04 (4417.97 to 10947.58) | 40.19 (25.09 to 56.55) | 26.77 (15.71 to 39.37) | -1.13 (-1.24 to -1.03) | 147130.34 (81562.91 to 213944.74) | 208008.22 (118676.15 to 309720.51) | 956.32 (588.27 to 1349.26) | 591.65 (336.64 to 872.26) | -1.53 (-1.65 to -1.40) |
| American Samoa | 7.63 (3.86 to 11.67) | 13.68 (6.51 to 22.13) | 38.17 (19.10 to 58.76) | 32.72 (15.64 to 52.74) | -0.62 (-0.71 to -0.52) | 291.45 (139.89 to 450.21) | 436.08 (203.19 to 701.38) | 980.34 (498.78 to 1508.84) | 910.45 (418.64 to 1465.40) | -0.35 (-0.44 to -0.27) |
| Andorra | 4.77 (1.85 to 8.44) | 5.29 (2.12 to 9.57) | 9.00 (3.56 to 15.75) | 3.25 (1.30 to 5.92) | -3.07 (-3.30 to -2.84) | 136.09 (53.46 to 238.25) | 144.87 (59.39 to 254.08) | 241.21 (95.09 to 423.30) | 97.53 (40.55 to 170.38) | -2.78 (-2.96 to -2.60) |
| Angola | 1069.43 (391.77 to 1848.26) | 1230.93 (550.63 to 2083.91) | 14.02 (6.29 to 22.95) | 9.66 (4.66 to 15.65) | -1.23 (-1.38 to -1.08) | 73517.37 (24140.31 to 129435.57) | 56579.44 (23388.47 to 95979.00) | 581.74 (229.97 to 985.78) | 283.71 (128.77 to 466.65) | -2.41 (-2.52 to -2.29) |
| Antigua and Barbuda | 5.46 (3.00 to 8.42) | 5.84 (2.75 to 9.28) | 9.82 (5.42 to 15.20) | 6.16 (2.88 to 9.73) | -1.73 (-1.94 to -1.52) | 130.39 (69.95 to 203.10) | 165.69 (75.40 to 268.01) | 246.39 (133.70 to 382.12) | 161.27 (73.00 to 259.24) | -1.57 (-1.75 to -1.40) |
| Argentina | 6042.43 (3149.64 to 9370.28) | 5313.10 (2369.72 to 8633.68) | 19.92 (10.44 to 30.94) | 9.48 (4.23 to 15.31) | -1.91 (-2.06 to -1.77) | 171151.37 (88245.90 to 266211.31) | 134624.48 (59326.79 to 218792.88) | 536.64 (277.26 to 833.52) | 254.55 (112.04 to 411.07) | -2.07 (-2.20 to -1.93) |
| Armenia | 1153.25 (674.22 to 1671.03) | 1092.59 (648.98 to 1599.90) | 45.73 (27.23 to 65.96) | 25.97 (15.27 to 38.06) | -2.29 (-2.49 to -2.10) | 39190.90 (19943.04 to 59310.90) | 23743.35 (13435.56 to 34681.70) | 1300.48 (690.33 to 1950.68) | 606.47 (346.10 to 894.73) | -2.75 (-2.88 to -2.62) |
| Australia | 1940.74 (938.20 to 3130.22) | 1131.44 (487.30 to 1916.30) | 10.29 (4.99 to 16.64) | 2.45 (1.04 to 4.12) | -4.64 (-4.73 to -4.55) | 48862.62 (23010.27 to 77954.94) | 28368.95 (12030.14 to 47072.89) | 260.61 (122.19 to 416.39) | 71.60 (30.95 to 118.22) | -4.13 (-4.23 to -4.03) |
| Austria | 1171.25 (637.15 to 1822.53) | 845.60 (432.32 to 1317.74) | 10.09 (5.48 to 15.82) | 4.51 (2.28 to 7.07) | -2.77 (-3.01 to -2.52) | 27880.83 (14837.62 to 43442.51) | 20536.38 (10433.92 to 32092.99) | 262.67 (138.49 to 409.13) | 128.46 (64.36 to 201.51) | -2.45 (-2.66 to -2.25) |
| Azerbaijan | 2917.57 (1610.06 to 4370.92) | 3078.77 (1919.66 to 4410.48) | 53.19 (31.33 to 77.64) | 37.61 (23.80 to 53.28) | -0.98 (-1.17 to -0.78) | 147953.30 (64090.69 to 232352.80) | 88236.52 (51932.20 to 127852.18) | 2061.54 (997.98 to 3178.08) | 966.31 (554.21 to 1417.23) | -2.47 (-2.66 to -2.28) |
| Bahamas | 14.41 (7.05 to 23.02) | 22.52 (10.66 to 36.57) | 9.37 (4.64 to 14.87) | 5.98 (2.81 to 9.73) | -1.45 (-1.53 to -1.37) | 486.08 (232.50 to 772.25) | 723.22 (327.63 to 1170.59) | 270.21 (129.94 to 430.31) | 175.17 (78.85 to 284.61) | -1.33 (-1.39 to -1.26) |
| Bahrain | 62.42 (34.97 to 90.69) | 138.63 (70.72 to 209.97) | 46.41 (26.12 to 67.93) | 24.26 (12.29 to 37.07) | -2.50 (-2.82 to -2.17) | 2143.21 (1184.91 to 3179.07) | 5005.79 (2444.74 to 7722.59) | 1014.82 (572.57 to 1495.27) | 530.43 (254.84 to 814.23) | -2.54 (-2.76 to -2.33) |
| Bangladesh | 27693.78 (12314.18 to 44418.23) | 27166.14 (14768.19 to 40922.05) | 37.50 (19.28 to 57.94) | 22.29 (12.01 to 33.42) | -1.89 (-2.15 to -1.63) | 1680649.86 (652781.13 to 2735153.74) | 809932.29 (404769.42 to 1241003.53) | 1401.72 (637.44 to 2234.66) | 588.37 (294.70 to 900.36) | -2.96 (-3.12 to -2.81) |
| Barbados | 19.07 (9.74 to 30.39) | 15.37 (6.42 to 25.54) | 6.62 (3.36 to 10.59) | 3.06 (1.27 to 5.08) | -2.79 (-3.05 to -2.53) | 458.30 (225.29 to 725.06) | 407.15 (165.50 to 681.43) | 171.91 (84.59 to 274.19) | 87.70 (35.31 to 146.54) | -2.48 (-2.71 to -2.26) |
| Belarus | 3055.11 (1691.35 to 4515.95) | 2967.60 (1683.02 to 4463.25) | 25.11 (13.81 to 37.27) | 18.60 (10.53 to 28.01) | -1.34 (-1.57 to -1.11) | 75753.52 (41989.98 to 112981.63) | 65718.47 (37331.65 to 98035.46) | 633.25 (346.75 to 946.71) | 438.59 (248.54 to 656.83) | -1.68 (-1.97 to -1.39) |
| Belgium | 1931.18 (959.95 to 3021.73) | 881.26 (371.33 to 1503.93) | 12.99 (6.46 to 20.11) | 3.63 (1.50 to 6.16) | -4.03 (-4.18 to -3.88) | 45978.59 (22368.80 to 71336.00) | 21203.66 (8598.74 to 35936.16) | 333.18 (160.82 to 512.11) | 107.17 (43.36 to 180.32) | -3.64 (-3.76 to -3.51) |
| Belize | 11.13 (5.18 to 17.67) | 17.66 (8.04 to 28.86) | 10.00 (4.91 to 15.80) | 6.22 (2.80 to 10.27) | -1.79 (-2.18 to -1.39) | 457.21 (192.08 to 735.65) | 589.79 (254.74 to 972.71) | 305.94 (142.36 to 486.26) | 178.95 (77.83 to 297.15) | -1.88 (-2.15 to -1.60) |
| Benin | 600.18 (207.68 to 1015.61) | 408.66 (159.71 to 663.11) | 15.41 (6.62 to 24.50) | 6.41 (2.75 to 10.32) | -3.09 (-3.24 to -2.94) | 40069.35 (12296.77 to 70638.80) | 20952.40 (7426.51 to 35998.67) | 620.89 (229.26 to 1034.25) | 200.15 (81.39 to 322.46) | -3.86 (-4.01 to -3.71) |
| Bermuda | 7.06 (3.61 to 11.80) | 6.96 (3.06 to 12.15) | 12.23 (6.21 to 20.46) | 4.74 (2.06 to 8.28) | -3.16 (-3.35 to -2.97) | 170.13 (86.60 to 283.95) | 153.49 (69.40 to 263.65) | 276.98 (140.40 to 461.52) | 121.59 (54.88 to 209.66) | -2.73 (-2.95 to -2.50) |
| Bhutan | 65.87 (26.77 to 107.97) | 72.95 (34.30 to 119.49) | 20.40 (9.85 to 32.84) | 13.03 (6.05 to 21.17) | -1.61 (-1.78 to -1.45) | 3815.52 (1250.62 to 6490.12) | 2012.16 (945.95 to 3294.85) | 692.58 (293.95 to 1126.44) | 327.30 (153.59 to 531.17) | -2.67 (-2.84 to -2.50) |
| Bolivia (Plurinational State of) | 955.77 (361.16 to 1589.47) | 613.85 (252.91 to 1051.30) | 19.69 (8.44 to 31.33) | 7.49 (3.11 to 12.87) | -2.58 (-2.92 to -2.24) | 60404.65 (21135.01 to 105200.19) | 20570.03 (8132.17 to 34665.04) | 806.56 (305.32 to 1338.96) | 209.34 (83.04 to 352.77) | -3.80 (-4.14 to -3.47) |
| Bosnia and Herzegovina | 897.61 (514.74 to 1310.12) | 1160.02 (618.06 to 1825.24) | 24.88 (14.13 to 36.64) | 18.44 (9.80 to 28.88) | -1.07 (-1.28 to -0.86) | 24693.37 (13621.32 to 36503.21) | 25499.41 (13274.81 to 40167.86) | 598.09 (331.80 to 878.74) | 426.61 (221.22 to 669.43) | -1.18 (-1.40 to -0.96) |
| Botswana | 194.32 (84.66 to 318.18) | 227.08 (96.39 to 365.05) | 33.80 (15.31 to 55.40) | 17.10 (7.43 to 27.67) | -2.17 (-2.45 to -1.89) | 8424.38 (3421.11 to 13974.69) | 8304.83 (3474.21 to 13573.42) | 942.81 (416.43 to 1542.57) | 481.68 (203.13 to 771.96) | -2.14 (-2.43 to -1.85) |
| Brazil | 25422.99 (13458.36 to 37572.75) | 20637.70 (9922.54 to 32394.23) | 29.62 (15.88 to 43.67) | 8.41 (4.02 to 13.22) | -4.11 (-4.23 to -4.00) | 968963.34 (462680.57 to 1462946.89) | 574903.76 (279610.85 to 905847.33) | 874.99 (441.01 to 1305.24) | 230.74 (111.37 to 364.44) | -4.39 (-4.50 to -4.27) |
| Brunei Darussalam | 24.95 (12.46 to 37.83) | 26.51 (11.94 to 43.55) | 25.70 (12.90 to 39.41) | 8.72 (3.88 to 14.32) | -3.01 (-3.33 to -2.68) | 846.21 (416.07 to 1293.17) | 1026.84 (464.48 to 1659.06) | 638.62 (316.90 to 977.06) | 254.12 (112.40 to 416.31) | -2.74 (-3.06 to -2.41) |
| Bulgaria | 4795.73 (3015.89 to 6657.74) | 2753.68 (1518.11 to 4096.32) | 52.83 (33.00 to 74.23) | 20.82 (11.58 to 30.68) | -3.40 (-3.68 to -3.12) | 123510.24 (75122.00 to 173053.51) | 62372.16 (32817.87 to 94083.69) | 1238.41 (737.55 to 1751.37) | 527.00 (269.12 to 789.53) | -3.18 (-3.45 to -2.92) |
| Burkina Faso | 1948.12 (661.42 to 3333.40) | 1646.77 (652.74 to 2812.08) | 23.65 (9.55 to 39.87) | 12.89 (5.52 to 21.54) | -1.88 (-1.99 to -1.78) | 135973.97 (41736.46 to 237689.71) | 93461.13 (34384.33 to 165718.38) | 984.41 (349.68 to 1662.64) | 421.62 (170.00 to 709.80) | -2.54 (-2.68 to -2.40) |
| Burundi | 764.88 (296.88 to 1272.21) | 370.55 (162.75 to 604.38) | 18.82 (8.38 to 30.41) | 6.03 (2.79 to 9.69) | -3.97 (-4.31 to -3.63) | 48136.06 (15870.57 to 83801.78) | 18201.84 (7216.61 to 30481.22) | 758.36 (318.36 to 1248.92) | 197.15 (89.38 to 323.30) | -4.51 (-4.77 to -4.25) |
| Côte d'Ivoire | 24.64 (11.26 to 39.31) | 36.31 (16.61 to 56.48) | 9.38 (4.44 to 15.16) | 8.39 (3.88 to 13.13) | -1.70 (-2.25 to -1.15) | 928.16 (373.70 to 1494.00) | 1017.51 (440.68 to 1618.44) | 297.04 (135.03 to 473.91) | 216.27 (94.95 to 341.79) | -2.08 (-2.60 to -1.56) |
| Cabo Verde | 3842.76 (1543.73 to 6093.38) | 3249.41 (1549.22 to 5057.47) | 45.85 (21.26 to 70.67) | 31.92 (15.49 to 49.52) | -0.76 (-1.13 to -0.38) | 270559.00 (100567.67 to 440821.67) | 109293.88 (48463.36 to 173883.40) | 1962.76 (809.48 to 3102.86) | 824.91 (368.29 to 1297.87) | -1.45 (-1.74 to -1.16) |
| Cambodia | 1211.52 (439.59 to 2044.63) | 1289.44 (541.72 to 2150.37) | 16.44 (7.09 to 26.75) | 8.94 (3.99 to 14.45) | -1.48 (-1.68 to -1.28) | 78101.00 (25165.04 to 136820.31) | 63194.52 (24263.78 to 110407.86) | 611.06 (232.74 to 1025.83) | 272.06 (116.57 to 451.06) | -3.22 (-3.49 to -2.95) |
| Cameroon | 4572.71 (2376.23 to 6930.81) | 2581.02 (1167.78 to 4240.52) | 14.45 (7.53 to 21.99) | 3.60 (1.64 to 5.87) | -2.05 (-2.32 to -1.78) | 116680.77 (61249.71 to 176453.90) | 71371.88 (32141.89 to 116066.18) | 374.35 (196.68 to 565.82) | 114.56 (52.49 to 185.38) | -2.65 (-2.93 to -2.36) |
| Canada | 484.09 (167.94 to 825.97) | 388.28 (162.30 to 644.27) | 23.98 (10.17 to 39.02) | 14.88 (6.81 to 24.07) | -4.68 (-4.84 to -4.52) | 32372.75 (9757.76 to 57223.27) | 20190.36 (7592.97 to 34858.81) | 964.73 (358.65 to 1584.44) | 479.55 (203.52 to 790.81) | -4.01 (-4.16 to -3.86) |
| Central African Republic | 984.61 (368.42 to 1718.07) | 1316.22 (494.07 to 2251.35) | 17.30 (7.62 to 28.72) | 12.09 (5.49 to 19.91) | -1.68 (-1.83 to -1.54) | 68100.36 (20815.04 to 121672.88) | 87004.98 (28317.81 to 151051.32) | 758.15 (298.70 to 1299.96) | 442.42 (182.49 to 750.03) | -2.40 (-2.56 to -2.23) |
| Chad | 2365.05 (1176.27 to 3524.89) | 1825.92 (875.13 to 2892.32) | 26.32 (13.12 to 39.42) | 7.02 (3.38 to 11.08) | -1.32 (-1.59 to -1.04) | 62768.50 (30208.90 to 94576.54) | 43893.00 (20838.30 to 68636.52) | 596.40 (291.91 to 898.32) | 176.74 (83.98 to 275.99) | -1.86 (-2.16 to -1.56) |
| Chile | 365894.91 (184101.29 to 554439.83) | 483037.07 (255883.10 to 724826.36) | 57.48 (28.91 to 87.48) | 26.64 (14.24 to 40.19) | -4.02 (-4.19 to -3.84) | 12833332.38 (5947015.18 to 19479864.66) | 10654491.04 (5568188.51 to 15728464.98) | 1474.50 (695.41 to 2221.79) | 558.60 (290.88 to 830.95) | -3.72 (-3.91 to -3.52) |
| China | 2493.21 (1282.16 to 3832.38) | 2711.05 (1321.09 to 4327.75) | 13.98 (7.51 to 21.30) | 4.82 (2.34 to 7.68) | -2.73 (-2.83 to -2.63) | 95872.78 (43058.73 to 152330.30) | 68691.87 (32284.59 to 109830.07) | 396.19 (195.78 to 612.43) | 127.30 (58.91 to 204.39) | -3.50 (-3.63 to -3.37) |
| Colombia | 82.30 (29.53 to 138.58) | 69.30 (29.78 to 117.15) | 27.05 (11.90 to 45.13) | 15.10 (6.50 to 25.46) | -3.82 (-4.01 to -3.64) | 5122.90 (1625.30 to 8870.27) | 2578.21 (1064.18 to 4387.34) | 988.23 (373.30 to 1661.36) | 440.41 (183.87 to 750.95) | -3.96 (-4.13 to -3.79) |
| Comoros | 243.11 (109.15 to 409.11) | 329.51 (151.69 to 529.86) | 20.65 (9.71 to 33.63) | 13.23 (6.04 to 21.17) | -2.21 (-2.35 to -2.07) | 10985.50 (4447.75 to 19043.19) | 12165.06 (5364.53 to 19532.01) | 619.24 (279.55 to 1040.75) | 357.68 (160.26 to 574.49) | -2.92 (-3.07 to -2.76) |
| Congo | 4.72 (2.16 to 7.38) | 5.43 (2.50 to 8.71) | 41.79 (19.12 to 64.88) | 21.94 (10.08 to 35.08) | -1.82 (-1.99 to -1.64) | 158.97 (70.51 to 250.56) | 145.55 (64.87 to 238.02) | 1119.20 (502.34 to 1757.07) | 610.40 (271.17 to 993.68) | -2.18 (-2.37 to -1.99) |
| Cook Islands | 191.67 (100.10 to 301.90) | 237.00 (109.13 to 398.49) | 10.92 (5.81 to 17.33) | 4.24 (1.96 to 7.14) | -2.37 (-2.53 to -2.21) | 6062.25 (2865.61 to 9278.87) | 6826.55 (3119.37 to 11300.23) | 285.77 (141.60 to 441.64) | 126.87 (57.50 to 209.90) | -2.29 (-2.44 to -2.13) |
| Costa Rica | 1608.71 (591.73 to 2676.26) | 1945.38 (807.59 to 3106.89) | 23.91 (11.12 to 37.89) | 16.02 (7.17 to 26.10) | -3.43 (-3.76 to -3.10) | 105247.94 (34993.09 to 177724.02) | 92866.40 (35212.38 to 155999.00) | 817.58 (326.88 to 1319.77) | 468.21 (197.14 to 757.32) | -2.94 (-3.24 to -2.64) |
| Croatia | 2335.99 (1357.36 to 3320.33) | 1590.08 (804.12 to 2516.03) | 44.14 (25.72 to 62.57) | 16.81 (8.44 to 26.49) | -2.98 (-3.09 to -2.87) | 53230.54 (30134.32 to 76348.38) | 30813.09 (15517.72 to 48390.19) | 932.00 (528.61 to 1333.56) | 367.57 (186.17 to 578.35) | -2.82 (-2.92 to -2.72) |
| Cuba | 2473.92 (1287.36 to 3747.28) | 2430.11 (1141.74 to 3914.50) | 25.88 (13.59 to 39.12) | 11.84 (5.53 to 19.09) | -3.00 (-3.25 to -2.75) | 56298.40 (29193.28 to 84958.48) | 51588.80 (24314.26 to 82553.35) | 562.91 (292.41 to 851.89) | 277.44 (130.54 to 441.38) | -2.69 (-2.93 to -2.45) |
| Cyprus | 141.64 (78.39 to 211.72) | 141.87 (72.61 to 218.67) | 24.51 (13.31 to 36.50) | 8.17 (4.21 to 12.63) | -3.54 (-3.70 to -3.38) | 3319.20 (1830.82 to 4947.01) | 3487.02 (1828.25 to 5357.42) | 475.02 (265.69 to 700.00) | 187.03 (98.02 to 285.90) | -3.02 (-3.08 to -2.95) |
| Czechia | 4056.18 (2232.92 to 6077.76) | 2459.83 (1237.07 to 3872.83) | 31.09 (17.29 to 46.43) | 11.09 (5.64 to 17.51) | -3.11 (-3.19 to -3.03) | 95435.53 (52244.05 to 143158.36) | 50553.68 (25129.04 to 79919.83) | 746.52 (408.22 to 1122.03) | 258.85 (129.20 to 405.71) | -3.21 (-3.33 to -3.08) |
| Democratic People's Republic of Korea | 5967.95 (3000.04 to 9243.78) | 9715.75 (4910.96 to 15062.85) | 45.41 (22.53 to 70.74) | 33.00 (16.67 to 51.42) | -1.18 (-1.36 to -0.99) | 195566.78 (91901.25 to 311054.89) | 245491.98 (124726.50 to 377945.88) | 1131.80 (552.29 to 1768.57) | 780.06 (390.96 to 1200.02) | -1.33 (-1.49 to -1.17) |
| Democratic Republic of the Congo | 5043.72 (1741.74 to 8765.55) | 4176.70 (1741.32 to 7002.29) | 19.69 (8.31 to 33.44) | 11.70 (5.16 to 19.74) | -1.88 (-1.98 to -1.78) | 332102.48 (101270.16 to 591248.48) | 179848.17 (72769.70 to 305114.21) | 720.78 (263.79 to 1236.44) | 316.79 (134.04 to 532.84) | -2.82 (-2.92 to -2.72) |
| Denmark | 1476.01 (744.24 to 2299.72) | 465.04 (195.52 to 764.89) | 18.52 (9.36 to 28.78) | 3.79 (1.61 to 6.20) | -5.53 (-5.75 to -5.32) | 33555.35 (16663.65 to 52284.51) | 10196.92 (4484.65 to 16561.46) | 462.32 (231.96 to 724.01) | 97.82 (43.84 to 159.64) | -5.37 (-5.58 to -5.17) |
| Djibouti | 52.38 (21.62 to 87.87) | 109.38 (47.82 to 181.24) | 26.36 (12.12 to 43.01) | 20.19 (8.94 to 32.97) | -0.87 (-1.04 to -0.70) | 3263.49 (1213.70 to 5704.73) | 4350.91 (1794.72 to 7318.88) | 878.67 (381.84 to 1448.77) | 529.83 (231.63 to 879.75) | -1.68 (-1.94 to -1.42) |
| Dominica | 6.54 (3.11 to 10.40) | 5.81 (2.74 to 9.69) | 11.36 (5.45 to 17.97) | 7.56 (3.54 to 12.54) | -1.36 (-1.53 to -1.19) | 162.80 (74.36 to 255.39) | 158.16 (71.60 to 262.61) | 269.72 (124.68 to 425.33) | 206.39 (92.13 to 342.49) | -0.81 (-1.01 to -0.60) |
| Dominican Republic | 442.53 (229.59 to 679.46) | 765.16 (400.06 to 1282.72) | 11.96 (6.44 to 18.29) | 7.76 (4.06 to 13.02) | -0.98 (-1.32 to -0.64) | 17896.80 (8289.66 to 28282.10) | 22243.92 (11081.46 to 36882.64) | 332.02 (170.25 to 513.91) | 216.11 (107.84 to 359.00) | -1.11 (-1.32 to -0.89) |
| Ecuador | 627.74 (283.62 to 983.74) | 628.20 (285.62 to 1022.93) | 11.27 (5.18 to 17.50) | 4.19 (1.92 to 6.78) | -2.92 (-3.12 to -2.72) | 26355.38 (11121.47 to 42665.63) | 18336.76 (8022.17 to 29777.45) | 319.22 (144.22 to 502.79) | 112.16 (49.21 to 182.60) | -3.24 (-3.42 to -3.05) |
| Egypt | 16793.29 (8905.46 to 24773.07) | 20054.87 (11299.89 to 29603.77) | 56.61 (33.78 to 81.85) | 38.07 (21.02 to 56.54) | -0.84 (-1.01 to -0.66) | 852238.59 (382645.85 to 1337114.17) | 672741.53 (366912.99 to 999214.72) | 1723.08 (911.34 to 2538.12) | 942.88 (518.47 to 1396.92) | -1.55 (-1.70 to -1.39) |
| El Salvador | 209.14 (100.64 to 329.30) | 351.40 (156.05 to 560.17) | 5.99 (3.04 to 9.29) | 5.41 (2.42 to 8.67) | -0.17 (-0.31 to -0.03) | 8974.66 (3769.71 to 14584.97) | 10037.76 (4432.61 to 15912.69) | 197.38 (92.62 to 309.20) | 162.01 (71.34 to 257.13) | -0.48 (-0.61 to -0.36) |
| Equatorial Guinea | 105.06 (40.26 to 169.93) | 86.58 (35.26 to 148.48) | 34.22 (15.25 to 54.86) | 17.79 (7.50 to 29.87) | -2.44 (-2.81 to -2.07) | 6599.17 (2220.32 to 11068.88) | 3524.70 (1310.21 to 6075.61) | 1274.65 (509.34 to 2067.04) | 469.11 (191.14 to 793.79) | -3.77 (-4.21 to -3.32) |
| Eritrea | 385.86 (146.94 to 669.88) | 400.30 (181.06 to 696.79) | 20.18 (9.06 to 34.80) | 13.32 (6.12 to 22.91) | -1.27 (-1.30 to -1.24) | 23866.21 (8070.09 to 42000.69) | 18024.60 (7371.42 to 32191.80) | 743.17 (305.82 to 1278.79) | 402.58 (182.52 to 692.29) | -1.86 (-1.92 to -1.79) |
| Estonia | 539.65 (312.68 to 810.83) | 164.20 (85.62 to 264.12) | 27.94 (16.22 to 42.07) | 5.67 (2.97 to 9.11) | -5.79 (-6.35 to -5.22) | 12615.70 (7061.43 to 19259.02) | 3514.26 (1759.94 to 5581.58) | 659.98 (369.03 to 1007.56) | 149.43 (73.84 to 242.78) | -5.49 (-6.00 to -4.97) |
| Eswatini | 80.47 (33.84 to 131.69) | 90.08 (35.62 to 153.68) | 22.48 (10.04 to 36.65) | 15.45 (6.43 to 25.77) | -0.83 (-1.44 to -0.20) | 4095.78 (1529.17 to 6940.91) | 3592.29 (1356.94 to 6222.70) | 671.13 (295.26 to 1081.94) | 468.41 (181.94 to 805.43) | -0.81 (-1.47 to -0.15) |
| Ethiopia | 5234.34 (1949.34 to 8882.97) | 2489.44 (1091.62 to 4124.09) | 15.07 (6.41 to 25.13) | 4.93 (2.23 to 8.04) | -4.05 (-4.24 to -3.85) | 328874.29 (109591.12 to 570982.19) | 113119.42 (46789.64 to 188641.15) | 596.69 (236.43 to 1006.93) | 154.80 (68.44 to 253.43) | -4.76 (-4.97 to -4.55) |
| Fiji | 133.93 (68.08 to 207.75) | 226.48 (101.60 to 368.61) | 41.46 (21.36 to 63.09) | 35.66 (15.85 to 57.66) | -0.68 (-0.81 to -0.54) | 4908.21 (2391.92 to 7671.93) | 7099.15 (3140.58 to 11604.86) | 1087.16 (543.61 to 1684.77) | 904.24 (403.65 to 1461.73) | -0.66 (-0.75 to -0.58) |
| Finland | 622.21 (326.02 to 1011.72) | 268.75 (132.08 to 445.47) | 9.11 (4.79 to 14.67) | 2.11 (1.02 to 3.48) | -4.63 (-4.71 to -4.54) | 16513.64 (8803.05 to 26520.35) | 6788.06 (3170.43 to 11308.03) | 253.19 (134.50 to 405.16) | 69.82 (32.40 to 114.68) | -4.03 (-4.12 to -3.95) |
| France | 5405.63 (2496.60 to 9025.85) | 3469.79 (1381.78 to 6195.54) | 6.82 (3.17 to 11.37) | 2.30 (0.93 to 4.08) | -3.50 (-3.64 to -3.37) | 128503.45 (59071.11 to 216205.66) | 81820.76 (33720.42 to 143695.00) | 178.09 (81.89 to 299.69) | 73.35 (30.22 to 126.83) | -2.87 (-2.99 to -2.75) |
| Gabon | 106.73 (45.02 to 175.55) | 119.09 (48.52 to 189.67) | 17.14 (7.64 to 27.58) | 12.64 (5.29 to 20.06) | -1.16 (-1.33 to -0.98) | 4682.64 (1727.79 to 8124.86) | 4227.40 (1680.77 to 6817.15) | 531.10 (220.93 to 877.36) | 341.76 (139.06 to 551.20) | -1.53 (-1.70 to -1.36) |
| Gambia | 171.93 (67.43 to 277.11) | 210.92 (98.96 to 349.27) | 32.66 (14.72 to 51.48) | 22.22 (10.39 to 36.79) | -1.31 (-1.48 to -1.15) | 10404.24 (3721.80 to 17766.35) | 7613.04 (3399.79 to 12479.87) | 1037.33 (429.31 to 1651.63) | 560.31 (268.08 to 927.34) | -2.11 (-2.29 to -1.93) |
| Georgia | 2455.65 (1380.91 to 3577.49) | 1474.85 (840.71 to 2176.37) | 43.68 (24.42 to 63.81) | 23.80 (13.52 to 35.23) | -1.95 (-2.17 to -1.74) | 74410.69 (40591.85 to 111895.36) | 33071.90 (18489.08 to 50065.65) | 1359.12 (715.55 to 2055.29) | 594.86 (331.98 to 908.17) | -2.86 (-3.09 to -2.63) |
| Germany | 13258.11 (7094.29 to 19809.10) | 6670.88 (3079.52 to 11226.78) | 10.83 (5.85 to 16.11) | 3.44 (1.60 to 5.81) | -3.68 (-3.85 to -3.51) | 330120.99 (182324.03 to 488634.75) | 163809.03 (73627.30 to 269636.34) | 291.62 (160.56 to 430.85) | 103.94 (46.92 to 170.93) | -3.30 (-3.47 to -3.13) |
| Ghana | 813.20 (364.65 to 1344.01) | 1088.06 (508.21 to 1734.38) | 9.34 (4.70 to 14.83) | 6.29 (3.04 to 9.93) | -0.88 (-1.06 to -0.70) | 44999.59 (17787.16 to 75998.97) | 44679.94 (19577.91 to 74728.14) | 332.93 (157.62 to 544.42) | 191.25 (88.03 to 311.39) | -1.21 (-1.40 to -1.01) |
| Greece | 3028.37 (1758.68 to 4396.53) | 2265.59 (1069.21 to 3602.26) | 21.23 (12.47 to 30.63) | 8.48 (3.90 to 13.42) | -3.10 (-3.40 to -2.79) | 66916.71 (37223.24 to 98006.50) | 46510.20 (20693.60 to 73630.89) | 472.63 (261.89 to 690.67) | 226.37 (101.65 to 356.68) | -2.45 (-2.69 to -2.22) |
| Greenland | 10.99 (5.12 to 17.86) | 7.05 (2.99 to 12.30) | 35.18 (16.51 to 57.70) | 10.87 (4.70 to 18.72) | -3.82 (-3.92 to -3.72) | 366.74 (174.17 to 587.68) | 214.26 (89.65 to 364.21) | 916.77 (433.43 to 1481.29) | 297.27 (125.43 to 498.46) | -3.69 (-3.78 to -3.60) |
| Grenada | 9.89 (5.00 to 15.86) | 7.64 (3.65 to 12.17) | 12.73 (6.51 to 20.17) | 7.66 (3.51 to 12.16) | -1.76 (-2.03 to -1.49) | 263.66 (128.25 to 418.74) | 223.32 (102.60 to 361.62) | 351.44 (175.90 to 550.08) | 202.26 (91.16 to 326.60) | -1.80 (-1.95 to -1.66) |
| Guam | 14.66 (7.36 to 22.25) | 24.31 (13.01 to 39.52) | 24.81 (12.54 to 37.48) | 11.61 (6.17 to 18.83) | -1.99 (-2.29 to -1.69) | 501.34 (243.67 to 765.95) | 789.05 (416.56 to 1252.58) | 584.11 (288.65 to 891.47) | 400.85 (210.33 to 633.56) | -0.89 (-1.07 to -0.71) |
| Guatemala | 803.02 (304.06 to 1324.13) | 885.07 (370.99 to 1442.66) | 17.33 (6.98 to 27.83) | 8.29 (3.48 to 13.46) | -2.51 (-2.72 to -2.30) | 47425.48 (16847.50 to 79822.71) | 33539.93 (12831.22 to 56058.17) | 554.47 (215.13 to 905.79) | 267.88 (105.24 to 445.07) | -2.28 (-2.47 to -2.10) |
| Guinea | 1551.71 (533.93 to 2616.69) | 1107.84 (441.55 to 1839.18) | 26.23 (10.35 to 42.10) | 16.09 (7.04 to 26.09) | -1.47 (-1.57 to -1.38) | 104778.94 (31842.31 to 184866.35) | 54013.73 (19465.10 to 92789.32) | 1170.18 (404.14 to 1958.38) | 499.11 (203.50 to 824.24) | -2.58 (-2.68 to -2.48) |
| Guinea-Bissau | 91.02 (37.01 to 161.06) | 108.68 (49.10 to 178.03) | 15.67 (7.13 to 26.56) | 14.75 (6.83 to 23.76) | 0.25 (0.07 to 0.44) | 5210.86 (1826.56 to 9422.61) | 4537.43 (1935.79 to 7462.57) | 548.70 (236.86 to 958.56) | 406.84 (185.04 to 660.37) | -0.57 (-0.72 to -0.41) |
| Guyana | 90.69 (47.43 to 136.56) | 64.73 (30.59 to 105.79) | 23.33 (12.63 to 34.91) | 10.95 (5.17 to 17.70) | -2.46 (-2.66 to -2.25) | 3272.02 (1594.00 to 5060.48) | 2222.74 (977.54 to 3676.36) | 659.46 (335.67 to 987.87) | 327.05 (144.07 to 537.54) | -2.22 (-2.37 to -2.07) |
| Haiti | 967.54 (406.04 to 1603.56) | 690.56 (301.00 to 1114.47) | 21.85 (10.71 to 34.49) | 9.77 (4.41 to 15.71) | -2.51 (-2.75 to -2.27) | 57373.00 (20531.90 to 100724.50) | 29564.20 (11823.28 to 49720.47) | 807.71 (340.75 to 1336.10) | 298.11 (125.45 to 489.23) | -3.04 (-3.32 to -2.76) |
| Honduras | 394.69 (182.15 to 619.00) | 913.06 (470.72 to 1463.34) | 15.43 (7.97 to 24.15) | 16.66 (8.59 to 26.93) | 0.33 (0.11 to 0.54) | 20536.86 (8162.77 to 34236.85) | 26755.63 (13023.16 to 43366.93) | 493.82 (228.90 to 765.08) | 401.36 (199.45 to 651.75) | -0.60 (-0.73 to -0.46) |
| Hungary | 4299.51 (2569.14 to 6097.25) | 2227.52 (1088.46 to 3507.15) | 32.13 (19.33 to 45.25) | 11.45 (5.50 to 17.94) | -3.52 (-3.71 to -3.34) | 111330.65 (65737.22 to 158136.39) | 52521.12 (24792.66 to 83146.65) | 836.66 (487.75 to 1188.16) | 306.25 (144.44 to 489.05) | -3.48 (-3.63 to -3.32) |
| Iceland | 34.04 (19.82 to 49.89) | 18.90 (9.40 to 30.20) | 11.84 (6.90 to 17.23) | 3.15 (1.57 to 5.06) | -4.40 (-4.54 to -4.25) | 830.27 (484.05 to 1199.98) | 481.12 (235.47 to 773.07) | 306.70 (178.52 to 441.07) | 94.12 (46.20 to 149.31) | -4.03 (-4.21 to -3.84) |
| India | 163780.55 (76140.39 to 254020.80) | 220564.16 (111902.91 to 333755.30) | 32.80 (16.33 to 50.72) | 21.18 (10.53 to 32.31) | -1.39 (-1.51 to -1.27) | 8327688.81 (3358546.58 to 13438163.12) | 6469377.14 (3244890.92 to 9803644.52) | 1093.48 (492.60 to 1702.96) | 554.28 (274.76 to 849.08) | -2.16 (-2.25 to -2.07) |
| Indonesia | 28746.39 (15117.48 to 42925.32) | 52710.25 (29795.36 to 79009.69) | 26.99 (14.85 to 40.04) | 26.80 (15.00 to 39.99) | 0.08 (-0.02 to 0.17) | 1397035.98 (639889.44 to 2142681.69) | 1641906.30 (916770.29 to 2445068.27) | 929.25 (467.17 to 1397.26) | 676.39 (371.55 to 1012.61) | -0.90 (-0.96 to -0.85) |
| Iran (Islamic Republic of) | 4621.92 (2726.99 to 6544.53) | 6820.60 (4161.63 to 9716.60) | 18.04 (11.23 to 25.17) | 9.55 (5.85 to 13.73) | -2.12 (-2.30 to -1.94) | 194138.43 (100269.57 to 292795.45) | 202278.26 (117103.30 to 289503.86) | 507.81 (293.92 to 723.87) | 247.14 (143.41 to 355.67) | -2.26 (-2.40 to -2.12) |
| Iraq | 4310.04 (2569.29 to 6177.34) | 7323.61 (4167.73 to 11245.22) | 48.84 (30.63 to 68.94) | 36.97 (21.49 to 55.60) | -1.45 (-1.65 to -1.24) | 164727.55 (84207.02 to 245763.63) | 227607.86 (122298.44 to 343861.54) | 1372.31 (810.51 to 1966.04) | 894.87 (494.24 to 1366.75) | -1.80 (-1.93 to -1.66) |
| Ireland | 898.88 (458.60 to 1394.99) | 302.76 (128.28 to 493.14) | 23.00 (11.78 to 35.59) | 3.73 (1.58 to 6.09) | -6.02 (-6.29 to -5.75) | 19949.19 (10362.45 to 30758.81) | 7115.08 (3047.05 to 11689.59) | 515.33 (266.94 to 793.37) | 96.58 (41.30 to 158.81) | -5.61 (-5.85 to -5.37) |
| Israel | 559.30 (288.01 to 853.43) | 376.86 (154.93 to 649.84) | 12.41 (6.33 to 19.06) | 2.95 (1.20 to 5.11) | -4.73 (-4.86 to -4.61) | 14384.98 (7455.32 to 21628.34) | 10172.91 (4175.35 to 17312.22) | 313.04 (161.79 to 471.82) | 88.96 (36.67 to 150.07) | -4.15 (-4.25 to -4.06) |
| Italy | 10783.24 (5814.83 to 16078.51) | 6198.12 (2955.35 to 9837.87) | 12.69 (6.87 to 18.79) | 3.82 (1.78 to 6.07) | -3.87 (-3.96 to -3.77) | 262273.25 (134403.47 to 393630.00) | 132360.83 (59507.61 to 209478.54) | 325.98 (166.82 to 488.01) | 105.86 (47.08 to 169.36) | -3.62 (-3.70 to -3.54) |
| Jamaica | 213.17 (103.71 to 328.24) | 228.74 (102.29 to 373.59) | 11.60 (5.67 to 17.82) | 6.94 (3.09 to 11.37) | -2.08 (-2.41 to -1.75) | 5523.94 (2603.37 to 8574.45) | 5589.79 (2485.92 to 9149.97) | 296.74 (142.01 to 459.95) | 180.89 (80.03 to 296.29) | -2.04 (-2.35 to -1.73) |
| Japan | 18433.86 (9518.23 to 27861.47) | 13051.46 (5693.35 to 21658.96) | 11.99 (6.17 to 18.19) | 3.00 (1.32 to 4.90) | -4.52 (-4.59 to -4.45) | 431787.49 (221077.77 to 646732.95) | 274243.26 (121429.28 to 449298.35) | 272.74 (139.25 to 409.51) | 94.54 (44.04 to 152.23) | -3.54 (-3.63 to -3.44) |
| Jordan | 543.16 (312.32 to 787.61) | 927.23 (482.04 to 1469.12) | 40.20 (24.33 to 57.41) | 14.72 (7.75 to 23.32) | -3.81 (-4.21 to -3.40) | 22235.33 (11385.85 to 33163.83) | 35549.94 (18171.69 to 56519.79) | 1068.27 (617.25 to 1550.11) | 425.13 (216.60 to 679.04) | -3.42 (-3.74 to -3.10) |
| Kazakhstan | 4489.13 (2381.38 to 7002.01) | 3151.97 (1701.15 to 4620.52) | 36.73 (19.84 to 56.94) | 21.41 (11.79 to 31.50) | -2.42 (-2.83 to -2.02) | 166870.12 (78529.08 to 262361.18) | 86090.39 (44454.72 to 128050.01) | 1139.01 (557.71 to 1786.83) | 491.81 (257.31 to 735.68) | -3.49 (-3.94 to -3.04) |
| Kenya | 1991.77 (755.87 to 3303.72) | 1702.65 (742.23 to 2792.27) | 13.45 (5.82 to 22.06) | 7.94 (3.50 to 13.00) | -1.77 (-2.07 to -1.46) | 131837.63 (45873.34 to 225071.23) | 67762.58 (28536.56 to 111757.87) | 491.90 (196.44 to 811.74) | 214.92 (93.47 to 351.89) | -2.64 (-2.89 to -2.39) |
| Kiribati | 24.34 (12.41 to 38.09) | 43.70 (21.62 to 68.36) | 64.73 (33.82 to 100.61) | 69.97 (35.28 to 110.98) | 0.12 (-0.10 to 0.33) | 1062.08 (501.55 to 1665.27) | 1563.38 (764.14 to 2434.86) | 1903.66 (971.99 to 2956.98) | 1881.82 (925.79 to 2946.18) | -0.15 (-0.34 to 0.04) |
| Kuwait | 146.02 (89.61 to 204.34) | 375.37 (213.85 to 540.69) | 24.41 (14.97 to 34.22) | 13.60 (7.44 to 19.91) | -1.56 (-1.95 to -1.17) | 5817.98 (3330.83 to 8304.67) | 15312.10 (8158.02 to 22667.58) | 658.00 (396.83 to 933.76) | 409.46 (219.01 to 614.00) | -1.15 (-1.47 to -0.83) |
| Kyrgyzstan | 1538.02 (777.71 to 2313.24) | 1283.82 (807.50 to 1842.52) | 46.39 (24.94 to 68.14) | 31.90 (20.21 to 45.20) | -0.90 (-1.16 to -0.64) | 69905.81 (30591.67 to 108978.04) | 36066.26 (21926.60 to 53043.75) | 1565.51 (749.55 to 2392.84) | 720.71 (436.25 to 1047.40) | -2.44 (-2.63 to -2.25) |
| Lao People's Democratic Republic | 2070.96 (889.20 to 3294.92) | 1419.22 (697.08 to 2208.84) | 67.47 (33.93 to 103.83) | 34.88 (17.56 to 53.87) | -2.26 (-2.36 to -2.15) | 129377.74 (48346.93 to 213769.72) | 51619.30 (23692.86 to 81488.90) | 2611.34 (1136.40 to 4136.13) | 938.12 (439.74 to 1461.89) | -3.36 (-3.47 to -3.26) |
| Latvia | 1116.53 (649.67 to 1702.15) | 458.02 (253.96 to 736.34) | 32.57 (19.01 to 49.47) | 10.76 (6.01 to 17.21) | -3.89 (-4.18 to -3.60) | 25728.04 (14684.83 to 38613.04) | 9524.09 (5187.35 to 15426.41) | 763.41 (430.33 to 1141.16) | 268.23 (139.83 to 429.19) | -3.88 (-4.23 to -3.53) |
| Lebanon | 667.19 (378.11 to 998.66) | 1108.65 (551.32 to 1719.18) | 32.59 (18.92 to 48.67) | 17.21 (8.45 to 26.70) | -2.04 (-2.14 to -1.95) | 22085.00 (11329.86 to 33814.16) | 27285.31 (12449.15 to 42623.95) | 886.16 (478.60 to 1324.11) | 454.26 (205.80 to 708.02) | -2.15 (-2.24 to -2.06) |
| Lesotho | 211.14 (87.81 to 351.01) | 408.76 (178.63 to 649.65) | 21.36 (9.70 to 35.04) | 38.03 (17.13 to 59.81) | 3.19 (2.64 to 3.75) | 9858.89 (3662.91 to 16495.87) | 15840.33 (6443.18 to 25549.10) | 698.28 (286.73 to 1163.79) | 1165.58 (497.28 to 1857.15) | 2.85 (2.32 to 3.38) |
| Liberia | 336.84 (120.81 to 593.59) | 189.40 (82.28 to 309.80) | 15.18 (6.38 to 24.96) | 8.44 (3.91 to 13.54) | -2.02 (-2.15 to -1.90) | 23462.66 (7564.90 to 42863.77) | 8247.98 (3359.39 to 13944.22) | 676.96 (248.76 to 1172.34) | 241.43 (107.51 to 393.81) | -3.47 (-3.81 to -3.13) |
| Libya | 502.09 (289.19 to 740.85) | 1377.17 (745.39 to 2114.09) | 25.31 (14.75 to 37.06) | 27.47 (14.96 to 41.94) | 0.91 (0.67 to 1.15) | 18094.57 (10171.14 to 27188.06) | 46255.81 (24554.68 to 70557.32) | 707.82 (410.16 to 1029.54) | 772.83 (410.24 to 1183.16) | 0.87 (0.68 to 1.07) |
| Lithuania | 973.98 (519.70 to 1517.89) | 529.85 (291.04 to 862.85) | 22.25 (11.90 to 34.67) | 8.64 (4.79 to 14.06) | -3.31 (-3.56 to -3.06) | 22954.24 (12350.26 to 35803.43) | 11023.29 (5997.88 to 17766.14) | 532.35 (287.78 to 826.46) | 217.41 (118.14 to 349.52) | -3.14 (-3.44 to -2.84) |
| Luxembourg | 70.45 (32.94 to 105.87) | 38.51 (16.55 to 64.28) | 13.44 (6.29 to 20.24) | 3.46 (1.44 to 5.78) | -4.46 (-4.59 to -4.33) | 1718.17 (783.57 to 2602.09) | 977.16 (382.19 to 1633.20) | 337.36 (152.97 to 508.09) | 97.43 (37.68 to 162.44) | -4.20 (-4.33 to -4.07) |
| Madagascar | 2964.07 (1208.63 to 4825.83) | 1834.32 (766.10 to 3105.65) | 34.69 (15.59 to 55.30) | 13.90 (5.90 to 23.57) | -3.15 (-3.30 to -3.00) | 189538.67 (71674.14 to 315639.20) | 92418.26 (35596.22 to 156067.85) | 1343.61 (568.69 to 2154.04) | 441.46 (184.79 to 745.01) | -3.66 (-3.81 to -3.52) |
| Malawi | 1321.13 (474.25 to 2251.10) | 979.51 (438.06 to 1588.00) | 16.57 (7.20 to 26.67) | 11.60 (5.42 to 18.79) | -1.43 (-1.68 to -1.17) | 92334.66 (29565.67 to 165865.49) | 44345.73 (19036.29 to 73365.48) | 706.13 (270.26 to 1172.51) | 356.94 (161.58 to 575.35) | -2.51 (-2.77 to -2.26) |
| Malaysia | 2694.27 (1438.25 to 4022.95) | 5659.49 (2852.42 to 8920.24) | 29.48 (15.98 to 43.97) | 22.45 (11.29 to 35.49) | -0.65 (-0.89 to -0.41) | 87563.79 (44424.85 to 133158.81) | 159365.39 (79119.12 to 251312.07) | 783.99 (417.55 to 1177.95) | 557.83 (278.73 to 882.38) | -0.88 (-1.01 to -0.75) |
| Maldives | 42.79 (22.07 to 64.11) | 44.56 (22.46 to 67.47) | 43.52 (23.87 to 63.94) | 15.20 (7.60 to 23.17) | -3.73 (-3.92 to -3.54) | 1988.15 (916.78 to 3090.82) | 1363.16 (680.19 to 2051.24) | 1269.08 (662.98 to 1882.89) | 368.29 (178.53 to 549.21) | -4.23 (-4.49 to -3.98) |
| Mali | 985.71 (373.87 to 1653.79) | 1597.31 (659.83 to 2632.71) | 17.46 (7.78 to 28.20) | 15.25 (6.79 to 24.16) | -0.29 (-0.53 to -0.06) | 58571.84 (19240.34 to 101428.43) | 81715.06 (31186.06 to 138688.89) | 602.33 (237.22 to 992.78) | 461.39 (194.88 to 757.22) | -0.78 (-1.13 to -0.42) |
| Malta | 64.66 (36.94 to 92.83) | 37.88 (18.15 to 61.26) | 15.93 (9.12 to 22.98) | 3.97 (1.90 to 6.38) | -4.43 (-4.52 to -4.34) | 1607.45 (908.47 to 2329.13) | 938.30 (439.65 to 1516.23) | 386.82 (218.23 to 562.04) | 120.59 (56.27 to 194.77) | -3.75 (-3.88 to -3.62) |
| Marshall Islands | 8.37 (3.93 to 13.22) | 14.11 (6.88 to 22.15) | 50.27 (24.50 to 79.51) | 46.77 (22.49 to 74.98) | -0.27 (-0.42 to -0.12) | 356.15 (155.15 to 576.96) | 544.36 (254.99 to 861.41) | 1381.70 (654.49 to 2171.32) | 1319.25 (624.84 to 2094.44) | -0.20 (-0.41 to 0.01) |
| Mauritania | 427.50 (195.88 to 678.35) | 379.71 (173.41 to 601.83) | 35.14 (17.19 to 55.76) | 17.94 (8.31 to 28.29) | -2.40 (-2.51 to -2.29) | 20760.66 (8649.10 to 33531.73) | 13617.97 (5844.01 to 21729.26) | 1072.17 (500.90 to 1694.86) | 468.25 (213.70 to 738.41) | -2.84 (-2.93 to -2.75) |
| Mauritius | 225.78 (122.60 to 331.75) | 348.91 (167.19 to 538.32) | 35.34 (19.20 to 52.37) | 20.38 (9.76 to 31.46) | -1.74 (-1.96 to -1.52) | 6870.29 (3621.88 to 10042.36) | 9810.63 (4513.18 to 15145.84) | 886.48 (471.29 to 1309.39) | 570.36 (262.45 to 877.77) | -1.32 (-1.55 to -1.09) |
| Mexico | 9193.29 (4185.49 to 14380.38) | 8020.15 (3850.25 to 12621.06) | 20.02 (9.35 to 31.03) | 6.77 (3.28 to 10.70) | -3.75 (-4.17 to -3.34) | 432344.21 (172901.51 to 691136.91) | 237036.09 (108912.73 to 378328.17) | 610.88 (269.50 to 958.55) | 186.88 (84.97 to 299.56) | -4.03 (-4.46 to -3.59) |
| Micronesia (Federated States of) | 32.05 (15.70 to 50.35) | 33.62 (16.47 to 51.93) | 64.46 (32.53 to 98.39) | 53.63 (26.24 to 81.96) | -0.61 (-0.63 to -0.59) | 1303.99 (603.19 to 2103.62) | 1149.73 (555.36 to 1796.88) | 1831.30 (894.48 to 2871.83) | 1437.00 (696.37 to 2228.50) | -0.79 (-0.82 to -0.77) |
| Monaco | 6.73 (3.08 to 10.55) | 4.77 (1.60 to 8.34) | 9.91 (4.40 to 15.87) | 5.00 (1.66 to 8.83) | -2.16 (-2.19 to -2.12) | 148.67 (67.88 to 237.40) | 110.49 (36.62 to 198.89) | 267.28 (120.32 to 423.63) | 147.84 (49.61 to 260.84) | -1.91 (-1.95 to -1.87) |
| Mongolia | 651.47 (307.89 to 1038.25) | 394.85 (222.59 to 619.34) | 39.06 (21.13 to 60.98) | 20.26 (11.57 to 32.45) | -2.66 (-2.88 to -2.44) | 42077.86 (16392.56 to 68644.97) | 13977.51 (7462.32 to 21238.89) | 1671.66 (786.13 to 2655.40) | 526.00 (286.24 to 803.82) | -4.13 (-4.32 to -3.94) |
| Montenegro | 159.84 (93.41 to 234.05) | 268.58 (140.61 to 397.43) | 27.05 (15.71 to 39.30) | 31.55 (16.84 to 46.85) | 0.55 (0.32 to 0.78) | 4069.76 (2260.80 to 5945.90) | 5558.04 (2863.29 to 8349.35) | 657.56 (367.24 to 962.80) | 612.28 (320.12 to 917.74) | -0.25 (-0.54 to 0.04) |
| Morocco | 5925.96 (3526.78 to 8715.14) | 7787.86 (4289.25 to 11783.83) | 38.45 (23.73 to 56.16) | 25.39 (14.05 to 37.70) | -1.46 (-1.58 to -1.35) | 244943.96 (123615.66 to 369098.31) | 220543.27 (122611.17 to 335420.28) | 1164.44 (675.07 to 1717.05) | 636.83 (352.98 to 963.19) | -2.07 (-2.19 to -1.94) |
| Mozambique | 1851.17 (646.57 to 3187.47) | 1487.16 (596.74 to 2447.30) | 16.70 (7.13 to 27.03) | 11.45 (4.84 to 19.07) | -0.70 (-0.87 to -0.53) | 127846.16 (40074.42 to 227196.79) | 70536.19 (26072.03 to 118103.17) | 717.35 (258.51 to 1228.89) | 345.10 (141.75 to 565.79) | -1.83 (-2.00 to -1.65) |
| Myanmar | 17875.80 (8215.27 to 27697.74) | 11615.03 (5410.31 to 18419.39) | 68.65 (33.50 to 105.85) | 27.48 (12.90 to 43.38) | -3.20 (-3.32 to -3.08) | 934306.40 (382264.73 to 1509788.05) | 361158.67 (162678.07 to 564086.49) | 2514.97 (1126.27 to 3945.13) | 737.57 (330.76 to 1148.83) | -4.18 (-4.34 to -4.02) |
| Namibia | 215.03 (93.27 to 351.86) | 251.93 (107.89 to 415.09) | 30.78 (13.88 to 49.98) | 19.48 (8.50 to 32.05) | -1.63 (-1.96 to -1.30) | 9766.94 (3947.83 to 16444.65) | 8770.55 (3635.25 to 14832.73) | 903.45 (396.65 to 1480.52) | 527.55 (223.88 to 868.60) | -1.85 (-2.17 to -1.52) |
| Nauru | 3.67 (1.85 to 5.44) | 3.84 (1.94 to 5.83) | 81.18 (41.58 to 119.19) | 69.86 (35.38 to 105.61) | -0.66 (-1.02 to -0.30) | 154.53 (73.48 to 232.50) | 147.90 (72.01 to 228.05) | 2286.70 (1151.82 to 3375.57) | 1996.51 (994.39 to 3017.32) | -0.63 (-1.08 to -0.17) |
| Nepal | 6937.47 (2681.86 to 11253.26) | 4547.65 (2198.97 to 7050.62) | 46.31 (21.04 to 73.84) | 23.05 (10.98 to 35.69) | -2.35 (-2.49 to -2.20) | 466128.54 (161374.89 to 759234.83) | 133776.92 (62229.43 to 211658.90) | 1871.94 (729.75 to 3023.49) | 561.43 (262.12 to 884.68) | -4.03 (-4.13 to -3.93) |
| Netherlands | 2029.14 (1018.51 to 3301.10) | 1035.44 (427.15 to 1745.99) | 10.48 (5.26 to 16.95) | 2.90 (1.19 to 4.90) | -4.24 (-4.43 to -4.05) | 51014.45 (25558.63 to 82295.19) | 25284.65 (10260.08 to 42605.09) | 277.93 (137.87 to 447.42) | 83.39 (33.85 to 139.58) | -3.87 (-4.01 to -3.74) |
| New Zealand | 402.51 (207.25 to 644.73) | 240.12 (110.50 to 409.77) | 10.69 (5.49 to 17.18) | 2.88 (1.32 to 4.85) | -4.34 (-4.50 to -4.18) | 10937.57 (5693.02 to 17601.94) | 6574.86 (2886.83 to 11097.18) | 298.12 (155.69 to 481.42) | 89.14 (39.81 to 150.13) | -3.96 (-4.16 to -3.75) |
| Nicaragua | 301.92 (119.05 to 497.47) | 340.60 (166.65 to 535.77) | 12.60 (6.02 to 19.70) | 7.58 (3.73 to 12.00) | -1.16 (-1.36 to -0.95) | 18435.59 (6705.65 to 31384.09) | 11964.02 (5508.05 to 19253.25) | 469.76 (192.83 to 760.35) | 229.03 (104.66 to 370.28) | -1.89 (-2.06 to -1.71) |
| Niger | 1654.10 (547.93 to 2931.18) | 1127.65 (419.41 to 1882.79) | 19.48 (7.66 to 32.28) | 8.93 (3.76 to 14.37) | -2.81 (-3.03 to -2.58) | 130109.03 (40547.88 to 235535.60) | 68584.71 (22156.31 to 119179.32) | 984.32 (347.12 to 1718.74) | 293.50 (117.33 to 476.45) | -4.30 (-4.63 to -3.97) |
| Nigeria | 8640.44 (3178.62 to 14745.89) | 6336.16 (2546.39 to 10467.94) | 10.43 (4.65 to 16.81) | 4.91 (2.28 to 7.78) | -2.53 (-2.65 to -2.41) | 598360.70 (193349.24 to 1045695.47) | 367485.27 (127405.64 to 656079.85) | 481.58 (181.74 to 819.99) | 174.36 (71.92 to 287.01) | -3.28 (-3.42 to -3.13) |
| Niue | 0.84 (0.43 to 1.32) | 0.68 (0.32 to 1.09) | 36.07 (18.25 to 56.58) | 34.16 (15.83 to 54.59) | -0.59 (-0.72 to -0.46) | 21.51 (10.26 to 33.79) | 19.74 (8.94 to 32.33) | 971.20 (465.15 to 1522.16) | 1058.51 (466.87 to 1689.69) | -0.46 (-0.69 to -0.23) |
| North Macedonia | 854.26 (511.93 to 1215.88) | 1024.32 (578.70 to 1538.72) | 51.26 (30.54 to 73.26) | 41.15 (23.67 to 60.77) | -1.09 (-1.59 to -0.58) | 22265.66 (12379.87 to 31882.91) | 22597.29 (11995.90 to 33992.93) | 1236.62 (681.00 to 1777.15) | 768.80 (418.65 to 1146.55) | -1.74 (-2.04 to -1.44) |
| Northern Mariana Islands | 4.71 (2.26 to 7.41) | 9.92 (4.92 to 15.53) | 33.12 (15.59 to 50.95) | 23.81 (11.71 to 37.33) | -1.15 (-1.25 to -1.05) | 185.11 (86.20 to 295.66) | 307.24 (149.76 to 480.18) | 792.43 (369.89 to 1228.05) | 592.14 (290.47 to 926.32) | -0.96 (-1.01 to -0.91) |
| Norway | 762.87 (410.37 to 1135.34) | 273.37 (117.97 to 452.13) | 11.22 (6.13 to 16.56) | 2.55 (1.12 to 4.20) | -5.03 (-5.16 to -4.90) | 17094.59 (9472.95 to 24822.01) | 6440.98 (2901.15 to 10406.81) | 287.40 (162.46 to 413.33) | 71.51 (32.96 to 114.30) | -4.72 (-4.83 to -4.61) |
| Oman | 177.41 (93.58 to 292.56) | 228.05 (113.98 to 361.11) | 24.69 (13.50 to 40.74) | 14.12 (6.92 to 22.17) | -1.32 (-1.49 to -1.16) | 7157.26 (3575.02 to 11700.93) | 8194.48 (4075.59 to 13016.72) | 657.11 (349.98 to 1074.60) | 339.54 (165.77 to 533.73) | -1.70 (-1.83 to -1.56) |
| Pakistan | 24343.23 (11155.20 to 36943.04) | 29319.50 (15564.52 to 44075.30) | 33.71 (17.50 to 50.85) | 25.18 (13.59 to 37.55) | -1.19 (-1.48 to -0.90) | 1293222.93 (521817.49 to 2026150.31) | 1161133.76 (552645.12 to 1791844.56) | 1157.24 (532.85 to 1757.97) | 721.53 (361.88 to 1070.34) | -1.54 (-1.77 to -1.31) |
| Palau | 3.20 (1.62 to 5.08) | 5.45 (2.62 to 8.76) | 36.62 (18.49 to 58.21) | 31.05 (14.67 to 49.08) | -0.46 (-0.50 to -0.42) | 110.15 (54.06 to 175.96) | 175.16 (83.19 to 284.25) | 1001.69 (493.21 to 1588.56) | 819.31 (384.30 to 1295.61) | -0.58 (-0.62 to -0.55) |
| Palestine | 363.24 (202.58 to 533.96) | 496.98 (265.50 to 735.84) | 42.15 (24.34 to 61.21) | 24.42 (13.16 to 36.26) | -1.96 (-2.19 to -1.74) | 12867.23 (6254.26 to 20006.16) | 15693.92 (8346.43 to 23677.09) | 1008.85 (553.88 to 1469.55) | 564.74 (301.36 to 847.18) | -2.02 (-2.17 to -1.87) |
| Panama | 130.97 (65.83 to 204.32) | 160.86 (75.73 to 270.38) | 8.80 (4.53 to 13.68) | 3.56 (1.67 to 5.98) | -3.16 (-3.36 to -2.97) | 4299.81 (2013.31 to 6915.92) | 4702.04 (2141.57 to 7975.26) | 241.50 (118.01 to 377.03) | 108.16 (48.51 to 183.79) | -2.79 (-2.97 to -2.61) |
| Papua New Guinea | 1323.86 (518.88 to 2130.73) | 2561.26 (1124.87 to 3994.56) | 58.36 (26.11 to 94.71) | 50.28 (22.94 to 78.68) | -0.46 (-0.55 to -0.38) | 78200.15 (29565.74 to 124700.30) | 123834.73 (51573.43 to 197615.99) | 1952.05 (777.55 to 3162.05) | 1479.33 (642.80 to 2329.02) | -0.74 (-0.88 to -0.61) |
| Paraguay | 381.21 (200.04 to 585.07) | 592.45 (250.72 to 965.06) | 16.09 (8.67 to 24.61) | 10.64 (4.51 to 17.33) | -1.13 (-1.26 to -1.01) | 14185.71 (6650.15 to 22203.87) | 16331.08 (7152.33 to 26164.02) | 453.25 (233.29 to 700.09) | 272.09 (118.35 to 436.27) | -1.58 (-1.67 to -1.49) |
| Peru | 1708.11 (657.78 to 2791.70) | 1127.17 (437.74 to 1988.21) | 10.63 (4.39 to 17.04) | 3.31 (1.28 to 5.82) | -4.54 (-4.94 to -4.13) | 94447.74 (32718.49 to 161318.59) | 32439.18 (12677.57 to 55396.47) | 419.99 (156.61 to 695.14) | 93.66 (36.34 to 159.85) | -5.35 (-5.69 to -5.01) |
| Philippines | 12301.97 (6089.89 to 18708.67) | 18391.81 (9681.21 to 27997.54) | 38.42 (20.74 to 57.31) | 25.44 (12.96 to 38.69) | -0.83 (-1.00 to -0.66) | 633071.11 (271339.87 to 1006832.77) | 583611.44 (296448.63 to 885160.61) | 1172.30 (573.22 to 1783.97) | 660.15 (337.52 to 1002.55) | -1.42 (-1.55 to -1.29) |
| Poland | 14040.52 (8789.13 to 19636.90) | 6519.00 (3360.26 to 10406.24) | 34.32 (21.65 to 48.19) | 8.95 (4.55 to 14.31) | -4.63 (-4.80 to -4.45) | 357650.31 (219808.70 to 499804.41) | 153659.90 (74548.58 to 246494.17) | 853.44 (523.45 to 1197.63) | 236.03 (113.36 to 377.57) | -4.31 (-4.49 to -4.13) |
| Portugal | 2137.51 (1182.62 to 3250.20) | 1265.11 (562.37 to 2053.95) | 17.46 (9.75 to 26.45) | 4.58 (2.00 to 7.48) | -4.54 (-4.73 to -4.34) | 49945.43 (26767.35 to 76550.99) | 27234.62 (11233.88 to 44875.62) | 406.98 (215.29 to 621.61) | 129.56 (53.74 to 211.64) | -3.95 (-4.13 to -3.77) |
| Puerto Rico | 246.59 (124.30 to 395.80) | 221.27 (100.14 to 363.04) | 7.41 (3.71 to 11.90) | 2.80 (1.25 to 4.62) | -3.50 (-3.71 to -3.30) | 6232.44 (2966.55 to 10004.17) | 5489.08 (2318.11 to 9049.20) | 178.55 (84.67 to 286.70) | 90.95 (38.20 to 149.96) | -2.52 (-2.70 to -2.35) |
| Qatar | 33.69 (18.91 to 50.41) | 102.75 (53.61 to 159.73) | 42.40 (24.52 to 62.13) | 17.73 (8.87 to 27.05) | -3.21 (-3.92 to -2.50) | 1255.86 (674.60 to 1894.12) | 4826.77 (2438.23 to 7570.42) | 890.42 (505.57 to 1316.89) | 411.72 (202.60 to 635.27) | -2.77 (-3.29 to -2.24) |
| Republic of Korea | 5336.22 (2908.55 to 7878.57) | 4558.34 (1938.50 to 7607.81) | 23.76 (12.55 to 35.24) | 5.02 (2.13 to 8.34) | -5.45 (-5.67 to -5.23) | 160847.88 (86025.40 to 238242.59) | 125604.64 (54214.26 to 209799.89) | 539.32 (285.65 to 799.56) | 142.10 (61.73 to 237.29) | -4.73 (-4.92 to -4.54) |
| Republic of Moldova | 1115.70 (668.11 to 1575.44) | 862.98 (535.90 to 1235.11) | 30.03 (17.94 to 42.65) | 14.93 (9.32 to 21.35) | -2.75 (-3.08 to -2.41) | 34431.88 (19091.13 to 49592.33) | 21998.85 (13232.09 to 31685.75) | 823.96 (461.26 to 1185.03) | 416.62 (241.53 to 609.38) | -2.54 (-2.89 to -2.18) |
| Romania | 7492.18 (4116.93 to 10893.08) | 4926.15 (2582.88 to 7957.58) | 33.29 (18.26 to 48.59) | 13.46 (6.87 to 21.84) | -3.37 (-3.61 to -3.13) | 227431.34 (112652.19 to 341226.16) | 115499.79 (56464.83 to 188712.72) | 1018.71 (474.06 to 1539.02) | 373.57 (177.06 to 602.98) | -3.54 (-3.79 to -3.28) |
| Russian Federation | 41322.07 (25980.68 to 56743.52) | 33569.53 (20684.77 to 47333.13) | 26.33 (16.99 to 36.55) | 14.34 (8.84 to 20.06) | -2.50 (-3.27 to -1.71) | 1092352.85 (644636.69 to 1535274.13) | 818663.52 (487676.43 to 1173255.08) | 674.93 (389.57 to 948.92) | 373.43 (220.25 to 534.61) | -2.47 (-3.23 to -1.71) |
| Rwanda | 1131.84 (429.86 to 1874.79) | 560.14 (242.00 to 923.99) | 22.89 (10.49 to 37.30) | 8.78 (3.83 to 14.46) | -4.01 (-4.41 to -3.61) | 71912.15 (23930.06 to 124160.61) | 23185.64 (9644.02 to 39101.24) | 911.07 (370.29 to 1499.32) | 256.10 (112.39 to 418.60) | -5.07 (-5.45 to -4.70) |
| Saint Kitts and Nevis | 5.36 (2.86 to 8.07) | 3.93 (1.89 to 6.23) | 15.14 (8.05 to 22.85) | 6.89 (3.26 to 10.89) | -2.62 (-2.80 to -2.44) | 135.15 (70.82 to 203.36) | 117.38 (54.82 to 189.81) | 377.48 (200.20 to 568.49) | 177.17 (82.65 to 285.34) | -2.57 (-2.73 to -2.41) |
| Saint Lucia | 10.48 (5.35 to 16.24) | 10.55 (4.85 to 17.43) | 13.50 (6.90 to 21.15) | 4.63 (2.13 to 7.66) | -4.41 (-4.81 to -4.00) | 301.25 (147.49 to 469.66) | 305.39 (134.82 to 505.70) | 331.70 (165.11 to 516.19) | 134.19 (58.83 to 223.34) | -3.54 (-3.80 to -3.28) |
| Saint Vincent and the Grenadines | 8.66 (4.07 to 13.18) | 9.69 (4.38 to 15.29) | 12.88 (6.09 to 19.39) | 7.55 (3.39 to 11.83) | -1.98 (-2.15 to -1.82) | 247.17 (111.97 to 390.43) | 271.51 (121.78 to 437.33) | 324.01 (148.42 to 508.24) | 202.09 (90.09 to 325.87) | -1.81 (-1.96 to -1.67) |
| Samoa | 39.15 (19.69 to 61.54) | 59.87 (31.13 to 89.86) | 48.43 (25.08 to 75.28) | 45.64 (24.16 to 68.49) | -0.28 (-0.36 to -0.19) | 1422.98 (654.46 to 2276.96) | 1858.30 (942.87 to 2811.40) | 1261.50 (624.51 to 1984.15) | 1184.33 (607.49 to 1787.25) | -0.25 (-0.31 to -0.18) |
| San Marino | 2.41 (1.11 to 3.89) | 1.46 (0.60 to 2.53) | 6.86 (3.16 to 11.14) | 1.79 (0.69 to 3.21) | -3.56 (-3.87 to -3.24) | 56.41 (25.41 to 91.61) | 37.91 (15.37 to 66.21) | 177.90 (79.49 to 289.52) | 61.01 (24.39 to 105.73) | -2.95 (-3.17 to -2.74) |
| Sao Tome and Principe | 5.77 (2.27 to 9.84) | 5.43 (2.33 to 9.09) | 6.93 (2.98 to 11.59) | 4.92 (2.19 to 8.11) | -1.21 (-1.32 to -1.10) | 304.04 (104.79 to 531.61) | 205.76 (85.85 to 349.09) | 251.79 (101.26 to 427.40) | 142.17 (61.57 to 237.32) | -2.04 (-2.16 to -1.92) |
| Saudi Arabia | 1696.10 (978.20 to 2575.14) | 4144.96 (2338.11 to 6070.70) | 27.89 (16.72 to 41.55) | 21.20 (11.55 to 31.34) | -0.94 (-1.11 to -0.76) | 66138.02 (35116.58 to 104007.23) | 163199.61 (87998.70 to 240747.26) | 740.36 (428.93 to 1121.77) | 573.48 (310.98 to 839.11) | -0.72 (-0.88 to -0.57) |
| Senegal | 1579.30 (685.71 to 2519.48) | 1308.48 (612.94 to 2049.89) | 33.00 (16.69 to 50.53) | 18.02 (8.50 to 28.24) | -2.27 (-2.47 to -2.07) | 92321.07 (34064.21 to 154502.54) | 46748.98 (20689.71 to 74466.75) | 1117.96 (508.33 to 1773.12) | 467.28 (218.07 to 736.64) | -3.09 (-3.33 to -2.85) |
| Serbia | 3624.90 (2026.35 to 5355.01) | 3635.07 (1955.14 to 5591.28) | 41.32 (23.71 to 60.12) | 21.47 (11.50 to 33.20) | -2.65 (-3.04 to -2.27) | 88850.90 (47558.20 to 132299.63) | 74862.14 (38423.52 to 117254.44) | 892.03 (485.48 to 1310.54) | 472.44 (239.40 to 741.47) | -2.43 (-2.78 to -2.09) |
| Seychelles | 14.50 (7.24 to 22.46) | 16.48 (7.85 to 26.32) | 25.46 (12.77 to 39.34) | 16.22 (7.60 to 25.82) | -1.11 (-1.27 to -0.96) | 403.82 (199.27 to 620.11) | 495.78 (236.22 to 781.62) | 671.82 (334.11 to 1029.33) | 444.37 (209.06 to 701.89) | -0.97 (-1.11 to -0.84) |
| Sierra Leone | 1333.40 (475.38 to 2276.51) | 750.33 (319.68 to 1258.66) | 35.39 (15.05 to 56.55) | 18.41 (8.56 to 29.90) | -2.07 (-2.33 to -1.81) | 89667.32 (28842.34 to 159149.92) | 33256.58 (12607.36 to 58537.27) | 1494.89 (544.07 to 2521.84) | 519.03 (222.30 to 865.74) | -3.53 (-3.76 to -3.29) |
| Singapore | 298.30 (157.21 to 464.98) | 278.26 (124.63 to 461.40) | 14.55 (7.54 to 22.66) | 3.36 (1.49 to 5.57) | -4.45 (-4.55 to -4.36) | 9454.76 (4977.11 to 14623.43) | 8261.85 (3833.35 to 13442.14) | 394.39 (203.60 to 612.02) | 100.12 (46.62 to 161.57) | -4.22 (-4.30 to -4.14) |
| Slovakia | 1944.38 (1067.53 to 3012.19) | 1286.14 (662.03 to 2120.96) | 34.26 (18.82 to 53.06) | 13.91 (7.11 to 22.91) | -2.71 (-2.86 to -2.56) | 49828.04 (26323.94 to 76427.30) | 29055.81 (14246.45 to 47662.46) | 886.35 (462.80 to 1362.68) | 335.08 (161.72 to 548.91) | -2.94 (-3.07 to -2.81) |
| Slovenia | 488.88 (266.54 to 704.53) | 320.65 (148.01 to 512.16) | 20.42 (11.03 to 29.67) | 6.60 (2.98 to 10.51) | -3.69 (-3.82 to -3.56) | 12320.62 (6437.32 to 18027.54) | 7021.61 (2993.17 to 11121.36) | 530.03 (274.06 to 784.13) | 175.84 (72.54 to 280.06) | -3.58 (-3.68 to -3.48) |
| Solomon Islands | 97.24 (42.86 to 153.50) | 190.90 (91.31 to 300.93) | 67.14 (31.73 to 104.97) | 61.04 (29.72 to 96.63) | -0.20 (-0.36 to -0.05) | 4790.70 (1928.67 to 7593.94) | 7159.12 (3294.24 to 11254.98) | 1871.13 (841.11 to 2937.45) | 1601.29 (765.17 to 2524.58) | -0.38 (-0.53 to -0.22) |
| Somalia | 1264.10 (433.82 to 2207.53) | 1397.79 (516.82 to 2352.97) | 25.48 (11.58 to 41.41) | 16.11 (6.62 to 26.61) | -1.33 (-1.40 to -1.26) | 88176.27 (27225.31 to 155508.49) | 81300.93 (26289.79 to 142638.31) | 958.48 (378.68 to 1597.76) | 497.84 (201.26 to 825.62) | -1.95 (-2.06 to -1.85) |
| South Africa | 7053.11 (3040.96 to 10980.56) | 7691.31 (3399.62 to 11973.76) | 28.32 (12.78 to 43.86) | 17.90 (7.96 to 27.79) | -1.26 (-1.67 to -0.85) | 357470.05 (139897.63 to 568904.97) | 262544.24 (113772.20 to 411431.15) | 1065.28 (445.37 to 1665.64) | 531.79 (230.22 to 834.47) | -1.93 (-2.34 to -1.51) |
| South Sudan | 898.59 (328.52 to 1538.74) | 735.79 (289.98 to 1273.99) | 19.84 (8.33 to 32.50) | 14.06 (6.05 to 23.62) | -1.10 (-1.35 to -0.85) | 58969.28 (19920.97 to 106305.50) | 41424.72 (14840.97 to 75411.93) | 810.43 (305.29 to 1370.42) | 469.75 (190.44 to 804.65) | -1.61 (-2.01 to -1.22) |
| Spain | 9484.66 (4944.64 to 14369.60) | 5291.85 (2277.16 to 8852.59) | 18.26 (9.56 to 27.69) | 4.57 (1.95 to 7.79) | -4.45 (-4.65 to -4.24) | 206420.94 (106331.45 to 316807.77) | 115329.65 (48949.71 to 194804.18) | 409.76 (210.52 to 626.76) | 127.95 (53.68 to 217.55) | -3.82 (-3.98 to -3.65) |
| Sri Lanka | 2099.40 (1074.25 to 3213.67) | 2766.55 (1209.53 to 4829.64) | 23.77 (12.38 to 35.80) | 11.60 (5.15 to 20.07) | -1.96 (-2.11 to -1.80) | 62705.03 (31683.81 to 95319.91) | 73312.54 (32335.70 to 127746.38) | 549.64 (276.56 to 835.35) | 285.34 (125.49 to 495.45) | -1.98 (-2.09 to -1.87) |
| Sudan | 4859.96 (2605.89 to 7328.72) | 4466.01 (2468.72 to 6974.14) | 40.90 (24.37 to 58.97) | 23.49 (13.32 to 36.08) | -2.00 (-2.09 to -1.92) | 247083.39 (109745.98 to 414785.28) | 153414.94 (79929.31 to 242236.76) | 1335.76 (726.11 to 1996.27) | 619.88 (334.89 to 954.78) | -2.63 (-2.71 to -2.56) |
| Suriname | 54.83 (29.37 to 84.75) | 62.53 (30.10 to 104.85) | 21.47 (11.57 to 33.55) | 10.28 (4.89 to 17.24) | -2.45 (-2.72 to -2.17) | 1848.84 (922.45 to 2856.88) | 2009.53 (959.73 to 3341.73) | 620.04 (318.46 to 955.75) | 320.36 (150.07 to 529.99) | -2.29 (-2.53 to -2.04) |
| Sweden | 1256.17 (701.54 to 1888.26) | 513.51 (263.50 to 806.69) | 8.39 (4.70 to 12.61) | 2.20 (1.15 to 3.45) | -4.17 (-4.25 to -4.08) | 28229.59 (15934.29 to 42905.11) | 11703.88 (5820.90 to 18306.98) | 214.99 (120.70 to 328.41) | 62.88 (31.43 to 99.25) | -3.82 (-3.88 to -3.76) |
| Switzerland | 1088.35 (526.12 to 1643.92) | 427.08 (178.68 to 732.43) | 10.46 (5.10 to 15.70) | 2.11 (0.86 to 3.62) | -5.26 (-5.45 to -5.08) | 24706.22 (11888.22 to 37538.16) | 10936.06 (4530.62 to 18415.86) | 262.02 (125.98 to 400.94) | 69.12 (28.31 to 116.16) | -4.48 (-4.69 to -4.28) |
| Syrian Arab Republic | 2551.70 (1342.80 to 3769.48) | 3049.41 (1526.69 to 4785.83) | 45.33 (24.69 to 65.71) | 27.55 (14.02 to 42.80) | -1.96 (-2.15 to -1.76) | 100707.13 (50145.77 to 155299.01) | 91336.27 (45418.75 to 142111.65) | 1254.53 (659.86 to 1843.30) | 685.21 (343.74 to 1056.49) | -2.12 (-2.34 to -1.90) |
| Taiwan (Province of China) | 2991.40 (1585.33 to 4483.33) | 4212.02 (1713.92 to 7006.88) | 23.40 (12.27 to 35.06) | 9.67 (3.94 to 16.03) | -2.80 (-2.90 to -2.69) | 85103.40 (43606.93 to 127749.31) | 97850.20 (41147.66 to 157614.05) | 550.58 (282.96 to 823.48) | 246.65 (103.84 to 394.39) | -2.57 (-2.66 to -2.47) |
| Tajikistan | 1871.40 (951.72 to 2863.87) | 1104.35 (628.12 to 1638.45) | 49.23 (29.52 to 70.40) | 20.30 (12.10 to 29.75) | -3.16 (-3.47 to -2.84) | 100788.17 (41832.75 to 164821.63) | 44822.23 (22365.41 to 70675.24) | 1714.00 (867.28 to 2628.64) | 564.60 (303.13 to 855.16) | -3.79 (-3.97 to -3.61) |
| Thailand | 6560.89 (3331.18 to 9838.11) | 10483.24 (4645.72 to 16992.94) | 20.81 (10.53 to 31.39) | 9.99 (4.42 to 16.10) | -2.77 (-2.92 to -2.62) | 225162.62 (109321.65 to 338689.62) | 266857.69 (116936.57 to 429425.85) | 567.85 (277.62 to 856.73) | 276.20 (121.91 to 440.99) | -2.66 (-2.81 to -2.50) |
| Timor-Leste | 284.22 (118.72 to 456.25) | 247.15 (123.22 to 378.13) | 45.07 (22.38 to 68.88) | 30.85 (16.15 to 46.62) | -0.98 (-1.25 to -0.71) | 21282.72 (7882.77 to 35812.28) | 9405.19 (4262.95 to 14457.75) | 1972.44 (866.86 to 3147.92) | 842.00 (405.76 to 1276.64) | -2.62 (-2.97 to -2.28) |
| Togo | 386.22 (154.83 to 627.30) | 519.24 (236.25 to 822.25) | 19.25 (9.12 to 30.61) | 12.85 (6.25 to 20.07) | -1.42 (-1.56 to -1.29) | 23472.05 (8455.44 to 39782.13) | 21749.10 (9641.18 to 35081.41) | 674.32 (290.39 to 1073.73) | 384.44 (180.37 to 607.84) | -1.90 (-2.05 to -1.76) |
| Tokelau | 0.51 (0.26 to 0.80) | 0.47 (0.23 to 0.77) | 40.61 (20.91 to 63.05) | 33.39 (16.05 to 54.39) | -1.03 (-1.17 to -0.90) | 15.50 (7.41 to 24.39) | 14.51 (6.72 to 23.25) | 1107.72 (546.65 to 1751.92) | 1118.94 (507.32 to 1789.02) | -0.85 (-1.18 to -0.53) |
| Tonga | 18.92 (8.31 to 29.65) | 25.12 (11.30 to 42.22) | 35.56 (16.00 to 55.38) | 32.02 (14.41 to 53.52) | -0.32 (-0.47 to -0.17) | 697.65 (291.53 to 1105.23) | 720.39 (314.91 to 1219.89) | 961.13 (420.16 to 1504.27) | 835.28 (372.04 to 1413.44) | -0.48 (-0.62 to -0.35) |
| Trinidad and Tobago | 187.89 (91.03 to 280.34) | 195.86 (85.76 to 313.92) | 24.98 (12.16 to 37.29) | 10.48 (4.58 to 16.80) | -3.28 (-3.53 to -3.03) | 5501.97 (2607.96 to 8357.98) | 5687.08 (2438.22 to 9090.95) | 636.01 (301.44 to 963.83) | 307.51 (131.76 to 493.44) | -2.88 (-3.13 to -2.62) |
| Tunisia | 1638.98 (935.19 to 2381.24) | 2371.61 (1238.09 to 3742.74) | 36.05 (21.15 to 51.76) | 19.72 (10.36 to 31.04) | -2.28 (-2.43 to -2.13) | 60888.14 (31160.61 to 95245.75) | 64209.46 (32834.69 to 100492.55) | 966.10 (532.16 to 1408.76) | 493.76 (251.66 to 773.79) | -2.38 (-2.46 to -2.29) |
| Türkiye | 16343.21 (8586.57 to 24055.76) | 18841.54 (9461.22 to 29068.09) | 48.41 (27.14 to 69.95) | 22.37 (11.32 to 34.36) | -2.47 (-2.85 to -2.09) | 629232.54 (295846.21 to 974325.03) | 439971.77 (211192.83 to 681261.84) | 1371.11 (690.49 to 2039.10) | 488.74 (237.18 to 750.26) | -3.48 (-3.82 to -3.13) |
| Turkmenistan | 1380.93 (721.68 to 2033.67) | 1315.62 (809.67 to 1868.00) | 57.96 (34.21 to 80.70) | 35.67 (22.14 to 49.98) | -2.26 (-2.62 to -1.89) | 71981.12 (31382.95 to 111933.51) | 41581.09 (23749.07 to 60684.82) | 1949.98 (996.87 to 2889.56) | 952.70 (550.96 to 1375.21) | -2.83 (-3.19 to -2.48) |
| Tuvalu | 4.40 (2.08 to 6.69) | 4.28 (2.23 to 6.59) | 64.36 (31.83 to 97.84) | 46.50 (24.02 to 71.93) | -1.00 (-1.08 to -0.93) | 205.51 (89.08 to 327.68) | 132.93 (68.13 to 206.81) | 2082.58 (971.45 to 3189.47) | 1243.82 (639.47 to 1917.26) | -1.54 (-1.67 to -1.41) |
| Uganda | 1281.89 (473.71 to 2239.37) | 1107.70 (465.88 to 1859.88) | 11.56 (5.19 to 19.52) | 6.74 (3.00 to 11.16) | -2.42 (-2.80 to -2.03) | 81673.65 (26656.17 to 145807.40) | 51629.45 (19286.92 to 89801.04) | 396.99 (157.32 to 679.65) | 185.91 (79.66 to 311.87) | -3.14 (-3.58 to -2.71) |
| Ukraine | 25994.49 (15333.11 to 38341.02) | 14995.21 (8418.49 to 23855.03) | 40.00 (23.66 to 58.80) | 19.40 (11.01 to 30.73) | -3.03 (-3.67 to -2.40) | 575226.44 (328439.00 to 853119.10) | 324262.00 (183410.57 to 508222.82) | 870.91 (498.53 to 1300.02) | 452.21 (255.90 to 700.87) | -2.72 (-3.28 to -2.16) |
| United Arab Emirates | 145.51 (77.94 to 226.10) | 467.16 (256.38 to 714.72) | 36.30 (19.51 to 56.49) | 22.30 (12.04 to 34.37) | 0.07 (-0.51 to 0.64) | 5724.13 (3062.99 to 8897.70) | 21143.47 (11466.47 to 32093.48) | 881.33 (477.32 to 1377.37) | 480.21 (262.14 to 737.62) | -0.83 (-1.25 to -0.40) |
| United Kingdom | 12281.39 (6855.57 to 17993.84) | 3338.56 (1567.36 to 5436.33) | 14.42 (8.09 to 20.87) | 2.67 (1.26 to 4.33) | -5.61 (-5.87 to -5.35) | 307135.49 (174349.24 to 444463.02) | 97197.13 (44889.58 to 156576.52) | 395.12 (223.74 to 568.39) | 92.66 (42.17 to 147.54) | -4.90 (-5.16 to -4.64) |
| United Republic of Tanzania | 5117.08 (1819.53 to 8644.63) | 2540.88 (1088.89 to 4089.78) | 22.70 (9.50 to 37.13) | 8.82 (4.02 to 14.11) | -3.49 (-3.67 to -3.32) | 368063.61 (122966.27 to 628641.28) | 118987.14 (46737.73 to 202178.44) | 995.18 (365.62 to 1642.15) | 265.78 (113.86 to 429.00) | -4.65 (-4.82 to -4.47) |
| United States of America | 37109.76 (20533.91 to 55207.69) | 24387.29 (12564.30 to 37133.90) | 11.90 (6.59 to 17.63) | 4.26 (2.22 to 6.49) | -3.59 (-3.71 to -3.47) | 970535.23 (520761.81 to 1442716.95) | 694836.83 (347251.73 to 1066080.60) | 331.11 (177.06 to 491.55) | 135.25 (67.91 to 207.45) | -3.12 (-3.23 to -3.01) |
| United States Virgin Islands | 8.14 (4.17 to 13.06) | 9.26 (4.54 to 15.30) | 11.20 (5.61 to 18.00) | 5.56 (2.72 to 9.13) | -2.12 (-2.28 to -1.97) | 245.81 (123.14 to 394.12) | 252.55 (117.63 to 410.66) | 277.12 (137.03 to 444.62) | 163.10 (78.33 to 263.42) | -1.57 (-1.69 to -1.44) |
| Uruguay | 738.04 (382.19 to 1104.41) | 479.02 (212.34 to 757.27) | 19.67 (10.21 to 29.48) | 8.18 (3.62 to 12.86) | -3.33 (-3.59 to -3.08) | 17917.28 (8923.99 to 27283.65) | 10836.56 (4798.73 to 17090.65) | 497.12 (246.87 to 758.24) | 219.13 (95.83 to 348.40) | -3.11 (-3.34 to -2.88) |
| Uzbekistan | 3359.07 (1586.18 to 5416.20) | 5047.90 (2763.17 to 7978.76) | 22.54 (12.00 to 36.05) | 21.96 (12.19 to 35.37) | -0.14 (-0.75 to 0.48) | 172143.02 (69792.16 to 287054.81) | 173817.85 (91351.62 to 269644.99) | 801.18 (377.29 to 1289.20) | 596.17 (317.42 to 917.97) | -0.99 (-1.58 to -0.40) |
| Vanuatu | 23.12 (11.49 to 36.93) | 41.42 (21.69 to 62.88) | 35.33 (18.25 to 54.39) | 25.51 (13.59 to 38.88) | -1.26 (-1.38 to -1.14) | 1070.88 (473.70 to 1741.18) | 1584.97 (789.57 to 2397.93) | 1002.48 (508.98 to 1561.22) | 725.82 (375.03 to 1100.88) | -1.25 (-1.40 to -1.10) |
| Venezuela (Bolivarian Republic of) | 1702.50 (909.06 to 2510.56) | 2880.69 (1351.23 to 4627.71) | 17.29 (9.44 to 25.30) | 10.13 (4.74 to 16.24) | -1.86 (-2.13 to -1.58) | 62253.75 (30147.94 to 93778.53) | 80684.76 (36995.48 to 128745.53) | 473.16 (244.63 to 704.29) | 275.35 (125.55 to 441.25) | -1.78 (-2.04 to -1.51) |
| Viet Nam | 13077.48 (6838.83 to 20511.58) | 18812.86 (9747.08 to 28614.02) | 31.47 (17.10 to 49.63) | 22.12 (11.29 to 33.22) | -1.30 (-1.41 to -1.19) | 509943.21 (234784.53 to 800630.96) | 489129.35 (246461.81 to 762661.21) | 895.44 (448.70 to 1402.24) | 513.45 (252.18 to 787.27) | -1.83 (-1.87 to -1.79) |
| Yemen | 3350.81 (1709.30 to 5196.06) | 4563.15 (2448.58 to 6906.55) | 48.74 (28.68 to 70.21) | 35.22 (19.38 to 53.77) | -1.17 (-1.25 to -1.10) | 187554.85 (78806.14 to 316343.14) | 156299.53 (80214.84 to 236122.64) | 1502.95 (816.39 to 2258.00) | 874.07 (460.60 to 1316.10) | -1.86 (-1.96 to -1.77) |
| Zambia | 1372.67 (506.88 to 2310.66) | 979.23 (418.58 to 1609.79) | 22.46 (9.54 to 36.27) | 12.20 (5.32 to 19.63) | -2.30 (-2.51 to -2.08) | 97771.94 (32597.64 to 170394.49) | 46882.66 (18578.50 to 79780.96) | 929.16 (365.82 to 1520.44) | 366.65 (155.91 to 603.23) | -3.25 (-3.47 to -3.02) |
| Zimbabwe | 973.49 (395.11 to 1630.55) | 1650.83 (701.97 to 2679.08) | 18.91 (8.47 to 31.31) | 21.27 (9.71 to 34.30) | 1.12 (0.48 to 1.77) | 52964.95 (18754.21 to 90313.66) | 76009.00 (30135.97 to 127076.03) | 590.70 (245.77 to 985.26) | 676.61 (288.77 to 1091.65) | 1.16 (0.57 to 1.75) |

ASDR, age-standardized Deaths rates; ASDALYR, age-standardized disability-adjusted life years rates; CI, confidence interval; DALYs, disability-adjusted life years rates; EAPC, estimated annual percentage change; UI, uncertainty interval.

Table S9 Deaths and DALYs of high alcohol use in 1990 and 2021 at regional levels

| Region | Deaths (×1000, 95% UI) | | ASDR per 100,000 (95% UI) | | 1990-2021 EAPC,  (95% CI) | DALYs (×1000, 95% UI) | | ASDALYR per 100,000 (95% UI) | | 1990-2021 EAPC,  (95% CI) |
| --- | --- | --- | --- | --- | --- | --- | --- | --- | --- | --- |
|  | 1990 | 2021 | 1990 | 2021 |  | 1990 | 2021 | 1990 | 2021 |  |
| High SDI | 301.75 (247.91 to 378.37) | 356.96 (300.86 to 430.35) | 28.57 (23.63 to 35.47) | 19.10 (16.55 to 22.24) | -1.33 (-1.37 to -1.28) | 12273.93 (10533.31 to 14236.03) | 13141.93 (11371.87 to 14973.14) | 1220.78 (1049.23 to 1407.33) | 866.86 (752.84 to 994.22) | -1.13 (-1.17 to -1.09) |
| High-middle SDI | 381.52 (299.7 to 487.46) | 443.74 (350.34 to 559.37) | 38.48 (29.76 to 51.10) | 23.76 (18.91 to 29.66) | -2.00 (-2.44 to -1.55) | 15811.74 (13101.19 to 18846.05) | 16705.99 (14106.18 to 19663.9) | 1491.69 (1237.21 to 1784.04) | 964.25 (815.39 to 1126.58) | -1.92 (-2.38 to -1.46) |
| Middle SDI | 323.3 (227.96 to 450.17) | 549.29 (419.91 to 698.2) | 28.13 (19.52 to 39.48) | 20.58 (15.55 to 26.44) | -1.04 (-1.09 to -0.98) | 14731.09 (10982.27 to 19597.08) | 21862.91 (17563.88 to 26880.35) | 1079.09 (809.66 to 1449.66) | 797.53 (640.12 to 981.78) | -1.06 (-1.10 to -1.01) |
| Low-middle SDI | 168.15 (90.07 to 322.53) | 315.94 (213.93 to 480.41) | 24.11 (13.24 to 46.19) | 20.18 (13.59 to 30.80) | -0.45 (-0.54 to -0.36) | 7955.34 (4775.16 to 13853.48) | 14093.63 (9950.58 to 20197.21) | 974.19 (582.50 to 1717.21) | 813.01 (572.07 to 1166.31) | -0.47 (-0.57 to -0.38) |
| Low SDI | 87.85 (36.38 to 186.74) | 141.5 (79.46 to 243.37) | 35.02 (15.18 to 73.63) | 24.86 (14.15 to 43.33) | -1.21 (-1.31 to -1.11) | 3759.99 (1684.9 to 7607.1) | 6374.85 (3805.09 to 10278.92) | 1252.13 (572.04 to 2533.09) | 900.18 (537.65 to 1470.36) | -1.17 (-1.24 to -1.09) |
| Andean Latin America | 8 (4.26 to 13.02) | 11.57 (8.53 to 15.29) | 33.71 (19.25 to 53.83) | 18.92 (14.00 to 24.99) | -1.89 (-2.05 to -1.74) | 376.5 (201.2 to 607.86) | 491.26 (373.61 to 624.42) | 1357.26 (770.52 to 2151.20) | 758.58 (578.73 to 966.43) | -1.89 (-2.03 to -1.75) |
| Australasia | 5.2 (4.24 to 6.68) | 7.7 (6.32 to 9.5) | 22.97 (18.72 to 29.79) | 15.63 (13.09 to 18.78) | -1.07 (-1.17 to -0.97) | 227.81 (192.29 to 268.56) | 318.88 (266.88 to 375.76) | 1025.11 (864.82 to 1207.33) | 801.62 (660.91 to 946.20) | -0.68 (-0.77 to -0.58) |
| Caribbean | 7.49 (5.9 to 9.77) | 12.85 (10.41 to 15.93) | 26.78 (21.18 to 34.68) | 24.24 (19.60 to 30.10) | -0.19 (-0.33 to -0.05) | 353.4 (281.92 to 446.38) | 549.45 (453.35 to 665.55) | 1155.21 (934.97 to 1467.10) | 1060.22 (872.82 to 1283.98) | -0.12 (-0.25 to 0.01) |
| Central Asia | 16.27 (13.09 to 20.35) | 25.61 (21.55 to 30.9) | 31.63 (25.25 to 39.87) | 29.03 (24.33 to 35.30) | -0.56 (-0.97 to -0.16) | 850.4 (693.41 to 1030.45) | 1223.25 (1034.51 to 1445.19) | 1492.59 (1223.64 to 1813.09) | 1274.90 (1077.72 to 1508.22) | -0.92 (-1.33 to -0.51) |
| Central Europe | 66.99 (51.21 to 90.57) | 74.63 (59.82 to 93.3) | 46.78 (35.47 to 64.12) | 37.47 (31.05 to 45.48) | -1.01 (-1.14 to -0.88) | 2609.97 (2174.28 to 3118.36) | 2567.08 (2197.14 to 2985.3) | 1840.71 (1541.80 to 2196.27) | 1501.31 (1300.28 to 1724.76) | -0.93 (-1.05 to -0.81) |
| Central Latin America | 38.56 (32.95 to 45.28) | 60.47 (50.91 to 71.28) | 36.99 (31.98 to 43.71) | 23.23 (19.57 to 27.44) | -1.83 (-2.05 to -1.62) | 2042.76 (1733.58 to 2358.29) | 2972.37 (2536.04 to 3508.12) | 1655.18 (1429.04 to 1909.52) | 1116.82 (953.34 to 1317.92) | -1.52 (-1.71 to -1.33) |
| Central Sub-Saharan Africa | 14.52 (4.26 to 32.34) | 27.66 (11.46 to 54.48) | 56.73 (17.39 to 127.31) | 41.64 (17.52 to 83.48) | -0.72 (-1.09 to -0.34) | 609.77 (192.4 to 1326.53) | 1230.46 (532.3 to 2315.82) | 1938.35 (628.17 to 4225.92) | 1470.46 (660.12 to 2808.57) | -0.62 (-0.95 to -0.29) |
| East Asia | 284.27 (192.31 to 397.99) | 394.84 (254.44 to 562.75) | 32.02 (21.05 to 45.43) | 19.08 (12.39 to 27.10) | -1.67 (-1.78 to -1.56) | 11505.88 (8465.2 to 15464.04) | 13700.62 (9964.68 to 18230.46) | 1097.84 (793.17 to 1474.32) | 692.83 (514.61 to 906.46) | -1.57 (-1.70 to -1.44) |
| Eastern Europe | 135.19 (112.08 to 173.39) | 162.92 (141.12 to 195.14) | 51.51 (42.80 to 65.99) | 54.50 (47.46 to 64.29) | -0.77 (-1.79 to 0.25) | 6343.74 (5429.94 to 7445.7) | 6878.01 (6033.27 to 7889.61) | 2473.49 (2126.31 to 2885.78) | 2510.40 (2217.99 to 2824.00) | -0.89 (-1.84 to 0.07) |
| Eastern Sub-Saharan Africa | 44.96 (14.76 to 98.28) | 70.85 (37.27 to 119.69) | 54.72 (19.83 to 118.60) | 37.19 (20.18 to 62.73) | -1.50 (-1.60 to -1.41) | 1868.01 (661.82 to 3902.59) | 3150.19 (1750.64 to 5121.46) | 1837.44 (690.84 to 3835.01) | 1272.03 (720.63 to 2056.42) | -1.41 (-1.49 to -1.32) |
| High-income Asia Pacific | 58.59 (45.35 to 74.84) | 53.07 (40.64 to 69.43) | 29.74 (22.49 to 38.53) | 12.92 (10.63 to 15.83) | -2.97 (-3.06 to -2.87) | 2226.5 (1851.13 to 2655.45) | 1670.9 (1378.28 to 2018.44) | 1114.12 (927.69 to 1332.76) | 569.63 (476.18 to 676.51) | -2.33 (-2.39 to -2.27) |
| High-income North America | 67.31 (59.63 to 79.76) | 122.07 (105.9 to 141.05) | 20.57 (18.21 to 24.09) | 21.72 (19.10 to 24.61) | 0.38 (0.23 to 0.53) | 3418.05 (2958 to 3928.05) | 4984.29 (4361.76 to 5635.76) | 1099.75 (949.65 to 1266.65) | 1049.25 (919.44 to 1189.38) | 0.07 (-0.08 to 0.22) |
| North Africa and Middle East | 8.96 (6.79 to 12.25) | 13.18 (10.49 to 16.73) | 4.97 (3.72 to 6.86) | 2.85 (2.27 to 3.61) | -1.86 (-1.94 to -1.78) | 450.74 (349.52 to 599.59) | 702.39 (575.18 to 859.28) | 193.96 (154.39 to 255.69) | 123.77 (102.91 to 151.14) | -1.47 (-1.52 to -1.41) |
| Oceania | 0.53 (0.24 to 1.09) | 0.89 (0.49 to 1.69) | 14.07 (6.46 to 30.06) | 9.58 (5.20 to 18.53) | -1.06 (-1.34 to -0.78) | 27.98 (14.05 to 52.12) | 50.38 (31.62 to 85.23) | 599.70 (301.26 to 1140.38) | 443.95 (273.82 to 763.58) | -0.83 (-1.06 to -0.60) |
| South Asia | 148.6 (74.25 to 314.23) | 284.74 (191.13 to 442.22) | 21.75 (11.10 to 46.02) | 17.85 (11.86 to 27.91) | -0.52 (-0.63 to -0.41) | 7417.76 (4267.63 to 13940.18) | 12925.96 (9115.5 to 18826.21) | 931.62 (535.75 to 1774.13) | 737.40 (519.73 to 1079.74) | -0.67 (-0.75 to -0.60) |
| Southeast Asia | 61.9 (40.61 to 98.75) | 156.72 (112.88 to 210.45) | 21.15 (13.99 to 33.82) | 23.14 (16.47 to 31.26) | 0.38 (0.27 to 0.49) | 2759.09 (1847.31 to 4204.38) | 6049.56 (4460.15 to 8004.33) | 796.60 (539.08 to 1212.24) | 825.73 (612.11 to 1095.31) | 0.13 (0.05 to 0.21) |
| Southern Latin America | 18.79 (14.99 to 23.4) | 16.18 (13.37 to 19.61) | 40.74 (32.39 to 51.33) | 19.19 (15.99 to 23.10) | -2.08 (-2.20 to -1.96) | 761.72 (637.46 to 894.9) | 696.81 (590.59 to 811.75) | 1606.19 (1344.63 to 1888.71) | 891.49 (756.20 to 1043.77) | -1.68 (-1.76 to -1.61) |
| Southern Sub-Saharan Africa | 17.37 (7.25 to 30.62) | 28.25 (14.08 to 46.75) | 52.20 (23.01 to 92.46) | 43.57 (22.95 to 72.06) | -0.75 (-1.21 to -0.28) | 866.88 (400.72 to 1442.01) | 1300.83 (686.54 to 2081.42) | 2220.84 (1026.33 to 3737.06) | 1752.87 (946.80 to 2791.24) | -0.90 (-1.32 to -0.47) |
| Tropical Latin America | 34.16 (28.37 to 41.26) | 54.78 (47.27 to 63.92) | 30.97 (25.11 to 38.53) | 21.28 (18.29 to 24.89) | -1.20 (-1.43 to -0.97) | 1908.42 (1603.55 to 2217.95) | 2797.42 (2395.47 to 3210.24) | 1488.67 (1253.56 to 1737.11) | 1091.32 (933.41 to 1259.16) | -1.02 (-1.25 to -0.78) |
| Western Europe | 188.43 (148.1 to 247.94) | 163.46 (134.41 to 199.49) | 34.88 (28.05 to 44.75) | 18.62 (15.93 to 21.93) | -2.04 (-2.09 to -2.00) | 6587.71 (5581.16 to 7686.31) | 5366.14 (4627.3 to 6226.98) | 1372.01 (1173.33 to 1576.01) | 826.78 (708.71 to 967.51) | -1.71 (-1.77 to -1.66) |
| Western Sub-Saharan Africa | 38.12 (19.2 to 69.93) | 66.99 (41.63 to 104.89) | 41.80 (20.97 to 76.74) | 31.69 (18.89 to 50.53) | -1.00 (-1.15 to -0.85) | 1385.64 (697.69 to 2504.14) | 2627.99 (1686.23 to 4055.33) | 1282.34 (658.01 to 2316.49) | 977.89 (618.79 to 1513.71) | -0.97 (-1.11 to -0.83) |

ASDR, age-standardized Deaths rates; ASDALYR, age-standardized disability-adjusted life years rates; CI, confidence interval; DALYs, disability-adjusted life years rates; EAPC, estimated annual percentage change; UI, uncertainty interval.

Table S10 Deaths and DALYs of high alcohol use in 1990 and 2021 at national levels

| Location | Deaths (95% UI) | | ASDR per 100,000 (95% UI) | | 1990-2021 EAPC,  (95% CI) | DALYs (95% UI) | | ASDALYR per 100,000 (95% UI) | | 1990-2021 EAPC,  (95% CI) |
| --- | --- | --- | --- | --- | --- | --- | --- | --- | --- | --- |
|  | 1990 | 2021 | 1990 | 2021 |  | 1990 | 2021 | 1990 | 2021 |  |
| Afghanistan | 224.61 (135.71 to 350.76) | 296.18 (181.28 to 454.92) | 3.31 (2.05 to 5.11) | 2.57 (1.67 to 3.75) | -0.54 (-0.80 to -0.28) | 9820.21 (6956.50 to 13598.91) | 21975.58 (15978.91 to 30674.85) | 134.64 (95.67 to 185.03) | 118.12 (88.69 to 164.05) | -0.09 (-0.35 to 0.16) |
| Albania | 312.57 (219.35 to 442.82) | 624.65 (371.57 to 956.90) | 15.21 (10.22 to 22.41) | 14.93 (9.07 to 22.79) | 0.06 (-0.34 to 0.46) | 15371.12 (11842.62 to 19177.35) | 21089.88 (15600.59 to 28103.14) | 596.71 (453.22 to 760.54) | 573.00 (434.32 to 754.09) | -0.13 (-0.51 to 0.26) |
| Algeria | 330.63 (222.07 to 549.52) | 804.63 (589.84 to 1155.62) | 2.56 (1.76 to 4.21) | 2.37 (1.71 to 3.37) | -0.03 (-0.14 to 0.09) | 22888.74 (16782.66 to 32627.36) | 46898.60 (36866.29 to 60050.96) | 126.25 (93.26 to 181.62) | 111.93 (88.16 to 143.39) | -0.35 (-0.41 to -0.30) |
| American Samoa | 1.61 (1.11 to 2.47) | 2.12 (1.55 to 3.00) | 5.46 (3.75 to 8.42) | 4.18 (3.05 to 6.03) | -1.01 (-1.30 to -0.72) | 106.14 (77.36 to 150.83) | 118.99 (90.97 to 160.02) | 291.37 (217.44 to 409.43) | 233.00 (178.08 to 311.69) | -0.86 (-1.10 to -0.61) |
| Andorra | 16.52 (11.67 to 22.87) | 24.18 (16.47 to 32.83) | 29.19 (20.18 to 40.78) | 16.13 (10.99 to 21.83) | -1.79 (-1.94 to -1.63) | 710.98 (550.12 to 931.95) | 927.35 (696.56 to 1168.76) | 1164.77 (894.71 to 1529.65) | 752.62 (571.30 to 947.19) | -1.37 (-1.45 to -1.29) |
| Angola | 2929.10 (942.42 to 6373.25) | 7612.97 (3715.85 to 13149.19) | 61.01 (21.38 to 135.06) | 52.49 (26.84 to 89.85) | -0.06 (-0.44 to 0.33) | 126324.07 (43432.30 to 266866.74) | 338126.24 (168967.30 to 575170.07) | 2151.92 (760.84 to 4571.72) | 1842.64 (936.96 to 3073.62) | -0.08 (-0.44 to 0.28) |
| Antigua and Barbuda | 10.81 (8.98 to 13.34) | 25.72 (22.27 to 29.50) | 20.78 (17.33 to 25.36) | 23.91 (20.49 to 27.76) | 0.57 (0.39 to 0.74) | 497.50 (414.37 to 589.19) | 1093.46 (942.61 to 1258.51) | 939.23 (787.26 to 1114.38) | 1004.71 (867.65 to 1159.61) | 0.42 (0.32 to 0.53) |
| Argentina | 12556.24 (9840.12 to 15961.09) | 9518.52 (7714.75 to 11744.88) | 39.42 (30.71 to 50.46) | 17.53 (14.35 to 21.48) | -2.23 (-2.39 to -2.07) | 497807.66 (409233.18 to 591518.16) | 420786.15 (351396.03 to 496838.42) | 1545.50 (1268.86 to 1841.84) | 823.82 (687.72 to 973.40) | -1.82 (-1.93 to -1.71) |
| Armenia | 548.26 (446.48 to 693.27) | 611.44 (507.59 to 747.51) | 19.09 (15.59 to 24.28) | 14.88 (12.34 to 18.09) | -0.69 (-1.34 to -0.04) | 29812.23 (24321.41 to 36082.98) | 26300.65 (22137.82 to 31352.45) | 933.32 (767.93 to 1121.73) | 704.96 (594.56 to 849.99) | -1.03 (-1.42 to -0.63) |
| Australia | 4276.35 (3490.87 to 5493.31) | 6608.10 (5434.18 to 8095.67) | 22.69 (18.50 to 29.43) | 15.98 (13.47 to 19.02) | -0.94 (-1.04 to -0.85) | 184837.58 (156221.87 to 217612.00) | 263653.75 (222815.25 to 309634.74) | 997.23 (840.85 to 1176.21) | 787.43 (654.33 to 926.31) | -0.65 (-0.72 to -0.59) |
| Austria | 5086.42 (4135.49 to 6487.75) | 3604.68 (3045.78 to 4296.71) | 46.28 (38.36 to 57.79) | 21.25 (18.47 to 24.53) | -2.32 (-2.43 to -2.20) | 170189.81 (145446.81 to 196745.21) | 128085.87 (110860.39 to 148778.86) | 1739.01 (1496.92 to 1995.90) | 970.78 (833.12 to 1140.61) | -1.80 (-1.86 to -1.74) |
| Azerbaijan | 1662.14 (1242.81 to 2191.74) | 2520.03 (1958.42 to 3246.55) | 30.61 (23.29 to 40.01) | 24.52 (19.07 to 31.61) | -0.83 (-1.05 to -0.62) | 84206.78 (62982.74 to 109258.90) | 114949.68 (91095.89 to 142894.87) | 1345.74 (1010.83 to 1730.34) | 997.02 (793.05 to 1235.40) | -1.33 (-1.52 to -1.15) |
| Bahamas | 71.39 (60.26 to 85.00) | 125.74 (92.16 to 166.07) | 39.55 (33.15 to 47.49) | 29.43 (21.59 to 39.01) | -0.82 (-1.05 to -0.59) | 3494.69 (2924.71 to 4171.17) | 5776.17 (4398.92 to 7402.10) | 1659.71 (1409.07 to 1965.41) | 1310.75 (999.86 to 1685.51) | -0.59 (-0.78 to -0.40) |
| Bahrain | 19.59 (15.04 to 25.20) | 32.36 (25.25 to 44.26) | 9.85 (7.48 to 12.99) | 3.43 (2.67 to 4.49) | -4.24 (-4.52 to -3.95) | 1073.24 (859.67 to 1322.37) | 2185.47 (1728.35 to 2783.51) | 326.69 (258.56 to 409.24) | 143.85 (115.54 to 181.92) | -3.31 (-3.55 to -3.08) |
| Bangladesh | 5971.27 (4163.34 to 8224.03) | 8998.12 (5862.57 to 13022.66) | 11.92 (8.41 to 16.54) | 6.63 (4.48 to 9.53) | -2.09 (-2.33 to -1.85) | 303407.35 (226086.00 to 391635.74) | 468518.59 (341094.88 to 638197.53) | 481.32 (356.45 to 628.84) | 300.04 (220.13 to 410.61) | -1.65 (-1.79 to -1.52) |
| Barbados | 62.36 (50.26 to 78.71) | 99.35 (73.35 to 133.28) | 22.73 (18.56 to 28.11) | 20.83 (15.56 to 27.69) | -0.20 (-0.36 to -0.05) | 2493.93 (2135.95 to 2899.22) | 3571.45 (2857.69 to 4478.02) | 957.00 (822.79 to 1107.25) | 863.13 (690.72 to 1080.44) | -0.22 (-0.32 to -0.12) |
| Belarus | 5515.87 (4645.16 to 7044.39) | 6785.06 (5455.11 to 8410.12) | 45.48 (38.67 to 57.47) | 48.43 (39.18 to 59.52) | -0.43 (-1.11 to 0.26) | 256140.59 (219571.45 to 303672.84) | 277025.61 (226895.06 to 332304.51) | 2187.91 (1888.03 to 2570.80) | 2182.97 (1795.58 to 2608.72) | -0.68 (-1.33 to -0.03) |
| Belgium | 4497.84 (3403.44 to 5972.97) | 4130.34 (3318.21 to 5069.87) | 31.62 (24.37 to 41.05) | 19.62 (16.37 to 23.08) | -1.36 (-1.50 to -1.22) | 162476.51 (134091.66 to 193568.85) | 151071.64 (128712.22 to 177945.72) | 1304.93 (1089.27 to 1530.54) | 934.85 (785.27 to 1111.53) | -1.11 (-1.26 to -0.96) |
| Belize | 22.57 (17.66 to 28.89) | 88.06 (73.96 to 104.47) | 21.23 (16.69 to 26.80) | 25.10 (21.02 to 29.83) | 0.63 (0.27 to 0.98) | 1167.56 (912.84 to 1461.30) | 4469.97 (3775.09 to 5292.84) | 938.96 (747.62 to 1168.53) | 1126.14 (956.82 to 1326.67) | 0.70 (0.41 to 0.98) |
| Benin | 586.18 (305.46 to 1065.94) | 1338.73 (829.56 to 2137.48) | 27.58 (14.72 to 49.80) | 22.33 (13.93 to 35.70) | -0.66 (-0.79 to -0.52) | 21902.32 (11311.42 to 40193.07) | 56059.93 (35494.06 to 88034.96) | 892.05 (470.61 to 1614.50) | 756.92 (484.31 to 1191.20) | -0.49 (-0.63 to -0.35) |
| Bermuda | 17.06 (14.24 to 20.90) | 18.71 (14.56 to 24.21) | 26.96 (22.41 to 33.35) | 15.24 (11.86 to 19.48) | -1.75 (-2.14 to -1.35) | 716.40 (614.83 to 844.93) | 657.72 (540.05 to 810.14) | 1084.26 (931.93 to 1277.31) | 675.89 (552.14 to 830.01) | -1.43 (-1.72 to -1.13) |
| Bhutan | 96.73 (36.41 to 193.85) | 58.78 (36.58 to 94.39) | 30.94 (12.38 to 62.45) | 9.15 (5.84 to 14.50) | -4.91 (-5.32 to -4.49) | 5170.61 (2585.19 to 9103.39) | 3803.98 (2758.02 to 5276.99) | 1309.75 (608.95 to 2371.04) | 509.21 (367.55 to 706.19) | -3.75 (-4.03 to -3.46) |
| Bolivia (Plurinational State of) | 1724.36 (728.87 to 3075.58) | 2557.92 (1604.10 to 3707.65) | 47.30 (20.27 to 83.29) | 26.89 (16.69 to 38.61) | -1.78 (-1.95 to -1.60) | 75896.80 (34907.53 to 131816.10) | 104027.52 (72768.22 to 141892.94) | 1773.57 (847.40 to 3016.03) | 981.30 (685.28 to 1344.07) | -1.91 (-2.11 to -1.71) |
| Bosnia and Herzegovina | 1195.06 (926.99 to 1544.73) | 1433.55 (983.57 to 2022.89) | 27.59 (20.70 to 36.69) | 24.32 (16.96 to 33.81) | -0.64 (-0.86 to -0.43) | 56426.49 (45049.75 to 69676.27) | 48074.28 (36578.64 to 60598.45) | 1203.47 (958.28 to 1484.52) | 947.71 (732.00 to 1166.34) | -0.98 (-1.15 to -0.82) |
| Botswana | 340.37 (85.33 to 783.23) | 440.42 (206.84 to 806.33) | 50.48 (15.44 to 114.55) | 24.84 (12.73 to 45.38) | -2.72 (-3.19 to -2.23) | 16155.18 (4763.34 to 34809.99) | 23759.33 (13012.20 to 39002.01) | 1985.65 (625.18 to 4279.29) | 1098.55 (618.79 to 1806.74) | -2.33 (-2.77 to -1.88) |
| Brazil | 33479.47 (27825.00 to 40396.14) | 53062.69 (45808.29 to 61993.22) | 31.00 (25.17 to 38.53) | 21.14 (18.22 to 24.77) | -1.23 (-1.46 to -0.99) | 1872930.04 (1576647.97 to 2176020.29) | 2710367.10 (2321799.90 to 3108244.90) | 1494.80 (1261.38 to 1741.68) | 1086.94 (930.51 to 1254.76) | -1.04 (-1.28 to -0.80) |
| Brunei Darussalam | 11.69 (8.37 to 17.69) | 15.10 (11.06 to 19.90) | 9.41 (6.74 to 14.59) | 3.79 (2.79 to 4.92) | -1.97 (-2.50 to -1.44) | 721.97 (549.03 to 964.23) | 982.52 (750.27 to 1248.90) | 398.80 (309.60 to 550.04) | 202.00 (156.23 to 253.04) | -1.48 (-1.90 to -1.07) |
| Bulgaria | 5586.90 (3781.63 to 8064.03) | 7456.34 (4953.85 to 10494.78) | 51.14 (34.44 to 75.44) | 56.26 (38.78 to 76.97) | 0.39 (0.26 to 0.52) | 196117.62 (149641.41 to 254553.95) | 204540.78 (155337.72 to 263162.22) | 1784.91 (1382.51 to 2303.31) | 1820.87 (1437.11 to 2287.77) | -0.01 (-0.14 to 0.11) |
| Burkina Faso | 2262.12 (1176.96 to 3863.49) | 4457.82 (2670.28 to 7172.24) | 51.81 (26.81 to 88.83) | 45.46 (27.78 to 71.69) | -0.36 (-0.50 to -0.23) | 79946.83 (40119.23 to 135294.21) | 167428.01 (101397.13 to 264320.30) | 1549.18 (791.78 to 2643.51) | 1369.52 (840.34 to 2160.87) | -0.34 (-0.46 to -0.22) |
| Burundi | 3261.89 (771.51 to 6874.37) | 3099.99 (1118.18 to 6236.25) | 126.74 (33.84 to 268.68) | 54.36 (20.48 to 108.06) | -3.37 (-3.62 to -3.12) | 126900.70 (28350.65 to 267123.51) | 134587.97 (51555.29 to 262819.68) | 4139.39 (1010.85 to 8611.91) | 1822.04 (720.28 to 3520.80) | -3.23 (-3.46 to -3.00) |
| Côte d'Ivoire | 63.27 (35.49 to 101.66) | 152.79 (100.98 to 218.82) | 27.58 (15.49 to 44.10) | 31.87 (20.66 to 45.97) | -0.87 (-1.06 to -0.69) | 2377.94 (1361.24 to 3734.28) | 5720.33 (4025.77 to 7922.06) | 1016.25 (588.09 to 1586.50) | 1087.41 (746.40 to 1511.34) | -0.87 (-1.06 to -0.68) |
| Cabo Verde | 1713.96 (798.54 to 3296.83) | 6718.33 (3781.30 to 10692.34) | 33.14 (15.83 to 64.07) | 50.40 (28.58 to 79.90) | -0.05 (-0.32 to 0.22) | 71792.17 (33174.62 to 139271.79) | 263652.99 (148280.03 to 417730.68) | 1173.67 (560.36 to 2223.94) | 1738.57 (974.28 to 2739.85) | -0.11 (-0.29 to 0.07) |
| Cambodia | 2289.28 (1007.84 to 4026.70) | 6687.90 (3907.78 to 10358.64) | 48.61 (21.48 to 85.51) | 47.45 (27.15 to 73.68) | 0.92 (0.69 to 1.16) | 86812.36 (38457.22 to 150373.01) | 269136.34 (164943.61 to 407847.90) | 1519.08 (687.48 to 2645.27) | 1490.32 (898.38 to 2278.47) | 0.81 (0.59 to 1.04) |
| Cameroon | 5785.50 (4876.44 to 7092.17) | 10374.37 (8840.70 to 12407.38) | 18.40 (15.49 to 22.52) | 16.54 (14.24 to 19.17) | 0.24 (0.04 to 0.43) | 263306.76 (219926.60 to 307983.63) | 404614.31 (346667.93 to 466997.58) | 862.62 (720.16 to 1007.12) | 802.70 (685.19 to 929.38) | 0.23 (0.03 to 0.43) |
| Canada | 1403.85 (315.14 to 3319.70) | 1647.91 (401.02 to 4262.02) | 104.04 (24.27 to 245.93) | 58.33 (15.12 to 149.75) | -0.13 (-0.24 to -0.01) | 57576.49 (13403.52 to 133489.15) | 72287.73 (19190.40 to 179184.17) | 3541.62 (857.12 to 8206.02) | 2064.98 (579.22 to 5135.73) | -0.08 (-0.18 to 0.01) |
| Central African Republic | 678.41 (304.65 to 1648.55) | 2671.93 (1035.66 to 5758.53) | 22.95 (10.44 to 55.56) | 39.89 (15.77 to 85.62) | -1.76 (-1.82 to -1.69) | 24847.75 (11300.61 to 59940.06) | 108259.95 (42592.31 to 230269.06) | 749.62 (348.20 to 1767.04) | 1295.33 (520.43 to 2731.00) | -1.64 (-1.71 to -1.56) |
| Chad | 5071.46 (4105.14 to 6149.99) | 5560.39 (4594.31 to 6642.26) | 47.36 (37.97 to 58.20) | 22.39 (18.65 to 26.60) | 2.30 (1.97 to 2.63) | 223119.51 (187148.33 to 262071.40) | 236693.79 (201433.10 to 278443.98) | 1888.46 (1587.45 to 2220.40) | 1037.99 (876.77 to 1227.41) | 2.31 (1.97 to 2.65) |
| Chile | 272586.72 (184374.36 to 383621.06) | 381044.18 (243540.46 to 546229.52) | 31.95 (21.11 to 45.60) | 19.10 (12.31 to 27.29) | -1.93 (-2.07 to -1.78) | 11048871.17 (8150625.07 to 14856392.09) | 13188837.96 (9595141.41 to 17548362.29) | 1094.32 (789.26 to 1472.09) | 690.98 (511.98 to 903.06) | -1.57 (-1.69 to -1.45) |
| China | 5588.65 (4021.52 to 7150.96) | 4965.89 (3868.72 to 6316.23) | 21.83 (16.62 to 27.78) | 9.10 (7.09 to 11.56) | -1.65 (-1.76 to -1.54) | 371308.04 (283506.35 to 469526.15) | 346645.07 (273722.44 to 432065.87) | 1239.99 (965.02 to 1532.82) | 638.86 (503.85 to 795.03) | -1.56 (-1.69 to -1.43) |
| Colombia | 27.67 (15.65 to 53.84) | 59.44 (36.76 to 113.79) | 13.25 (7.79 to 25.27) | 11.62 (7.09 to 21.89) | -3.46 (-3.74 to -3.18) | 1330.26 (857.42 to 2388.80) | 2813.26 (1875.24 to 4863.80) | 508.29 (330.15 to 908.98) | 458.11 (311.60 to 790.73) | -2.64 (-2.86 to -2.43) |
| Comoros | 766.36 (285.42 to 1593.49) | 1695.73 (824.89 to 2836.80) | 64.13 (24.21 to 135.65) | 55.05 (27.89 to 90.71) | -0.51 (-0.63 to -0.38) | 32002.27 (12064.95 to 66010.31) | 72487.04 (35948.49 to 117999.92) | 2226.42 (856.77 to 4579.98) | 1845.47 (950.33 to 3015.81) | -0.41 (-0.55 to -0.27) |
| Congo | 1.05 (0.53 to 2.01) | 6.41 (4.63 to 8.43) | 7.52 (3.80 to 14.68) | 26.59 (19.28 to 35.00) | -0.10 (-0.57 to 0.37) | 61.70 (37.70 to 103.48) | 237.03 (175.27 to 310.66) | 388.20 (238.72 to 648.96) | 1074.58 (804.31 to 1399.95) | -0.27 (-0.69 to 0.15) |
| Cook Islands | 409.98 (343.76 to 492.37) | 922.29 (772.26 to 1079.97) | 20.68 (17.19 to 25.11) | 16.92 (14.17 to 19.80) | 5.35 (4.73 to 5.97) | 22135.66 (18650.26 to 25971.97) | 44262.66 (37903.31 to 52162.32) | 938.50 (799.86 to 1104.51) | 825.38 (706.26 to 971.25) | 4.34 (3.90 to 4.79) |
| Costa Rica | 2436.44 (1038.43 to 4335.17) | 5671.82 (3384.61 to 8743.74) | 53.14 (22.46 to 94.88) | 45.06 (25.88 to 71.04) | -1.42 (-1.81 to -1.04) | 101439.69 (43714.32 to 177989.32) | 232214.42 (140487.16 to 358516.45) | 1676.64 (732.86 to 2958.16) | 1417.96 (856.19 to 2130.36) | -1.01 (-1.27 to -0.74) |
| Croatia | 3225.51 (2440.06 to 4362.16) | 2327.75 (1845.53 to 3023.71) | 55.12 (40.69 to 76.45) | 28.14 (22.79 to 35.56) | -2.36 (-2.58 to -2.13) | 118284.61 (96503.16 to 143071.23) | 75139.01 (62434.88 to 90920.03) | 1996.21 (1628.42 to 2432.58) | 1104.16 (927.87 to 1315.30) | -2.01 (-2.17 to -1.85) |
| Cuba | 1545.80 (1250.76 to 1965.83) | 3820.23 (3149.20 to 4587.39) | 14.49 (11.72 to 18.66) | 20.85 (17.34 to 24.87) | 1.39 (1.15 to 1.64) | 79541.57 (65043.42 to 94703.48) | 142975.93 (121881.97 to 165612.24) | 707.67 (581.30 to 841.96) | 879.34 (756.43 to 1028.65) | 0.87 (0.68 to 1.06) |
| Cyprus | 193.87 (143.33 to 261.63) | 222.96 (166.58 to 295.72) | 30.24 (20.78 to 43.68) | 11.87 (8.84 to 15.89) | -2.98 (-3.14 to -2.83) | 7415.38 (5959.44 to 9044.36) | 9256.69 (7383.82 to 11528.73) | 963.55 (768.22 to 1193.64) | 538.89 (426.62 to 675.62) | -1.93 (-2.14 to -1.72) |
| Czechia | 6077.89 (3994.91 to 9039.02) | 5106.08 (4174.07 to 6353.23) | 46.15 (30.85 to 68.03) | 26.91 (22.31 to 32.64) | -1.88 (-2.07 to -1.69) | 215042.56 (169172.32 to 274967.32) | 189794.07 (159462.30 to 224393.96) | 1737.42 (1384.53 to 2182.81) | 1204.66 (1012.68 to 1415.59) | -1.17 (-1.28 to -1.06) |
| Democratic People's Republic of Korea | 5307.17 (2255.62 to 9531.35) | 7577.56 (3693.78 to 12735.03) | 30.65 (13.18 to 55.87) | 22.86 (11.09 to 38.70) | -0.89 (-0.94 to -0.84) | 211233.89 (94358.80 to 363089.95) | 285133.28 (150603.53 to 467341.06) | 1091.64 (498.83 to 1876.12) | 865.35 (459.40 to 1410.79) | -0.73 (-0.76 to -0.70) |
| Democratic Republic of the Congo | 8734.19 (2312.14 to 20509.98) | 15821.74 (4939.24 to 34739.98) | 50.06 (13.41 to 118.78) | 35.76 (11.25 to 80.39) | -0.87 (-1.66 to -0.08) | 368342.93 (110423.25 to 824038.10) | 710645.46 (263340.72 to 1486042.99) | 1700.24 (514.57 to 3852.53) | 1275.28 (455.71 to 2721.36) | -0.72 (-1.43 to 0.00) |
| Denmark | 2266.29 (1772.75 to 3155.54) | 2395.56 (1961.75 to 2947.25) | 31.30 (25.06 to 41.75) | 21.83 (18.69 to 25.94) | -1.35 (-1.80 to -0.91) | 90292.44 (76452.36 to 108265.85) | 83303.59 (71634.53 to 97734.40) | 1402.39 (1189.26 to 1649.57) | 967.35 (829.73 to 1147.94) | -1.33 (-1.70 to -0.97) |
| Djibouti | 40.96 (14.82 to 93.20) | 75.15 (48.86 to 112.86) | 24.27 (10.21 to 53.03) | 10.49 (6.99 to 15.45) | -3.29 (-3.60 to -2.98) | 2094.68 (912.16 to 4424.01) | 4234.96 (2934.45 to 5823.11) | 906.29 (414.70 to 1890.98) | 438.63 (316.04 to 602.37) | -2.87 (-3.14 to -2.59) |
| Dominica | 18.55 (14.62 to 23.52) | 22.50 (17.50 to 28.18) | 31.23 (24.62 to 39.61) | 27.26 (21.20 to 34.18) | -0.38 (-0.54 to -0.21) | 749.32 (614.99 to 913.37) | 921.06 (747.86 to 1115.21) | 1230.74 (1011.08 to 1496.55) | 1150.86 (931.98 to 1391.37) | -0.11 (-0.25 to 0.04) |
| Dominican Republic | 1212.44 (837.32 to 1670.96) | 2391.91 (1659.34 to 3249.76) | 27.92 (20.27 to 37.63) | 22.82 (15.78 to 30.96) | -0.44 (-0.56 to -0.32) | 61374.08 (40693.76 to 83644.20) | 110230.27 (83519.22 to 139201.89) | 1141.89 (800.95 to 1524.49) | 997.29 (753.59 to 1260.46) | -0.16 (-0.29 to -0.03) |
| Ecuador | 1725.88 (1268.01 to 2415.97) | 2531.57 (1837.96 to 3332.56) | 27.52 (21.02 to 37.84) | 14.95 (10.89 to 19.72) | -1.51 (-1.99 to -1.04) | 87699.81 (63974.11 to 119645.85) | 119428.77 (92455.35 to 149483.55) | 1193.62 (890.71 to 1597.85) | 670.04 (519.67 to 839.72) | -1.49 (-1.94 to -1.03) |
| Egypt | 1571.55 (1173.91 to 2082.22) | 3084.22 (2167.75 to 4254.35) | 6.77 (4.92 to 9.25) | 4.99 (3.54 to 6.90) | -0.59 (-0.74 to -0.44) | 65415.83 (50929.73 to 83780.08) | 128445.40 (100197.08 to 166550.33) | 194.89 (151.38 to 246.31) | 163.17 (127.30 to 210.03) | -0.19 (-0.36 to -0.02) |
| El Salvador | 1788.75 (1434.39 to 2112.23) | 2050.45 (1609.55 to 2663.21) | 50.13 (39.36 to 59.10) | 32.88 (25.79 to 42.69) | -1.67 (-1.98 to -1.36) | 98937.12 (81789.18 to 115177.43) | 111617.97 (90272.23 to 136825.95) | 2497.41 (2057.73 to 2895.13) | 1761.76 (1424.51 to 2164.86) | -1.32 (-1.58 to -1.06) |
| Equatorial Guinea | 140.53 (35.70 to 376.10) | 248.22 (138.62 to 399.33) | 64.38 (16.21 to 169.91) | 38.73 (21.99 to 60.92) | -1.97 (-2.29 to -1.65) | 5651.56 (1540.81 to 14619.41) | 11953.49 (7072.22 to 18594.35) | 2222.63 (620.43 to 5776.96) | 1329.55 (778.87 to 2057.85) | -2.00 (-2.31 to -1.68) |
| Eritrea | 697.28 (190.06 to 1791.93) | 870.85 (320.54 to 2034.68) | 46.02 (15.02 to 112.92) | 24.60 (10.47 to 54.71) | -2.99 (-3.31 to -2.67) | 32907.26 (9328.70 to 82415.01) | 43813.52 (18023.32 to 93893.50) | 1718.32 (558.87 to 4171.36) | 958.65 (422.64 to 2031.12) | -2.84 (-3.15 to -2.52) |
| Estonia | 788.14 (648.77 to 1051.16) | 859.08 (676.18 to 1099.76) | 42.32 (35.10 to 55.40) | 38.96 (32.12 to 47.40) | -1.14 (-1.83 to -0.45) | 37108.68 (31301.10 to 44732.52) | 30200.54 (25330.72 to 35736.42) | 2095.06 (1777.85 to 2491.66) | 1643.41 (1407.11 to 1914.97) | -1.65 (-2.29 to -1.02) |
| Eswatini | 205.10 (85.45 to 409.69) | 385.13 (174.49 to 706.66) | 60.87 (27.51 to 118.96) | 56.12 (28.06 to 101.09) | 0.02 (-0.66 to 0.71) | 9187.47 (4288.30 to 17531.38) | 17912.79 (8771.07 to 31982.87) | 2174.24 (1020.04 to 4164.04) | 2107.68 (1047.51 to 3732.26) | 0.17 (-0.49 to 0.84) |
| Ethiopia | 11393.17 (2809.58 to 31303.33) | 14278.69 (7085.08 to 26308.15) | 48.71 (13.32 to 132.90) | 28.60 (14.14 to 53.41) | -2.09 (-2.55 to -1.62) | 520560.14 (169456.39 to 1307082.23) | 734692.77 (432203.23 to 1208889.20) | 1850.85 (615.03 to 4677.38) | 1106.61 (645.61 to 1873.59) | -1.85 (-2.23 to -1.46) |
| Fiji | 39.60 (24.75 to 58.42) | 79.14 (48.06 to 118.84) | 8.34 (4.90 to 12.50) | 9.58 (5.82 to 14.57) | 0.41 (0.09 to 0.74) | 2327.32 (1598.21 to 3192.19) | 3828.45 (2612.62 to 5324.34) | 386.68 (259.01 to 541.14) | 424.10 (289.72 to 590.49) | 0.31 (0.06 to 0.57) |
| Finland | 1805.05 (1401.30 to 2414.45) | 2295.64 (1915.35 to 2829.51) | 28.18 (22.23 to 36.86) | 22.60 (19.61 to 26.47) | -0.42 (-0.83 to -0.00) | 82413.99 (68990.89 to 97653.76) | 83250.78 (71749.24 to 96681.65) | 1383.23 (1153.49 to 1626.90) | 1069.43 (920.08 to 1240.66) | -0.68 (-1.08 to -0.28) |
| France | 34550.33 (27986.11 to 41918.22) | 26945.95 (22447.28 to 32671.16) | 45.05 (37.33 to 53.34) | 20.98 (18.23 to 24.17) | -2.37 (-2.45 to -2.30) | 1211668.15 (1044733.09 to 1382132.92) | 893020.26 (776741.45 to 1039139.63) | 1755.94 (1526.09 to 2000.37) | 941.73 (809.51 to 1095.29) | -1.98 (-2.07 to -1.90) |
| Gabon | 542.12 (276.74 to 868.40) | 629.63 (375.00 to 935.23) | 91.71 (47.99 to 147.16) | 56.08 (33.72 to 81.82) | -1.69 (-1.77 to -1.61) | 19868.68 (9751.17 to 31693.08) | 24957.78 (14967.16 to 36936.60) | 3018.62 (1510.09 to 4813.07) | 1851.04 (1144.06 to 2708.04) | -1.68 (-1.76 to -1.60) |
| Gambia | 104.28 (47.37 to 215.29) | 368.51 (194.92 to 624.23) | 26.82 (12.63 to 54.49) | 34.23 (18.12 to 59.71) | 0.66 (0.32 to 1.00) | 4192.80 (1832.68 to 8791.17) | 14285.50 (7528.45 to 23366.79) | 878.50 (404.33 to 1800.88) | 1081.55 (581.65 to 1784.25) | 0.53 (0.19 to 0.87) |
| Georgia | 1479.86 (1044.08 to 2117.95) | 1735.06 (1170.26 to 2459.43) | 23.97 (16.93 to 34.44) | 31.68 (22.31 to 43.47) | 2.02 (1.65 to 2.40) | 68737.02 (51063.32 to 90046.39) | 60068.49 (45820.42 to 76251.93) | 1136.95 (841.81 to 1481.71) | 1270.14 (1001.87 to 1575.36) | 1.24 (0.91 to 1.57) |
| Germany | 56074.12 (45452.82 to 71403.28) | 47456.14 (39534.55 to 57879.65) | 47.32 (39.18 to 58.72) | 26.46 (22.89 to 31.06) | -1.95 (-2.05 to -1.85) | 1922413.40 (1653115.00 to 2222120.59) | 1455573.99 (1266137.00 to 1673701.06) | 1829.71 (1583.92 to 2082.93) | 1064.26 (927.73 to 1236.55) | -1.94 (-2.03 to -1.84) |
| Ghana | 3264.33 (1149.27 to 6473.94) | 6663.21 (3318.76 to 11324.15) | 48.40 (17.29 to 95.68) | 37.31 (18.14 to 64.47) | -0.72 (-0.93 to -0.51) | 126652.33 (44847.54 to 245883.28) | 256176.19 (131753.05 to 427316.52) | 1519.96 (552.46 to 2954.04) | 1154.70 (584.68 to 1918.66) | -0.77 (-0.97 to -0.57) |
| Greece | 3531.24 (2143.23 to 5448.21) | 2957.72 (1941.59 to 4278.08) | 24.75 (15.07 to 38.17) | 12.27 (8.71 to 16.57) | -2.55 (-2.69 to -2.41) | 111895.54 (84248.98 to 143904.76) | 86094.36 (65162.14 to 109604.29) | 864.77 (657.96 to 1104.77) | 553.95 (436.60 to 688.79) | -1.59 (-1.66 to -1.52) |
| Greenland | 32.04 (23.11 to 41.75) | 29.86 (21.92 to 38.13) | 66.01 (47.61 to 90.16) | 43.42 (31.40 to 56.31) | -1.12 (-1.19 to -1.04) | 1751.33 (1293.72 to 2240.64) | 1364.23 (1048.58 to 1675.66) | 3053.07 (2312.19 to 3883.75) | 2037.28 (1566.72 to 2504.40) | -1.18 (-1.24 to -1.13) |
| Grenada | 21.67 (16.67 to 28.27) | 31.67 (26.16 to 37.76) | 31.24 (24.59 to 40.09) | 27.08 (22.24 to 32.58) | -0.63 (-0.86 to -0.39) | 851.79 (709.23 to 1018.75) | 1280.86 (1074.49 to 1504.15) | 1256.84 (1042.35 to 1495.23) | 1080.18 (910.61 to 1266.97) | -0.49 (-0.62 to -0.37) |
| Guam | 9.23 (4.51 to 18.11) | 21.18 (9.01 to 35.00) | 9.29 (4.93 to 18.36) | 11.10 (4.54 to 18.34) | 1.01 (0.67 to 1.36) | 529.27 (279.66 to 937.57) | 1018.19 (486.77 to 1571.39) | 432.32 (239.74 to 785.79) | 570.04 (270.99 to 882.04) | 1.22 (0.91 to 1.52) |
| Guatemala | 2720.12 (2305.97 to 3277.52) | 4170.94 (3369.48 to 4988.03) | 59.38 (50.62 to 72.15) | 32.83 (26.50 to 39.52) | -2.25 (-2.48 to -2.01) | 153630.04 (130988.21 to 182206.52) | 235569.69 (196614.46 to 276994.22) | 2837.63 (2434.61 to 3360.51) | 1675.24 (1415.45 to 1976.71) | -2.00 (-2.19 to -1.82) |
| Guinea | 652.79 (370.01 to 1176.65) | 1234.65 (684.74 to 2160.35) | 19.34 (11.01 to 35.13) | 20.21 (11.38 to 34.67) | 0.03 (-0.13 to 0.20) | 22859.94 (13197.02 to 40363.01) | 48094.94 (28159.04 to 80788.86) | 613.88 (357.16 to 1089.24) | 655.81 (380.95 to 1112.59) | 0.11 (-0.04 to 0.26) |
| Guinea-Bissau | 339.30 (125.43 to 703.50) | 454.29 (227.79 to 798.00) | 77.52 (28.81 to 160.32) | 51.38 (25.52 to 94.94) | -1.28 (-1.42 to -1.13) | 12868.64 (4431.83 to 26575.66) | 19169.71 (9803.19 to 32866.45) | 2465.61 (910.36 to 5070.65) | 1677.51 (863.73 to 2925.81) | -1.17 (-1.32 to -1.03) |
| Guyana | 309.14 (233.88 to 406.51) | 313.00 (231.79 to 411.61) | 69.09 (50.08 to 91.52) | 44.46 (33.14 to 58.90) | -0.89 (-1.05 to -0.73) | 13631.39 (10297.16 to 17230.61) | 13971.49 (10720.50 to 17707.02) | 2531.14 (1942.28 to 3236.47) | 1853.86 (1428.40 to 2341.04) | -0.52 (-0.66 to -0.39) |
| Haiti | 2142.38 (1427.60 to 3751.65) | 3443.40 (2286.25 to 5146.67) | 58.05 (39.73 to 98.00) | 40.97 (27.10 to 61.05) | -1.02 (-1.11 to -0.94) | 94893.19 (66140.44 to 158211.76) | 161179.59 (115032.07 to 224735.69) | 2184.38 (1547.92 to 3656.55) | 1570.89 (1117.44 to 2212.16) | -0.94 (-1.05 to -0.82) |
| Honduras | 759.20 (574.29 to 1024.51) | 1851.65 (1345.42 to 2552.57) | 30.09 (22.35 to 40.99) | 26.39 (19.37 to 35.48) | -0.28 (-0.45 to -0.11) | 41257.45 (31988.89 to 53863.74) | 91512.41 (70752.40 to 119500.15) | 1376.32 (1074.41 to 1780.94) | 1115.66 (864.08 to 1447.56) | -0.62 (-0.81 to -0.43) |
| Hungary | 11025.07 (9053.28 to 13785.40) | 7168.31 (6000.90 to 8662.72) | 81.22 (66.92 to 100.46) | 41.45 (35.01 to 49.54) | -2.97 (-3.24 to -2.71) | 399145.10 (342422.56 to 460942.77) | 238106.58 (203861.79 to 276163.28) | 3093.72 (2670.83 to 3544.62) | 1591.30 (1364.86 to 1837.50) | -3.02 (-3.29 to -2.74) |
| Iceland | 24.50 (15.98 to 40.01) | 58.79 (44.36 to 77.84) | 8.95 (6.00 to 14.24) | 11.05 (8.66 to 13.98) | 0.86 (0.73 to 1.00) | 1545.39 (1201.60 to 1991.56) | 2738.01 (2242.55 to 3334.47) | 579.54 (453.70 to 741.11) | 631.78 (513.83 to 779.54) | 0.36 (0.26 to 0.45) |
| India | 137039.07 (64655.92 to 296463.50) | 261096.39 (170846.55 to 400404.85) | 24.77 (11.88 to 54.30) | 20.38 (13.34 to 31.76) | -0.49 (-0.61 to -0.36) | 6752740.45 (3743829.72 to 13017462.15) | 11493741.43 (8010155.84 to 16772009.13) | 1048.33 (578.59 to 2058.13) | 827.47 (578.12 to 1215.07) | -0.66 (-0.74 to -0.59) |
| Indonesia | 14551.12 (9157.51 to 27065.15) | 23577.91 (17299.84 to 34328.10) | 13.60 (8.71 to 24.66) | 9.86 (7.25 to 14.13) | -1.11 (-1.18 to -1.03) | 603264.82 (382024.66 to 1155840.36) | 896450.76 (662312.26 to 1322479.76) | 462.00 (300.27 to 836.12) | 320.57 (239.17 to 471.56) | -1.28 (-1.34 to -1.21) |
| Iran (Islamic Republic of) | 470.01 (385.02 to 583.72) | 1693.80 (1380.13 to 2085.45) | 1.76 (1.44 to 2.23) | 2.13 (1.73 to 2.65) | 1.39 (1.04 to 1.75) | 31684.90 (25422.43 to 39192.32) | 95274.67 (78852.76 to 114333.28) | 84.61 (68.57 to 102.27) | 105.66 (87.76 to 125.86) | 1.36 (1.05 to 1.67) |
| Iraq | 218.30 (150.13 to 343.11) | 398.65 (268.23 to 551.32) | 2.52 (1.66 to 4.00) | 1.68 (1.15 to 2.33) | -1.66 (-1.86 to -1.47) | 13491.39 (9932.41 to 19263.59) | 27790.38 (21397.15 to 36511.22) | 116.14 (84.48 to 164.71) | 82.06 (63.91 to 106.89) | -1.31 (-1.39 to -1.23) |
| Ireland | 750.13 (554.37 to 1074.91) | 869.68 (702.79 to 1098.49) | 19.40 (14.34 to 27.74) | 11.77 (9.76 to 14.59) | -1.70 (-1.94 to -1.45) | 35572.36 (28582.94 to 43841.02) | 44376.95 (36692.27 to 53671.40) | 962.94 (777.98 to 1181.28) | 744.26 (605.54 to 918.63) | -0.94 (-1.21 to -0.68) |
| Israel | 268.96 (206.96 to 370.74) | 572.28 (466.19 to 710.29) | 5.65 (4.36 to 7.84) | 4.93 (4.06 to 6.02) | -0.48 (-0.77 to -0.20) | 13446.12 (10766.75 to 16719.56) | 26166.70 (21814.71 to 31533.50) | 284.59 (228.16 to 354.16) | 256.27 (210.97 to 310.40) | -0.35 (-0.59 to -0.11) |
| Italy | 31508.28 (23450.12 to 41245.83) | 22419.27 (16827.02 to 28956.60) | 36.97 (27.65 to 47.92) | 14.54 (11.58 to 17.98) | -3.11 (-3.21 to -3.01) | 969553.10 (802190.74 to 1149059.59) | 598883.18 (498969.13 to 722621.56) | 1268.61 (1063.57 to 1489.53) | 591.06 (500.49 to 703.64) | -2.59 (-2.66 to -2.52) |
| Jamaica | 214.74 (156.02 to 281.81) | 399.94 (275.14 to 551.62) | 11.90 (8.64 to 15.55) | 12.81 (8.83 to 17.64) | 0.42 (0.04 to 0.80) | 9820.26 (7841.79 to 12122.44) | 17251.27 (13045.59 to 22221.98) | 514.16 (409.92 to 627.99) | 553.64 (418.79 to 713.32) | 0.26 (-0.03 to 0.56) |
| Japan | 36387.11 (26569.27 to 48165.99) | 36591.12 (26351.53 to 49387.67) | 22.42 (16.15 to 29.93) | 11.36 (9.04 to 14.12) | -2.48 (-2.59 to -2.37) | 1231211.69 (980007.02 to 1500891.43) | 983684.03 (781297.76 to 1230066.41) | 775.81 (621.47 to 945.88) | 454.36 (369.21 to 543.70) | -1.97 (-2.10 to -1.85) |
| Jordan | 25.31 (18.93 to 34.99) | 72.79 (54.08 to 99.54) | 1.82 (1.32 to 2.56) | 0.98 (0.71 to 1.34) | -2.34 (-2.82 to -1.86) | 2104.32 (1581.07 to 2734.52) | 7232.06 (5413.20 to 9536.67) | 90.64 (69.93 to 115.84) | 64.98 (50.26 to 83.87) | -1.28 (-1.54 to -1.01) |
| Kazakhstan | 6008.64 (4578.10 to 7834.70) | 7308.60 (5989.82 to 9004.11) | 43.46 (32.82 to 57.91) | 39.07 (31.43 to 48.91) | -0.96 (-1.64 to -0.27) | 310305.70 (241885.19 to 382737.40) | 346708.41 (289067.05 to 416893.11) | 2056.68 (1601.13 to 2557.03) | 1749.34 (1460.13 to 2113.59) | -1.17 (-1.90 to -0.42) |
| Kenya | 5648.31 (1185.58 to 12394.49) | 12824.02 (4151.70 to 25049.54) | 64.33 (14.68 to 142.90) | 51.13 (17.29 to 101.32) | -0.51 (-0.76 to -0.26) | 208694.66 (47942.83 to 444617.84) | 486123.84 (163276.65 to 920213.10) | 1911.10 (454.61 to 4074.68) | 1565.50 (546.78 to 3011.86) | -0.41 (-0.71 to -0.11) |
| Kiribati | 10.71 (2.46 to 31.89) | 10.08 (3.66 to 26.30) | 22.61 (5.62 to 67.78) | 11.05 (4.18 to 28.94) | -2.70 (-2.99 to -2.41) | 516.15 (146.44 to 1440.34) | 514.09 (209.33 to 1251.49) | 928.76 (277.22 to 2621.87) | 490.92 (206.68 to 1184.24) | -2.42 (-2.69 to -2.15) |
| Kuwait | 11.60 (9.88 to 13.46) | 18.90 (13.94 to 25.66) | 1.44 (1.19 to 1.75) | 0.63 (0.46 to 0.85) | -2.77 (-3.40 to -2.13) | 1224.55 (934.93 to 1652.12) | 2822.37 (2007.32 to 3818.58) | 84.54 (69.13 to 105.58) | 53.37 (39.73 to 71.56) | -1.64 (-1.98 to -1.30) |
| Kyrgyzstan | 1399.67 (1169.37 to 1690.68) | 2148.94 (1768.93 to 2610.11) | 42.96 (35.97 to 51.96) | 37.34 (30.60 to 45.38) | -0.75 (-1.47 to -0.03) | 72115.19 (60561.53 to 84835.24) | 108423.53 (90299.74 to 130179.96) | 2049.08 (1727.98 to 2415.06) | 1733.21 (1442.61 to 2080.90) | -0.91 (-1.54 to -0.28) |
| Lao People's Democratic Republic | 1334.97 (435.80 to 2794.54) | 2012.53 (1149.18 to 3231.35) | 55.40 (18.14 to 117.28) | 38.98 (21.54 to 63.17) | -1.25 (-1.50 to -1.00) | 56748.08 (18981.29 to 114931.26) | 84843.36 (50206.25 to 130689.79) | 2062.59 (728.82 to 4155.73) | 1381.83 (833.56 to 2147.67) | -1.43 (-1.65 to -1.22) |
| Latvia | 1435.93 (1153.40 to 1941.79) | 1729.58 (1364.50 to 2278.97) | 44.24 (35.93 to 58.74) | 55.68 (46.46 to 67.92) | -0.07 (-0.61 to 0.47) | 65716.49 (54967.07 to 79256.87) | 61729.95 (52073.89 to 73734.95) | 2146.78 (1802.34 to 2556.30) | 2369.85 (2044.75 to 2724.77) | -0.46 (-0.93 to 0.01) |
| Lebanon | 270.37 (183.46 to 368.79) | 206.01 (141.32 to 288.90) | 11.70 (7.72 to 16.18) | 3.36 (2.31 to 4.71) | -3.38 (-3.69 to -3.07) | 12117.33 (8857.75 to 15846.03) | 9125.30 (6901.63 to 11411.98) | 462.67 (337.20 to 606.87) | 152.27 (115.20 to 190.46) | -2.70 (-3.06 to -2.35) |
| Lesotho | 492.84 (76.21 to 1151.50) | 1096.84 (208.74 to 2391.18) | 53.01 (9.14 to 122.38) | 83.86 (18.31 to 180.38) | 2.13 (1.64 to 2.61) | 20512.68 (4074.49 to 45765.04) | 48508.24 (11038.48 to 102484.46) | 2000.04 (418.08 to 4445.26) | 3261.31 (759.13 to 6928.08) | 2.22 (1.73 to 2.71) |
| Liberia | 634.12 (325.97 to 1074.51) | 890.81 (497.42 to 1383.85) | 52.94 (26.83 to 90.33) | 35.09 (20.22 to 54.10) | -1.78 (-2.01 to -1.55) | 22942.26 (12076.20 to 38057.14) | 37350.88 (21567.90 to 57282.61) | 1646.85 (865.17 to 2754.14) | 1137.06 (655.09 to 1722.95) | -1.65 (-1.93 to -1.37) |
| Libya | 33.35 (21.43 to 57.15) | 146.05 (99.81 to 216.02) | 1.71 (1.07 to 3.05) | 2.56 (1.74 to 3.73) | 1.76 (1.32 to 2.20) | 2488.26 (1817.95 to 3400.67) | 8495.50 (6371.13 to 11196.77) | 88.83 (64.84 to 124.93) | 119.77 (90.65 to 160.81) | 1.26 (0.92 to 1.61) |
| Lithuania | 1600.03 (1303.26 to 2053.77) | 1843.38 (1545.28 to 2308.19) | 38.31 (31.21 to 48.43) | 41.60 (35.58 to 49.38) | -0.37 (-1.19 to 0.45) | 84136.11 (71289.35 to 99334.36) | 70378.93 (60241.30 to 82691.38) | 2074.43 (1761.78 to 2443.13) | 1877.57 (1614.33 to 2168.11) | -0.86 (-1.56 to -0.15) |
| Luxembourg | 235.93 (174.56 to 314.47) | 195.79 (157.94 to 242.98) | 46.07 (34.64 to 61.03) | 18.72 (15.38 to 22.81) | -2.73 (-2.81 to -2.64) | 8302.42 (6909.96 to 9938.39) | 7568.16 (6438.15 to 8907.92) | 1741.49 (1460.23 to 2058.09) | 857.92 (723.96 to 1026.51) | -2.26 (-2.31 to -2.21) |
| Madagascar | 2389.33 (720.47 to 5395.54) | 2870.31 (1216.18 to 6005.90) | 42.77 (13.94 to 93.44) | 22.10 (10.09 to 45.81) | -2.23 (-2.55 to -1.91) | 101557.26 (33197.34 to 222892.77) | 140063.86 (68635.87 to 270184.45) | 1487.43 (516.85 to 3212.66) | 803.45 (395.48 to 1552.26) | -2.05 (-2.34 to -1.77) |
| Malawi | 1746.11 (638.70 to 4102.88) | 3517.99 (1899.82 to 6190.04) | 41.02 (16.30 to 96.04) | 39.75 (21.87 to 70.98) | -0.32 (-0.58 to -0.06) | 74372.42 (30236.88 to 161776.01) | 156621.69 (89516.24 to 266104.26) | 1400.22 (589.90 to 3079.93) | 1395.00 (809.17 to 2376.80) | -0.22 (-0.48 to 0.05) |
| Malaysia | 1387.31 (825.34 to 2306.35) | 2287.60 (1649.23 to 3098.38) | 13.14 (7.85 to 22.38) | 7.70 (5.55 to 10.32) | -2.28 (-2.56 to -1.99) | 63040.70 (40392.83 to 99134.24) | 97781.15 (72182.92 to 126065.71) | 497.81 (316.86 to 789.56) | 302.02 (222.99 to 392.05) | -2.11 (-2.41 to -1.81) |
| Maldives | 7.74 (4.76 to 13.46) | 13.11 (8.77 to 19.45) | 8.20 (5.48 to 13.60) | 3.59 (2.41 to 5.28) | -4.16 (-4.94 to -3.37) | 373.28 (248.15 to 618.68) | 955.49 (677.98 to 1309.43) | 305.43 (207.75 to 502.07) | 173.77 (125.05 to 238.76) | -3.19 (-3.83 to -2.55) |
| Mali | 1031.03 (494.19 to 2415.07) | 1738.91 (1097.51 to 2992.45) | 26.45 (12.10 to 63.82) | 19.40 (11.64 to 35.37) | -0.92 (-0.98 to -0.87) | 35546.95 (18208.93 to 78690.07) | 66173.87 (44158.25 to 108601.84) | 766.26 (381.25 to 1734.34) | 581.87 (379.70 to 969.04) | -0.76 (-0.84 to -0.68) |
| Malta | 52.61 (35.77 to 80.93) | 71.53 (51.73 to 98.15) | 12.66 (8.58 to 19.69) | 8.22 (6.29 to 10.79) | -1.20 (-1.38 to -1.02) | 2381.07 (1876.64 to 3033.13) | 2927.97 (2335.00 to 3626.27) | 587.72 (462.02 to 747.45) | 473.51 (379.71 to 589.80) | -0.59 (-0.70 to -0.48) |
| Marshall Islands | 6.53 (2.28 to 15.72) | 10.24 (4.22 to 21.08) | 28.01 (10.09 to 71.19) | 23.43 (9.43 to 51.78) | -0.47 (-0.55 to -0.39) | 336.53 (134.26 to 718.53) | 507.29 (242.15 to 939.17) | 1180.14 (474.87 to 2662.88) | 986.55 (458.71 to 1905.25) | -0.52 (-0.59 to -0.46) |
| Mauritania | 139.64 (78.00 to 225.12) | 173.19 (113.60 to 251.92) | 13.71 (7.65 to 22.44) | 7.85 (5.11 to 11.36) | -2.02 (-2.18 to -1.85) | 4886.06 (3135.29 to 7226.44) | 6622.70 (4663.21 to 9236.89) | 428.39 (266.96 to 642.53) | 253.87 (177.56 to 354.71) | -1.86 (-2.01 to -1.71) |
| Mauritius | 280.32 (212.42 to 356.23) | 273.88 (204.44 to 347.13) | 33.58 (24.60 to 43.48) | 15.49 (11.54 to 19.58) | -3.75 (-4.26 to -3.25) | 12470.87 (10041.17 to 15054.14) | 11779.12 (9500.90 to 14204.44) | 1332.26 (1044.05 to 1630.29) | 711.17 (581.45 to 856.30) | -3.05 (-3.44 to -2.66) |
| Mexico | 22957.23 (19978.48 to 26571.65) | 38852.83 (31559.85 to 46925.49) | 44.53 (38.84 to 51.74) | 29.20 (23.79 to 35.30) | -1.74 (-2.01 to -1.48) | 1123206.26 (978297.81 to 1282826.97) | 1752151.07 (1457893.00 to 2091429.92) | 1850.88 (1624.03 to 2115.12) | 1279.65 (1066.43 to 1525.01) | -1.50 (-1.78 to -1.22) |
| Micronesia (Federated States of) | 18.73 (10.68 to 29.91) | 12.92 (7.18 to 20.82) | 31.48 (17.43 to 52.00) | 14.62 (8.25 to 23.84) | -2.83 (-2.99 to -2.67) | 896.50 (532.31 to 1359.09) | 660.94 (418.77 to 990.56) | 1305.72 (783.94 to 2021.36) | 680.91 (430.76 to 1029.61) | -2.40 (-2.53 to -2.26) |
| Monaco | 14.02 (5.54 to 28.63) | 15.98 (6.67 to 28.12) | 21.48 (9.02 to 40.26) | 18.11 (7.72 to 30.75) | -0.50 (-0.54 to -0.46) | 394.02 (193.92 to 671.32) | 451.09 (223.81 to 718.72) | 829.49 (435.54 to 1299.74) | 748.62 (386.97 to 1147.54) | -0.32 (-0.38 to -0.26) |
| Mongolia | 729.39 (543.20 to 1007.68) | 2058.64 (1612.34 to 2532.12) | 61.31 (46.05 to 83.29) | 75.20 (58.51 to 91.77) | 1.16 (0.79 to 1.53) | 31915.05 (23521.78 to 43515.58) | 96613.78 (77992.99 to 117058.97) | 2298.27 (1708.11 to 3084.72) | 3064.39 (2495.77 to 3695.66) | 1.42 (1.06 to 1.78) |
| Montenegro | 204.59 (128.93 to 293.24) | 294.86 (184.33 to 430.06) | 33.01 (20.40 to 48.02) | 32.69 (20.48 to 48.22) | -0.04 (-0.18 to 0.10) | 7872.65 (6045.74 to 10071.17) | 9207.41 (6788.97 to 12161.51) | 1218.15 (931.07 to 1561.35) | 1077.58 (814.17 to 1411.04) | -0.41 (-0.56 to -0.26) |
| Morocco | 453.64 (277.14 to 890.47) | 517.95 (342.89 to 795.23) | 2.58 (1.61 to 4.77) | 1.48 (0.98 to 2.23) | -1.70 (-1.77 to -1.63) | 30096.62 (20266.63 to 52710.45) | 33769.28 (24811.05 to 45171.10) | 143.57 (99.32 to 252.47) | 88.47 (65.31 to 117.86) | -1.55 (-1.61 to -1.49) |
| Mozambique | 1122.21 (553.83 to 2270.94) | 4646.31 (1468.40 to 10445.20) | 17.91 (9.55 to 34.64) | 35.92 (13.32 to 78.21) | 2.57 (2.40 to 2.73) | 49741.52 (26996.12 to 92912.17) | 208275.63 (77884.65 to 452211.90) | 641.24 (358.07 to 1197.70) | 1247.73 (497.28 to 2667.93) | 2.51 (2.35 to 2.67) |
| Myanmar | 7538.85 (4602.95 to 11873.85) | 18870.70 (12071.00 to 27391.20) | 26.64 (16.53 to 41.92) | 35.15 (22.54 to 51.92) | 1.14 (1.04 to 1.24) | 341374.04 (220002.67 to 524658.39) | 785244.61 (519829.52 to 1098827.30) | 1105.18 (711.32 to 1692.98) | 1387.94 (913.81 to 1958.40) | 0.95 (0.86 to 1.05) |
| Namibia | 390.02 (91.33 to 879.64) | 901.15 (429.19 to 1542.14) | 54.87 (15.59 to 122.84) | 60.18 (29.69 to 101.94) | 0.21 (-0.27 to 0.69) | 17314.27 (5279.02 to 36060.43) | 39089.35 (19628.62 to 66270.25) | 1984.33 (611.24 to 4196.35) | 2099.93 (1092.79 to 3463.06) | 0.06 (-0.40 to 0.53) |
| Nauru | 2.25 (1.02 to 3.94) | 2.52 (1.37 to 4.03) | 37.32 (16.12 to 66.79) | 33.09 (18.28 to 53.31) | -0.45 (-0.64 to -0.27) | 110.49 (52.48 to 184.64) | 127.05 (75.35 to 188.25) | 1504.40 (720.64 to 2542.54) | 1417.05 (841.41 to 2148.50) | -0.25 (-0.43 to -0.07) |
| Nepal | 1372.23 (754.04 to 2386.21) | 4944.41 (2830.19 to 8917.77) | 12.58 (6.76 to 21.40) | 20.16 (11.44 to 36.30) | 1.66 (1.42 to 1.90) | 70283.27 (46016.75 to 109275.84) | 213049.53 (132302.40 to 354076.17) | 542.78 (350.28 to 856.23) | 777.60 (481.25 to 1311.33) | 1.27 (1.05 to 1.49) |
| Netherlands | 3897.68 (2883.10 to 5477.01) | 5274.75 (4108.48 to 6836.78) | 20.10 (15.14 to 27.85) | 15.60 (12.43 to 19.52) | -0.98 (-1.15 to -0.80) | 144634.20 (118540.66 to 175894.69) | 169674.94 (142128.26 to 199815.97) | 801.13 (661.82 to 963.23) | 653.08 (547.29 to 778.31) | -0.88 (-1.03 to -0.72) |
| New Zealand | 923.12 (730.72 to 1197.63) | 1093.70 (852.94 to 1414.00) | 24.31 (19.28 to 31.65) | 13.88 (10.96 to 17.50) | -1.72 (-1.87 to -1.57) | 42971.25 (35645.68 to 51390.42) | 55226.94 (44943.56 to 67210.16) | 1160.75 (961.29 to 1384.54) | 869.92 (702.15 to 1064.99) | -0.78 (-1.08 to -0.48) |
| Nicaragua | 498.28 (387.31 to 656.43) | 1150.44 (917.21 to 1423.85) | 25.91 (20.40 to 34.08) | 20.58 (16.24 to 25.78) | -0.61 (-0.79 to -0.44) | 28910.15 (22783.88 to 35976.10) | 65595.51 (53960.65 to 78853.24) | 1220.84 (966.95 to 1526.73) | 1058.21 (867.59 to 1266.40) | -0.40 (-0.55 to -0.24) |
| Niger | 445.15 (297.28 to 665.74) | 982.74 (590.90 to 1617.12) | 14.87 (9.91 to 22.18) | 11.03 (6.81 to 18.03) | -0.80 (-0.96 to -0.64) | 17307.08 (12305.07 to 24625.09) | 40705.97 (26363.23 to 64048.02) | 474.22 (328.80 to 677.03) | 364.25 (234.31 to 576.17) | -0.69 (-0.86 to -0.52) |
| Nigeria | 21117.26 (10201.19 to 39769.45) | 30275.27 (17645.52 to 51355.04) | 46.68 (22.84 to 89.44) | 31.75 (18.52 to 53.95) | -1.47 (-1.69 to -1.25) | 743176.99 (375653.42 to 1397991.23) | 1168393.18 (716787.11 to 1950344.19) | 1406.89 (711.00 to 2636.01) | 947.83 (584.44 to 1566.09) | -1.50 (-1.70 to -1.29) |
| Niue | 0.38 (0.12 to 0.63) | 0.38 (0.17 to 0.58) | 17.96 (5.55 to 30.01) | 18.64 (8.34 to 27.89) | -0.68 (-1.06 to -0.30) | 16.18 (5.97 to 25.65) | 15.93 (8.43 to 22.73) | 791.16 (293.25 to 1254.35) | 821.80 (442.61 to 1161.82) | -0.62 (-0.95 to -0.29) |
| North Macedonia | 846.03 (537.13 to 1270.00) | 1011.99 (595.51 to 1539.20) | 48.18 (28.75 to 75.75) | 34.51 (18.81 to 54.55) | -1.55 (-1.81 to -1.28) | 28816.82 (21738.41 to 37624.08) | 31111.38 (21542.35 to 41913.78) | 1486.58 (1092.26 to 1973.00) | 1021.58 (716.53 to 1376.62) | -1.58 (-1.75 to -1.40) |
| Northern Mariana Islands | 5.39 (2.24 to 10.06) | 9.22 (4.38 to 14.79) | 18.49 (7.88 to 34.46) | 16.92 (7.75 to 27.16) | -0.72 (-0.95 to -0.50) | 319.02 (162.68 to 546.77) | 420.22 (216.99 to 635.22) | 802.69 (408.20 to 1379.78) | 747.12 (385.40 to 1124.35) | -0.59 (-0.77 to -0.40) |
| Norway | 881.13 (611.80 to 1385.61) | 1006.19 (773.25 to 1299.95) | 15.22 (11.21 to 22.33) | 10.79 (8.62 to 13.52) | -0.99 (-1.19 to -0.79) | 41842.51 (33477.29 to 52536.48) | 39549.86 (32435.43 to 47611.86) | 842.20 (687.37 to 1025.93) | 538.19 (440.02 to 646.93) | -1.30 (-1.43 to -1.17) |
| Oman | 24.09 (15.93 to 34.66) | 45.75 (33.05 to 62.75) | 2.74 (1.79 to 3.99) | 1.81 (1.31 to 2.46) | -1.02 (-1.55 to -0.48) | 1821.51 (1342.42 to 2443.97) | 4293.45 (3209.99 to 5621.90) | 133.31 (98.27 to 177.23) | 97.04 (75.32 to 125.31) | -0.91 (-1.34 to -0.47) |
| Pakistan | 4116.76 (1608.70 to 10644.39) | 9637.82 (4796.49 to 19869.46) | 6.58 (2.65 to 16.93) | 6.43 (3.43 to 12.89) | -0.19 (-0.39 to 0.02) | 286160.05 (167858.02 to 526682.90) | 746842.69 (483453.94 to 1234332.97) | 385.03 (223.01 to 727.59) | 395.77 (258.25 to 659.98) | -0.01 (-0.15 to 0.13) |
| Palau | 1.85 (0.67 to 3.67) | 3.89 (1.59 to 7.30) | 15.63 (5.75 to 31.89) | 17.25 (7.00 to 32.94) | 0.30 (0.21 to 0.39) | 92.92 (39.54 to 168.72) | 179.22 (79.65 to 313.44) | 681.39 (287.87 to 1271.13) | 798.56 (366.57 to 1371.59) | 0.50 (0.41 to 0.58) |
| Palestine | 49.28 (31.00 to 76.00) | 79.44 (60.19 to 103.18) | 5.66 (3.57 to 8.72) | 3.06 (2.27 to 4.12) | -2.16 (-2.45 to -1.87) | 2177.67 (1609.63 to 3062.78) | 4632.88 (3639.44 to 5820.75) | 190.85 (135.96 to 274.78) | 124.53 (98.61 to 154.45) | -1.50 (-1.68 to -1.32) |
| Panama | 320.20 (238.37 to 422.63) | 620.80 (447.05 to 804.23) | 19.03 (14.08 to 25.45) | 14.02 (10.10 to 18.11) | -0.95 (-1.15 to -0.75) | 17124.47 (13152.63 to 21796.91) | 31447.54 (24853.67 to 38182.38) | 862.10 (661.58 to 1089.30) | 713.22 (563.71 to 865.66) | -0.56 (-0.74 to -0.39) |
| Papua New Guinea | 341.13 (115.57 to 799.77) | 584.81 (257.10 to 1267.72) | 15.08 (5.21 to 37.80) | 9.03 (3.89 to 20.74) | -1.37 (-1.76 to -0.97) | 18008.94 (7642.55 to 37601.32) | 34582.96 (19191.68 to 65191.03) | 629.42 (271.62 to 1350.40) | 418.57 (229.16 to 814.56) | -1.10 (-1.42 to -0.78) |
| Paraguay | 685.45 (488.61 to 921.56) | 1721.63 (1256.55 to 2354.01) | 28.15 (19.64 to 38.59) | 27.66 (20.10 to 38.09) | -0.10 (-0.24 to 0.05) | 35486.41 (27021.84 to 44842.47) | 87053.18 (67870.63 to 110781.23) | 1216.76 (923.61 to 1529.57) | 1273.24 (993.38 to 1623.90) | 0.06 (-0.06 to 0.17) |
| Peru | 4548.60 (2141.28 to 7833.29) | 6475.70 (4587.98 to 8876.19) | 32.82 (16.85 to 54.82) | 18.75 (13.38 to 25.68) | -2.11 (-2.54 to -1.68) | 212907.42 (96861.45 to 365200.48) | 267807.69 (191090.62 to 355091.07) | 1315.92 (657.69 to 2189.78) | 739.23 (532.40 to 976.68) | -2.10 (-2.47 to -1.73) |
| Philippines | 12284.06 (5251.60 to 21491.57) | 27477.57 (15567.18 to 44006.21) | 34.26 (15.07 to 60.87) | 30.53 (17.31 to 49.55) | -0.19 (-0.30 to -0.09) | 587927.70 (270535.24 to 997284.12) | 1155732.06 (651416.15 to 1805621.59) | 1326.39 (607.90 to 2254.80) | 1143.56 (650.55 to 1779.55) | -0.39 (-0.46 to -0.31) |
| Poland | 14221.56 (10627.34 to 19891.88) | 22858.58 (19304.50 to 27460.69) | 33.88 (25.24 to 47.60) | 37.41 (32.05 to 43.85) | 0.29 (0.12 to 0.46) | 635015.43 (524427.30 to 771526.41) | 911435.59 (790034.95 to 1033123.25) | 1531.63 (1265.98 to 1853.30) | 1700.18 (1479.58 to 1935.69) | 0.34 (0.20 to 0.49) |
| Portugal | 7045.11 (4692.62 to 9987.79) | 4812.99 (3717.71 to 6181.33) | 54.72 (36.07 to 78.28) | 20.87 (17.08 to 25.27) | -3.35 (-3.45 to -3.25) | 235632.07 (187223.30 to 289363.79) | 157289.34 (133291.61 to 184067.43) | 1945.20 (1570.22 to 2358.44) | 961.14 (813.01 to 1144.45) | -2.43 (-2.51 to -2.36) |
| Puerto Rico | 1160.33 (1031.53 to 1316.85) | 930.42 (755.10 to 1126.73) | 32.33 (28.80 to 36.73) | 16.80 (13.63 to 20.25) | -2.33 (-2.58 to -2.08) | 52544.45 (46182.51 to 59894.32) | 36845.96 (30700.39 to 43942.26) | 1456.76 (1280.46 to 1658.02) | 835.37 (694.70 to 1013.28) | -2.01 (-2.22 to -1.80) |
| Qatar | 9.78 (6.68 to 14.52) | 39.21 (27.68 to 55.62) | 6.97 (4.92 to 9.79) | 3.71 (2.60 to 5.12) | -2.30 (-2.98 to -1.63) | 710.84 (523.94 to 943.90) | 3657.27 (2786.45 to 4845.70) | 236.31 (179.15 to 316.77) | 143.24 (110.02 to 185.71) | -1.69 (-2.16 to -1.22) |
| Republic of Korea | 22042.68 (17353.03 to 27664.34) | 16324.75 (13512.16 to 20399.83) | 68.70 (51.81 to 89.94) | 18.69 (15.38 to 23.11) | -4.51 (-4.67 to -4.36) | 985943.63 (808166.05 to 1188908.08) | 675794.02 (570446.00 to 799050.27) | 2516.48 (2029.37 to 3067.01) | 880.53 (734.98 to 1047.35) | -3.48 (-3.57 to -3.39) |
| Republic of Moldova | 3583.59 (2987.47 to 4317.21) | 3250.91 (2727.74 to 3923.09) | 80.07 (65.83 to 97.62) | 58.37 (49.21 to 69.89) | -1.34 (-1.66 to -1.02) | 143726.90 (122666.83 to 169346.80) | 118927.40 (101329.39 to 139560.61) | 3133.61 (2670.36 to 3698.85) | 2295.96 (1954.33 to 2676.38) | -1.29 (-1.61 to -0.96) |
| Romania | 14413.47 (10834.35 to 19146.81) | 16648.42 (12396.22 to 22306.86) | 54.72 (40.04 to 73.98) | 48.83 (38.09 to 63.00) | -0.94 (-1.25 to -0.63) | 558577.24 (456853.87 to 673253.07) | 510204.29 (419382.04 to 627508.96) | 2114.34 (1730.74 to 2554.52) | 1748.50 (1474.59 to 2102.72) | -1.20 (-1.50 to -0.91) |
| Russian Federation | 88487.86 (71535.07 to 113960.88) | 117582.75 (101319.06 to 140568.80) | 51.41 (41.58 to 66.55) | 56.92 (49.55 to 66.99) | -0.80 (-1.95 to 0.36) | 4287891.28 (3635204.97 to 5046473.94) | 4986953.45 (4392101.72 to 5628647.08) | 2522.77 (2145.51 to 2958.04) | 2618.58 (2322.37 to 2940.80) | -0.94 (-2.01 to 0.14) |
| Rwanda | 4251.48 (1336.95 to 8120.77) | 3380.34 (1840.36 to 5613.80) | 132.35 (45.18 to 253.43) | 47.61 (26.93 to 78.48) | -4.50 (-4.97 to -4.03) | 180516.83 (57129.40 to 335793.48) | 160141.13 (95191.19 to 248337.60) | 4526.98 (1524.38 to 8395.53) | 1751.21 (1055.47 to 2727.94) | -4.18 (-4.61 to -3.74) |
| Saint Kitts and Nevis | 16.07 (10.31 to 20.20) | 23.38 (16.90 to 33.43) | 46.86 (31.07 to 58.34) | 31.50 (22.39 to 45.98) | -1.08 (-1.55 to -0.61) | 665.04 (456.36 to 789.24) | 1012.85 (751.59 to 1371.21) | 1994.69 (1412.67 to 2347.20) | 1332.01 (976.68 to 1820.17) | -1.19 (-1.60 to -0.77) |
| Saint Lucia | 44.67 (35.97 to 54.70) | 79.68 (62.47 to 98.75) | 49.85 (39.38 to 62.36) | 34.20 (26.71 to 42.46) | -1.27 (-1.54 to -0.99) | 1889.35 (1580.31 to 2224.18) | 3170.70 (2559.47 to 3793.07) | 1891.20 (1584.90 to 2232.35) | 1410.29 (1141.96 to 1696.47) | -0.88 (-1.04 to -0.72) |
| Saint Vincent and the Grenadines | 20.95 (16.46 to 26.46) | 53.80 (44.40 to 65.89) | 27.92 (21.86 to 35.56) | 39.13 (32.30 to 48.18) | 1.13 (0.76 to 1.51) | 981.16 (804.61 to 1213.33) | 2126.51 (1804.40 to 2553.08) | 1201.41 (995.88 to 1469.77) | 1584.84 (1344.64 to 1899.55) | 0.97 (0.72 to 1.22) |
| Samoa | 17.04 (10.63 to 25.84) | 16.65 (10.30 to 25.19) | 17.25 (10.46 to 26.40) | 10.36 (6.37 to 15.86) | -1.90 (-2.14 to -1.66) | 817.72 (547.68 to 1161.63) | 860.75 (581.65 to 1217.82) | 714.35 (475.27 to 1030.71) | 490.16 (329.36 to 698.21) | -1.40 (-1.58 to -1.22) |
| San Marino | 8.19 (2.73 to 12.89) | 8.75 (2.95 to 14.87) | 23.86 (8.04 to 36.82) | 11.59 (4.18 to 19.17) | -1.48 (-1.79 to -1.18) | 267.33 (112.19 to 372.10) | 275.05 (121.10 to 415.45) | 886.68 (382.14 to 1219.17) | 554.44 (263.16 to 804.70) | -1.05 (-1.22 to -0.89) |
| Sao Tome and Principe | 28.25 (16.55 to 43.81) | 48.66 (30.18 to 67.65) | 43.06 (25.07 to 66.99) | 40.55 (24.92 to 56.91) | -0.61 (-0.99 to -0.22) | 961.66 (583.60 to 1446.16) | 1842.46 (1188.90 to 2531.91) | 1360.39 (824.81 to 2071.40) | 1258.94 (814.81 to 1727.99) | -0.71 (-1.09 to -0.32) |
| Saudi Arabia | 283.81 (153.76 to 504.00) | 444.80 (265.83 to 673.26) | 4.36 (2.45 to 7.75) | 2.15 (1.42 to 3.07) | -1.71 (-1.95 to -1.48) | 16714.98 (10520.31 to 25234.38) | 37193.58 (25793.59 to 51486.34) | 168.79 (108.86 to 271.64) | 100.42 (73.38 to 133.66) | -1.15 (-1.37 to -0.94) |
| Senegal | 532.50 (300.00 to 1015.15) | 775.23 (509.11 to 1204.09) | 15.51 (8.93 to 28.89) | 9.30 (5.92 to 14.29) | -1.64 (-1.90 to -1.39) | 20703.60 (11742.57 to 39159.24) | 31847.54 (21799.65 to 45717.56) | 514.35 (292.50 to 960.59) | 319.64 (217.16 to 463.70) | -1.49 (-1.72 to -1.26) |
| Serbia | 4477.83 (2972.26 to 6558.88) | 4853.34 (3295.53 to 6990.69) | 43.93 (27.32 to 67.42) | 30.81 (21.72 to 43.23) | -1.29 (-1.44 to -1.15) | 166553.42 (125857.93 to 211226.05) | 150531.46 (115860.75 to 191493.49) | 1518.22 (1130.45 to 1958.35) | 1107.38 (885.43 to 1367.89) | -1.14 (-1.28 to -1.01) |
| Seychelles | 17.17 (13.29 to 21.81) | 25.10 (19.78 to 30.99) | 30.07 (23.28 to 38.10) | 20.54 (16.07 to 25.34) | -1.49 (-1.85 to -1.13) | 667.75 (538.63 to 820.20) | 992.62 (806.74 to 1192.04) | 1130.11 (905.05 to 1389.09) | 788.05 (641.84 to 944.66) | -1.42 (-1.76 to -1.08) |
| Sierra Leone | 1039.67 (473.95 to 1986.67) | 1257.63 (702.18 to 2207.43) | 49.23 (22.19 to 94.59) | 29.67 (16.39 to 52.27) | -1.60 (-1.79 to -1.41) | 36262.89 (16847.35 to 68468.78) | 50494.97 (29308.13 to 89325.79) | 1504.55 (699.59 to 2832.52) | 968.52 (562.17 to 1702.55) | -1.35 (-1.52 to -1.17) |
| Singapore | 146.08 (88.45 to 216.61) | 143.89 (93.42 to 201.58) | 5.68 (3.26 to 8.75) | 1.77 (1.15 to 2.46) | -4.23 (-4.51 to -3.96) | 8621.26 (6267.06 to 11193.30) | 10442.38 (7962.73 to 13562.41) | 278.65 (196.69 to 370.79) | 142.38 (106.47 to 187.28) | -2.65 (-2.92 to -2.38) |
| Slovakia | 3071.36 (2486.08 to 4014.51) | 2773.24 (2176.34 to 3577.63) | 52.93 (43.12 to 69.11) | 31.64 (25.19 to 40.33) | -1.54 (-1.62 to -1.46) | 122504.58 (102945.58 to 146237.73) | 106726.55 (86294.01 to 127785.55) | 2149.26 (1809.29 to 2550.19) | 1364.27 (1121.48 to 1621.57) | -1.31 (-1.39 to -1.24) |
| Slovenia | 1261.92 (970.48 to 1641.23) | 985.22 (742.49 to 1292.92) | 52.37 (40.43 to 67.88) | 24.09 (18.65 to 30.66) | -2.90 (-3.11 to -2.69) | 48505.15 (39978.04 to 57781.92) | 33750.93 (26938.41 to 42246.53) | 2077.14 (1717.76 to 2462.98) | 1019.06 (812.97 to 1263.31) | -2.63 (-2.79 to -2.47) |
| Solomon Islands | 15.37 (8.22 to 24.61) | 46.68 (29.75 to 68.26) | 8.67 (4.89 to 13.76) | 10.00 (6.41 to 14.78) | 0.92 (0.59 to 1.26) | 908.17 (567.46 to 1316.47) | 2752.34 (1908.20 to 3738.06) | 422.02 (266.26 to 616.61) | 498.40 (342.91 to 682.27) | 0.91 (0.61 to 1.21) |
| Somalia | 488.16 (306.21 to 710.21) | 1022.57 (651.78 to 1501.30) | 18.33 (11.77 to 26.63) | 14.78 (9.42 to 21.78) | -0.76 (-0.83 to -0.69) | 23676.85 (16661.30 to 32494.82) | 53431.63 (38610.81 to 73294.49) | 650.88 (446.15 to 909.03) | 544.05 (381.58 to 747.17) | -0.64 (-0.70 to -0.59) |
| South Africa | 13877.14 (6282.22 to 23706.97) | 21000.35 (12017.16 to 33115.99) | 53.46 (25.54 to 90.33) | 41.32 (24.24 to 64.87) | -1.12 (-1.63 to -0.60) | 713427.13 (341813.16 to 1157747.04) | 972414.52 (565912.87 to 1477686.96) | 2384.84 (1149.88 to 3872.77) | 1712.56 (1010.52 to 2588.45) | -1.31 (-1.77 to -0.85) |
| South Sudan | 429.17 (255.99 to 722.75) | 588.57 (381.36 to 935.89) | 15.97 (9.80 to 25.90) | 13.66 (9.04 to 21.03) | -0.65 (-0.82 to -0.47) | 19659.33 (13142.35 to 30247.32) | 28257.70 (19658.71 to 40827.35) | 608.86 (411.48 to 934.27) | 512.63 (367.57 to 747.69) | -0.66 (-0.81 to -0.51) |
| Spain | 17615.37 (13802.02 to 22547.82) | 13082.29 (10772.01 to 16018.15) | 34.62 (27.35 to 43.94) | 13.96 (11.90 to 16.44) | -3.07 (-3.21 to -2.92) | 606995.06 (510843.35 to 711985.60) | 434302.88 (373961.91 to 510470.71) | 1301.85 (1100.25 to 1511.58) | 628.86 (531.67 to 740.37) | -2.51 (-2.63 to -2.38) |
| Sri Lanka | 3374.07 (2504.89 to 4366.04) | 3541.86 (2121.81 to 5176.61) | 27.25 (20.04 to 35.94) | 13.61 (8.19 to 19.87) | -2.79 (-3.21 to -2.36) | 151562.92 (117854.25 to 187301.71) | 143729.41 (99078.45 to 195733.96) | 1050.33 (812.70 to 1310.62) | 562.05 (388.60 to 755.48) | -2.79 (-3.24 to -2.33) |
| Sudan | 1276.86 (813.57 to 2081.62) | 314.62 (195.33 to 502.34) | 12.87 (8.06 to 21.82) | 1.59 (1.00 to 2.67) | -7.77 (-8.57 to -6.96) | 52850.56 (34700.26 to 80935.46) | 24370.66 (17863.56 to 32966.68) | 435.48 (285.83 to 676.33) | 78.49 (58.53 to 107.71) | -6.41 (-7.05 to -5.76) |
| Suriname | 88.78 (70.14 to 110.31) | 144.15 (105.17 to 189.80) | 30.87 (24.63 to 38.85) | 22.51 (16.49 to 29.48) | -0.95 (-1.10 to -0.79) | 4236.69 (3349.68 to 5083.78) | 6418.51 (4977.93 to 8018.53) | 1300.24 (1034.26 to 1561.93) | 1007.78 (787.61 to 1250.73) | -0.75 (-0.89 to -0.61) |
| Sweden | 2279.40 (1624.33 to 3544.10) | 2648.91 (2037.98 to 3553.06) | 18.03 (13.93 to 25.90) | 13.24 (10.60 to 16.88) | -0.64 (-0.75 to -0.53) | 101965.58 (82469.19 to 126841.31) | 96053.12 (78614.93 to 116663.01) | 967.76 (795.46 to 1170.32) | 661.13 (538.90 to 802.09) | -0.98 (-1.09 to -0.87) |
| Switzerland | 2855.18 (2260.64 to 3748.37) | 2370.97 (1877.41 to 2994.59) | 29.18 (23.65 to 37.33) | 13.40 (11.12 to 16.07) | -2.39 (-2.50 to -2.27) | 111743.28 (93447.63 to 131444.05) | 89359.87 (74697.20 to 106553.52) | 1295.83 (1089.18 to 1524.75) | 689.02 (571.30 to 834.53) | -2.10 (-2.17 to -2.02) |
| Syrian Arab Republic | 310.32 (209.06 to 442.44) | 311.92 (194.52 to 479.99) | 5.52 (3.66 to 8.10) | 2.50 (1.58 to 3.88) | -3.00 (-3.27 to -2.73) | 15251.66 (11328.65 to 20169.04) | 15154.38 (10945.76 to 20638.89) | 206.71 (153.53 to 278.49) | 108.46 (79.04 to 146.13) | -2.42 (-2.60 to -2.24) |
| Taiwan (Province of China) | 6373.74 (5090.58 to 7903.70) | 6213.74 (5361.04 to 7192.81) | 37.97 (29.95 to 48.08) | 16.09 (13.93 to 18.55) | -3.06 (-3.25 to -2.86) | 245779.49 (201319.10 to 296055.96) | 226652.77 (197675.16 to 257318.99) | 1307.98 (1068.37 to 1586.94) | 646.16 (567.44 to 731.65) | -2.56 (-2.72 to -2.39) |
| Tajikistan | 744.69 (596.35 to 957.91) | 1117.10 (861.08 to 1480.60) | 23.60 (19.05 to 30.27) | 15.42 (12.06 to 19.90) | -1.94 (-2.17 to -1.70) | 42748.10 (33839.64 to 54536.14) | 67390.05 (53312.83 to 86312.11) | 1172.18 (937.14 to 1485.61) | 778.75 (622.17 to 985.66) | -1.95 (-2.16 to -1.74) |
| Thailand | 13777.18 (9186.96 to 20576.08) | 25334.92 (18525.17 to 33765.86) | 33.47 (21.84 to 52.23) | 25.88 (19.06 to 34.23) | -1.22 (-1.39 to -1.05) | 628108.29 (446762.58 to 866088.46) | 981253.54 (752624.93 to 1283140.98) | 1283.98 (894.39 to 1818.91) | 1108.58 (854.64 to 1419.16) | -0.96 (-1.16 to -0.75) |
| Timor-Leste | 78.85 (17.99 to 212.99) | 212.92 (78.82 to 441.44) | 22.28 (5.26 to 60.75) | 24.07 (8.97 to 50.39) | -0.02 (-0.29 to 0.25) | 3777.62 (1202.36 to 9373.81) | 8151.68 (3712.79 to 15815.79) | 805.76 (251.71 to 2048.99) | 818.49 (362.71 to 1587.32) | -0.22 (-0.51 to 0.08) |
| Togo | 478.03 (182.54 to 990.21) | 1145.89 (559.36 to 2077.99) | 34.05 (12.94 to 71.07) | 25.58 (12.51 to 46.31) | -0.66 (-0.78 to -0.54) | 19903.23 (7801.31 to 40445.57) | 47983.34 (25872.29 to 85435.99) | 1116.10 (448.37 to 2234.43) | 870.13 (456.76 to 1525.83) | -0.52 (-0.64 to -0.40) |
| Tokelau | 0.18 (0.09 to 0.32) | 0.18 (0.10 to 0.28) | 13.28 (6.87 to 23.85) | 12.41 (7.22 to 19.56) | -0.39 (-0.49 to -0.29) | 7.58 (4.26 to 12.35) | 8.05 (5.17 to 11.66) | 565.86 (317.59 to 922.87) | 579.51 (374.08 to 839.44) | -0.11 (-0.20 to -0.01) |
| Tonga | 6.47 (4.37 to 9.20) | 7.11 (4.84 to 10.11) | 10.66 (7.13 to 15.24) | 8.52 (5.83 to 12.03) | -0.96 (-1.49 to -0.43) | 294.86 (212.09 to 399.95) | 319.13 (238.00 to 438.19) | 435.00 (313.54 to 592.85) | 363.20 (270.02 to 500.39) | -0.79 (-1.27 to -0.31) |
| Trinidad and Tobago | 226.72 (177.95 to 283.44) | 372.29 (265.53 to 507.27) | 25.42 (19.45 to 32.20) | 20.76 (14.82 to 28.09) | -0.28 (-0.50 to -0.05) | 10520.56 (8510.69 to 12753.62) | 16505.76 (12952.76 to 20766.36) | 1043.48 (854.41 to 1273.12) | 987.80 (778.49 to 1234.62) | 0.29 (0.06 to 0.52) |
| Tunisia | 233.56 (175.91 to 327.50) | 522.55 (358.11 to 742.50) | 4.33 (3.18 to 6.13) | 4.03 (2.77 to 5.72) | -0.41 (-0.48 to -0.34) | 12212.86 (9528.61 to 16018.28) | 23384.33 (17435.98 to 30500.17) | 185.30 (144.55 to 243.04) | 178.15 (133.18 to 231.35) | -0.27 (-0.33 to -0.21) |
| Türkiye | 2604.85 (1731.04 to 3817.82) | 3527.40 (2519.98 to 4757.36) | 6.75 (4.41 to 9.90) | 3.81 (2.71 to 5.19) | -2.23 (-2.58 to -1.87) | 129625.84 (92232.64 to 178600.96) | 158679.64 (123247.50 to 197855.71) | 280.39 (196.67 to 389.17) | 169.13 (131.74 to 211.07) | -1.96 (-2.22 to -1.71) |
| Turkmenistan | 603.06 (470.82 to 771.86) | 2064.07 (1486.96 to 2730.48) | 27.24 (21.63 to 34.54) | 44.46 (32.10 to 58.86) | 1.64 (1.22 to 2.07) | 33053.94 (26266.21 to 41552.14) | 98326.84 (74377.59 to 124369.83) | 1264.45 (1006.58 to 1574.15) | 1956.34 (1476.61 to 2492.32) | 1.40 (1.00 to 1.80) |
| Tuvalu | 1.59 (0.55 to 3.92) | 1.62 (0.80 to 3.25) | 21.00 (7.39 to 52.51) | 14.50 (7.07 to 29.64) | -1.27 (-1.68 to -0.87) | 70.68 (29.43 to 157.44) | 77.83 (44.10 to 136.72) | 862.72 (362.25 to 1938.27) | 661.73 (376.53 to 1170.35) | -0.96 (-1.30 to -0.61) |
| Uganda | 4331.03 (1474.05 to 8666.50) | 8751.88 (4149.32 to 14777.60) | 60.57 (21.80 to 121.19) | 49.34 (24.83 to 82.70) | -1.26 (-1.61 to -0.91) | 169166.95 (56982.07 to 340688.60) | 378308.22 (179498.42 to 627788.11) | 1967.05 (704.70 to 3848.92) | 1656.40 (806.70 to 2726.27) | -1.21 (-1.58 to -0.84) |
| Ukraine | 33780.54 (28544.24 to 43043.16) | 30870.79 (21521.18 to 41549.08) | 52.47 (44.54 to 66.14) | 48.98 (33.77 to 65.35) | -0.79 (-1.54 to -0.04) | 1469015.88 (1265659.68 to 1717467.00) | 1332790.56 (979113.54 to 1734840.06) | 2391.57 (2067.61 to 2769.37) | 2318.62 (1724.17 to 2977.74) | -0.76 (-1.46 to -0.06) |
| United Arab Emirates | 75.88 (50.85 to 108.44) | 247.13 (176.68 to 345.47) | 11.54 (7.34 to 17.65) | 6.21 (4.15 to 9.07) | -1.21 (-1.48 to -0.94) | 5102.05 (3770.96 to 6800.52) | 20886.19 (15663.56 to 26744.11) | 428.23 (299.19 to 604.67) | 249.72 (185.63 to 331.05) | -1.40 (-1.49 to -1.30) |
| United Kingdom | 12821.37 (8388.81 to 20779.60) | 19874.30 (16114.43 to 24710.86) | 15.65 (10.77 to 24.19) | 17.16 (14.47 to 20.40) | 0.38 (0.04 to 0.71) | 548545.01 (428509.31 to 715346.96) | 801211.39 (689202.61 to 936436.15) | 797.89 (634.68 to 993.43) | 897.37 (760.08 to 1063.06) | 0.41 (0.14 to 0.68) |
| United Republic of Tanzania | 6756.76 (2605.69 to 14040.63) | 10785.02 (6195.64 to 18020.86) | 59.37 (23.87 to 123.40) | 39.94 (23.03 to 65.46) | -1.42 (-1.51 to -1.32) | 260026.48 (101443.67 to 534359.77) | 434535.81 (259998.64 to 699249.40) | 1887.78 (785.98 to 3814.67) | 1273.36 (772.57 to 2032.49) | -1.41 (-1.51 to -1.31) |
| United States of America | 61486.36 (54579.42 to 72735.10) | 111663.18 (96406.12 to 129044.29) | 20.82 (18.50 to 24.37) | 22.33 (19.62 to 25.31) | 0.43 (0.28 to 0.58) | 3152914.35 (2738808.51 to 3625301.35) | 4578232.26 (3990148.18 to 5156740.40) | 1125.78 (975.99 to 1298.02) | 1077.67 (942.50 to 1223.29) | 0.09 (-0.07 to 0.24) |
| United States Virgin Islands | 27.37 (15.62 to 41.73) | 31.60 (16.05 to 48.92) | 29.34 (16.30 to 45.85) | 25.00 (11.49 to 38.52) | -0.03 (-0.19 to 0.14) | 1365.38 (794.64 to 1964.33) | 1393.69 (749.20 to 2023.13) | 1333.50 (770.52 to 1938.38) | 1352.03 (678.87 to 1945.44) | 0.61 (0.45 to 0.78) |
| Uruguay | 1164.43 (862.04 to 1554.65) | 1103.63 (852.73 to 1386.17) | 31.20 (23.37 to 41.31) | 21.67 (17.45 to 26.69) | -1.41 (-1.54 to -1.28) | 40759.63 (33056.39 to 49154.74) | 39294.81 (33035.39 to 46388.11) | 1167.90 (958.56 to 1390.94) | 924.40 (779.47 to 1086.12) | -0.86 (-0.98 to -0.74) |
| Uzbekistan | 3091.71 (2377.73 to 3950.58) | 6045.25 (4732.03 to 7608.07) | 23.79 (18.17 to 30.19) | 20.03 (15.68 to 25.44) | -1.15 (-1.56 to -0.75) | 177507.00 (141198.37 to 218265.55) | 304471.37 (244516.23 to 369934.83) | 1183.94 (946.45 to 1455.62) | 908.11 (734.91 to 1102.86) | -1.56 (-1.97 to -1.16) |
| Vanuatu | 14.70 (7.45 to 23.83) | 36.18 (21.13 to 56.14) | 17.36 (8.55 to 29.18) | 16.22 (9.34 to 25.54) | -0.10 (-0.89 to 0.69) | 771.84 (438.06 to 1195.86) | 1887.02 (1213.18 to 2708.06) | 751.16 (415.66 to 1179.73) | 733.12 (457.75 to 1072.32) | 0.00 (-0.68 to 0.68) |
| Venezuela (Bolivarian Republic of) | 3514.64 (2865.75 to 4212.37) | 5884.48 (4182.03 to 7786.35) | 30.30 (24.68 to 36.72) | 20.70 (14.75 to 27.59) | -1.21 (-1.39 to -1.02) | 186250.66 (154464.99 to 218360.81) | 293566.90 (220801.92 to 378944.77) | 1301.66 (1098.10 to 1525.92) | 1065.10 (796.45 to 1383.12) | -0.52 (-0.70 to -0.33) |
| Viet Nam | 5461.59 (3571.08 to 7782.16) | 46156.24 (30557.36 to 64285.72) | 13.22 (8.77 to 18.99) | 46.58 (30.27 to 65.25) | 4.86 (4.37 to 5.35) | 233985.96 (166611.21 to 315520.30) | 1610560.26 (1139876.86 to 2174769.42) | 496.34 (349.75 to 663.74) | 1504.98 (1056.41 to 2037.20) | 4.34 (3.92 to 4.77) |
| Yemen | 457.01 (288.53 to 754.68) | 367.73 (238.41 to 553.94) | 8.14 (5.16 to 13.27) | 2.30 (1.46 to 3.43) | -4.67 (-4.99 to -4.34) | 21617.07 (15021.52 to 33410.65) | 25463.03 (18790.12 to 32962.44) | 304.48 (206.94 to 477.92) | 108.77 (79.72 to 143.06) | -3.75 (-3.99 to -3.50) |
| Zambia | 2348.89 (759.81 to 4643.01) | 4019.86 (2553.64 to 5935.94) | 74.19 (26.21 to 144.21) | 48.83 (30.91 to 72.06) | -1.96 (-2.25 to -1.67) | 95465.33 (32443.10 to 184515.80) | 181549.54 (120100.12 to 256366.56) | 2404.96 (861.27 to 4635.47) | 1645.77 (1100.83 to 2356.09) | -1.84 (-2.15 to -1.54) |
| Zimbabwe | 2067.80 (610.90 to 4030.98) | 4429.33 (1121.64 to 9239.35) | 45.59 (14.07 to 90.31) | 53.42 (15.07 to 111.28) | 0.89 (0.68 to 1.10) | 90283.00 (33375.29 to 163174.04) | 199144.87 (61654.00 to 393163.94) | 1588.85 (577.48 to 2913.24) | 1904.84 (605.22 to 3763.83) | 0.91 (0.66 to 1.16) |

ASDR, age-standardized Deaths rates; ASDALYR, age-standardized disability-adjusted life years rates; CI, confidence interval; DALYs, disability-adjusted life years rates; EAPC, estimated annual percentage change; UI, uncertainty interval.

Table S11 Deaths and DALYs of drug use in 1990 and 2021 at regional levels

| Region | Deaths (×1000, 95% UI) | | ASDR per 100,000 (95% UI) | | 1990-2021 EAPC,  (95% CI) | DALYs (×1000, 95% UI) | | ASDALYR per 100,000 (95% UI) | | 1990-2021 EAPC,  (95% CI) |
| --- | --- | --- | --- | --- | --- | --- | --- | --- | --- | --- |
|  | 1990 | 2021 | 1990 | 2021 |  | 1990 | 2021 | 1990 | 2021 |  |
| High SDI | 44.57 (38.71 to 52.18) | 158.73 (145.56 to 172.81) | 4.43 (3.85 to 5.20) | 11.21 (10.32 to 12.24) | 2.91 (2.77 to 3.05) | 3392.44 (2863.87 to 3931.57) | 10528.68 (9108.48 to 11876.63) | 351.55 (295.64 to 409.21) | 899.89 (767.73 to 1022.89) | 3.00 (2.75 to 3.25) |
| High-middle SDI | 47.52 (39.89 to 56.29) | 79.25 (68.71 to 89.37) | 4.41 (3.70 to 5.20) | 4.59 (4.03 to 5.14) | -0.32 (-0.65 to 0.01) | 3642.47 (3037.99 to 4255.95) | 4486.11 (3931.46 to 5046.19) | 322.86 (269.89 to 376.70) | 297.83 (258.31 to 337.69) | -0.75 (-1.10 to -0.40) |
| Middle SDI | 68.95 (57.33 to 83.91) | 122.63 (103.3 to 142.26) | 5.02 (4.13 to 6.16) | 4.45 (3.75 to 5.18) | -0.76 (-0.92 to -0.60) | 5103.06 (4303.08 to 5958.81) | 6729.83 (5790.24 to 7654.72) | 315.86 (268.04 to 372.90) | 248.77 (213.68 to 282.64) | -1.18 (-1.35 to -1.01) |
| Low-middle SDI | 27.97 (20.07 to 38.2) | 80.22 (62.92 to 98.66) | 3.52 (2.50 to 4.84) | 4.80 (3.76 to 5.95) | 0.59 (0.00 to 1.18) | 1798.74 (1426.19 to 2278.21) | 4466 (3685.36 to 5345.06) | 195.91 (155.05 to 247.74) | 243.30 (199.76 to 289.51) | 0.32 (-0.21 to 0.86) |
| Low SDI | 8.27 (6.05 to 10.95) | 22.24 (17.24 to 27.93) | 2.71 (1.97 to 3.63) | 3.23 (2.43 to 4.08) | -0.21 (-0.87 to 0.45) | 551.98 (436.71 to 682.31) | 1440.1 (1170.93 to 1740.77) | 154.52 (120.21 to 191.35) | 171.14 (138.75 to 208.52) | -0.36 (-0.94 to 0.22) |
| Andean Latin America | 0.41 (0.23 to 0.64) | 1.18 (0.74 to 1.72) | 1.55 (0.82 to 2.47) | 1.88 (1.14 to 2.76) | 0.42 (0.26 to 0.57) | 42.89 (31.49 to 54.6) | 93.01 (69.6 to 116.7) | 129.00 (93.30 to 168.66) | 136.34 (101.91 to 173.14) | 0.12 (0.01 to 0.23) |
| Australasia | 0.92 (0.64 to 1.15) | 2.81 (1.92 to 3.29) | 4.17 (2.93 to 5.25) | 7.21 (5.39 to 8.43) | 1.18 (0.75 to 1.61) | 98.12 (79.41 to 117.39) | 184.53 (153.82 to 213.45) | 451.98 (365.92 to 541.16) | 559.44 (472.82 to 652.43) | 0.25 (-0.04 to 0.54) |
| Caribbean | 0.87 (0.6 to 1.19) | 1.83 (1.26 to 2.49) | 2.94 (2.01 to 4.12) | 3.51 (2.42 to 4.74) | -0.70 (-1.31 to -0.10) | 69.02 (53.46 to 87.15) | 109.22 (82.98 to 135.6) | 209.25 (160.88 to 262.31) | 215.53 (164.15 to 266.31) | -0.88 (-1.36 to -0.41) |
| Central Asia | 3.3 (2.29 to 4.58) | 9.36 (7 to 12.01) | 6.30 (4.24 to 8.79) | 10.18 (7.50 to 13.07) | 1.36 (0.88 to 1.84) | 204.84 (158.19 to 259.63) | 478.19 (375.12 to 582.83) | 343.36 (262.28 to 440.89) | 487.10 (386.72 to 589.43) | 0.96 (0.51 to 1.41) |
| Central Europe | 4.08 (3.02 to 5.38) | 7.17 (5.61 to 8.9) | 2.83 (2.12 to 3.71) | 4.10 (3.24 to 4.96) | 0.78 (0.54 to 1.02) | 245.78 (201.83 to 299.88) | 325.1 (270.19 to 387.77) | 182.97 (151.08 to 223.42) | 234.23 (195.92 to 274.33) | 0.53 (0.37 to 0.69) |
| Central Latin America | 4.2 (2.72 to 5.93) | 12 (8.43 to 15.87) | 3.77 (2.40 to 5.42) | 4.54 (3.19 to 6.02) | 0.20 (0.06 to 0.34) | 298.21 (228.94 to 386.88) | 647.42 (507.21 to 797.63) | 224.99 (167.75 to 297.33) | 241.41 (189.21 to 297.55) | -0.05 (-0.15 to 0.05) |
| Central Sub-Saharan Africa | 0.62 (0.44 to 0.87) | 1.91 (1.34 to 2.68) | 1.81 (1.27 to 2.56) | 2.15 (1.50 to 3.05) | -0.69 (-1.50 to 0.14) | 47.97 (37.16 to 60.93) | 141.93 (107.42 to 183.14) | 119.63 (92.04 to 152.13) | 134.91 (100.68 to 177.26) | -0.56 (-1.20 to 0.08) |
| East Asia | 57.35 (46.75 to 69.84) | 57.39 (45.43 to 70.99) | 5.18 (4.23 to 6.27) | 2.82 (2.26 to 3.47) | -2.69 (-3.15 to -2.23) | 4613.89 (3799.19 to 5478.87) | 3146.73 (2616.28 to 3722.7) | 358.85 (299.55 to 425.95) | 185.17 (152.56 to 219.76) | -3.00 (-3.39 to -2.60) |
| Eastern Europe | 15.25 (12.91 to 18.25) | 35.98 (31.91 to 40.28) | 5.97 (5.06 to 7.14) | 13.89 (12.38 to 15.52) | 2.20 (1.37 to 3.05) | 1175.24 (966.27 to 1377.5) | 2121.95 (1884.8 to 2364.24) | 484.63 (398.96 to 566.69) | 911.11 (802.09 to 1020.06) | 1.53 (0.75 to 2.31) |
| Eastern Sub-Saharan Africa | 3.16 (2.23 to 4.33) | 9.4 (7.42 to 11.81) | 2.92 (2.10 to 3.90) | 3.65 (2.80 to 4.66) | -1.14 (-2.44 to 0.18) | 211.65 (163.58 to 268.13) | 586.4 (476.65 to 712.51) | 164.33 (124.97 to 210.80) | 189.81 (152.70 to 231.46) | -1.21 (-2.39 to -0.02) |
| High-income Asia Pacific | 5.02 (3.11 to 7.56) | 9.84 (7.03 to 13.13) | 2.41 (1.51 to 3.61) | 2.73 (2.01 to 3.51) | 0.03 (-0.26 to 0.31) | 345.24 (262.95 to 453.37) | 401.38 (321.68 to 495.15) | 175.93 (134.60 to 230.70) | 170.48 (139.80 to 209.34) | -0.25 (-0.43 to -0.07) |
| High-income North America | 23.91 (21.21 to 26.74) | 115.1 (104.76 to 126.03) | 7.48 (6.67 to 8.37) | 25.99 (23.69 to 28.57) | 4.18 (3.96 to 4.41) | 1799.13 (1524.62 to 2067.03) | 8135.69 (7027.69 to 9228.8) | 580.73 (491.00 to 668.97) | 2117.63 (1814.29 to 2416.00) | 4.46 (4.11 to 4.82) |
| North Africa and Middle East | 4.87 (4.12 to 5.87) | 16.57 (13.77 to 21.31) | 2.06 (1.72 to 2.55) | 2.95 (2.39 to 3.93) | 1.17 (0.89 to 1.44) | 529.02 (424.89 to 642.18) | 1416.05 (1167.5 to 1678.28) | 180.56 (148.04 to 217.77) | 221.39 (183.05 to 263.24) | 0.71 (0.48 to 0.95) |
| Oceania | 0.14 (0.07 to 0.26) | 0.32 (0.17 to 0.45) | 3.44 (1.62 to 6.19) | 3.32 (1.78 to 4.64) | -0.18 (-0.24 to -0.11) | 9.97 (6.75 to 14.99) | 21.88 (15.99 to 27.66) | 192.39 (122.39 to 302.72) | 180.82 (127.34 to 231.46) | -0.23 (-0.29 to -0.18) |
| South Asia | 29.51 (22.09 to 39.79) | 76.41 (58.02 to 96.61) | 3.88 (2.90 to 5.27) | 4.58 (3.47 to 5.79) | 0.55 (0.18 to 0.93) | 1754.16 (1394.02 to 2220.75) | 4093.19 (3262.55 to 5005.69) | 200.62 (158.76 to 254.80) | 223.11 (178.08 to 272.36) | 0.33 (-0.02 to 0.69) |
| Southeast Asia | 15.42 (10.25 to 22.9) | 39.28 (28.9 to 49.02) | 4.69 (3.04 to 6.97) | 5.42 (4.03 to 6.79) | 0.30 (-0.12 to 0.71) | 929.61 (694.91 to 1263) | 1961.56 (1572.89 to 2357.15) | 238.96 (173.24 to 333.16) | 259.89 (208.75 to 312.64) | 0.07 (-0.34 to 0.48) |
| Southern Latin America | 1.49 (0.58 to 2.48) | 3.06 (1.62 to 4.32) | 3.16 (1.23 to 5.29) | 3.77 (2.02 to 5.28) | 0.80 (0.62 to 0.98) | 110.67 (70.23 to 148.99) | 180.39 (128.55 to 225.52) | 229.72 (143.94 to 311.70) | 239.56 (173.66 to 295.31) | 0.19 (0.05 to 0.33) |
| Southern Sub-Saharan Africa | 1.17 (0.92 to 1.48) | 5.05 (4.29 to 6.06) | 3.00 (2.36 to 3.82) | 6.68 (5.64 to 7.99) | 1.09 (-1.00 to 3.22) | 117.08 (93.01 to 140.97) | 336.1 (275.37 to 403.63) | 257.60 (205.32 to 308.99) | 418.53 (345.75 to 501.99) | 0.58 (-1.08 to 2.26) |
| Tropical Latin America | 4.87 (3.45 to 6.4) | 12.63 (10.23 to 14.8) | 3.94 (2.77 to 5.25) | 4.82 (3.90 to 5.66) | -0.02 (-0.28 to 0.23) | 384.89 (309.1 to 472.15) | 763.26 (640.29 to 885.2) | 273.97 (218.59 to 337.13) | 300.28 (252.31 to 349.81) | -0.21 (-0.41 to -0.01) |
| Western Europe | 18.53 (14.65 to 23.09) | 32.32 (27.36 to 37.74) | 4.04 (3.25 to 4.96) | 4.86 (4.28 to 5.51) | 0.16 (0.00 to 0.32) | 1341.05 (1127.85 to 1547.87) | 1710.64 (1476.36 to 1929.12) | 319.81 (269.28 to 370.61) | 356.60 (305.12 to 405.73) | -0.08 (-0.24 to 0.09) |
| Western Sub-Saharan Africa | 2.31 (1.54 to 3.39) | 13.74 (10.7 to 17.46) | 1.91 (1.29 to 2.77) | 4.55 (3.57 to 5.74) | 0.53 (-0.95 to 2.03) | 170.1 (128.86 to 225.88) | 811.73 (653.73 to 1004.49) | 121.57 (91.39 to 161.92) | 238.85 (193.58 to 293.88) | 0.30 (-0.98 to 1.60) |

ASDR, age-standardized Deaths rates; ASDALYR, age-standardized disability-adjusted life years rates; CI, confidence interval; DALYs, disability-adjusted life years rates; EAPC, estimated annual percentage change; UI, uncertainty interval.

Table S12 Deaths and DALYs of drug use in 1990 and 2021 at the national levels

| Location | Deaths (95% UI) | | ASDR per 100,000 (95% UI) | | 1990-2021 EAPC,  (95% CI) | DALYs (95% UI) | | ASDALYR per 100,000 (95% UI) | | 1990-2021 EAPC,  (95% CI) |
| --- | --- | --- | --- | --- | --- | --- | --- | --- | --- | --- |
|  | 1990 | 2021 | 1990 | 2021 |  | 1990 | 2021 | 1990 | 2021 |  |
| Afghanistan | 178.97 (120.05 to 253.60) | 588.44 (437.60 to 787.95) | 2.51 (1.71 to 3.48) | 3.03 (2.18 to 4.17) | 0.50 (0.33 to 0.67) | 16163.63 (12695.26 to 20087.05) | 62069.15 (49527.65 to 75720.54) | 219.83 (172.53 to 273.74) | 243.37 (195.91 to 298.92) | 0.47 (0.31 to 0.63) |
| Albania | 66.81 (27.06 to 100.43) | 115.91 (35.26 to 183.60) | 2.59 (0.89 to 4.08) | 2.96 (1.07 to 4.51) | 0.44 (0.26 to 0.62) | 4882.94 (3571.65 to 6137.37) | 5976.48 (3951.77 to 7636.74) | 156.84 (103.37 to 205.31) | 187.98 (138.34 to 233.51) | 0.67 (0.51 to 0.84) |
| Algeria | 281.37 (200.86 to 373.99) | 1045.91 (705.17 to 1452.67) | 1.64 (1.17 to 2.20) | 2.48 (1.64 to 3.47) | 1.35 (1.21 to 1.50) | 33060.37 (25935.84 to 41253.15) | 90375.52 (71596.17 to 112070.99) | 151.41 (119.86 to 187.51) | 200.15 (158.62 to 248.82) | 0.93 (0.83 to 1.02) |
| American Samoa | 0.98 (0.22 to 1.47) | 1.97 (0.58 to 2.76) | 3.15 (0.61 to 4.82) | 3.94 (1.12 to 5.50) | 0.67 (0.53 to 0.82) | 73.89 (41.79 to 98.34) | 102.23 (53.63 to 132.89) | 185.37 (88.62 to 251.02) | 204.20 (109.43 to 262.84) | 0.28 (0.16 to 0.40) |
| Andorra | 1.53 (0.35 to 2.56) | 4.38 (0.97 to 7.38) | 2.52 (0.56 to 4.21) | 2.97 (0.71 to 4.91) | 0.62 (0.47 to 0.78) | 120.09 (71.98 to 166.00) | 209.98 (116.24 to 288.85) | 187.59 (111.53 to 260.49) | 191.52 (125.01 to 250.32) | 0.14 (0.07 to 0.21) |
| Angola | 65.52 (31.55 to 120.70) | 609.55 (400.53 to 892.51) | 1.09 (0.48 to 2.11) | 3.06 (2.00 to 4.51) | 3.28 (2.84 to 3.73) | 6519.45 (4562.67 to 8983.44) | 41373.95 (30112.00 to 54093.60) | 83.86 (56.02 to 122.33) | 179.28 (128.47 to 240.41) | 2.48 (2.18 to 2.78) |
| Antigua and Barbuda | 1.06 (0.50 to 1.80) | 2.55 (0.98 to 3.95) | 2.13 (0.98 to 3.67) | 2.30 (0.91 to 3.53) | -0.34 (-0.68 to -0.00) | 94.70 (67.81 to 125.97) | 166.16 (111.42 to 213.89) | 164.98 (113.60 to 224.04) | 160.14 (111.53 to 204.03) | -0.39 (-0.63 to -0.16) |
| Argentina | 762.55 (176.48 to 1419.61) | 1618.33 (472.49 to 2398.01) | 2.37 (0.55 to 4.41) | 3.10 (0.94 to 4.58) | 0.80 (0.58 to 1.01) | 63445.74 (37821.93 to 86948.43) | 107277.97 (67703.37 to 136708.52) | 198.24 (118.10 to 271.11) | 216.84 (138.70 to 274.91) | 0.14 (-0.03 to 0.31) |
| Armenia | 28.30 (11.12 to 51.39) | 64.63 (20.76 to 122.06) | 0.90 (0.34 to 1.62) | 1.65 (0.56 to 3.02) | 2.46 (1.71 to 3.20) | 5470.52 (3917.13 to 7131.40) | 5451.93 (3887.00 to 7521.22) | 153.20 (108.48 to 199.35) | 165.36 (117.59 to 220.44) | 0.47 (0.17 to 0.76) |
| Australia | 837.46 (566.84 to 1041.38) | 2558.98 (1659.89 to 3034.29) | 4.57 (3.11 to 5.68) | 7.90 (5.70 to 9.27) | 1.13 (0.67 to 1.59) | 88824.67 (71362.68 to 106111.50) | 166217.08 (137848.36 to 192774.94) | 491.70 (395.99 to 587.84) | 604.03 (508.03 to 706.65) | 0.22 (-0.10 to 0.53) |
| Austria | 271.31 (147.41 to 430.26) | 446.49 (243.99 to 607.64) | 3.02 (1.68 to 4.71) | 3.63 (2.29 to 4.70) | 0.04 (-0.26 to 0.36) | 25050.12 (18810.74 to 32359.17) | 30003.14 (23423.56 to 35943.22) | 292.44 (220.96 to 371.24) | 316.95 (256.72 to 372.08) | -0.09 (-0.34 to 0.16) |
| Azerbaijan | 263.37 (59.03 to 460.04) | 698.83 (132.16 to 1142.09) | 4.59 (0.96 to 8.10) | 6.38 (1.15 to 10.64) | 0.64 (0.27 to 1.01) | 18160.12 (10229.41 to 25492.96) | 34070.95 (16413.54 to 47228.67) | 270.17 (143.71 to 396.24) | 288.89 (139.39 to 405.60) | -0.11 (-0.35 to 0.14) |
| Bahamas | 7.47 (4.12 to 11.61) | 17.82 (9.09 to 25.51) | 3.62 (1.86 to 5.78) | 3.95 (2.03 to 5.64) | -1.39 (-2.09 to -0.69) | 591.17 (422.53 to 775.12) | 1078.92 (735.34 to 1365.87) | 241.86 (163.81 to 325.67) | 243.42 (168.83 to 306.37) | -1.27 (-1.83 to -0.70) |
| Bahrain | 5.68 (4.22 to 7.68) | 28.33 (17.63 to 42.78) | 1.51 (0.96 to 2.30) | 1.85 (0.96 to 3.07) | -0.20 (-0.62 to 0.23) | 820.45 (610.03 to 1036.42) | 2843.15 (2165.95 to 3633.00) | 146.40 (110.13 to 183.90) | 151.78 (114.50 to 196.47) | -0.17 (-0.39 to 0.05) |
| Bangladesh | 522.46 (318.63 to 763.06) | 1434.75 (794.16 to 2250.10) | 0.78 (0.46 to 1.14) | 0.93 (0.52 to 1.44) | 0.58 (0.49 to 0.66) | 64694.11 (48372.16 to 82514.27) | 139144.78 (103218.65 to 181699.16) | 73.61 (55.68 to 92.63) | 81.84 (60.72 to 107.72) | 0.43 (0.39 to 0.48) |
| Barbados | 4.94 (2.52 to 7.56) | 7.53 (3.09 to 11.71) | 2.03 (1.03 to 3.21) | 1.72 (0.79 to 2.56) | -1.46 (-1.89 to -1.03) | 450.14 (326.73 to 582.11) | 500.60 (353.73 to 641.80) | 173.29 (124.32 to 225.44) | 148.42 (110.49 to 186.32) | -1.04 (-1.30 to -0.78) |
| Belarus | 577.58 (370.22 to 757.75) | 1069.37 (729.99 to 1364.68) | 4.84 (3.22 to 6.33) | 8.31 (5.92 to 10.61) | 1.42 (0.47 to 2.38) | 38745.19 (30432.33 to 47231.88) | 53630.75 (41875.24 to 64746.81) | 343.75 (271.10 to 419.41) | 482.78 (384.37 to 574.93) | 0.86 (0.02 to 1.70) |
| Belgium | 238.83 (122.21 to 371.73) | 623.61 (317.82 to 844.19) | 1.97 (1.07 to 3.02) | 3.86 (2.36 to 4.97) | 1.55 (1.28 to 1.83) | 20199.05 (14904.29 to 25706.35) | 35303.67 (26628.06 to 42174.81) | 187.18 (140.67 to 235.30) | 284.08 (229.81 to 333.25) | 0.99 (0.81 to 1.17) |
| Belize | 2.33 (1.22 to 3.70) | 14.95 (6.88 to 22.27) | 2.16 (1.08 to 3.53) | 4.01 (1.74 to 6.03) | 1.52 (0.85 to 2.19) | 244.20 (177.74 to 313.94) | 991.08 (674.17 to 1259.70) | 168.63 (118.51 to 225.82) | 234.50 (154.70 to 303.96) | 0.80 (0.35 to 1.25) |
| Benin | 15.93 (4.56 to 31.16) | 133.80 (76.78 to 210.71) | 0.68 (0.18 to 1.36) | 1.80 (1.01 to 2.83) | 1.58 (-0.05 to 3.24) | 2035.93 (1321.67 to 2818.67) | 10561.18 (7553.41 to 13887.12) | 60.57 (39.44 to 87.40) | 111.17 (77.20 to 149.10) | 0.94 (-0.25 to 2.15) |
| Bermuda | 1.81 (1.20 to 2.54) | 2.09 (1.24 to 2.87) | 2.66 (1.74 to 3.73) | 2.16 (1.45 to 2.84) | -1.89 (-2.53 to -1.25) | 138.29 (104.76 to 171.26) | 124.96 (93.82 to 151.46) | 200.36 (151.18 to 249.53) | 177.26 (136.53 to 211.69) | -1.24 (-1.71 to -0.77) |
| Bhutan | 9.87 (2.29 to 23.97) | 23.99 (5.91 to 47.59) | 2.63 (0.60 to 6.36) | 3.40 (0.84 to 6.67) | 0.83 (0.74 to 0.92) | 715.02 (362.07 to 1371.19) | 1364.58 (631.16 to 2293.77) | 151.93 (66.98 to 307.16) | 172.79 (76.59 to 294.36) | 0.42 (0.33 to 0.51) |
| Bolivia (Plurinational State of) | 77.55 (30.60 to 135.09) | 231.26 (89.99 to 391.56) | 1.83 (0.65 to 3.40) | 2.25 (0.82 to 3.91) | 0.52 (0.42 to 0.62) | 8046.10 (5668.08 to 10549.10) | 18297.91 (13054.93 to 24417.31) | 151.33 (103.58 to 208.78) | 155.31 (107.76 to 212.09) | -0.05 (-0.15 to 0.05) |
| Bosnia and Herzegovina | 104.21 (24.15 to 177.31) | 126.20 (33.56 to 200.13) | 2.16 (0.50 to 3.69) | 2.24 (0.72 to 3.44) | -0.12 (-0.28 to 0.05) | 6552.92 (3718.95 to 9303.24) | 5471.27 (2997.05 to 7467.07) | 132.75 (74.94 to 189.39) | 128.67 (85.13 to 168.40) | -0.02 (-0.16 to 0.13) |
| Botswana | 25.79 (16.50 to 40.70) | 158.46 (118.98 to 215.78) | 3.16 (2.05 to 4.96) | 7.18 (5.35 to 9.91) | -0.91 (-3.08 to 1.30) | 2087.60 (1532.04 to 2863.74) | 9567.46 (7555.49 to 12563.04) | 215.21 (153.97 to 298.33) | 399.98 (317.43 to 520.26) | -0.91 (-2.81 to 1.04) |
| Brazil | 4826.13 (3424.84 to 6341.96) | 12357.09 (10011.06 to 14532.55) | 4.00 (2.80 to 5.33) | 4.84 (3.92 to 5.68) | -0.07 (-0.32 to 0.19) | 380143.59 (305800.33 to 466549.92) | 747206.92 (626707.61 to 867305.33) | 277.17 (221.19 to 340.60) | 302.77 (254.08 to 352.76) | -0.23 (-0.44 to -0.03) |
| Brunei Darussalam | 4.48 (2.19 to 6.58) | 12.84 (4.62 to 19.01) | 2.54 (1.02 to 4.17) | 2.82 (0.92 to 4.43) | 0.17 (-0.07 to 0.41) | 515.69 (382.92 to 639.54) | 980.80 (670.77 to 1233.82) | 209.75 (145.47 to 270.49) | 190.25 (125.96 to 245.10) | -0.49 (-0.67 to -0.31) |
| Bulgaria | 336.90 (95.01 to 527.74) | 535.78 (188.17 to 764.42) | 2.95 (0.95 to 4.49) | 4.98 (2.05 to 6.87) | 1.60 (1.32 to 1.87) | 18597.14 (9950.52 to 25305.82) | 23324.57 (12573.29 to 30570.69) | 189.43 (113.27 to 252.62) | 284.18 (183.80 to 358.38) | 1.22 (1.00 to 1.43) |
| Burkina Faso | 289.23 (177.64 to 461.65) | 168.96 (96.79 to 277.26) | 5.06 (3.12 to 7.97) | 1.41 (0.76 to 2.38) | -5.99 (-6.77 to -5.21) | 16660.81 (11062.14 to 24316.12) | 14252.13 (10364.76 to 19464.98) | 270.96 (178.62 to 402.83) | 90.99 (65.13 to 127.81) | -5.22 (-5.89 to -4.55) |
| Burundi | 72.89 (46.08 to 120.52) | 159.82 (84.47 to 264.36) | 2.20 (1.37 to 3.61) | 2.00 (1.04 to 3.21) | -2.25 (-3.13 to -1.36) | 5252.61 (3781.00 to 7646.50) | 11745.66 (8011.10 to 16918.36) | 132.62 (94.98 to 196.82) | 117.34 (78.68 to 173.99) | -1.99 (-2.72 to -1.25) |
| Côte d'Ivoire | 1.81 (0.91 to 3.09) | 6.23 (2.51 to 11.66) | 0.81 (0.39 to 1.40) | 1.19 (0.45 to 2.23) | -3.64 (-5.03 to -2.24) | 205.87 (149.11 to 271.03) | 519.49 (352.03 to 774.95) | 77.64 (54.15 to 105.47) | 87.82 (58.63 to 132.75) | -3.45 (-4.72 to -2.16) |
| Cabo Verde | 284.95 (28.82 to 670.12) | 1063.86 (318.50 to 1960.47) | 4.94 (0.46 to 11.91) | 7.10 (2.07 to 13.13) | -0.38 (-1.37 to 0.62) | 16610.33 (6384.26 to 32873.34) | 54550.85 (24633.03 to 92357.70) | 237.39 (74.63 to 491.26) | 334.19 (145.74 to 573.84) | -0.48 (-1.12 to 0.17) |
| Cambodia | 43.45 (20.50 to 74.51) | 376.60 (250.58 to 563.60) | 0.76 (0.35 to 1.33) | 1.97 (1.26 to 2.97) | 0.85 (-0.27 to 1.99) | 5455.78 (3817.48 to 7233.37) | 29648.33 (22362.05 to 38411.64) | 70.52 (48.59 to 93.73) | 124.15 (91.79 to 162.81) | 0.88 (-0.25 to 2.02) |
| Cameroon | 741.80 (607.39 to 978.95) | 4761.77 (4012.90 to 5408.65) | 2.37 (1.94 to 3.17) | 11.34 (9.86 to 12.85) | 2.43 (0.91 to 3.98) | 122561.62 (95443.80 to 149421.48) | 372246.35 (324464.27 to 423801.10) | 405.33 (315.67 to 496.09) | 990.45 (861.39 to 1125.22) | 1.57 (0.47 to 2.69) |
| Canada | 46.41 (28.14 to 73.40) | 170.95 (110.96 to 251.97) | 2.57 (1.52 to 4.09) | 4.63 (2.93 to 6.92) | 5.06 (4.62 to 5.51) | 3311.33 (2351.86 to 4649.04) | 10112.42 (7150.51 to 13797.73) | 160.66 (111.51 to 230.67) | 243.77 (168.43 to 336.38) | 2.92 (2.54 to 3.31) |
| Central African Republic | 36.15 (18.35 to 64.68) | 188.36 (95.22 to 332.93) | 1.05 (0.52 to 1.91) | 2.27 (1.07 to 4.08) | -0.84 (-2.35 to 0.69) | 3425.78 (2439.44 to 4692.91) | 13460.05 (9216.70 to 20095.07) | 82.06 (57.92 to 116.67) | 127.53 (84.04 to 196.04) | -0.98 (-2.30 to 0.36) |
| Chad | 677.58 (86.41 to 1225.06) | 1316.85 (184.99 to 1907.71) | 5.96 (0.70 to 11.01) | 5.37 (0.81 to 7.69) | 0.87 (-0.48 to 2.24) | 41623.33 (17538.83 to 62639.81) | 64262.34 (29833.35 to 81809.55) | 331.72 (128.73 to 516.26) | 290.33 (146.36 to 364.38) | 0.31 (-0.74 to 1.37) |
| Chile | 55978.88 (45643.91 to 67948.45) | 54762.20 (43331.58 to 67928.35) | 5.24 (4.29 to 6.36) | 2.79 (2.20 to 3.44) | 0.32 (0.08 to 0.56) | 4515493.98 (3720509.72 to 5370204.46) | 2998219.35 (2482505.34 to 3553239.62) | 363.29 (302.88 to 431.42) | 183.06 (150.52 to 217.02) | -0.01 (-0.17 to 0.16) |
| China | 340.41 (143.85 to 495.95) | 789.36 (253.09 to 1232.74) | 1.37 (0.50 to 2.12) | 1.44 (0.47 to 2.26) | -2.80 (-3.27 to -2.32) | 42067.02 (30042.96 to 53425.62) | 72894.98 (51631.02 to 91010.37) | 134.80 (93.38 to 170.55) | 134.88 (95.89 to 168.52) | -3.10 (-3.50 to -2.69) |
| Colombia | 3.55 (1.70 to 6.11) | 13.15 (7.10 to 21.40) | 1.39 (0.69 to 2.32) | 2.10 (1.15 to 3.39) | -0.36 (-0.53 to -0.20) | 295.44 (196.44 to 416.57) | 864.93 (592.90 to 1256.85) | 90.26 (58.88 to 129.77) | 122.51 (82.39 to 179.02) | -0.16 (-0.23 to -0.08) |
| Comoros | 43.14 (27.16 to 65.62) | 159.90 (108.06 to 227.27) | 2.82 (1.73 to 4.34) | 3.63 (2.39 to 5.21) | 0.98 (0.70 to 1.27) | 2994.80 (2154.79 to 4188.81) | 9892.87 (7139.34 to 13109.47) | 171.15 (120.67 to 242.81) | 203.47 (146.55 to 269.14) | 0.77 (0.56 to 0.99) |
| Congo | 0.25 (0.08 to 0.40) | 0.55 (0.16 to 0.84) | 1.75 (0.53 to 2.85) | 2.23 (0.74 to 3.36) | -1.17 (-2.08 to -0.25) | 21.87 (14.51 to 29.25) | 26.74 (16.34 to 35.08) | 125.80 (78.71 to 168.64) | 132.37 (87.64 to 168.86) | -1.06 (-1.83 to -0.29) |
| Cook Islands | 48.66 (12.04 to 78.75) | 191.20 (47.39 to 299.58) | 2.26 (0.51 to 3.83) | 3.49 (0.88 to 5.47) | 0.84 (0.75 to 0.92) | 4203.31 (2558.06 to 5665.59) | 10099.09 (5421.29 to 13727.77) | 158.58 (87.13 to 221.57) | 189.31 (104.33 to 254.53) | 0.23 (0.18 to 0.28) |
| Costa Rica | 371.97 (213.18 to 684.95) | 522.85 (369.31 to 732.54) | 4.92 (2.82 to 8.79) | 2.90 (1.96 to 4.24) | 0.69 (0.40 to 0.97) | 23093.21 (14875.84 to 38389.86) | 34231.99 (26140.86 to 43945.82) | 268.41 (170.83 to 456.15) | 161.45 (123.16 to 212.56) | 0.11 (-0.05 to 0.28) |
| Croatia | 250.66 (83.32 to 389.64) | 225.01 (89.07 to 312.29) | 4.10 (1.48 to 6.18) | 3.41 (1.64 to 4.57) | -0.63 (-0.99 to -0.27) | 14155.60 (8042.36 to 19125.26) | 11983.87 (8360.77 to 14783.59) | 252.94 (156.02 to 330.66) | 251.99 (189.22 to 300.89) | 0.08 (-0.33 to 0.50) |
| Cuba | 133.29 (63.67 to 224.98) | 335.74 (91.30 to 546.96) | 1.23 (0.57 to 2.10) | 1.92 (0.59 to 3.07) | 1.27 (0.89 to 1.65) | 16103.42 (11708.08 to 20859.53) | 19631.31 (11879.85 to 26995.78) | 134.17 (96.58 to 174.09) | 145.45 (97.52 to 190.41) | 0.15 (-0.00 to 0.30) |
| Cyprus | 13.06 (8.99 to 17.89) | 28.25 (17.63 to 39.01) | 1.67 (1.14 to 2.27) | 1.68 (1.10 to 2.26) | -0.12 (-0.29 to 0.06) | 1364.76 (1074.77 to 1661.72) | 2626.49 (2027.79 to 3275.88) | 167.14 (131.43 to 203.84) | 175.43 (136.75 to 217.04) | -0.06 (-0.19 to 0.08) |
| Czechia | 235.86 (99.50 to 390.63) | 441.86 (217.71 to 619.42) | 1.92 (0.87 to 3.13) | 2.75 (1.54 to 3.71) | 1.43 (1.22 to 1.63) | 16906.24 (11078.77 to 22903.96) | 24480.35 (16941.92 to 30630.76) | 155.72 (104.66 to 207.80) | 213.16 (158.03 to 262.96) | 1.43 (1.27 to 1.58) |
| Democratic People's Republic of Korea | 608.85 (254.23 to 1033.04) | 1133.61 (414.65 to 1777.37) | 3.08 (1.26 to 5.25) | 3.39 (1.29 to 5.24) | 0.30 (0.23 to 0.37) | 45082.21 (29897.99 to 62924.51) | 66950.26 (40783.53 to 91392.58) | 212.94 (139.65 to 299.66) | 213.53 (136.15 to 288.99) | 0.01 (-0.03 to 0.05) |
| Democratic Republic of the Congo | 450.09 (302.31 to 650.98) | 874.69 (414.58 to 1497.99) | 1.91 (1.27 to 2.70) | 1.51 (0.68 to 2.63) | -1.73 (-2.58 to -0.87) | 34014.46 (25082.03 to 44459.60) | 74273.49 (49232.04 to 108699.17) | 124.21 (90.91 to 164.46) | 104.16 (67.23 to 156.19) | -1.34 (-1.99 to -0.69) |
| Denmark | 276.17 (208.77 to 354.80) | 407.53 (291.71 to 500.94) | 4.71 (3.61 to 6.02) | 5.37 (4.18 to 6.29) | 0.13 (-0.06 to 0.33) | 22590.29 (18455.99 to 26801.91) | 26568.12 (21799.00 to 30683.52) | 405.52 (333.91 to 479.56) | 431.72 (361.32 to 495.89) | 0.03 (-0.09 to 0.15) |
| Djibouti | 3.43 (1.75 to 5.67) | 25.21 (12.76 to 41.74) | 1.41 (0.75 to 2.31) | 2.37 (1.25 to 4.01) | 1.50 (1.28 to 1.72) | 300.52 (207.56 to 420.78) | 1683.44 (1092.98 to 2549.06) | 91.98 (61.40 to 130.97) | 134.49 (87.46 to 204.57) | 1.13 (0.96 to 1.31) |
| Dominica | 1.00 (0.45 to 1.71) | 2.16 (0.92 to 3.24) | 1.78 (0.78 to 3.04) | 2.67 (1.20 to 3.92) | 0.82 (0.53 to 1.11) | 105.58 (77.18 to 138.04) | 141.40 (96.49 to 181.02) | 159.13 (114.18 to 213.46) | 189.58 (133.93 to 240.18) | 0.23 (0.02 to 0.44) |
| Dominican Republic | 99.88 (30.93 to 198.71) | 363.02 (107.71 to 631.06) | 2.10 (0.57 to 4.22) | 3.39 (0.98 to 5.92) | 0.56 (-0.23 to 1.36) | 8739.83 (5530.04 to 13154.25) | 21995.50 (12315.79 to 31698.24) | 144.31 (83.62 to 231.54) | 194.63 (106.06 to 282.30) | 0.25 (-0.37 to 0.88) |
| Ecuador | 98.36 (31.64 to 168.69) | 317.82 (138.05 to 484.49) | 1.44 (0.40 to 2.56) | 1.84 (0.77 to 2.83) | 0.86 (0.61 to 1.11) | 10848.58 (7476.33 to 14125.00) | 25046.55 (18712.15 to 32700.12) | 123.60 (80.81 to 167.02) | 135.57 (99.88 to 177.07) | 0.35 (0.18 to 0.52) |
| Egypt | 304.73 (93.41 to 554.50) | 1235.17 (415.66 to 2318.55) | 0.99 (0.26 to 1.83) | 1.74 (0.50 to 3.35) | 2.22 (1.97 to 2.47) | 55206.68 (39666.97 to 72221.27) | 141978.79 (101897.92 to 192193.41) | 113.52 (82.29 to 151.14) | 146.67 (102.82 to 203.98) | 0.99 (0.91 to 1.07) |
| El Salvador | 136.36 (43.86 to 224.80) | 270.70 (76.98 to 430.22) | 3.71 (1.09 to 6.39) | 4.42 (1.23 to 7.05) | 0.36 (0.13 to 0.58) | 9097.10 (4821.15 to 13253.99) | 14938.82 (7229.50 to 20711.51) | 219.00 (104.16 to 328.92) | 236.32 (110.94 to 329.68) | 0.09 (-0.07 to 0.26) |
| Equatorial Guinea | 2.83 (1.53 to 4.55) | 51.04 (30.43 to 90.10) | 1.07 (0.56 to 1.74) | 5.06 (3.09 to 8.85) | 5.21 (4.19 to 6.24) | 267.46 (199.96 to 354.02) | 3352.48 (2233.05 to 5441.31) | 85.42 (62.63 to 115.34) | 285.53 (186.10 to 469.92) | 4.21 (3.45 to 4.98) |
| Eritrea | 32.75 (18.61 to 56.81) | 125.24 (63.60 to 219.66) | 1.72 (0.99 to 2.97) | 2.62 (1.36 to 4.60) | 0.70 (0.26 to 1.15) | 2622.58 (1819.56 to 3904.00) | 8439.41 (5517.92 to 12914.59) | 108.70 (73.13 to 165.34) | 148.25 (93.57 to 228.76) | 0.49 (0.14 to 0.85) |
| Estonia | 106.48 (68.59 to 131.41) | 182.23 (127.31 to 222.54) | 5.91 (3.97 to 7.27) | 10.65 (8.24 to 12.74) | 1.43 (0.74 to 2.12) | 7365.72 (5944.88 to 8722.78) | 13066.49 (10734.34 to 15129.82) | 444.35 (357.28 to 523.62) | 997.18 (824.23 to 1165.30) | 2.37 (1.83 to 2.91) |
| Eswatini | 5.80 (3.40 to 9.35) | 80.90 (58.25 to 109.21) | 1.42 (0.85 to 2.28) | 9.15 (6.32 to 12.79) | 5.67 (2.56 to 8.88) | 730.71 (552.17 to 947.23) | 5017.63 (3832.00 to 6415.47) | 125.80 (94.09 to 167.38) | 509.77 (382.71 to 668.24) | 4.51 (1.95 to 7.12) |
| Ethiopia | 337.06 (227.91 to 474.40) | 892.99 (574.06 to 1304.47) | 1.22 (0.81 to 1.74) | 1.38 (0.89 to 2.00) | -1.04 (-1.84 to -0.23) | 29687.69 (22955.56 to 37532.05) | 75151.72 (55807.33 to 95934.87) | 84.76 (65.83 to 108.35) | 89.05 (65.53 to 115.35) | -0.87 (-1.46 to -0.27) |
| Fiji | 14.68 (3.25 to 23.46) | 24.58 (6.12 to 36.84) | 2.84 (0.54 to 4.83) | 2.91 (0.68 to 4.35) | -0.05 (-0.17 to 0.07) | 1123.85 (601.51 to 1510.30) | 1457.73 (810.68 to 1946.74) | 170.97 (81.38 to 240.25) | 158.82 (86.65 to 212.51) | -0.33 (-0.40 to -0.25) |
| Finland | 234.64 (182.77 to 311.60) | 504.73 (363.37 to 625.16) | 4.03 (3.16 to 5.36) | 7.43 (5.85 to 8.79) | 2.28 (1.99 to 2.58) | 18704.86 (15394.80 to 22682.57) | 33350.06 (27465.98 to 38305.30) | 340.34 (280.74 to 414.20) | 609.14 (519.40 to 696.98) | 2.13 (1.93 to 2.33) |
| France | 2940.24 (1075.37 to 4201.23) | 5924.69 (2574.67 to 7654.27) | 4.28 (1.61 to 5.96) | 5.19 (2.52 to 6.40) | 0.54 (0.38 to 0.69) | 171864.30 (107901.28 to 214150.86) | 238337.23 (161017.04 to 283112.76) | 271.25 (177.64 to 335.35) | 295.77 (225.60 to 344.09) | 0.45 (0.18 to 0.73) |
| Gabon | 9.78 (5.95 to 14.61) | 44.52 (29.52 to 63.33) | 1.43 (0.86 to 2.19) | 3.07 (2.03 to 4.43) | 1.12 (-0.07 to 2.31) | 862.33 (644.83 to 1121.57) | 2924.94 (2183.72 to 3933.21) | 109.54 (80.17 to 143.85) | 182.79 (135.76 to 245.59) | 0.74 (-0.16 to 1.66) |
| Gambia | 3.50 (1.44 to 6.55) | 56.08 (35.95 to 82.40) | 0.74 (0.28 to 1.39) | 3.83 (2.48 to 5.55) | 5.18 (4.01 to 6.35) | 492.88 (332.04 to 646.92) | 3527.12 (2497.88 to 4806.16) | 67.92 (45.02 to 92.02) | 204.93 (143.14 to 278.33) | 3.61 (2.80 to 4.43) |
| Georgia | 277.53 (64.12 to 488.20) | 316.71 (101.07 to 460.20) | 4.39 (1.06 to 7.64) | 6.25 (2.17 to 8.99) | 1.70 (1.41 to 1.99) | 16450.55 (8679.27 to 23984.92) | 14077.56 (7246.76 to 18600.92) | 273.62 (149.26 to 394.61) | 328.24 (188.14 to 422.35) | 1.16 (0.83 to 1.49) |
| Germany | 3870.90 (1840.46 to 5453.23) | 5979.95 (2931.00 to 7970.14) | 3.93 (1.99 to 5.47) | 4.50 (2.69 to 5.61) | -0.10 (-0.27 to 0.07) | 272546.03 (192865.89 to 344940.58) | 307527.02 (226740.61 to 368557.90) | 302.88 (222.73 to 374.03) | 320.28 (261.97 to 373.51) | -0.26 (-0.37 to -0.14) |
| Ghana | 108.57 (64.06 to 169.47) | 767.09 (547.87 to 1052.97) | 1.14 (0.64 to 1.79) | 3.01 (2.12 to 4.07) | 1.35 (0.11 to 2.61) | 10824.29 (7868.57 to 14185.52) | 51728.49 (38654.03 to 66484.98) | 93.85 (67.50 to 123.74) | 178.92 (133.45 to 228.99) | 0.88 (-0.05 to 1.82) |
| Greece | 179.49 (104.31 to 255.18) | 470.74 (296.19 to 587.99) | 1.49 (0.94 to 2.01) | 3.44 (2.54 to 4.03) | 1.80 (1.12 to 2.48) | 18387.69 (14424.22 to 22310.29) | 29811.34 (24581.72 to 34248.58) | 170.76 (134.65 to 203.38) | 297.83 (247.92 to 342.19) | 1.06 (0.43 to 1.70) |
| Greenland | 3.31 (1.60 to 7.44) | 4.53 (2.70 to 6.48) | 5.05 (2.61 to 10.83) | 6.63 (4.12 to 9.83) | 0.98 (0.87 to 1.09) | 328.99 (215.85 to 596.72) | 330.19 (247.79 to 442.67) | 479.44 (320.39 to 852.20) | 530.70 (400.85 to 723.60) | 0.38 (0.31 to 0.46) |
| Grenada | 1.39 (0.62 to 2.49) | 3.52 (1.50 to 5.37) | 2.25 (0.96 to 4.17) | 2.99 (1.31 to 4.51) | 0.15 (-0.32 to 0.63) | 125.53 (87.69 to 173.00) | 230.38 (156.22 to 297.73) | 175.56 (118.56 to 253.96) | 201.20 (139.38 to 258.94) | -0.03 (-0.34 to 0.28) |
| Guam | 4.78 (1.22 to 7.02) | 8.79 (2.08 to 11.93) | 4.48 (0.88 to 6.62) | 4.54 (1.20 to 6.15) | 0.26 (-0.00 to 0.53) | 312.64 (168.83 to 415.26) | 419.68 (197.20 to 538.75) | 238.60 (111.13 to 320.05) | 238.09 (121.90 to 304.78) | 0.14 (-0.05 to 0.33) |
| Guatemala | 288.09 (93.00 to 519.29) | 987.11 (259.53 to 1638.22) | 5.74 (1.68 to 10.43) | 7.54 (1.76 to 12.82) | 0.48 (0.16 to 0.80) | 17163.80 (8431.83 to 27532.91) | 51798.77 (24175.11 to 76265.61) | 303.36 (134.48 to 503.86) | 361.17 (147.86 to 545.26) | 0.24 (-0.03 to 0.51) |
| Guinea | 29.99 (12.81 to 56.99) | 178.06 (112.73 to 274.10) | 0.79 (0.33 to 1.53) | 2.35 (1.44 to 3.73) | 2.55 (1.30 to 3.81) | 2928.09 (2012.29 to 4055.12) | 12016.88 (8568.51 to 16276.86) | 65.40 (43.58 to 92.05) | 131.10 (92.87 to 181.72) | 1.67 (0.73 to 2.62) |
| Guinea-Bissau | 6.01 (2.10 to 11.17) | 60.97 (31.86 to 107.50) | 1.17 (0.38 to 2.24) | 4.89 (2.58 to 8.47) | 4.28 (3.35 to 5.22) | 543.28 (356.63 to 805.06) | 3654.16 (2215.52 to 6031.76) | 79.03 (48.68 to 123.75) | 251.64 (151.43 to 417.91) | 3.53 (2.78 to 4.29) |
| Guyana | 20.78 (9.01 to 37.08) | 42.11 (16.34 to 71.21) | 3.96 (1.59 to 7.14) | 5.69 (2.17 to 9.69) | 1.02 (0.50 to 1.53) | 1493.95 (967.22 to 2205.64) | 2312.80 (1312.34 to 3416.96) | 232.67 (139.64 to 364.95) | 300.65 (166.53 to 447.31) | 0.66 (0.21 to 1.12) |
| Haiti | 345.29 (182.63 to 545.51) | 624.34 (308.23 to 952.57) | 7.78 (4.10 to 12.34) | 6.23 (2.77 to 10.03) | -2.16 (-2.84 to -1.49) | 20147.75 (12922.76 to 30446.25) | 36787.08 (24342.94 to 48334.44) | 413.52 (255.45 to 624.36) | 319.83 (198.35 to 437.82) | -2.15 (-2.76 to -1.52) |
| Honduras | 120.79 (39.09 to 213.34) | 402.78 (108.18 to 750.48) | 4.27 (1.23 to 7.75) | 5.35 (1.27 to 10.19) | 0.67 (0.59 to 0.75) | 8018.03 (4463.58 to 11720.16) | 22535.84 (11759.13 to 33921.17) | 244.67 (121.69 to 382.72) | 256.47 (121.12 to 409.25) | 0.00 (-0.07 to 0.07) |
| Hungary | 270.73 (83.02 to 491.92) | 295.78 (84.15 to 513.55) | 2.19 (0.71 to 3.92) | 2.12 (0.72 to 3.47) | -1.54 (-1.99 to -1.10) | 19145.08 (11244.51 to 28384.13) | 18138.33 (10508.86 to 25258.24) | 173.23 (106.43 to 247.40) | 173.36 (115.37 to 227.75) | -0.77 (-1.02 to -0.52) |
| Iceland | 9.36 (7.30 to 11.30) | 30.04 (23.64 to 34.62) | 3.48 (2.71 to 4.21) | 7.14 (5.93 to 8.12) | 2.11 (1.84 to 2.38) | 846.48 (696.82 to 995.38) | 2099.64 (1808.65 to 2366.00) | 313.22 (258.03 to 368.55) | 576.05 (491.97 to 651.72) | 1.88 (1.67 to 2.09) |
| India | 26033.20 (19755.83 to 33928.08) | 64186.48 (47719.90 to 81738.96) | 4.22 (3.21 to 5.53) | 4.86 (3.61 to 6.20) | 0.52 (0.10 to 0.94) | 1504917.88 (1206147.42 to 1858971.55) | 3354671.93 (2696903.21 to 4038584.02) | 214.26 (170.08 to 265.88) | 233.02 (186.07 to 280.61) | 0.29 (-0.12 to 0.69) |
| Indonesia | 3788.39 (2058.47 to 6783.90) | 8987.19 (5594.20 to 14070.88) | 3.03 (1.66 to 5.36) | 3.38 (2.13 to 5.23) | 0.42 (0.29 to 0.55) | 229600.23 (154083.67 to 358198.08) | 452962.93 (325475.12 to 646425.50) | 150.02 (97.25 to 239.61) | 152.35 (108.65 to 215.72) | 0.04 (-0.04 to 0.11) |
| Iran (Islamic Republic of) | 2319.70 (1987.12 to 2806.35) | 4714.49 (4253.21 to 5238.81) | 6.10 (5.17 to 7.50) | 5.16 (4.64 to 5.80) | -0.49 (-0.74 to -0.25) | 185978.76 (157265.01 to 218699.02) | 385018.60 (328161.85 to 440275.32) | 407.35 (345.47 to 481.39) | 407.93 (347.30 to 470.39) | 0.00 (-0.33 to 0.34) |
| Iraq | 213.05 (157.17 to 291.97) | 901.90 (592.38 to 1343.90) | 1.79 (1.28 to 2.53) | 2.65 (1.68 to 3.89) | 1.02 (0.85 to 1.19) | 23904.58 (18508.36 to 29990.56) | 78650.25 (60096.98 to 101547.87) | 158.82 (124.58 to 197.83) | 193.84 (145.49 to 253.21) | 0.52 (0.37 to 0.68) |
| Ireland | 76.19 (52.35 to 96.60) | 303.06 (220.98 to 355.29) | 2.11 (1.46 to 2.68) | 5.19 (4.06 to 5.94) | 2.40 (1.84 to 2.97) | 7873.47 (6383.48 to 9440.25) | 23303.68 (19632.92 to 26629.04) | 220.42 (178.74 to 264.29) | 470.09 (396.18 to 539.87) | 1.89 (1.48 to 2.30) |
| Israel | 101.05 (50.99 to 141.99) | 274.85 (140.05 to 358.66) | 2.18 (1.09 to 3.03) | 2.58 (1.42 to 3.25) | 0.16 (-0.21 to 0.54) | 8714.26 (6764.76 to 10529.76) | 18824.21 (15101.89 to 22325.04) | 183.08 (141.08 to 221.80) | 198.65 (162.25 to 235.80) | 0.00 (-0.28 to 0.29) |
| Italy | 4213.28 (3270.30 to 5227.02) | 3904.43 (2855.88 to 4995.02) | 5.88 (4.73 to 7.08) | 3.33 (2.62 to 4.05) | -2.44 (-2.71 to -2.18) | 266921.32 (228328.94 to 309166.52) | 160745.59 (134986.59 to 188444.12) | 418.53 (357.84 to 480.50) | 219.52 (180.86 to 258.58) | -2.76 (-2.95 to -2.57) |
| Jamaica | 24.18 (14.21 to 34.97) | 55.30 (32.73 to 77.61) | 1.41 (0.81 to 2.08) | 1.80 (1.07 to 2.54) | 0.13 (-0.52 to 0.79) | 3109.49 (2360.00 to 3950.89) | 4852.61 (3742.91 to 5987.73) | 144.20 (111.49 to 182.63) | 156.11 (120.86 to 192.86) | -0.16 (-0.55 to 0.23) |
| Japan | 3782.87 (2347.12 to 5657.84) | 8073.81 (5669.40 to 10846.06) | 2.25 (1.40 to 3.35) | 3.09 (2.29 to 3.97) | 0.64 (0.31 to 0.98) | 241403.35 (184976.04 to 319446.53) | 290917.68 (234443.35 to 358952.20) | 164.82 (126.93 to 214.93) | 176.00 (145.15 to 213.64) | 0.07 (-0.14 to 0.29) |
| Jordan | 14.15 (7.18 to 24.69) | 73.84 (26.50 to 131.71) | 0.68 (0.30 to 1.26) | 0.75 (0.25 to 1.39) | -0.25 (-0.56 to 0.06) | 3689.23 (2642.21 to 4940.17) | 14566.81 (10384.44 to 19287.02) | 112.68 (82.80 to 147.55) | 112.55 (80.76 to 150.01) | -0.00 (-0.11 to 0.10) |
| Kazakhstan | 1016.02 (399.03 to 1372.66) | 2699.32 (1149.46 to 3460.56) | 7.23 (2.63 to 9.98) | 14.20 (5.82 to 18.39) | 1.97 (1.23 to 2.70) | 62406.53 (42520.30 to 77140.81) | 141098.42 (91927.71 to 169411.71) | 399.74 (261.42 to 496.93) | 713.07 (465.64 to 855.10) | 1.55 (0.76 to 2.33) |
| Kenya | 1072.32 (666.34 to 1768.68) | 2711.55 (2147.00 to 3534.64) | 8.15 (5.13 to 13.15) | 7.83 (6.02 to 10.41) | -2.83 (-4.78 to -0.83) | 61823.49 (41380.95 to 97849.85) | 145963.51 (118237.08 to 183173.14) | 424.43 (282.98 to 666.35) | 382.05 (301.60 to 490.89) | -2.95 (-4.83 to -1.03) |
| Kiribati | 5.48 (3.08 to 7.82) | 11.88 (7.12 to 16.43) | 9.84 (5.01 to 14.55) | 11.75 (6.60 to 16.30) | 0.58 (0.51 to 0.66) | 318.13 (214.84 to 425.28) | 654.92 (458.83 to 876.22) | 496.22 (309.63 to 689.47) | 575.08 (383.69 to 770.80) | 0.48 (0.41 to 0.55) |
| Kuwait | 10.39 (6.78 to 14.91) | 64.18 (42.40 to 88.44) | 0.81 (0.47 to 1.24) | 1.30 (0.79 to 1.85) | 1.08 (0.23 to 1.94) | 2383.95 (1765.60 to 3126.13) | 8346.44 (6491.46 to 10309.40) | 122.52 (91.40 to 158.33) | 144.97 (112.07 to 179.55) | 0.50 (0.20 to 0.80) |
| Kyrgyzstan | 293.91 (101.56 to 436.32) | 715.03 (294.35 to 954.61) | 8.92 (2.86 to 13.47) | 12.14 (4.63 to 16.28) | 0.99 (0.28 to 1.71) | 16504.40 (9634.11 to 21677.88) | 37075.71 (22118.04 to 47248.63) | 453.33 (248.41 to 605.65) | 576.31 (327.87 to 737.83) | 0.76 (0.10 to 1.42) |
| Lao People's Democratic Republic | 182.59 (27.91 to 375.85) | 331.02 (69.42 to 573.95) | 7.23 (1.06 to 15.14) | 5.98 (1.18 to 10.23) | -0.84 (-0.94 to -0.74) | 9083.58 (3070.63 to 15877.29) | 16586.79 (7369.51 to 25634.49) | 318.17 (94.60 to 583.26) | 252.21 (101.72 to 397.79) | -0.93 (-1.03 to -0.84) |
| Latvia | 117.21 (88.91 to 151.46) | 201.08 (123.31 to 257.01) | 3.94 (3.03 to 5.09) | 8.65 (5.92 to 10.55) | 1.73 (1.27 to 2.19) | 9587.25 (7813.41 to 11320.30) | 11478.59 (8689.19 to 13537.91) | 342.98 (279.51 to 404.75) | 580.24 (463.45 to 667.97) | 1.03 (0.70 to 1.35) |
| Lebanon | 28.02 (16.84 to 42.85) | 87.29 (46.84 to 138.58) | 1.14 (0.69 to 1.73) | 1.42 (0.75 to 2.29) | 0.81 (0.64 to 0.99) | 3712.95 (2771.26 to 4715.83) | 11010.94 (8368.07 to 13741.39) | 132.58 (99.41 to 168.28) | 178.98 (135.62 to 224.05) | 1.10 (1.02 to 1.18) |
| Lesotho | 17.66 (11.31 to 27.01) | 177.01 (137.35 to 231.34) | 1.74 (1.13 to 2.66) | 12.23 (9.28 to 16.40) | 4.63 (2.28 to 7.02) | 1687.37 (1297.09 to 2154.27) | 10020.53 (8054.53 to 12545.89) | 144.90 (112.29 to 186.58) | 637.26 (512.69 to 815.77) | 3.76 (1.81 to 5.76) |
| Liberia | 10.97 (4.58 to 20.10) | 79.19 (47.08 to 123.63) | 0.78 (0.30 to 1.43) | 2.21 (1.24 to 3.48) | 2.95 (1.47 to 4.45) | 1290.54 (916.44 to 1749.11) | 5740.06 (4083.08 to 7683.18) | 69.25 (48.13 to 95.13) | 130.67 (90.67 to 180.50) | 1.96 (0.85 to 3.08) |
| Libya | 53.11 (39.35 to 69.02) | 313.57 (199.93 to 479.42) | 1.86 (1.36 to 2.46) | 4.17 (2.61 to 6.35) | 2.92 (2.80 to 3.04) | 5866.97 (4612.53 to 7242.88) | 24433.71 (18741.68 to 32970.93) | 166.03 (130.67 to 203.28) | 303.14 (232.23 to 405.38) | 2.24 (2.15 to 2.33) |
| Lithuania | 136.74 (93.99 to 184.75) | 324.30 (163.98 to 442.27) | 3.38 (2.37 to 4.48) | 9.63 (5.57 to 12.49) | 3.38 (2.67 to 4.08) | 10999.71 (8619.30 to 13495.99) | 19900.51 (14042.55 to 24400.33) | 281.43 (220.83 to 343.90) | 697.06 (527.07 to 832.12) | 2.92 (2.55 to 3.29) |
| Luxembourg | 26.56 (12.68 to 34.58) | 43.54 (20.99 to 55.29) | 5.68 (2.92 to 7.27) | 4.77 (2.59 to 5.89) | -1.01 (-1.22 to -0.80) | 1798.06 (1314.15 to 2140.70) | 2504.00 (1924.53 to 2900.06) | 413.66 (311.68 to 490.76) | 339.94 (275.78 to 392.83) | -1.28 (-1.56 to -1.01) |
| Madagascar | 89.09 (47.42 to 147.38) | 455.07 (193.77 to 886.64) | 1.33 (0.72 to 2.23) | 2.46 (1.03 to 4.75) | 2.06 (1.98 to 2.14) | 7653.99 (5614.48 to 10748.79) | 31146.33 (19028.84 to 51457.12) | 88.36 (61.87 to 125.59) | 138.63 (80.84 to 234.64) | 1.52 (1.49 to 1.56) |
| Malawi | 160.60 (104.61 to 231.12) | 426.10 (277.88 to 619.11) | 2.81 (1.80 to 3.99) | 3.62 (2.32 to 5.30) | -1.53 (-2.85 to -0.18) | 11163.76 (8088.20 to 14703.78) | 27410.66 (19840.06 to 36604.36) | 163.33 (116.25 to 215.30) | 195.20 (139.91 to 263.86) | -1.39 (-2.54 to -0.22) |
| Malaysia | 392.57 (233.04 to 557.66) | 2084.25 (1322.20 to 2564.98) | 3.05 (1.66 to 4.51) | 6.40 (3.89 to 8.03) | 2.07 (1.02 to 3.14) | 29061.72 (21999.86 to 37310.78) | 110544.52 (86255.11 to 128659.03) | 188.33 (134.87 to 247.08) | 321.37 (245.44 to 377.39) | 1.42 (0.47 to 2.37) |
| Maldives | 8.30 (2.19 to 12.02) | 16.81 (8.26 to 23.28) | 8.06 (2.05 to 11.99) | 4.16 (1.71 to 5.66) | -2.46 (-2.62 to -2.30) | 410.28 (185.67 to 544.71) | 1019.27 (713.16 to 1322.99) | 309.54 (115.14 to 421.34) | 180.33 (112.50 to 234.30) | -1.94 (-2.12 to -1.77) |
| Mali | 44.26 (17.17 to 87.69) | 274.34 (137.64 to 455.37) | 0.91 (0.34 to 1.86) | 2.33 (1.11 to 4.01) | 2.07 (1.00 to 3.16) | 4332.35 (2991.80 to 5982.91) | 18835.97 (12765.69 to 26399.40) | 70.20 (46.44 to 101.00) | 125.13 (78.90 to 187.82) | 1.24 (0.41 to 2.08) |
| Malta | 8.27 (3.47 to 11.54) | 20.06 (9.24 to 26.36) | 2.01 (0.89 to 2.76) | 3.07 (1.82 to 3.77) | 1.33 (1.15 to 1.52) | 747.03 (571.91 to 912.68) | 1278.25 (1030.94 to 1505.50) | 191.76 (147.19 to 233.26) | 275.85 (225.30 to 321.34) | 1.18 (0.83 to 1.52) |
| Marshall Islands | 1.50 (0.27 to 2.57) | 2.91 (0.66 to 4.80) | 6.48 (0.99 to 11.12) | 6.28 (1.22 to 10.37) | -0.14 (-0.18 to -0.10) | 94.13 (41.95 to 139.81) | 162.84 (74.49 to 237.69) | 324.26 (115.95 to 496.86) | 304.65 (128.02 to 450.61) | -0.25 (-0.27 to -0.22) |
| Mauritania | 7.98 (2.03 to 17.65) | 21.36 (4.15 to 48.00) | 0.70 (0.17 to 1.56) | 0.87 (0.16 to 1.90) | 0.52 (0.31 to 0.74) | 1001.47 (679.41 to 1454.54) | 2340.14 (1520.28 to 3473.39) | 64.30 (41.07 to 98.11) | 68.30 (42.00 to 105.81) | 0.18 (0.11 to 0.24) |
| Mauritius | 65.10 (12.58 to 91.55) | 115.90 (68.19 to 138.60) | 7.57 (1.38 to 10.77) | 6.96 (4.30 to 8.24) | -1.11 (-1.40 to -0.81) | 3604.50 (1678.63 to 4673.70) | 5811.70 (4323.69 to 6754.13) | 359.03 (151.03 to 474.93) | 382.36 (294.77 to 442.22) | -0.35 (-0.55 to -0.14) |
| Mexico | 2853.27 (1801.28 to 4278.52) | 8097.52 (5686.97 to 10750.00) | 5.04 (3.11 to 7.61) | 5.92 (4.13 to 7.87) | 0.07 (-0.11 to 0.24) | 184147.46 (135756.03 to 251942.73) | 407315.87 (314551.25 to 512942.37) | 277.24 (196.73 to 388.15) | 293.57 (226.68 to 370.03) | -0.16 (-0.30 to -0.01) |
| Micronesia (Federated States of) | 3.72 (0.67 to 5.94) | 5.00 (1.29 to 7.86) | 6.02 (0.91 to 9.65) | 5.55 (1.33 to 8.63) | -0.31 (-0.39 to -0.23) | 227.72 (96.94 to 335.75) | 279.27 (135.83 to 409.39) | 312.16 (110.72 to 466.21) | 280.18 (130.18 to 407.79) | -0.40 (-0.47 to -0.33) |
| Monaco | 0.89 (0.16 to 1.53) | 2.62 (0.37 to 4.26) | 1.89 (0.43 to 3.07) | 3.50 (0.79 to 5.35) | 2.09 (1.76 to 2.42) | 64.04 (39.34 to 85.33) | 107.21 (54.37 to 148.39) | 187.32 (124.99 to 243.93) | 233.90 (151.17 to 304.56) | 0.78 (0.62 to 0.94) |
| Mongolia | 148.60 (17.11 to 294.67) | 533.88 (74.02 to 889.95) | 12.89 (1.23 to 26.30) | 21.33 (2.46 to 37.26) | 1.81 (1.35 to 2.26) | 7260.16 (2988.08 to 12016.81) | 20933.66 (6279.98 to 32238.29) | 519.94 (160.75 to 935.56) | 716.20 (193.71 to 1129.60) | 1.18 (0.81 to 1.56) |
| Montenegro | 11.68 (4.15 to 17.61) | 22.58 (7.63 to 32.69) | 1.77 (0.63 to 2.67) | 2.46 (0.93 to 3.50) | 1.14 (0.96 to 1.32) | 877.29 (589.11 to 1132.14) | 1067.91 (633.34 to 1353.57) | 132.53 (89.23 to 171.16) | 143.82 (94.26 to 179.29) | 0.36 (0.24 to 0.47) |
| Morocco | 348.93 (253.75 to 465.25) | 1242.32 (736.53 to 1891.75) | 1.81 (1.31 to 2.47) | 3.29 (1.95 to 4.99) | 1.96 (1.53 to 2.39) | 39603.18 (31373.64 to 49604.78) | 89146.00 (66580.40 to 115563.63) | 170.22 (134.20 to 212.88) | 228.50 (170.56 to 296.82) | 1.02 (0.73 to 1.30) |
| Mozambique | 140.58 (41.73 to 282.78) | 944.09 (436.32 to 1384.62) | 1.86 (0.52 to 3.62) | 5.72 (2.24 to 9.18) | 4.08 (3.16 to 5.02) | 9711.92 (5605.55 to 15848.23) | 55027.42 (33397.17 to 73529.07) | 104.04 (55.12 to 174.28) | 270.39 (150.55 to 374.98) | 3.57 (2.75 to 4.40) |
| Myanmar | 2136.10 (169.89 to 4384.81) | 4485.54 (1330.55 to 6722.08) | 7.44 (0.54 to 15.53) | 8.01 (2.30 to 11.98) | 0.08 (-0.57 to 0.73) | 113741.31 (32317.50 to 204747.24) | 219439.38 (97498.39 to 302510.42) | 354.37 (81.89 to 662.15) | 380.35 (167.07 to 525.57) | 0.11 (-0.53 to 0.75) |
| Namibia | 14.55 (9.18 to 22.29) | 97.57 (71.02 to 133.31) | 1.71 (1.09 to 2.63) | 5.09 (3.66 to 7.18) | 1.67 (-0.59 to 3.98) | 1594.97 (1219.38 to 2120.14) | 6545.27 (5212.83 to 8355.82) | 145.03 (110.61 to 196.62) | 303.64 (239.21 to 392.12) | 1.11 (-0.69 to 2.95) |
| Nauru | 0.45 (0.09 to 0.74) | 0.50 (0.14 to 0.83) | 6.75 (1.25 to 10.99) | 6.43 (1.67 to 10.40) | -0.31 (-0.46 to -0.15) | 26.30 (11.79 to 39.95) | 29.39 (15.20 to 43.85) | 333.37 (128.58 to 511.01) | 314.22 (141.50 to 472.60) | -0.28 (-0.41 to -0.16) |
| Nepal | 232.64 (87.69 to 458.83) | 738.27 (320.36 to 1160.57) | 1.78 (0.69 to 3.48) | 2.76 (1.18 to 4.36) | 1.81 (1.55 to 2.08) | 18825.71 (11771.56 to 28543.09) | 45578.33 (27682.34 to 62469.76) | 121.83 (72.50 to 190.43) | 152.23 (90.15 to 210.37) | 1.02 (0.77 to 1.26) |
| Netherlands | 209.17 (114.30 to 301.21) | 480.52 (257.45 to 636.68) | 1.21 (0.67 to 1.76) | 2.00 (1.29 to 2.55) | 1.32 (1.10 to 1.53) | 26589.11 (20334.37 to 33416.65) | 35733.44 (27910.89 to 43014.07) | 160.68 (122.72 to 201.95) | 201.10 (160.39 to 243.85) | 0.36 (0.16 to 0.57) |
| New Zealand | 79.46 (46.97 to 117.35) | 246.31 (166.86 to 301.06) | 2.16 (1.30 to 3.19) | 3.73 (2.72 to 4.62) | 1.58 (1.45 to 1.72) | 9298.53 (6947.78 to 12131.37) | 18312.84 (14791.59 to 22092.54) | 255.42 (191.46 to 332.84) | 335.00 (268.92 to 409.10) | 0.51 (0.37 to 0.64) |
| Nicaragua | 51.31 (8.34 to 95.04) | 212.73 (23.44 to 377.41) | 2.39 (0.33 to 4.61) | 3.63 (0.37 to 6.60) | 1.47 (1.20 to 1.74) | 4445.21 (2426.47 to 6559.33) | 12802.80 (5201.55 to 18695.81) | 162.84 (75.17 to 255.24) | 197.60 (73.29 to 299.99) | 0.73 (0.54 to 0.93) |
| Niger | 32.05 (11.87 to 62.25) | 137.53 (47.11 to 288.60) | 0.84 (0.28 to 1.67) | 1.28 (0.41 to 2.61) | 0.39 (-0.71 to 1.50) | 3699.87 (2483.37 to 5232.20) | 12891.45 (8269.81 to 19531.17) | 68.47 (43.63 to 100.45) | 84.10 (49.86 to 136.38) | 0.03 (-0.77 to 0.83) |
| Nigeria | 1243.99 (811.20 to 1913.80) | 10428.81 (7695.76 to 13903.02) | 2.10 (1.38 to 3.19) | 7.22 (5.41 to 9.38) | 1.29 (-0.41 to 3.02) | 86053.87 (62814.74 to 118352.49) | 571242.10 (437695.12 to 746227.27) | 128.65 (93.58 to 178.44) | 363.29 (279.51 to 469.50) | 1.03 (-0.49 to 2.58) |
| Niue | 0.08 (0.02 to 0.14) | 0.09 (0.03 to 0.13) | 4.03 (0.89 to 6.71) | 4.19 (1.39 to 6.46) | -0.14 (-0.22 to -0.07) | 4.59 (2.33 to 6.58) | 4.04 (2.09 to 5.56) | 225.31 (112.02 to 325.19) | 218.93 (120.68 to 299.81) | -0.32 (-0.39 to -0.25) |
| North Macedonia | 49.64 (12.64 to 73.01) | 96.72 (22.73 to 144.30) | 2.43 (0.61 to 3.60) | 3.06 (0.80 to 4.49) | 0.63 (0.40 to 0.85) | 3306.72 (1953.16 to 4280.58) | 4594.06 (2594.61 to 5950.69) | 158.31 (93.20 to 205.30) | 169.75 (108.90 to 214.16) | 0.52 (0.27 to 0.77) |
| Northern Mariana Islands | 1.72 (0.37 to 2.89) | 3.00 (0.73 to 4.23) | 5.19 (0.91 to 8.75) | 5.42 (1.38 to 7.69) | 0.04 (-0.24 to 0.31) | 118.84 (61.07 to 175.46) | 143.57 (66.56 to 189.78) | 268.89 (117.48 to 401.71) | 262.78 (132.04 to 347.16) | -0.13 (-0.36 to 0.10) |
| Norway | 158.95 (135.49 to 185.61) | 428.85 (380.40 to 472.28) | 3.30 (2.85 to 3.88) | 6.13 (5.55 to 6.73) | 1.10 (0.17 to 2.03) | 15242.36 (12296.21 to 18094.89) | 30696.79 (26278.94 to 35088.53) | 340.23 (273.19 to 404.89) | 532.38 (450.31 to 614.99) | 0.47 (-0.36 to 1.30) |
| Oman | 11.85 (7.00 to 19.83) | 46.13 (24.56 to 77.74) | 0.97 (0.48 to 1.77) | 1.21 (0.51 to 2.19) | 1.04 (0.70 to 1.38) | 2109.03 (1555.02 to 2787.94) | 6621.65 (4910.18 to 8681.67) | 116.48 (84.74 to 156.58) | 121.49 (88.43 to 161.69) | 0.30 (0.09 to 0.52) |
| Pakistan | 2715.13 (1109.71 to 6088.93) | 10025.13 (4284.46 to 20421.36) | 3.97 (1.60 to 8.93) | 6.00 (2.65 to 11.87) | 0.99 (0.80 to 1.17) | 165012.04 (95711.74 to 299817.87) | 552425.70 (293070.99 to 1072564.20) | 208.68 (118.14 to 392.37) | 278.73 (144.40 to 534.71) | 0.72 (0.57 to 0.87) |
| Palau | 0.51 (0.07 to 0.84) | 1.04 (0.31 to 1.55) | 4.19 (0.61 to 6.81) | 4.37 (1.36 to 6.34) | 0.13 (0.07 to 0.20) | 32.81 (15.54 to 47.53) | 51.86 (25.57 to 71.60) | 229.70 (93.70 to 338.74) | 236.64 (129.76 to 318.51) | 0.11 (0.08 to 0.14) |
| Palestine | 4.97 (1.16 to 10.98) | 25.52 (7.28 to 51.27) | 0.50 (0.10 to 1.13) | 0.82 (0.19 to 1.67) | 1.54 (1.40 to 1.68) | 1800.73 (1218.73 to 2426.38) | 5461.26 (3749.33 to 7173.89) | 107.70 (74.54 to 143.81) | 110.60 (76.23 to 146.18) | 0.11 (0.04 to 0.19) |
| Panama | 34.99 (12.10 to 54.41) | 108.15 (35.02 to 168.53) | 1.95 (0.58 to 3.12) | 2.45 (0.80 to 3.82) | 0.53 (0.38 to 0.68) | 3175.63 (2126.57 to 4103.00) | 6785.94 (4375.53 to 8760.59) | 145.17 (94.67 to 192.36) | 154.03 (99.50 to 198.72) | 0.07 (-0.05 to 0.18) |
| Papua New Guinea | 71.89 (8.08 to 148.57) | 182.03 (49.50 to 277.33) | 2.85 (0.28 to 5.89) | 2.66 (0.62 to 4.11) | -0.36 (-0.49 to -0.23) | 5433.20 (2867.20 to 8742.62) | 14108.76 (9299.11 to 18319.15) | 167.57 (74.59 to 283.78) | 156.59 (90.52 to 209.53) | -0.29 (-0.38 to -0.19) |
| Paraguay | 45.49 (17.49 to 74.32) | 268.16 (127.61 to 397.50) | 1.67 (0.60 to 2.82) | 4.11 (1.85 to 6.20) | 3.02 (2.72 to 3.32) | 4744.91 (3200.59 to 6200.52) | 16056.26 (10664.13 to 20511.48) | 141.10 (93.36 to 187.39) | 224.68 (144.14 to 287.82) | 1.62 (1.42 to 1.82) |
| Peru | 236.76 (100.60 to 380.92) | 634.13 (278.81 to 972.01) | 1.53 (0.58 to 2.56) | 1.79 (0.76 to 2.77) | 0.16 (-0.09 to 0.41) | 23997.02 (16722.29 to 31955.40) | 49663.14 (34881.00 to 64631.35) | 125.25 (81.49 to 170.83) | 130.90 (90.59 to 170.72) | 0.05 (-0.07 to 0.18) |
| Philippines | 1176.44 (733.00 to 1831.89) | 4025.80 (2963.05 to 5181.54) | 3.02 (1.86 to 4.90) | 4.27 (3.14 to 5.48) | 1.13 (1.04 to 1.22) | 82912.83 (61549.27 to 112383.37) | 213330.90 (171755.18 to 259877.03) | 168.00 (124.04 to 237.49) | 199.53 (158.27 to 243.58) | 0.56 (0.50 to 0.63) |
| Poland | 1044.35 (809.24 to 1320.40) | 2539.21 (2051.54 to 3053.26) | 2.48 (1.93 to 3.12) | 4.60 (3.76 to 5.54) | 1.66 (1.40 to 1.92) | 71898.18 (58551.12 to 89249.37) | 118950.77 (99575.56 to 140807.62) | 181.65 (146.49 to 226.49) | 261.80 (218.50 to 308.94) | 0.86 (0.69 to 1.03) |
| Portugal | 509.86 (190.98 to 780.89) | 579.80 (209.48 to 809.19) | 4.38 (1.80 to 6.44) | 3.38 (1.55 to 4.44) | -2.10 (-2.59 to -1.61) | 30834.94 (19371.77 to 40747.44) | 27348.48 (17487.04 to 34098.71) | 284.99 (187.58 to 367.49) | 220.20 (165.25 to 265.57) | -1.96 (-2.40 to -1.51) |
| Puerto Rico | 152.04 (91.74 to 229.87) | 212.29 (138.42 to 292.34) | 4.24 (2.55 to 6.41) | 5.00 (3.58 to 6.43) | -2.09 (-3.21 to -0.97) | 11934.69 (8868.10 to 15352.31) | 11269.68 (8749.74 to 13804.12) | 328.54 (243.29 to 423.72) | 318.79 (258.29 to 377.64) | -2.32 (-3.28 to -1.35) |
| Qatar | 3.64 (2.18 to 5.78) | 31.08 (14.18 to 53.56) | 1.21 (0.54 to 2.18) | 1.54 (0.47 to 2.98) | 0.29 (-0.15 to 0.73) | 654.67 (487.34 to 842.70) | 4822.66 (3463.80 to 6408.89) | 126.48 (92.90 to 166.72) | 128.19 (90.77 to 177.57) | -0.06 (-0.25 to 0.12) |
| Republic of Korea | 1190.21 (294.76 to 2209.74) | 1682.25 (487.10 to 2687.82) | 2.97 (0.70 to 5.63) | 1.96 (0.66 to 3.02) | -1.62 (-1.72 to -1.52) | 98119.60 (57014.68 to 139305.94) | 101904.20 (61915.66 to 134478.33) | 209.35 (114.99 to 309.37) | 160.81 (111.77 to 207.84) | -0.97 (-1.03 to -0.91) |
| Republic of Moldova | 676.36 (151.08 to 1094.22) | 758.82 (232.51 to 1047.54) | 14.47 (3.28 to 23.25) | 14.07 (4.71 to 19.33) | -0.33 (-0.89 to 0.23) | 30496.48 (12848.90 to 43532.14) | 30438.03 (14014.12 to 39999.26) | 656.39 (280.02 to 932.99) | 628.76 (332.51 to 801.06) | -0.35 (-0.86 to 0.15) |
| Romania | 758.54 (286.85 to 1378.47) | 1508.95 (348.96 to 2432.68) | 2.84 (1.15 to 5.01) | 5.20 (1.43 to 8.01) | 1.03 (0.59 to 1.48) | 41072.01 (24498.40 to 63385.69) | 59756.94 (23875.92 to 86970.22) | 162.99 (101.58 to 245.17) | 243.71 (120.26 to 337.47) | 0.53 (0.18 to 0.88) |
| Russian Federation | 9972.89 (8410.20 to 12313.58) | 26406.23 (23787.47 to 29003.20) | 5.92 (4.98 to 7.35) | 14.81 (13.38 to 16.25) | 2.35 (1.55 to 3.15) | 844712.97 (695697.91 to 1006052.61) | 1588692.49 (1421031.30 to 1759190.52) | 524.44 (429.80 to 626.45) | 984.61 (874.06 to 1097.31) | 1.42 (0.65 to 2.21) |
| Rwanda | 82.88 (51.72 to 126.03) | 231.26 (132.62 to 364.06) | 2.02 (1.23 to 3.03) | 2.41 (1.35 to 3.77) | -1.19 (-1.97 to -0.41) | 6203.47 (4573.53 to 8314.78) | 15976.26 (11059.04 to 22535.22) | 121.48 (87.74 to 165.11) | 139.67 (95.15 to 196.32) | -0.91 (-1.53 to -0.28) |
| Saint Kitts and Nevis | 0.68 (0.20 to 1.35) | 2.77 (1.24 to 4.32) | 2.21 (0.62 to 4.41) | 3.59 (1.62 to 5.56) | 1.41 (1.06 to 1.75) | 59.21 (37.50 to 84.52) | 155.00 (101.27 to 204.50) | 165.79 (96.37 to 252.61) | 213.16 (143.35 to 276.05) | 0.68 (0.40 to 0.97) |
| Saint Lucia | 2.28 (0.82 to 3.99) | 5.44 (1.59 to 8.75) | 2.48 (0.86 to 4.44) | 2.32 (0.71 to 3.68) | -0.67 (-0.98 to -0.35) | 218.90 (150.12 to 297.84) | 353.27 (219.20 to 469.63) | 188.69 (121.46 to 264.56) | 168.96 (110.60 to 219.76) | -0.50 (-0.66 to -0.35) |
| Saint Vincent and the Grenadines | 1.80 (0.94 to 2.82) | 4.43 (1.63 to 6.46) | 2.46 (1.23 to 3.93) | 3.22 (1.25 to 4.63) | 0.36 (-0.14 to 0.86) | 162.96 (117.17 to 212.84) | 259.42 (163.12 to 333.55) | 180.39 (125.87 to 241.16) | 205.10 (134.85 to 259.11) | 0.04 (-0.32 to 0.39) |
| Samoa | 3.81 (0.79 to 6.10) | 6.67 (1.53 to 9.97) | 3.68 (0.67 to 6.02) | 4.15 (0.87 to 6.14) | 0.31 (0.13 to 0.49) | 266.52 (147.19 to 382.01) | 402.34 (223.47 to 542.04) | 207.03 (97.41 to 299.84) | 219.55 (114.41 to 298.72) | 0.12 (-0.02 to 0.25) |
| San Marino | 0.56 (0.11 to 0.90) | 1.02 (0.14 to 1.75) | 1.87 (0.40 to 2.93) | 1.68 (0.32 to 2.83) | 0.27 (0.01 to 0.52) | 49.08 (34.20 to 63.61) | 61.43 (39.61 to 81.45) | 183.24 (130.58 to 236.65) | 170.35 (124.01 to 222.27) | 0.02 (-0.10 to 0.14) |
| Sao Tome and Principe | 0.58 (0.16 to 1.22) | 2.03 (0.57 to 4.46) | 0.83 (0.21 to 1.75) | 1.38 (0.35 to 3.03) | 1.51 (1.29 to 1.73) | 63.21 (43.57 to 93.84) | 176.99 (111.78 to 273.19) | 72.80 (47.60 to 110.40) | 93.55 (55.25 to 153.04) | 0.71 (0.61 to 0.80) |
| Saudi Arabia | 119.98 (55.01 to 236.23) | 849.11 (469.98 to 1384.80) | 1.26 (0.50 to 2.68) | 2.24 (1.01 to 3.90) | 2.24 (1.88 to 2.60) | 19268.41 (13767.62 to 25960.91) | 81444.24 (58895.06 to 107638.74) | 136.99 (96.01 to 193.61) | 173.58 (120.53 to 234.28) | 1.04 (0.82 to 1.27) |
| Senegal | 32.96 (13.86 to 63.45) | 135.93 (58.85 to 254.23) | 0.82 (0.32 to 1.63) | 1.38 (0.57 to 2.60) | 1.05 (0.19 to 1.93) | 3900.99 (2741.78 to 5344.96) | 11390.39 (7832.26 to 16151.16) | 71.71 (48.87 to 102.32) | 91.12 (60.85 to 133.13) | 0.42 (-0.17 to 1.02) |
| Serbia | 518.98 (208.70 to 701.02) | 707.16 (308.14 to 964.49) | 4.43 (1.87 to 5.93) | 4.77 (2.47 to 6.42) | -0.08 (-0.36 to 0.21) | 23874.41 (14194.28 to 29901.90) | 25896.08 (17496.37 to 33042.63) | 215.43 (136.51 to 265.95) | 221.16 (161.92 to 279.36) | -0.03 (-0.29 to 0.22) |
| Seychelles | 3.36 (0.72 to 5.73) | 8.96 (3.66 to 12.29) | 5.97 (1.21 to 10.27) | 7.09 (2.88 to 9.76) | 0.49 (0.20 to 0.79) | 188.55 (85.21 to 278.45) | 423.87 (235.86 to 536.67) | 301.29 (120.26 to 466.89) | 342.96 (199.42 to 429.91) | 0.39 (0.12 to 0.65) |
| Sierra Leone | 15.77 (4.89 to 30.41) | 79.82 (38.86 to 133.16) | 0.66 (0.19 to 1.31) | 1.49 (0.67 to 2.51) | 2.74 (1.78 to 3.71) | 2164.19 (1462.99 to 2905.04) | 6895.04 (4752.44 to 9480.55) | 65.38 (42.83 to 92.47) | 98.74 (63.71 to 140.55) | 1.47 (0.87 to 2.07) |
| Singapore | 40.03 (11.62 to 64.81) | 74.14 (23.29 to 122.08) | 1.36 (0.36 to 2.29) | 0.89 (0.31 to 1.45) | -1.53 (-1.80 to -1.25) | 5202.47 (3772.38 to 6822.99) | 7576.31 (5571.14 to 9702.91) | 144.72 (101.24 to 194.58) | 118.87 (88.43 to 153.91) | -0.74 (-0.85 to -0.63) |
| Slovakia | 247.63 (70.27 to 392.47) | 321.13 (94.31 to 455.66) | 4.36 (1.26 to 6.93) | 3.88 (1.32 to 5.44) | -0.11 (-0.34 to 0.11) | 14502.58 (8024.66 to 20094.30) | 14802.46 (8193.02 to 19107.46) | 263.12 (147.98 to 362.17) | 220.99 (140.80 to 280.03) | -0.45 (-0.66 to -0.24) |
| Slovenia | 120.53 (35.99 to 176.60) | 127.50 (47.53 to 178.22) | 5.07 (1.61 to 7.39) | 3.63 (1.76 to 4.87) | -1.47 (-1.84 to -1.09) | 6082.08 (3040.35 to 8100.33) | 5929.07 (3831.11 to 7276.90) | 270.65 (146.20 to 355.55) | 257.12 (196.88 to 307.41) | -0.12 (-0.50 to 0.26) |
| Solomon Islands | 11.57 (2.02 to 22.28) | 33.70 (7.03 to 51.13) | 6.12 (0.84 to 11.72) | 7.23 (1.17 to 11.01) | 0.58 (0.51 to 0.64) | 687.21 (263.63 to 1150.70) | 1859.69 (801.47 to 2593.75) | 301.76 (97.47 to 530.76) | 333.29 (124.15 to 473.15) | 0.35 (0.30 to 0.40) |
| Somalia | 65.32 (28.51 to 126.30) | 269.95 (107.98 to 500.97) | 1.50 (0.66 to 2.85) | 2.36 (0.92 to 4.27) | 1.38 (1.16 to 1.60) | 5387.40 (3541.25 to 8387.00) | 19203.46 (11196.61 to 30235.88) | 95.29 (60.13 to 149.03) | 130.64 (70.43 to 211.93) | 0.96 (0.80 to 1.13) |
| South Africa | 742.01 (580.03 to 1022.73) | 3860.87 (3264.26 to 4668.36) | 2.46 (1.95 to 3.27) | 6.62 (5.62 to 7.96) | 2.61 (0.32 to 4.96) | 85828.52 (67412.20 to 106229.68) | 261882.61 (217059.78 to 310700.79) | 247.20 (196.18 to 301.04) | 430.07 (356.72 to 508.75) | 1.47 (-0.25 to 3.21) |
| South Sudan | 46.13 (25.92 to 76.95) | 159.73 (79.01 to 285.52) | 1.44 (0.83 to 2.43) | 2.77 (1.32 to 4.99) | 2.01 (1.57 to 2.44) | 3934.60 (2856.84 to 5440.90) | 10117.82 (6432.05 to 15395.61) | 92.88 (65.20 to 133.29) | 146.45 (90.19 to 227.73) | 1.45 (1.11 to 1.80) |
| Spain | 2885.08 (1469.97 to 3741.25) | 4030.14 (1807.89 to 5124.25) | 6.40 (3.55 to 8.05) | 4.95 (2.66 to 6.03) | -1.78 (-2.12 to -1.44) | 192860.73 (143886.49 to 226115.60) | 175092.48 (127377.75 to 205099.40) | 462.07 (356.38 to 537.03) | 322.14 (255.27 to 378.07) | -2.35 (-2.72 to -1.97) |
| Sri Lanka | 921.80 (288.93 to 1577.93) | 908.79 (236.15 to 1504.80) | 6.52 (1.87 to 11.74) | 3.46 (0.95 to 5.65) | -2.83 (-3.29 to -2.36) | 54193.90 (27387.65 to 84394.53) | 46650.21 (24455.85 to 65458.50) | 339.68 (158.52 to 530.76) | 190.70 (106.71 to 261.45) | -2.64 (-3.05 to -2.23) |
| Sudan | 281.38 (176.38 to 474.14) | 3045.33 (1678.38 to 6943.71) | 2.17 (1.29 to 3.80) | 11.73 (5.51 to 30.06) | 4.70 (3.78 to 5.63) | 27041.71 (20388.67 to 37147.64) | 172303.04 (116322.55 to 321519.54) | 167.63 (123.92 to 237.38) | 493.30 (314.50 to 994.02) | 3.11 (2.55 to 3.68) |
| Suriname | 9.63 (4.80 to 15.83) | 25.81 (12.20 to 40.17) | 3.12 (1.50 to 5.19) | 3.99 (1.92 to 6.13) | 0.35 (-0.13 to 0.82) | 718.58 (493.65 to 1017.71) | 1484.43 (964.37 to 2007.03) | 204.24 (135.78 to 294.22) | 238.75 (160.33 to 317.77) | 0.16 (-0.22 to 0.54) |
| Sweden | 194.58 (145.78 to 246.18) | 592.34 (470.22 to 709.66) | 1.98 (1.53 to 2.43) | 4.95 (4.15 to 5.85) | 3.67 (3.34 to 4.01) | 14830.34 (12193.26 to 17661.01) | 48244.02 (40249.65 to 56132.67) | 165.70 (136.96 to 196.11) | 479.79 (400.03 to 560.84) | 4.01 (3.77 to 4.25) |
| Switzerland | 597.66 (428.25 to 718.31) | 576.84 (312.71 to 718.17) | 7.39 (5.51 to 8.85) | 4.18 (2.69 to 4.99) | -2.06 (-2.20 to -1.93) | 51427.29 (42631.82 to 59478.59) | 32213.23 (25491.80 to 37340.92) | 681.91 (567.05 to 788.75) | 328.62 (272.32 to 379.81) | -2.83 (-3.01 to -2.66) |
| Syrian Arab Republic | 98.02 (67.82 to 141.68) | 292.19 (159.88 to 475.43) | 1.29 (0.85 to 1.98) | 2.14 (1.20 to 3.40) | 1.92 (1.72 to 2.11) | 13468.71 (10332.29 to 16938.58) | 22484.91 (16472.10 to 29718.86) | 131.74 (101.34 to 168.02) | 164.89 (123.34 to 215.40) | 0.87 (0.78 to 0.97) |
| Taiwan (Province of China) | 761.78 (340.97 to 1260.73) | 1494.87 (507.63 to 2385.04) | 3.93 (1.65 to 6.60) | 4.20 (1.64 to 6.38) | 0.23 (0.04 to 0.42) | 53310.17 (36843.24 to 74277.36) | 81555.92 (49892.73 to 107918.37) | 248.68 (165.34 to 355.91) | 277.77 (187.33 to 353.80) | 0.20 (0.13 to 0.28) |
| Tajikistan | 184.74 (45.86 to 311.68) | 500.82 (138.17 to 738.78) | 5.81 (1.28 to 10.14) | 6.89 (1.61 to 10.76) | 0.10 (-0.29 to 0.49) | 12367.28 (6896.77 to 17212.88) | 29488.37 (15560.37 to 39422.95) | 317.70 (154.64 to 464.02) | 331.05 (158.46 to 451.28) | -0.27 (-0.56 to 0.02) |
| Thailand | 3633.34 (1973.11 to 4787.26) | 7835.73 (3570.58 to 10694.44) | 7.30 (3.44 to 9.92) | 8.07 (3.92 to 10.76) | -0.62 (-1.29 to 0.07) | 228115.63 (163686.89 to 285324.80) | 356122.55 (222902.78 to 446193.89) | 404.73 (268.33 to 513.60) | 419.28 (279.52 to 516.40) | -0.91 (-1.63 to -0.19) |
[truncated: 24,005 more chars]
